# Facile Entry to Pharmaceutically Important 3-Difluoromethyl-Quinoxalin-2-Ones Enabled by Visible-Light-Driven Difluoromethylation of Quinoxalin-2-Ones

**DOI:** 10.3390/ph15121552

**Published:** 2022-12-13

**Authors:** Kai-Zhong Fu, Xu-Xin Chen, Ya-Shi Zhao, Yuan-Qing Gu, Guo-Kai Liu

**Affiliations:** 1 School of Pharmaceutical Sciences, Shenzhen University Health Science Centre, Shenzhen University, Shenzhen 518060, China; 2 Shenzhen Key Laboratory for Nano-Biosensing Technology, Shenzhen 518060, China

**Keywords:** 3-CF_2_H-quinoxaline-2-ones, difluoromethylation, *S*-(difluoromethyl)sulfonium salt, fluorine-containing pharmaceuticals

## Abstract

CF_2_H moiety has a significant potential utility in drug design and discovery, and the incorporation of CF_2_H into biologically active molecules represents an important and efficient strategy for seeking lead compounds and drug candidates. On the other hand, quinoxalin-2-one is of great interest to pharmaceutical chemists as a common skeleton frequently occurring in plenty of natural products and bioactive compounds. Herein, we reported a practical and efficient protocol for the synthesis of 3-CF_2_H-quinoxalin-2-ones. Thus, in the presence of 3 mol% of photocatalyst and *S*-(difluoromethyl)sulfonium salt as difluoromethyl radical sources, a wide range of quinoxalin-2-ones readily underwent a visible-light redox-catalyzed difluoromethylation reaction, to deliver structurally diverse 3-difluoromethyl-quinoxalin-2-ones. We believe that this would facilitate increasing chances and possibilities for seeking potential lead compounds and drug candidates and further boost the development of fluorine-containing pharmaceuticals.

## 1. Introduction

**Figure 1:**
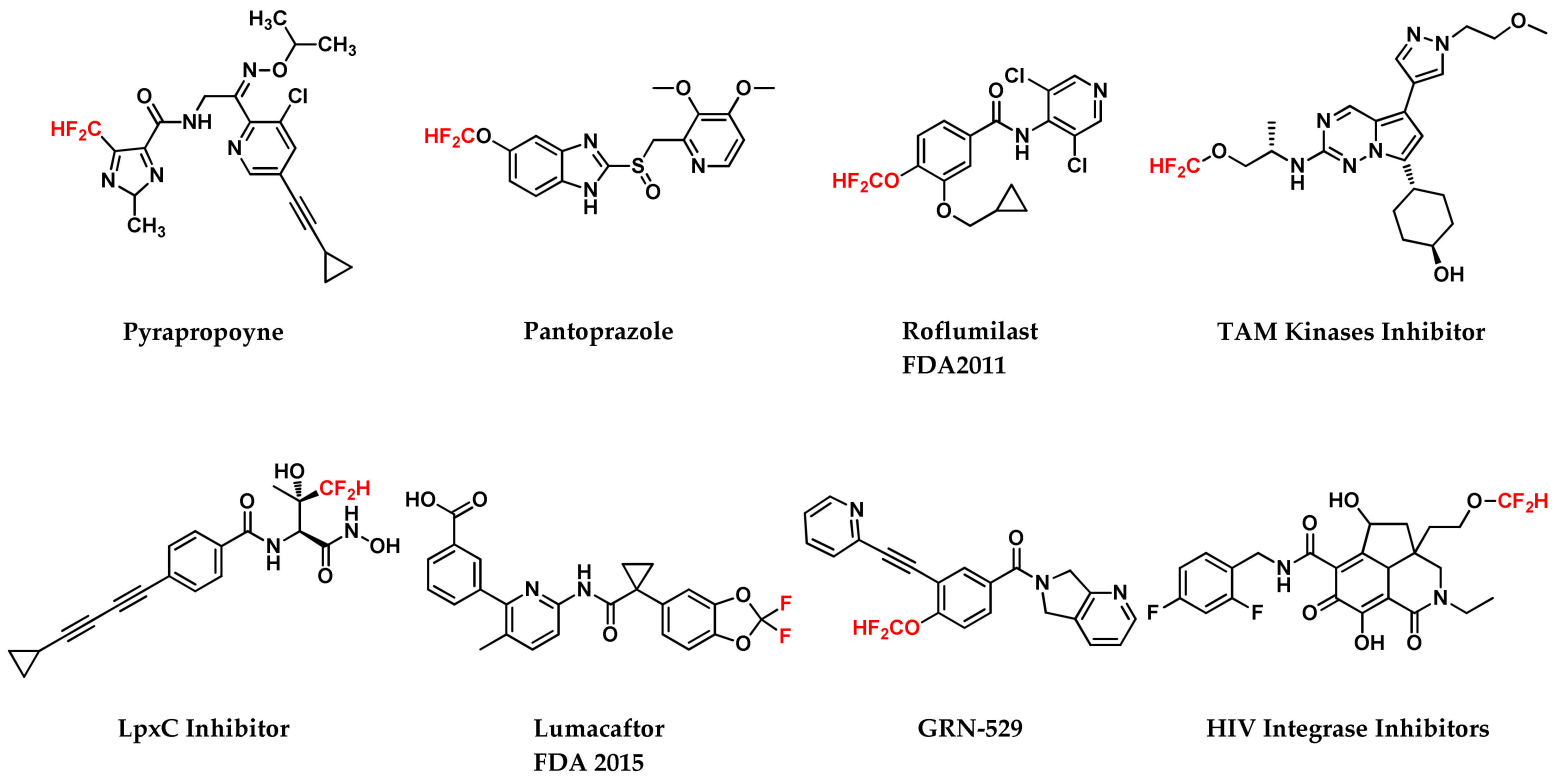
Selected examples of CF_2_H-containing pharmaceuticals and drug candidates over the past two decades.

The incorporation of fluorine and fluorinated moieties into organic molecules is of significant interest in drug design [1,2,3,4,5], agrochemicals [6,7,8], pharmaceuticals [9,10,11,12,13], and material science [14,15,16], due to the fact that fluorine can give the original molecules improved chemical, physical, and biological properties [2,10,17,18,19]. Particularly, thanks to an excellent lipophilicity, improved metabolic stability of fluorine-containing bioactive molecules that facilitates bioavailability, and better cell membrane permeability [2,3,19], it is becoming a routine tactic in new drug research and discovery to integrate fluorine or fluorine-containing structural motifs into bioactive molecules or lead compounds. As a result, the proportion of new molecule entities approved by the FDA has been higher and higher in recent years [20]. Notably, the difluoromethyl group (CF_2_H) is an important unit and plays a vital role in drug design and discovery, since CF_2_H can serve as a good hydrogen bond donor to enhance drug target affinity and target specificity as well as contribute to improved lipophilicity [21,22,23,24]. More importantly, the difluoromethyl group (CF_2_H) also constitutes a metabolically stable bioisosteres of alcohol, thiol, or amine groups, which are known to be three of the most common pharmacophores families in pharmaceuticals [3,25,26,27]. Subsequently, more and more pharmaceuticals and drug candidates bearing a CF_2_H structural motif have emerged over the past two decades (Figure 1).

**Scheme 1:**
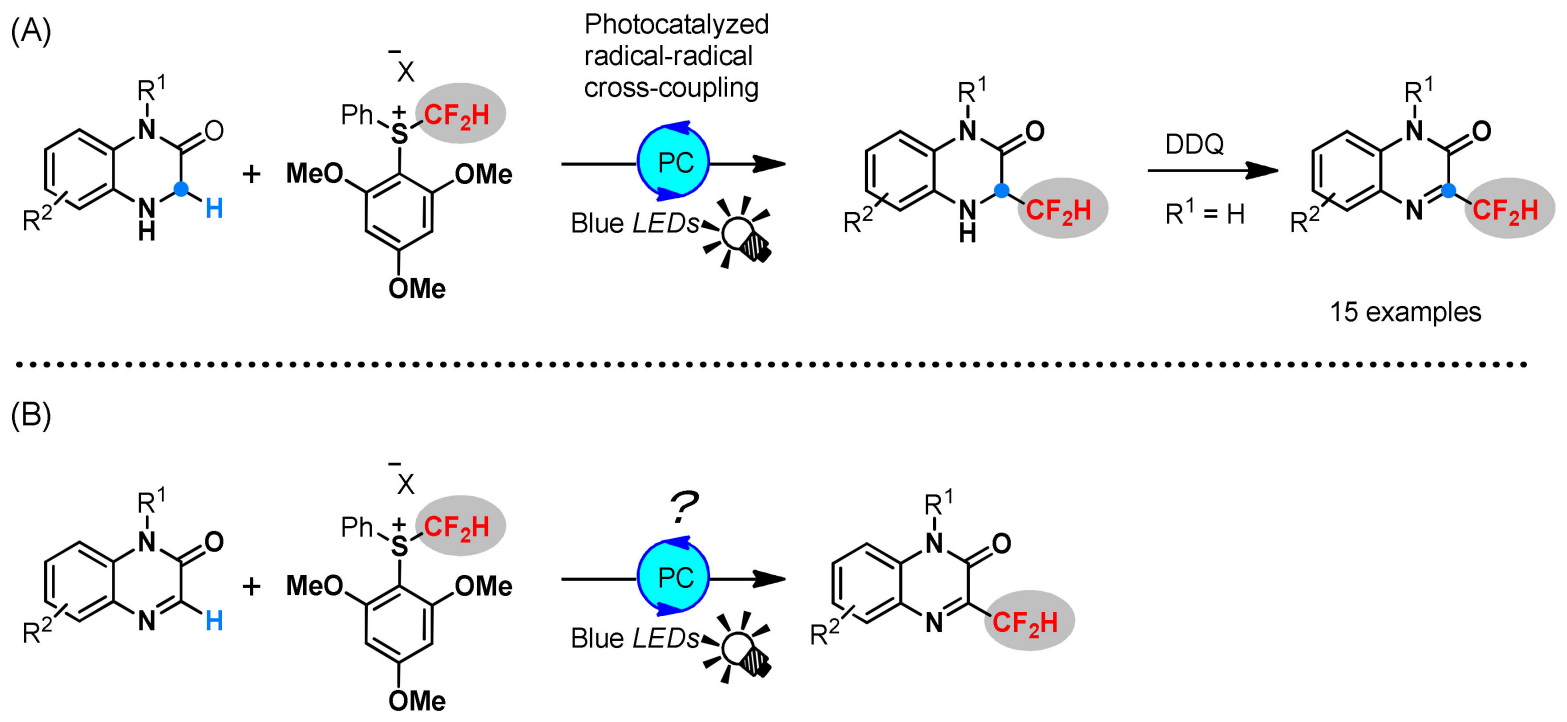
Synthesis of 3-CF_2_H-quinoxalin-2-ones. (**A**) A one-pot process for the synthesis of 3-CF_2_H- quinoxalin-2-ones, enabled by the visible-light redox-catalyzed difluoromethylation of 1, 4-dihydro-quinoxalin-2-ones, following oxidation with DDQ. (**B**)Protocol for direct difluoromethylation of quinoxalin-2-ones.

On the other hand, quinoxalin-2-one is considered to be an important and prevalent structural skeleton, frequently existing in plenty of bioactive molecules and drugs and having a wide variety of biological activities such as antiviral, anti-inflammatory, antidiabetic, antimicrobial, and anticancer properties. Usually, modification on the C-3 position of quinoxalin-2-one is a common strategy to access structurally diverse quinoxalin-2-ones and leads to compounds with an improved biological activity. Moreover, in view of the significant potential of CF_2_H in pharmaceuticals, quinoxalin-2-ones decorated with CF_2_H at the C-3 position could provide more chances to seek lead molecules and drug candidates. Consequently, the difluoromethylation of quinoxalin-2-ones gradually aroused the interest of the synthetic chemistry and medicinal chemistry community in recent years. However, it remains a greatly challenging task to introduce a CF_2_H unit into quinoxalin-2-ones, despite the fact that a few approaches with a limited substrate scope were documented over the past few years [28,29,30]. As one part of our continuous efforts in developing difluoromethylating reagents and the application of their evaluation [31,32,33,34,35,36], we reported bench-stable electrophilic difluoromethylating reagents, *S*-(difluoromethyl)solfonium salts [31], which proved to be good difluoromethyl radical (·CF_2_H) reagents and readily yielded ·CF_2_H species to deliver a variety of radical transformations under photoredox catalytic conditions [35,36]. Very recently, we presented a one-pot process for the synthesis of 3-CF_2_H- quinoxalin-2-ones, enabled by the visible-light redox-catalyzed difluoromethylation of 1, 4-dihydro-quinoxalin-2-ones, following oxidation with DDQ (Scheme 1A) [37]. Hence, we question whether direct difluoromethylation of quinoxalin-2-ones could be achieved to easily access 3-CF_2_H-quinoxalin-2-ones. Consequently, herein, we report a more practical protocol for direct difluoromethylation of quinoxalin-2-ones (Scheme 1B).

## 2. Results and Discussions

Initially, we commenced our investigation by employing 1-methyl-quinoxalin-2-one **1aa** as the model substrate and our difluoromethylating reagent **2** (2.0 equiv.) as a difluoromethyl radical source (·CF_2_H). To our delight, the reaction proceeded smoothly to afford the desired product, 3-CF_2_H-quinoxalin-2-one **3aa,** in a 53% isolated yield under blue light irradiation in the presence of 3 mol % photosensitizer PCI and 2.0 equivalents of LiOH (Table 1, entry 1). Encouraged by this result, we further examined other commercially available photocatalysts, as shown in Table 1; Perylene offered a comparable result (entry 3), and the yield was not improved when employing 4CzIPN (entry 2), whereas the common organic photosensitizers Rose Bengal and Eosin Y were less effective for this transformation (entries 4,5). Ir-complex *fac*-Ir(ppy)_3_ and Ir(dFppy)_3_ were also effective photocatalysts, albeit offering lower yields of 39% and 41% (entries 6–7), respectively. We were delighted that PCII facilitated this reaction with a satisfied yield of 60% (entry 8), which proved to be the best one. Next, the effect of various bases on this reaction was surveyed; a wide variety of inorganic bases, including LiOH, LiOAc, *^t^*BuLi, K_2_CO_3_, and Na_2_CO_3_, can process this reaction to deliver the desired product, and LiOAc provided a comparable yield (entry 9). In contrast to inorganic bases, the organic base DIPEA did not work thoroughly (entry 13), probably due to its interaction with sulfonium salt **2**. Additionally, screening of the solvent revealed that EtOAc was still the best one (entries 14–17 vs. 8), and aprotic polar solvent THF dramatically eroded this reaction, affording only a 24% yield (entry 17). Moreover, both increasing and decreasing reaction temperatures led to inferior results (entries 18, 19). Furthermore, the use of 5 mol% of PCII (entry 21) or prolonging the reaction time to 36 h (entry 20) did not further benefit the result. Predictably, both light and the photosensitizer were essential for this reaction, and no desired product was detected in the absence of each element (entries 22, 23).

**Figure 2:**
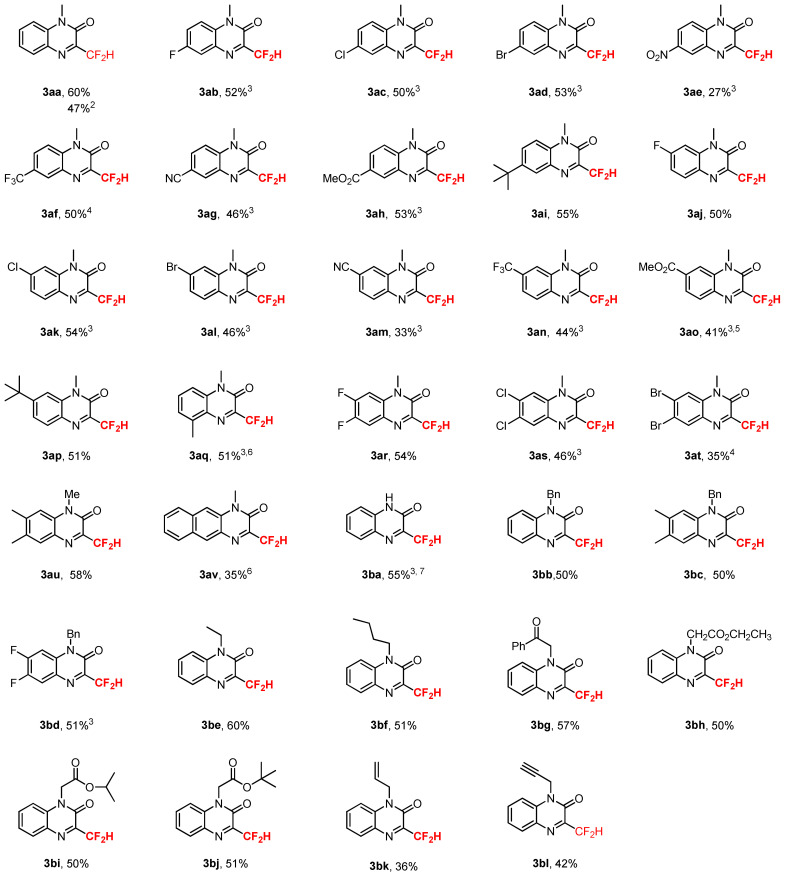
Scope of visible-light-induced difluoromethylation of quinoxalin-2-ones ^1^.^1^ Reaction conditions (unless otherwise specified): **1** (0.2 mmol, 1.0 equiv.), PC II (3 mol%), reagent **2** (0.4 mmol, 2.0 equiv.), LiOH (0.4 mmol, 2.0 equiv.), EtOAc(3.0 mL), 25 °C, overnight (18 h). Isolated yields. ^2^ large scale (5 mmol) reaction. ^3^ 3 equiv. of reagent **2** and LiOH were used. ^4^ 4 equiv. of reagent **2** and LiOH were used. ^5^ CH_3_CN was used as solvent. ^6^ PC I was used as photocatalyst. ^7^ Perylene used as photocatalyst.

To examine the generality of this method, we further explored the broad substrate’s scope to access structurally diverse 3-CF_2_H-quinoxaline-2-ones under the optimized reaction conditions or modified conditions (entry 8 in Table 1). As illustrated in Figure 2, the broad scope of substrates readily underwent this transformation to produce corresponding desired products in moderate to good yields. The tolerance of functional groups was investigated, and a wide range of functional groups, regardless of their electronic property, were compatible with this method, including F (**3ab**, **3aj**, **3ar**, **3bd**), Cl (**3ac**, **3ak, 3as**), Br (**3ad**, **3al, 3at**), NO_2_ (**3ae**), CF_3_ (**3af**, **3an**), CN (**3ag**, **3am**), CO_2_Me (**3ah**, **3ao**), *^t^*Bu (**3ai**, **3ap**), Me (**3aq**, **3au**, **3bc**), etc., providing corresponding desired products in moderate to good yields. Notably, in the case of **1ao**, CH_3_CN was used as solvent instead of EtOAc to give **3ao** in a 41% yield. Moreover, in many cases, three equivalents of reagent **2** and LiOH were used to gain better result, and four equivalents of reagent **2** and LiOH were necessary for **3af** and **3at**. Furthermore, the reaction was not sensible to a substituent position on the phenyl ring, and 5, 6, 7-substitutions were all well tolerated and exhibited a similar transformation efficiency (**3ab**-**3ai** vs. **3aj**–**3ap** and **3aq**). Remarkably, the reaction also worked well when employing di-substituted substrates, and di-F, di-Cl, di-Br and di-Me-quinoxaline-2-ones were transferred into corresponding desired products in good yields (**3ar**, **3as**, **3at**, **3au** and **3bc**, **3bd**). Benzoquinoxalin-2-one **1aq** was also shown to deliver **3aq** when photocatalyst PC I was used instead of PC II, albeit in a lower isolated yield of 35%. The influence of protection groups on N1 was also examined. As a result, other alkyl groups, such as ethyl and n-butyl, were also compatible for delivering **3be** and **3bf** in good yields of 60% and 51%, respectively. Benzyl also proved to be a compatible N1-protection group, providing **3bb**, **3bc** and **3bd** in good yields, regardless of electron-donating or electron-withdrawing group ornaments on the phenyl ring. Moreover, ketone and ester were also well-tolerant in this protocol and afforded **3bg**–**i** in good yields. Interestingly, alkene and alkyne groups remained unreactive to giving the corresponding desired products under the standard conditions, at 36% (**3bk**) and 42% yields (**3bl**), respectively. Notably, N1-unprotected quinoxalin-2-one also efficiently processed this transformation, and **1ba** readily converted to **3ba** in a 55% yield using perylene as the photosensitizer. Finally, a large-scale transformation of **1aa** (5.0 mmol) was demonstrated with a 47% isolated yield.

**Scheme 2:**
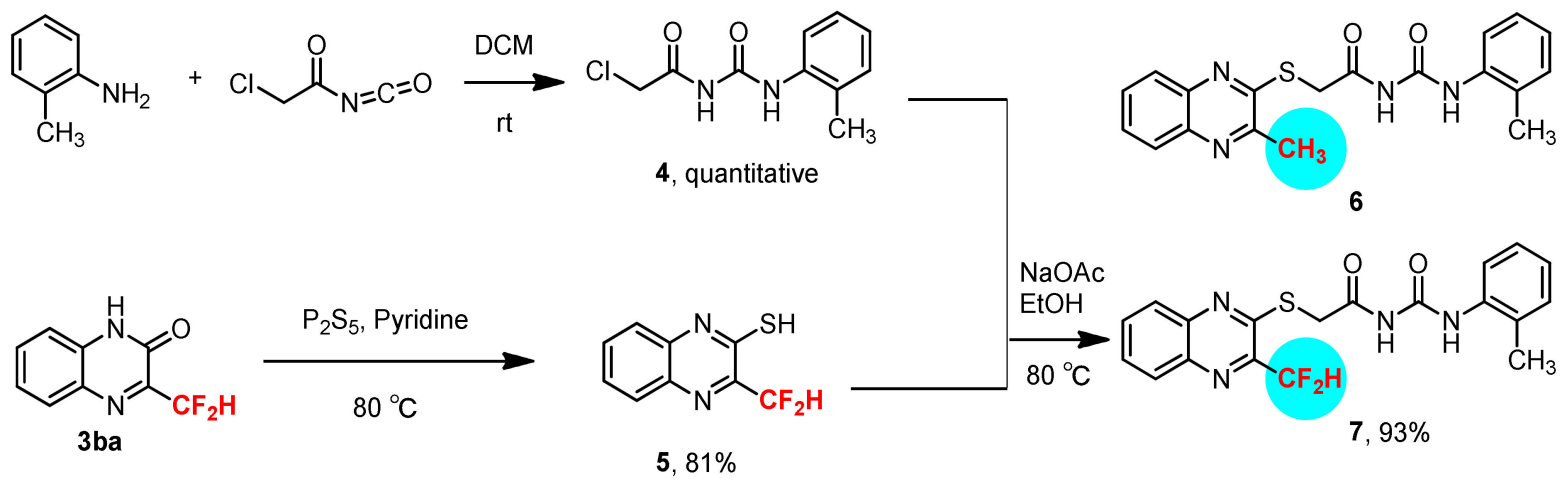
Synthesis of 3-CF_2_H-quinoxalin-2-mercapto-acetyl-urea.

3-methyl-quinoxalin-2-mercapto-acetyl-urea **6**, a vital quinoxaline-based, biologically active compound, exhibits significant potential as an inhibitor of Ebola and Marburg VP40 egress with low nM activity [38]. To further demonstrate the potential practicality of our approach, we conducted a rapid synthesis of 3-difluoromethyl-quinoxalin-2-mercapto-acetyl-urea **7** (Scheme 2), which might facilitate dramatically improved antiviral activity. Thus, difluoromethyl-functionalized compound **7** was readily accessed via three steps in a total yield of 75% from **3ba**.

**Scheme 3:**
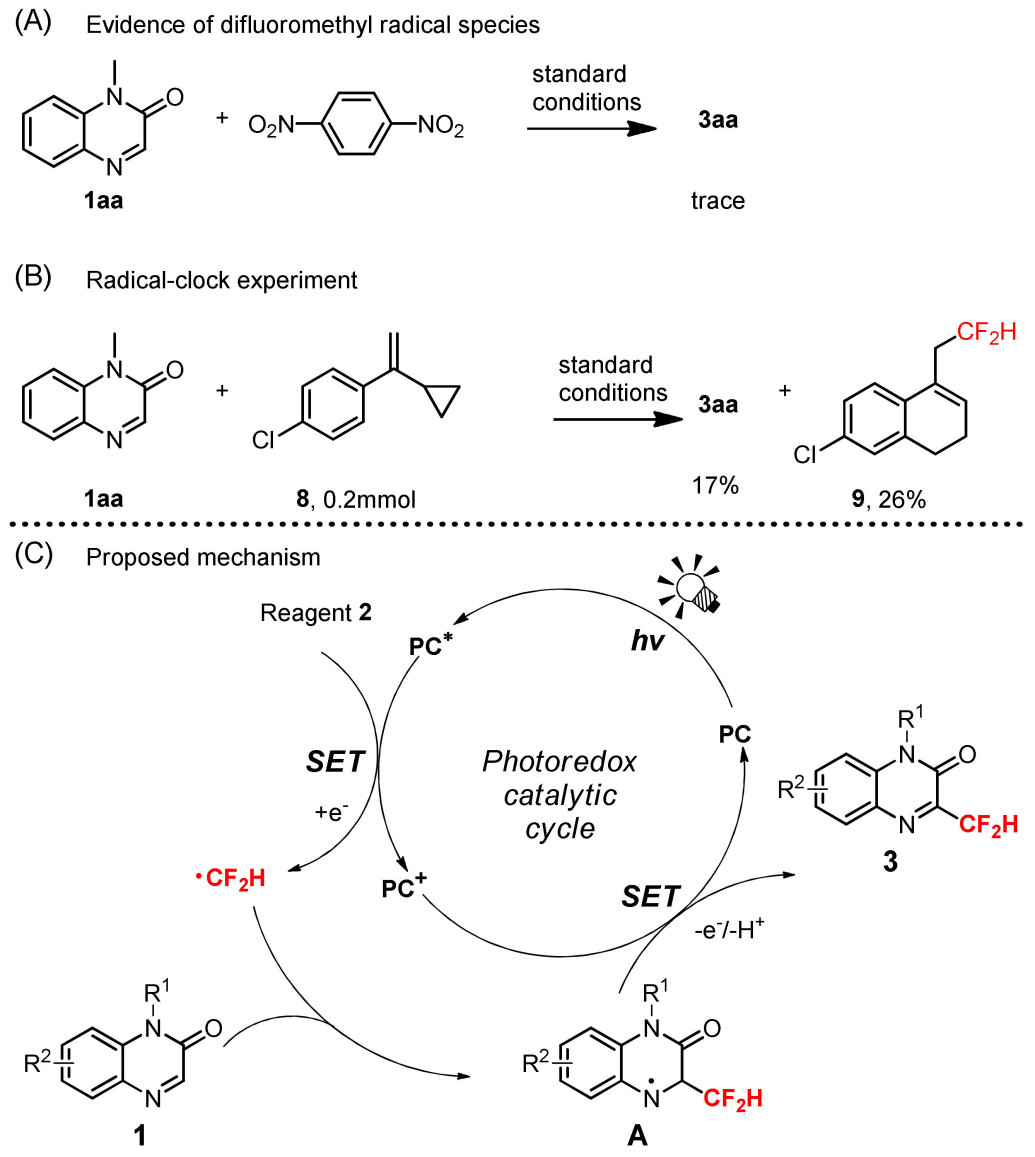
Mechanistic studies and proposed mechanism. (**A**) Evidence of difluoromethyl radical species (**B**)Radical – clock experiment (**C**) Proposed mechanism.

To reveal detailed insights into the mechanism of this transformation, several control experiments were carried out. The desired transformation was almost thoroughly depressed under the standard reaction conditions in the presence of radical inhibitor 1, 4-dinitrobenzene, and only a trace of **3aa** was detected (Scheme 3A), indicating that a radical pathway is probably involved in the reaction. Moreover, a radical clock experiment was also explored, and thus the desired product **3aa** was reduced to a 17% yield, while the rearranged product **9** from vinylcyclopropane **8** was isolated as the major product in a 26% yield (Scheme 3B). Consequently, the above experimental results clearly suggested that difluoromethyl radical species were involved in this transformation.

Based on these results from mechanistic experiments, a plausible reaction mechanism is proposed, as illustrated in Scheme 3C. Initially, difluoromethylating reagent **2** is reduced by the excited state PC* to deliver the CF_2_H radical and PC^+^; then, CF_2_H radical adds to quinoxalin-2-one **1**, leading to intermediate nitrogen radical **A**, which undergoes single-electron oxidation by PC^+^ and a deprotonation process to render the desired product **3**, along with the renewal of PC.

## 3. Materials and Methods

### 3.1. General Experimental Information

^1^H NMR spectra were recorded on either a Bruker Ascend 400 MHz spectrometer, a Bruker Ascend 500 MHz spectrometer or a Bruker Ascend 600 MHz spectrometer at ambient temperature unless otherwise indicated. Data were reported as follows: chemical shifts in ppm from tetramethylsilane as an internal standard in CDCl_3_ or DMSO-d6, integration, multiplicity (s = singlet, d = doublet, t = triplet, q = quartet, dd = doublet-doublet, m = multiplet, br = broad), coupling constants (Hz), and assignment. ^13^C NMR spectra were recorded on either a Bruker Ascend 500 MHz(126 MHz) spectrometer, a Bruker Ascend 400 MHz (101 MHz) spectrometer or a Bruker Ascend 600 MHz (151 MHz) spectrometer at ambient temperature and were proton-decoupled. Chemical shifts are reported in ppm from tetramethylsilane on a scale with the solvent resonance employed as the internal standard. ^19^F NMR spectra were recorded on a Bruker Ascend 400 MHz (377 MHz) spectrometer or a Bruker Ascend 500 MHz (471 MHz) spectrometer at ambient temperature. Chemical shifts are reported in ppm from CFCl3 as the internal standard. ESI-MS analyses were performed in positive ionization mode on an Agilent 1260-Infinity LC/MSD resolution mass spectrometer. All high-resolution mass spectra were obtained on a Thermo Scientific Q-Exactive (HR/AM) Orbitrap mass spectrometer. Commercially available reagents were used as received. Reactions were monitored by TLC (detection with UV light). Flash chromatography: silica gel (300–400 mesh). Visible light irradiation was performed by Blue LED lamps (10 W; λ = 450 nm) for a preparative scale. Regent **2** was synthesized based on a reported procedure, and analytical data were consistent with those reported in the literature [31]. The NMR and mass data of all compounds are provided in the Supplementary Materials.

### 3.2. Reaction Apparatus

**Figure 3:**
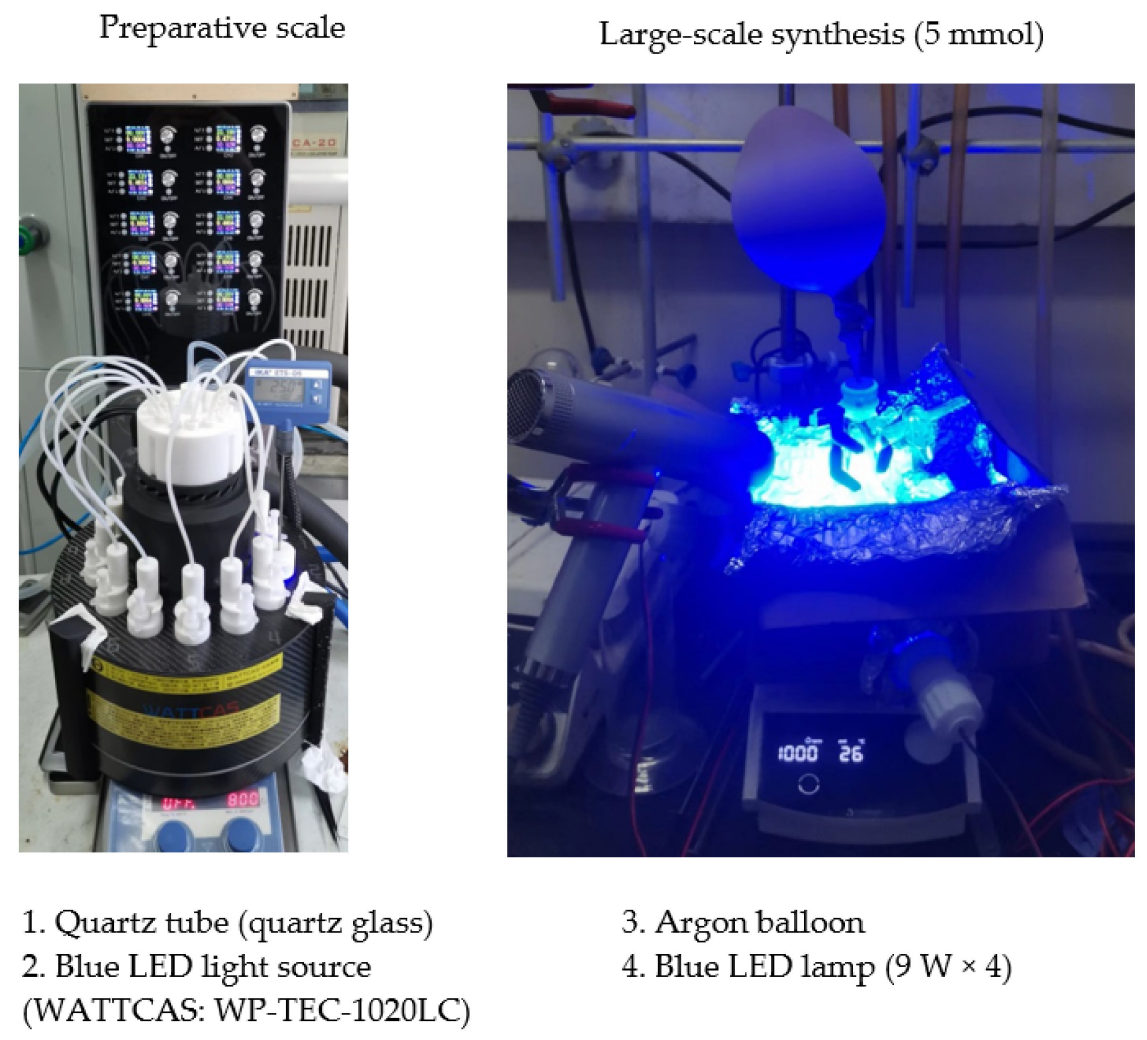
Reaction apparatus.

All reaction apparatus were illustrated in Figure 3 below.

### 3.3. Synthesis of Quinoxalin-2-Ones

All qunoxalin-2-ones are known and prepared according to these reported methods, and characteristic data are consistent with those reported in the literature. **1aa**–**1ab** and **1ba**–**1bd** [38], **1ac**–**1aq** and **1be**–**1bl** [39], **1ar**–**1av** [40].

### 3.4. General Procedure for Visible-Light Redox-Catalyzed Difluoromethylation of Quinoxalin-2 ones

**Scheme 4:**
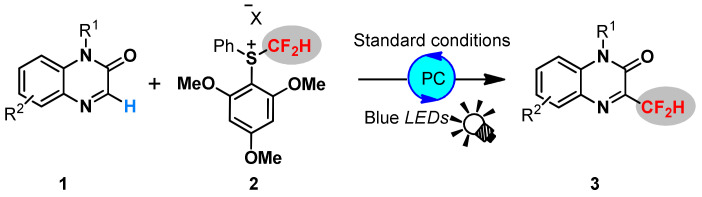
General reaction procedure.

1-methylquinoxalin-2(1H)-one **1** (0.2 mmol, 1.0 equiv.), **2** (166.0 mg, 0.4 mmol, 2.0 equiv., or 0.6 mmol, 3.0 equiv., or 0.8 mmol, 4.0 equiv.), photocatalyst (4.4.mg, 0.006 mmol, 0.03 equiv. PCII, or PCI, or Perylene), LiOH (9.6 mg, 0.4 mmol, 2.0 equiv., or 0.6 mmol, 3.0 equiv., or 0.8 mmol, 4.0 equiv.) was added to a 20 mL quartz tube (quartz glass) equipped with a magnetic stir bar. Then, the flask was flushed with argon 3 times, followed by the addition of EtOAc (3 mL). The reaction mixture was stirred and irradiated using a 10 W 450 nm LED lamp (WATTCAS: WP-TEC-1020LC) at 25 °C for 18 h. Then, the reaction mixture was concentrated in vacuo. The residue was purified by flash column chromatography on silica gel (petrol ether/dichloromethane/ethyl acetate) (Scheme 4).

### 3.5. Larger-Scale Experiment

**Scheme 5:**
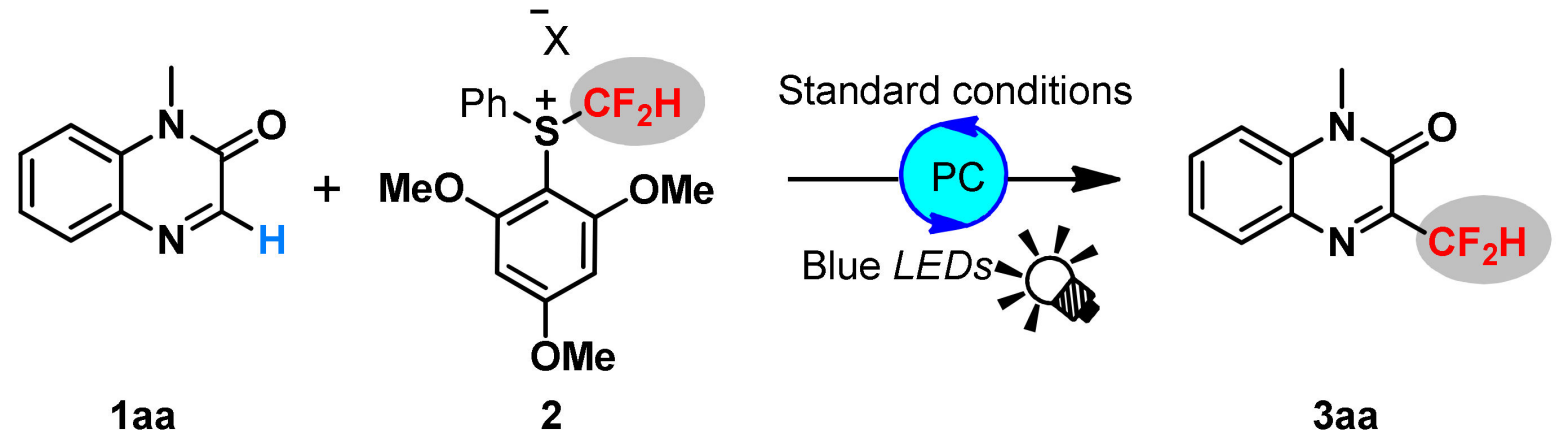
Larger-scale experiment.

A 200 mL flame-dried flask (Synthware Glass, Beijing F588100N) was charged with 1-methylquinoxalin-2(1*H*)-one **1aa** (0.80 g, 5.0 mmol, 1.0 equiv), *S*-(difluoromethyl)sulfonium salt **2** (4.14 g, 10.0 mmol, 2.0 equiv), PCII (110.6 mg, 0.15 mmol, 0.03 equiv), and LiOH (242.0 mg, 10.0 mmol, 2.0 equiv.). The flask was evacuated and backfilled with argon 3 times, and then the solvent EtOAc was added by syringe (30 mL). The tube was placed at a distance of ~1 cm away from blue LED lamps (9 W × 4, λ = 450 nm), and the resulting reaction mixture was stirred under irradiation of blue LEDs at 25 °C for 18 h. The solvent was removed under reduced pressure. The residue was purified by flash column chromatography on silica gel (petrol ether/dichloromethane/ethyl acetate = 30:10:1) to give the desired product 3-(difluoromethyl)-1-methylquinoxalin-2(1*H*)-one (**3aa**) as a yellow solid (0.49 g, 47%) (Scheme 5).

### 3.6. Synthesis of 3-Difluoroemthyl-quinoxalin-2-thiol **5**

**Scheme 6:**
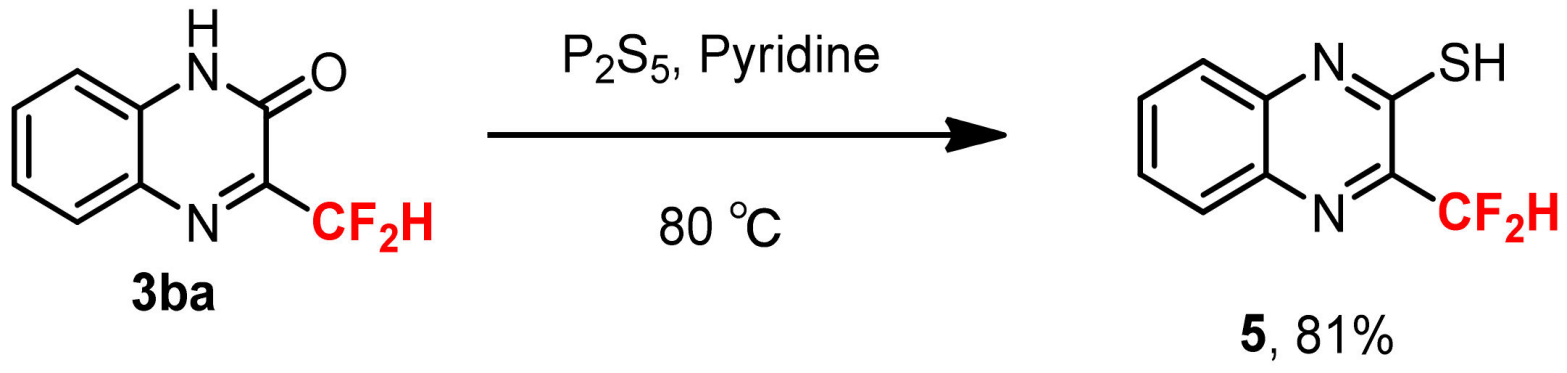
Synthesis of **5**.

Compound **5** was prepared using a modified method reported previously [41]: To a solution of difluoromethyl-1H-quinoxalin-2-one **3ba** (120 mg, 0.612 mmol) in pyridine (10 mL), we added P_2_S_5_ (412.2 mg, 1.84 mmol). The resulting mixture was vigorously stirred at 80 °C for 3 days. Then, the resulting reaction mixture was cooled to room temperature, and 50 mL water was added and extracted with EtOAc (4 × 50 mL). The combined organic layers were washed with brine (3 × 50 mL) and dried over Na_2_SO_4_. The filtration was concentrated under reduced pressure, and the crude was recrystallized from dichloromethane and petrol ether to give 105 mg (81%) **5** as a bright yellow solid (Scheme 6).

### 3.7. Synthesis of 1-(2-Chloroacetyl)-3-(2-methylphenyl)-urea **4**

**Scheme 7:**
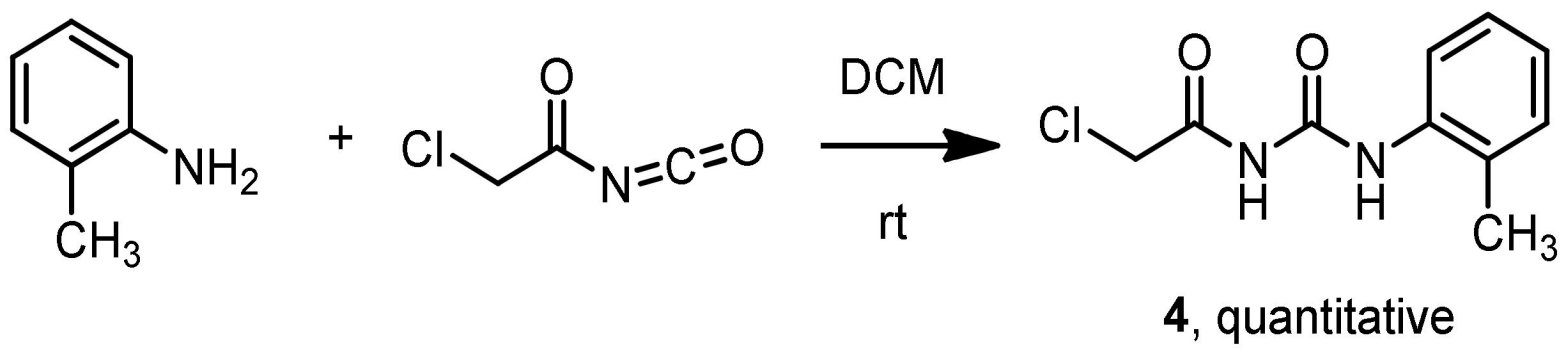
Synthesis of **4**.

Compound **4** was prepared using a modified method reported previously [41]: 2-Chloroacetylisocyanate (250 mg, 2 mmol) was added to a solution of 2-methylaniline (107 mg, 1 mmol) in DCM (5 mL) at room temperature. The resulting reaction mixture was stirred overnight and then concentrated in vacuo, and the residue was recrystallized from dichloromethane and petrol ether to give 287 mg **4** (quantitative yield) as a brown solid (Scheme 7).

### 3.8. Synthesis of 1-(2-Methylphenyl)-3-[2-(3-difluoromethyl-quinoxalin-2-ylsulfanyl)-acetyl]-urea **7**

**Scheme 8:**
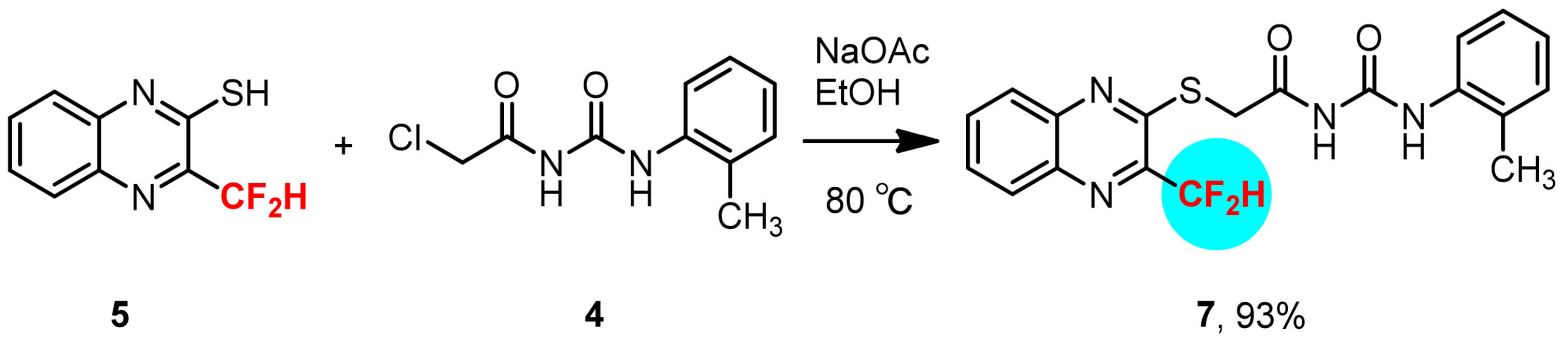
Synthesis of **7**.

Compound **7** was prepared using a modified method reported previously [41]: 3-Difluoromethyl-quinoxaline-2-thiol **5** (50 mg, 0.24 mmol), 1-(2-chloroacetyl)-3-(2-methylphenyl)-urea **4** (54.4 mg, 0.24 mmol), and sodium acetate (29.8 mg, 0.36 mol) were added to 5 mL EtOH, and the resulting mixture was heated to 80 °C and reacted overnight. The solvent was removed under reduced pressure, and the crude was recrystallized from CH_2_Cl_2_ and petrol ether, affording 90 mg **7** (93% yield) as an off-white solid (Scheme 8).

### 3.9. Radical Trapping Experiment

**Scheme 9:**
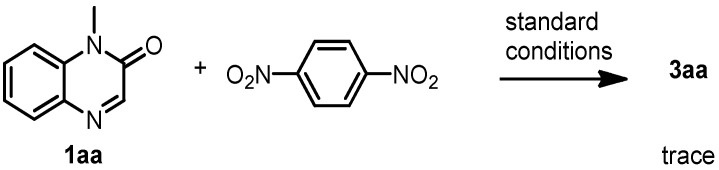
Radical trapping experiment.

The reaction was conducted under standard reaction conditions (see 3.4 above) with the addition of 1,4-dinitrobenzene (67.2 mg, 0.4 mmol, 2.0 equiv.). Additionally, the reaction mixture was monitored by **^19^F NMR** using PhF (0.2 mmol) as the internal standard, and only a trace of **3aa** was detected (Scheme 9).

### 3.10. Radical-Clock Experiment

**Scheme 10:**
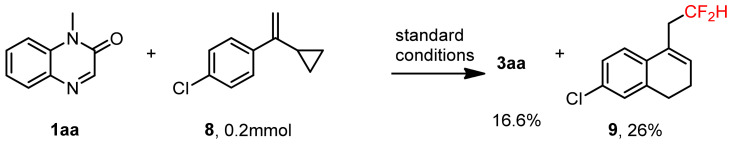
Radical-clock experiment.

The reaction was conducted under the standard reaction conditions (see 3.4 above) with the addition of 1-chloro-4-(1-cyclopropylvinyl)benzene **8** (36.0 mg, 0.2 mmol, 1.0 equiv.). The residue was purified by flash column chromatography on silica gel with petrol ether (**9**) and petrol ether/dichloromethane/ethyl acetate (**3aa**) to afford 11.8 mg **9** (26%) as a colorless oil and 7.0 mg **3aa** (17%), respectively (Scheme 10).

## 4. Conclusions

In conclusion, we have developed a facile approach for the direct difluoromethylation of quinoxalin-2-ones using electrophilic difluoromethylating reagent 2 as a difluoromethyl source. In the presence of 3 mol % photocatalyst, the reaction readily proceeded under visible light irradiation at room temperature, displaying a broad substrate scope and functional group tolerance and enabling access to a wide variety of 3-CF_2_H-quinoxalin-2-ones in moderate-to-good yields. Notably, these described products are of great interest to the pharmaceutical chemistry community and show great potential for the development of fluorine-containing drugs. Remarkably, a rapid synthesis of difluoromethylated derivative **7** from a pharmacologically antiviral agent was readily achieved. Furthermore, the reaction mechanism was also studied, and a rational pathway involving difluoromethyl radical species was proposed.

## Figures and Tables

**Table 1:** Optimization of conditions ^1^.

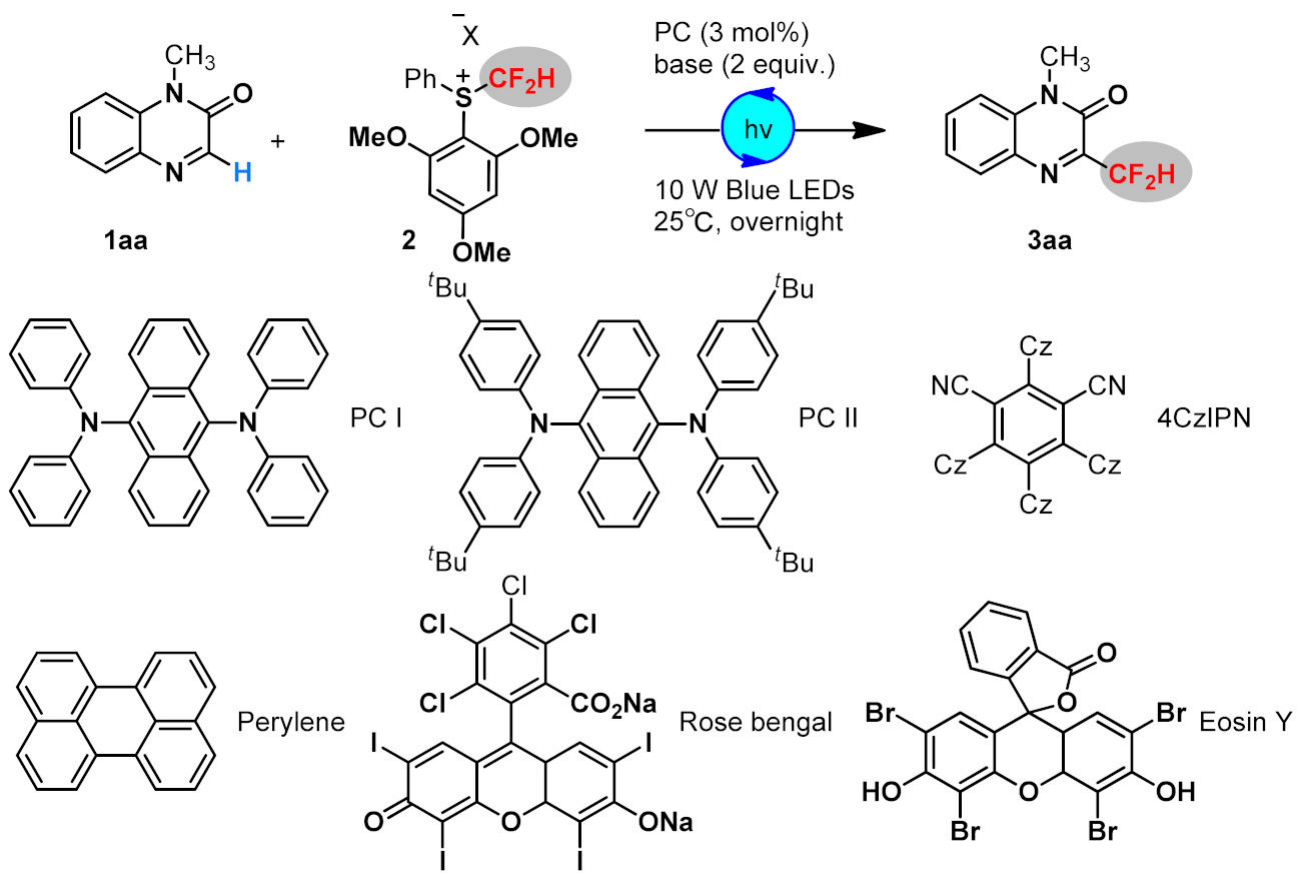					
Entry	PC	Base	Temp. (℃)	Solvent	Yield (%)
1	PC I	LiOH	25 °C	AcOEt	53
2	4CzIPN	LiOH	25 °C	AcOEt	35
3	Perylene	LiOH	25 °C	AcOEt	52
4	Rose Bengal	LiOH	25 °C	AcOEt	NR
5	Eosin Y	LiOH	25 °C	AcOEt	trace
6	*fac*-Ir(ppy)_3_	LiOH	25 °C	AcOEt	39
7	Ir(dFppy)_3_	LiOH	25 °C	AcOEt	41
8	PC II	LiOH	25 °C	AcOEt	60
9	PC II	LiOAc	25 °C	AcOEt	58
10	PC II	*^t^*BuOLi	25 °C	AcOEt	46
11	PC II	K_2_CO_3_	25 °C	AcOEt	36
12	PC II	Na_2_CO_3_	25 °C	AcOEt	24
13	PC II	DIPEA	25 °C	AcOEt	NR
14	PC II	LiOH	25 °C	DCM	56
15	PC II	LiOH	25 °C	CHCl_3_	45
16	PC II	LiOH	25 °C	CH_3_CN	45
17	PC II	LiOH	25 °C	THF	24
18	PC II	LiOH	40 °C	AcOEt	51
19	PC II	LiOH	0 °C	AcOEt	43
20 ^2^	PC II	LiOH	25 °C	AcOEt	60
21 ^3^	PC II (5 mol%)	LiOH	25 °C	AcOEt	57
22 ^4^	PC II/no light	LiOH	25 °C	AcOEt	NR
23 ^5^	no PC II	LiOH	25 °C	AcOEt	NR

^1^ Reaction conditions (unless otherwise specified): **1aa** (0.2 mmol, 1.0 equiv.), photocatalyst (3 mol%), reagent **2** (0.4 mmol, 2.0 equiv.), base (0.4 mmol, 2.0 equiv.), solvent (3.0 mL), 25 °C, overnight (18 h). Isolated yields. ^2^ Reaction time was prolonged to 36 h. ^3^ 5 mol% photocatalyst was used. ^4^ No blue light irradiation. ^5^ No photocatalyst.

## Data Availability

Data is contained within the article and the Supplementary Materials.

## Supplementary Materials

The following are available online at https://www.mdpi.com/article/10.3390/ph15121552/s1; Supplementary Materials is the NMR and mass data of compounds (Figure S1–S74).
