# Supplementary material for: Facile Entry to Pharmaceutically Important 3-Difluoromethyl-Quinoxalin-2-Ones Enabled by Visible-Light-Driven Difluoromethylation of Quinoxalin-2-Ones
Source: Pharmaceuticals (Basel). 2022 Dec 13;15(12):1552. doi: 10.3390/ph15121552 (PMC9781376; doi:10.3390/ph15121552)

Supplementary Materials

Facile entry to pharmaceutically important 3-difluoromethyl-quinoxalin-2-ones enabled by visible-light driven difluoro-methylation of quinoxalin-2-ones

Table of Contents

Contents		Page
**1.**	**General Experimental Information**	S2
**2.**	**Reaction Apparatus**	S3
**3.**	**Synthesis of Quinoxalin-2-ones 1**	S3
**4.**	**General Procedure for Visible-light Redox Catalyzed Difluoromethylation of Quinoxalin-2ones 3**	S3
**5.**	**Lager Scale Experiment**	S4
**6.**	**Synthetic Application**	S4
**7.**	**Mechanistic Experiments**	S6
**8.**	**Characterization Data for the Difluoromethylated quinoxalin-2(1*H*)-ones**	S7
**9.**	**References**	S18
**10.**	**NMR Spectra for New Compounds**	S19

1. General Experimental Information

^{1}H NMR spectra were recorded on either a Bruker Ascend 400 MHz spectrometer, a Bruker Ascend 500 MHz spectrometer or a Bruker Ascend 600 MHz spectrometer at ambient temperature unless otherwise indicated. Data were reported as follows: chemical shifts in ppm from tetramethylsilane as an internal standard in $CDCl_3$ or DMSO-d_6, integration, multiplicity (s = singlet, d = doublet, t = triplet, q = quartet, dd = doublet-doublet, m = multiplet, br = broad), coupling constants (Hz), and assignment. ^{13}C NMR spectra were recorded on either a Bruker Ascend 500 MHz(126 MHz)spectrometer, a Bruker Ascend 400 MHz(101 MHz) spectrometer or a Bruker Ascend 600 MHz (151 MHz) spectrometer at ambient temperature and were proton decoupled. Chemical shifts are reported in ppm from tetramethylsilane on the scale with the solvent resonance employed as the internal standard. ^{19}F NMR spectra were recorded on a Bruker Ascend 400 MHz (377 MHz) spectrometer or a Bruker Ascend 500 MHz (471 MHz) spectrometer at ambient temperature. Chemical shifts are reported in ppm from $CFCl_3$ as the internal standard. ESI-MS analysis were performed in positive ionization mode on an Agilent 1260-Infinity LC/MSD resolution mass spectrometer. All high-resolution mass spectra were obtained on a Thermo Scientific Q-Exactive (HR/AM) Orbitrap mass spectrometer. Commercially availa-

ble reagents were used as received. Reactions were monitored by TLC (detection with UV light). Flash chromatography: silica gel (300-400 mesh). Visible light irradiation was performed by Blue LED lamps (10 W; λ = 450 nm) for preparative scale. Regent **2** was synthesized based on reported procedure and analytical data are in consistent with those reported in the literature [1].

2. Reaction Apparatus

Preparative scale

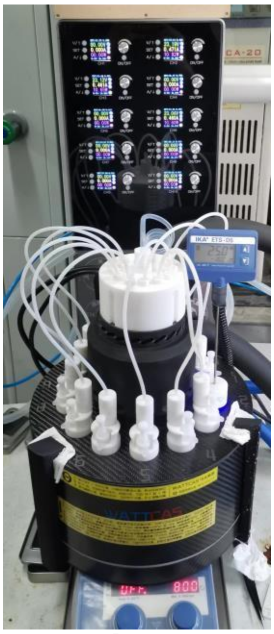

Gram-scale synthesis (5 mmol)

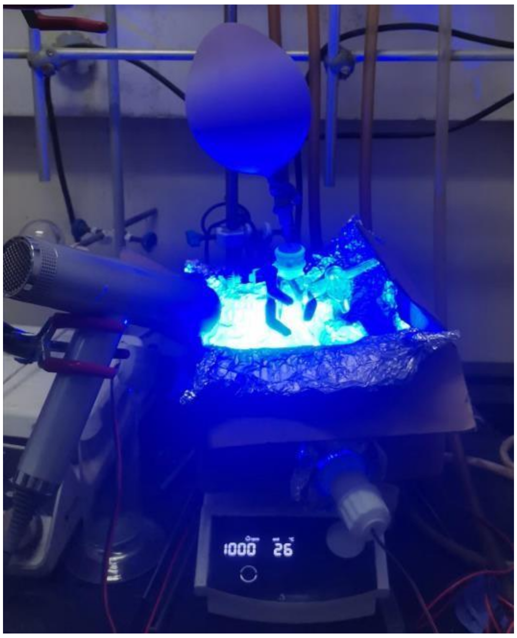

1. Quartz tube (quartz glass)
2. Blue LED light source (WATTCAS: WP-TEC-1020LC)
3. Argon balloon
4. Blue LED lamp (9 W × 4)

3. Synthesis of Quinoxalin-2-ones 1.

All All qunoxalin-2-ones are known and prepared according to these reported methods, and characteristic data are in consistent with those reported in literatures. **1aa-1ab** [2], **1ba-1bd** [2], **1be-1bl** [3], **1ac-1aq** [3], **1ar-1av** [4].

4. General Procedure for Visible-light Redox Catalyzed Difluoromethylation of Quinoxalin-2ones 3

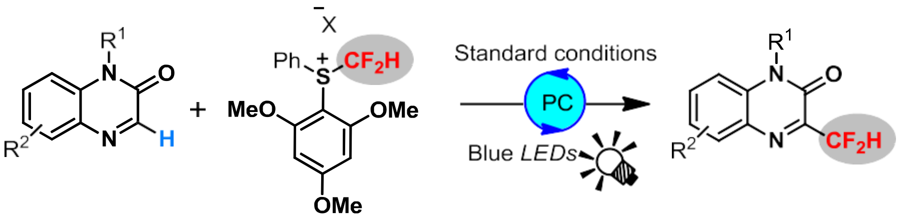

1 **2** **3**

To a 20 mL Quartz tube (quartz glass) equipped with a magnetic stir bar was added 1-methylquinoxalin-2(1H)-one **1** (0.2 mmol, 1.0 equiv.), **2** (166.0 mg, 0.4 mmol, 2.0 equiv. or 0.6 mmol, 3.0 equiv., or 0.8 mmol, 4.0 equiv.), photocatalyst (4.4.mg, 0.006 mmol, 0.03 equiv. PCⅡ,

or PC Ⅰ, or Perylene), LiOH (9.6 mg, 0.4 mmol, 2.0 equiv. or 0.6 mmol, 3.0 equiv., or 0.8 mmol, 4.0 equiv.). Then flask was flushed with argon 3 times, followed by the addition of EtOAc (3 mL).

The reaction mixture was stirred and irradiated using a 10 W 450 nm LED lamp (WATTCAS: WP-TEC-1020LC) at 25 °C for 18 hours. Then the reaction mixture was concentrated in vacuo. The residue was purified by flash column chromatography on silica gel (petrol ether/dichloromethane/ethyl acetate).

5. Lager Scale Experiment

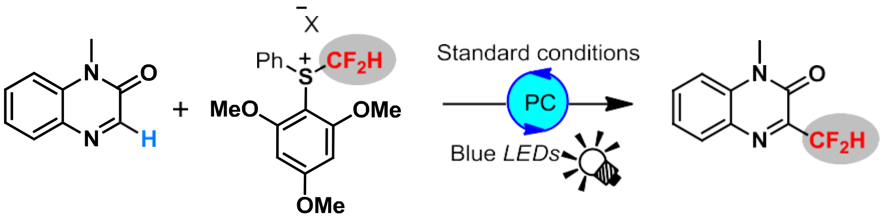

1aa **2** **3aa**

A 200 mL flame-dried flask (Synthware Glass, Beijing F588100N) was charged with 1-methylquinoxalin-2(1*H*)-one **1aa** (0.80 g, 5.0 mmol, 1.0 equiv), *S*-(difluoromethyl)sulfonium salt **2** (4.14 g, 10.0 mmol, 2.0 equiv), PC Ⅱ (110.6 mg, 0.15 mmol, 0.03 equiv), and LiOH (242.0 mg, 10.0 mmol, 2.0 equiv.). The flask was evacuated and backfilled with argon 3 times, then the solvent EtOAc was added by syringe (30 mL). The tube was placed at a distance of ~1 cm away from blue LED lamps (9 W × 4, λ = 450 nm), the resulting reaction mixture was stirred under irradiation of blue LEDs at 25 °C for 18 hours. The solvent was removed under reduced pressure. The residue was purified by flash column chromatography on silica gel (petrol ether/dichloromethane/ethyl acetate = 30:10:1) to give the desired product 3-(difluoromethyl)-1-methylquinoxalin-2(1*H*)-one (**3aa**) as a yellow solid (0.49g, 47%).

6. Synthetic Application

Synthesis of 3-Difluoroemthyl-Quinoxalin-2-Thiol 5

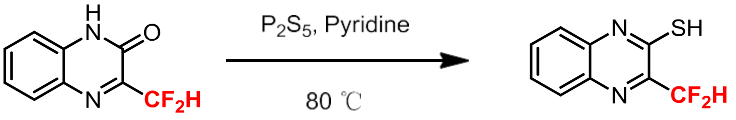

3ba **5**, 81%

Compound **5** was prepared using a modified method reported previously [5]: To a solution of 3-(difluoromethyl)quinoxalin-2(1*H*)-one **3ba** (120 mg, 0.612 mmol) in pyridine (10 mL) added P_2S_5 (412.2 mg, 1.84 mmol). The resulting mixture was vigorously stirred at 80 °C for 3 days. Then the resulting reaction mixture was cooled to room temperature, 50 mL water was added, extracted with EtOAc (4 × 50 mL). The combined organic layers were was washed with brine (3 × 50 mL) and dried with over Na_2SO_4. The filtration was concentrated under reduced pressure, and the crude was recrystallized from dichloromethane and petrol ether to give 105mg (81%) **5** as a bright yellow solid.

Mp: 218.2-219.4 °C. ^{1}H NMR (400 MHz, DMSO-d_6) δ 14.77 (s, 1H), 8.00 – 7.95 (m, 1H), 7.82 – 7.73 (m, 1H), 7.61 (d, *J* = 8.3 Hz, 1H), 7.54 (t, *J* = 52.0 Hz, 1H), 7.58 – 7.50 (m, 1H). ^{13}C NMR (151 MHz, DMSO-d_6) δ 172.8, 152.2 (t, *J* = 20.9 Hz), 134.8, 133.6, 133.4, 130.0, 127.0, 116.7, 110.1 (t, *J* = 240.0 Hz).

^{19}F NMR (377 MHz, DMSO-d_6) δ -123.0 (d, J = 53.7 Hz, 2F). HRMS (ESI): m/z $[M+H]^+$ calcd for $C_9H_7F_2N_2S^+$ 213.0298, found 213.0302.

Synthesis of 1-(2-Chloroacetyl)-3-(2-Methylphenyl)-urea 4

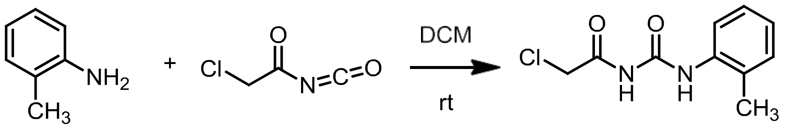

4, quantitative

Compound **4** was prepared using a modified method reported previously [5]: 2-Chloroacetylisocyanate (250 mg, 2 mmol) was added to a solution of 2-methylaniline (107 mg, 1 mmol) in DCM (5 mL) at room temperature. The resulting reaction mixture was stirred overnight then concentrated *in vacuo*, and the residue was recrystallized from dichloromethane and petrol ether to give 287 mg **4** [5] (quantitative yield) as a brown solid, and analytical data are in agreement with those reported in literature[5].

Synthesis of 1-(2-Methylphenyl)-3-[2-(3-Difluoromethyl-Quinoxalin-2-Ylsulfanyl) -Acetyl]-Urea 7

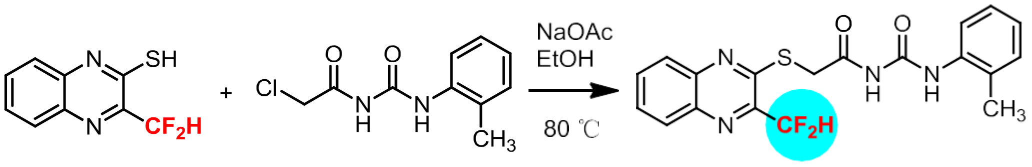

5 **4** **7**, 93%

Compound **7** was prepared using a modified method reported previously [5]: 3-Difluoromethyl-quinoxaline-2-thiol **5** (50 mg, 0.24 mmol), 1-(2-chloroacetyl)-3-(2-methylphenyl)-urea **4** (54.4 mg, 0.24 mmol), and sodium acetate (29.8 mg, 0.36 mol) were added to 5mL EtOH, and the resulting mixture was heated to 80 °C and reacted overnight. The solvent was removed under reduced pressure, and the crude was recrystallized from CH_2Cl_2 and petrol ether, affording 90 mg **7** (93% yield) as an off-white solid. Mp: 179.7-181.2 °C. ^1H NMR (600 MHz, DMSO-d_6) δ 11.28 (s, 1H), 10.29 – 10.26 (m, 1H), 8.14 (d, J = 8.3 Hz, 1H), 7.94 (td, J = 8.9, 5.2 Hz, 3H), 7.84 (ddd, J = 8.6, 6.3, 2.0 Hz, 1H), 7.28 (t, J = 53.5 Hz, 1H), 7.20 (dt, J = 7.1, 4.1 Hz, 2H), 7.02 (t, J = 7.4 Hz, 1H), 4.39 (s, 2H), 2.14 (s, 3H). ^{13}C NMR (151 MHz, DMSO-d_6) δ 171.5, 152.8, 151.2, 144.3 (t, J = 25.6 Hz), 142.1, 137.9, 136.3, 133.2, 130.7, 130.4, 129.8, 128.1, 127.5, 126.9, 124.4, 121.4, 113.9 (t, J = 240.7 Hz), 34.9, 17.9. ^{19}F NMR (471 MHz, DMSO-d_6) δ -118.6 (d, J = 53.2 Hz, 2F). HRMS (ESI): m/z $[M+Na]^+$ calcd for $C_{19}H_{16}F_2N_4O_2SNa^+$ 425.0860, found 425.0863.

7. Mechanistic Experiments.

Evidence of difluoromethyl radical species

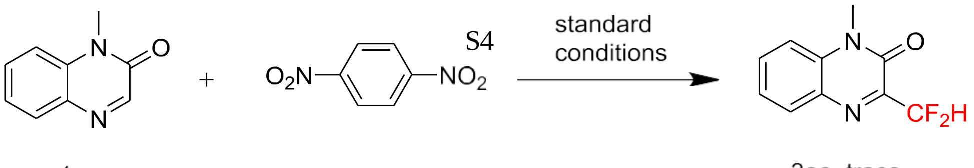

1aa 3aa, trace

The reaction was conducted under the standard reaction conditions (to see 4 above) with addition of 1,4-dinitrobenzene (67.2 mg, 0.4 mmol, 2.0 equiv.). And the reaction mixture was monitored by **^{19}F NMR** using PhF (0.2 mmol) as the internal standard, and only trace of **3aa** was detected.

Radical-clock experiment

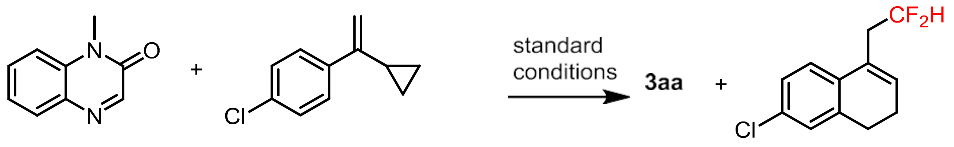

The reaction was conducted under the standard reaction conditions (to see 4 above) with addition of 1-chloro-4-(1-cyclopropylvinyl)benzene **8** (36.0 mg, 0.2 mmol, 1.0 equiv.), The residue was purified by flash column chromatography on silica gel with petrol ether (**9**) and petrol ether/dichloromethane/ethyl acetate (**3aa**) to afford 11.8 mg **9** (26%) as a colorless oil and 7.0 mg **3aa** (17%), respectively. ^{1}H NMR (500 MHz, $CDCl_3$) δ 7.23 – 7.08 (m, 3H), 6.04 (t, *J* = 4.7 Hz, 1H), 5.90 (tt, *J* = 56.7, 4.7 Hz, 1H), 3.03 – 2.88 (m, 2H), 2.74 (t, *J* = 8.1 Hz, 2H), 2.29 (td, *J* = 7.9, 4.5 Hz, 2H). ^{13}C NMR (151 MHz, $CDCl_3$) δ 138.4, 132.6, 132.5, 130.3, 127.9, 127.9, 126.5, 123.7, 116.1 (t, *J* = 241.2 Hz), 37.7 (t, *J* = 22.4 Hz), 27.9, 22.9. ^{19}F NMR (471 MHz, $CDCl_3$) δ -113.89 (dt, *J* = 57.1, 16.5 Hz, 2F). MS (ESI): *m/z* 229.02 ($M+H^+$)

8. Characterization Data for the Difluoromethylated quinoxalin-2(1*H*)-ones

3-(difluoromethyl)-1-methylquinoxalin-2(1*H*)-one (3aa)

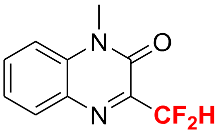

Following the general procedure for the preparation of products **3**, **3aa** was purified by silica gel chromatography (gradient eluent: PE/DCM/EtOAc = from 90/10/1 to 30/10/1) as a yellow solid (25.2 mg, 60%). ^{1}H NMR (500 MHz, $CDCl_3$) δ 7.99 (dd, *J* = 8.0, 1.5 Hz, 1H), 7.75 – 7.66 (m, 1H), 7.50 – 7.36 (m, 2H), 6.96 (t, *J* = 53.7 Hz, 1H), 3.74 (s, 3H). ^{13}C NMR (126 MHz, $CDCl_3$) δ 153.2, 148.6 (t, *J* = 22.4 Hz), 134.0, 132.7, 131.9, 131.4, 124.4, 114.0, 110.1 (t, *J* = 241.6 Hz), 29.0. ^{19}F NMR (471 MHz, $CDCl_3$) δ -124.36 (d, *J* = 53.7 Hz, 2F). MS (ESI): *m/z* 211.00 ($M+H^+$).

6-fluoro-3-(difluoromethyl)-1-methylquinoxalin-2(1*H*)-one (3ab)

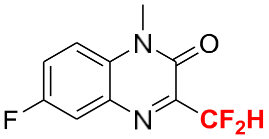

Following the general procedure for the preparation of products **3** (**2**: 0.6 mmol, 3.0 equiv., LiOH: 0.6 mmol, 3.0 equiv.), **3ab** was purified by silica gel chromatography (gradient eluent: PE/DCM/EtOAc = from 90/10/1 to 60/10/1) as a white solid (23.5 mg, 52%). ^{1}H NMR (500 MHz, $CDCl_3$) δ 7.71 (dd, *J* = 8.3, 2.9 Hz, 1H), 7.45 (ddd, *J* = 9.3, 7.6, 2.9 Hz, 1H), 7.36 (dd, *J* = 9.2, 4.6 Hz, 1H), 6.95 (t, *J* = 53.6 Hz, 1H), 3.74 (s, 3H). ^{13}C NMR (126 MHz, $CDCl_3$) δ 158.9 (d, *J* = 245.8 Hz), 152.9, 150.1 (t, *J* = 22.5 Hz), 132.4 (d, *J* = 11.3 Hz), 130.8, 120.7 (d, *J* = 24.1 Hz),

116.7 (d, *J* = 22.7 Hz), 115.2 (d, *J* = 8.7 Hz), 109.9 (t, *J* = 242.2 Hz), 29.3. ^{19}F NMR (471 MHz, $CDCl_3$) δ -117.3 (dt, *J* = 8.1, 4.0 Hz, 1F), -124.7 (d, *J* = 53.7 Hz, 2F). MS (ESI): *m/z* 250.90 ($M+Na^+$).

6-chloro-3-(difluoromethyl)-1-methylquinoxalin-2(1*H*)-one (3ac)

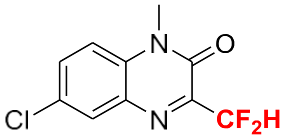

Following the general procedure for the preparation of products **3** (**2**: 0.6 mmol, 3.0 equiv., LiOH: 0.6 mmol, 3.0 equiv.), **3ac** was purified by silica gel chromatography (gradient eluent: PE/DCM/EtOAc = from 90/10/1 to 60/10/1) as a yellow solid (24.3 mg, 50%). ^{1}H NMR (500 MHz, $CDCl_3$) δ 7.99 (d, *J* = 2.5 Hz, 1H), 7.64 (dd, *J* = 9.0, 2.5 Hz, 1H), 7.33 (d, *J* = 8.9 Hz, 1H), 6.93 (t, *J* = 53.6 Hz, 1H), 3.73 (s, 3H). ^{13}C NMR (126 MHz, $CDCl_3$) δ 152.83, 149.91 (t, *J* = 22.7 Hz), 132.7, 132.7, 132.32, 130.6, 129.9, 115.1, 109.9 (t, *J* = 242.2 Hz), 29.2. ^{19}F NMR (471 MHz, $CDCl_3$) δ -124.6 (d, *J* = 53.6 Hz, 2F). MS (ESI): *m/z* 310.77 ($M+Na^+$).

6-bromo-3-(difluoromethyl)-1-methylquinoxalin-2(1*H*)-one (3ad)

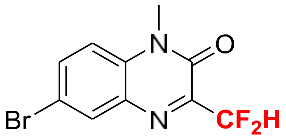

Following the general procedure for the preparation of products **3** (**2**: 0.6 mmol, 3.0 equiv., LiOH: 0.6 mmol, 3.0 equiv.), **3ad** was purified by silica gel chromatography (gradient eluent: PE/DCM/EtOAc = from 90/10/1 to 60/10/1) as a yellow solid (30.7 mg, 53%). ^{1}H NMR (500 MHz, $CDCl_3$) δ 8.15 (d, *J* = 2.3 Hz, 1H), 7.77 (dd, *J* = 8.9, 2.3 Hz, 1H), 7.31 – 7.22 (m, 1H), 6.93 (t, *J* = 53.6 Hz, 1H), 3.72 (s, 3H). ^{13}C NMR (126 MHz, $CDCl_3$) δ 152.8, 149.8 (t, *J* = 22.7 Hz), 135.4, 133.7, 133.2, 132.6, 117.0, 115.4, 109.9 (t, *J* = 242.3 Hz), 29.2. ^{19}F NMR (471 MHz, $CDCl_3$) δ -124.6 (d, *J* = 53.5 Hz, 2F). MS (ESI): *m/z* 266.87 ($M+Na^+$).

6-nitro-3-(difluoromethyl)-1-methylquinoxalin-2(1*H*)-one (3ae)

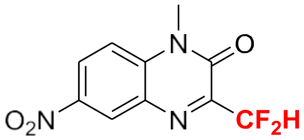

Following the general procedure for the preparation of products **3** (**2**: 0.6 mmol, 3.0 equiv., LiOH: 0.6 mmol, 3.0 equiv.), **3ae** was purified by silica gel chromatography (gradient eluent: PE/DCM/EtOAc = 40/20/1) as a yellow solid (13.6 mg, 27%), Mp: 159.6-160.8 °C. ^{1}H NMR (500 MHz, $CDCl_3$) δ 8.88 (d, *J* = 2.5 Hz, 1H), 8.53 (dd, *J* = 9.3, 2.6 Hz, 1H), 7.52 (d, *J* = 9.2 Hz, 1H), 6.92 (t, *J* = 53.3 Hz, 1H), 3.80 (s, 3H). ^{13}C NMR (126 MHz, $CDCl_3$) δ 152.7, 151.2 (t, *J* = 23.2 Hz), 143.8, 138.4, 130.9, 127.1, 126.9, 114.8, 109.8 (t, *J* = 243.1 Hz), 29.6. ^{19}F NMR (471 MHz, $CDCl_3$) δ -124.8 (d, *J* = 53.0 Hz, 2F). HRMS (ESI): *m/z* $[M+H]^+$ calcd for $C_{10}H_8F_2N_3O_3^+$ 256.0534, found 256.0531.

6-trifluoromethyl-3-(difluoromethyl)-1-methylquinoxalin-2(1*H*)-one (3af)

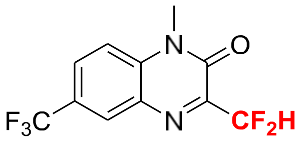

Following the general procedure for the preparation of products **3** (**2**: 0.8 mmol, 4.0 equiv., LiOH: 0.8 mmol, 4.0 equiv.), **3af** was purified by silica gel chromatography (gradient eluent: PE/DCM/EtOAc = from 90/10/1 to 60/10/1) as a yellow solid (27.9 mg, 50%), Mp: 99.5-101.9 °C. ^{1}H NMR (500 MHz, $CDCl_3$) δ 8.29 (d, *J* = 2.2 Hz, 1H), 7.91 (dd, *J* = 8.8, 2.3 Hz, 1H), 7.50 (d, *J* = 8.8 Hz, 1H), 6.94 (t, *J* = 53.5 Hz, 1H), 3.77 (s, 3H). ^{13}C NMR (126 MHz, $CDCl_3$) δ 152.9, 150.3 (t, *J* = 22.9 Hz), 136.3, 131.2, 128.9 (p, *J* = 3.9 Hz), 126.8 (d, *J* = 34.0 Hz), 124.5, 122.3, 114.8, 109.9 (t, *J* = 242.5 Hz), 29.3. ^{19}F NMR (471 MHz, $CDCl_3$) δ -62.3(s, 3F), -124.7 (d, *J* = 53.6 Hz, 2F). MS (ESI): *m/z* 300.86 (M+Na$^+$); HRMS (ESI): *m/z* [M+Na]$^+$ calcd for $C_{11}H_7F_5N_2ONa^+$ 301.0376, found 301.0379.

1-methyl-2-oxo-3-(difluoromethyl)-1,2-dihydroquinoxaline-6-carbonitrile (3ag)

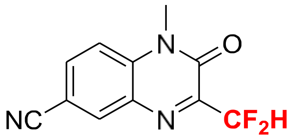

Following the general procedure for the preparation of products **3** (**2**: 0.6 mmol, 3.0 equiv., LiOH: 0.6 mmol, 3.0 equiv.), **3ag** was purified by silica gel chromatography (gradient eluent: PE/DCM/EtOAc = from 40/10/1 to 20/10/1) as a yellow solid (21.6 mg, 46%), Mp: 199.1-200.6 °C. ^{1}H NMR (500 MHz, $CDCl_3$) δ 8.31 (d, *J* = 2.0 Hz, 1H), 7.91 (dd, *J* = 8.7, 2.0 Hz, 1H), 7.49 (d, *J* = 8.7 Hz, 1H), 6.91 (t, *J* = 53.3 Hz, 1H), 3.76 (s, 3H). ^{13}C NMR (126 MHz, $CDCl_3$) δ 152.7, 150.8 (t, *J* = 23.1 Hz), 137.1, 135.7, 134.9, 131.4, 117.3, 115.3, 109.8 (t, *J* = 243.2 Hz) 107.9, 29.4. ^{19}F NMR (471 MHz, $CDCl_3$) δ -124.7 (d, *J* = 53.3 Hz, 2F). HRMS (ESI): *m/z* [M+H]$^+$ calcd for $C_{11}H_8F_2N_3O^+$ 236.0635, found 236.0636.

Methyl-3-(difluoromethyl)-1-methyl-2-oxo-1,2- dihydroquinoxaline-6-carboxylate (3ah)

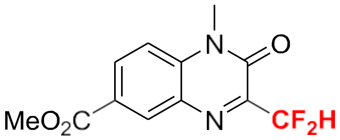

Following the general procedure for the preparation of products **3** (**2**: 0.6 mmol, 3.0 equiv., LiOH: 0.6 mmol, 3.0 equiv.), **3ah** was purified by silica gel chromatography (gradient eluent: PE/DCM/EtOAc = from 60/10/1 to 30/10/1) as a white solid (28.3 mg, 53%). ^{1}H NMR (500 MHz, $CDCl_3$) δ 8.66 (d, *J* = 2.1 Hz, 1H), 8.32 (dd, *J* = 8.9, 2.0 Hz, 1H), 7.43 (d, *J* = 8.8 Hz, 1H), 6.93 (t, *J* = 53.5 Hz, 1H), 3.98 (s, 3H), 3.76 (s, 3H). ^{13}C NMR (126 MHz, $CDCl_3$) δ 165.5, 153.0, 149.6 (t, *J* = 23.0 Hz), 137.1, 133.2, 133.2, 131.2, 126.4, 114.1, 110.0 (t, *J* = 242.3 Hz), 52.6, 29.3. ^{19}F NMR (471 MHz, $CDCl_3$) δ -124.54 (d, *J* = 53.9 Hz, 2F). MS (ESI): *m/z* 290.91 (M+Na$^+$).

6-(tert-butyl)-3-(difluoromethyl)-1-methylquinoxalin-2(1*H*)-one (3ai)

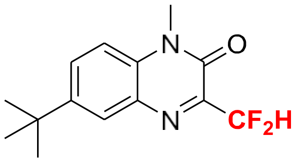

Following the general procedure for the preparation of products **3**, **3ai** was purified by silica gel chromatography (gradient eluent: PE/DCM/EtOAc = from 90/10/1 to 60/10/1) as a yellow solid (29.2 mg, 55%), Mp: 131.9-133.4 °C. ^{1}H NMR (500 MHz, $CDCl_3$) δ 8.01 (d, *J* = 2.3 Hz, 1H), 7.75 (dd, *J* = 8.8, 2.3 Hz, 1H), 7.34 (d, *J* = 8.8 Hz, 1H), 6.98 (t, *J* = 53.8 Hz, 1H), 3.73 (s, 3H), 1.39 (s, 9H). ^{13}C NMR (126 MHz, $CDCl_3$) δ 153.3, 148.4 (t, *J* = 22.2 Hz), 147.9, 131.7, 131.7, 130.6, 127.8, 113.6, 110.0 (t, *J* = 241.4 Hz), 34.6, 31.2, 29.0. ^{19}F NMR (471 MHz, $CDCl_3$) δ

-124.33 (d, *J* = 53.8 Hz, 2F). MS (ESI): *m/z* 288.93 (M+Na$^+$); HRMS (ESI): *m/z* [M+Na]$^+$ calcd for $C_{14}H_{16}F_2N_2ONa^+$ 289.1128, found 289.1128.

7-fluoro-3-(difluoromethyl)-1-methylquinoxalin-2(1*H*)-one (3aj)

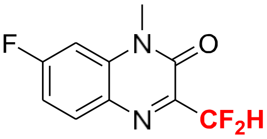

Following the general procedure for the preparation of products **3**, **3aj** was purified by silica gel chromatography (gradient eluent: PE/DCM/EtOAc = from 90/10/1 to 60/10/1) as a white solid (22.9 mg, 50%), Mp: 142.5-143.4 °C. ^{1}H NMR (500 MHz, $CDCl_3$) δ 8.00 (dd, *J* = 8.9, 5.9 Hz, 1H), 7.15 (ddd, *J* = 8.9, 7.9, 2.6 Hz, 1H), 7.06 (dd, *J* = 9.8, 2.6 Hz, 1H), 6.93 (t, *J* = 53.7 Hz, 1H), 3.70 (s, 3H). ^{13}C NMR (151 MHz, $CDCl_3$) δ 164.9 (d, *J* = 254.9 Hz), 153.1, 147.5 (td, *J* = 22.6, 3.8 Hz), 135.8 (d, *J* = 11.9 Hz), 133.7 (d, *J* = 10.8 Hz), 128.7 (d, *J* = 2.2 Hz), 112.6 (d, *J* = 23.7 Hz), 110.1 (t, *J* = 241.7 Hz), 101.0 (d, *J* = 28.0 Hz), 29.3. ^{19}F NMR (471 MHz, $CDCl_3$) δ -102.70 (td, *J* = 9.0, 6.2 Hz, 1F), -124.26 (d, *J* = 53.6 Hz, 2F). MS (ESI): *m/z* 250.91 (M+Na$^+$); HRMS (ESI): *m/z* [M+Na]$^+$ calcd for $C_{10}H_7F_3N_2O^+$ 251.0408, found 251.0412.

7-chloro-3-(difluoromethyl)-1-methylquinoxalin-2(1*H*)-one (3ak)

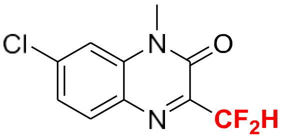

Following the general procedure for the preparation of products **3** (**2**: 0.6 mmol, 3.0 equiv., LiOH: 0.6 mmol, 3.0 equiv.), **3ak** was purified by silica gel chromatography (gradient eluent: PE/DCM/EtOAc = from 90/10/1 to 60/10/1) as a white solid (26.6 mg, 54%), Mp: 160.4-161.8 °C. ^{1}H NMR (500 MHz, $CDCl_3$) δ 7.92 (d, *J* = 8.4 Hz, 1H), 7.39 (d, *J* = 8.2 Hz, 2H), 6.93 (t, *J* = 53.6 Hz, 1H), 3.71 (s, 3H). ^{13}C NMR (126 MHz, $CDCl_3$) δ 152.9, 148.6 (t, *J* = 22.6 Hz), 139.0, 134.9, 132.5, 130.4, 125.0, 114.1, 110.0 (t, *J* = 241.9 Hz), 29.1. ^{19}F NMR (471 MHz, $CDCl_3$) δ -124.4 (d, *J* = 53.7 Hz, 2F). MS (ESI): *m/z* 266.89 (M+Na$^+$); HRMS (ESI): *m/z* [M+Na]$^+$ calcd for $C_{10}H_7ClF_2N_2O^+$ 267.0113, found 267.0117.

7-bromo-3-(difluoromethyl)-1-methylquinoxalin-2(1*H*)-one (3al)

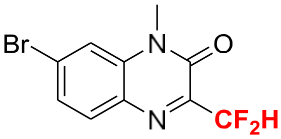

Following the general procedure for the preparation of products **3** (**2**: 0.6 mmol, 3.0 equiv., LiOH: 0.6 mmol, 3.0 equiv.), **3al** was purified by silica gel chromatography (gradient eluent: PE/DCM/EtOAc = from 90/10/1 to 60/10/1) as a yellow solid (26.6 mg, 46%). ^{1}H NMR (500 MHz, $CDCl_3$) δ 7.84 (d, *J* = 8.4 Hz, 1H), 7.54 (d, *J* = 7.9 Hz, 2H), 6.92 (t, *J* = 53.6 Hz, 1H), 3.71 (s, 3H). ^{13}C NMR (126 MHz, $CDCl_3$) δ 152.9, 148.9 (t, *J* = 22.7 Hz), 135.0, 132.6, 130.7, 127.8, 127.3, 117.1, 110.0 (t, *J* = 242.1 Hz), 29.1. ^{19}F NMR (471 MHz, $CDCl_3$) δ -124.48 (d, *J* = 53.7 Hz, 2F). MS (ESI): *m/z* 310.77 (M+Na$^+$).

1-methyl-2-oxo-3-(difluoromethyl)-1,2-dihydroquinoxaline-7-carbonitrile (3am)

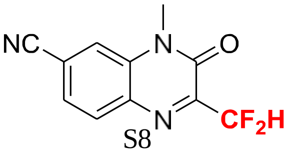

Following the general procedure for the preparation of products **3** (**2**: 0.6 mmol, 3.0 equiv., LiOH: 0.6 mmol, 3.0 equiv.), **3am** was purified by silica gel chromatography (gradient eluent: PE/DCM/EtOAc = from 60/10/2 to 30/10/2) as a yellow solid (15.3 mg, 33%), Mp: 186.9-188.7 °C. ^{1}H NMR (500 MHz, $CDCl_3$) δ 8.11 (d, *J* = 8.1 Hz, 1H), 7.70 – 7.65 (m, 2H), 6.94 (t, *J* = 53.4 Hz, 1H), 3.75 (s, 3H). ^{13}C NMR (126 MHz, $CDCl_3$) δ 152.6, 151.7 (t, *J* = 22.9 Hz), 134.4, 133.8, 132.4, 127.0, 118.2, 117.5, 115.7, 109.6 (t, *J* = 242.9 Hz), 29.3. ^{19}F NMR (377 MHz, $CDCl_3$) δ -125.0 (d, *J* = 53.4 Hz, 2F). HRMS (ESI): *m/z* $[M+Na]^+$ calcd for $C_{11}H_7F_2N_3O^+$ 258.0455, found 258.0456.

7-trifluoromethyl-3-(difluoromethyl)-1-methylquinoxalin-2(1*H*)-one (3an)

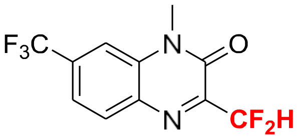

Following the general procedure for the preparation of products **3** (**2**: 0.6 mmol, 3.0 equiv., LiOH: 0.6 mmol, 3.0 equiv.), **3an** was purified by silica gel chromatography (gradient eluent: PE/DCM/EtOAc = from 90/10/1 to 60/10/1) as a white solid (24.6 mg, 44%), Mp: 157.7-159.5 °C. ^{1}H NMR (500 MHz, $CDCl_3$) δ 8.13 (d, *J* = 8.3 Hz, 1H), 7.73 – 7.54 (m, 2H), 6.96 (t, *J* = 53.5 Hz, 1H), 3.78 (s, 3H). ^{13}C NMR (126 MHz, $CDCl_3$) δ 152.8, 151.1 (t, *J* = 22.8 Hz), 134.1, 133.8, 133.4, 132.3, 123.3 (d, *J* = 273.2 Hz), 120.8 (d, *J* = 3.6 Hz), 111.5 (q, *J* = 4.2 Hz), 109.7 (t, *J* = 243.2 Hz), 29.2. ^{19}F NMR (471 MHz, $CDCl_3$) δ -62.7(s, 2F), -124.9 (d, *J* = 53.5 Hz, 2F). MS (ESI): *m/z* 300.90 ($M+Na^+$); HRMS (ESI): *m/z* $[M+Na]^+$ calcd for $C_{11}H_7F_5N_2ONa^+$ 301.0376, found 301.0383.

Methyl-3-(difluoromethyl)-1-methyl-2-oxo-1,2- dihydroquinoxaline-7-carboxylate (3ao)

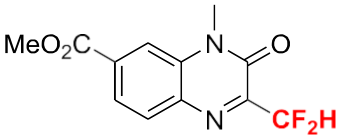

Following the general procedure for the preparation of products **3** (**2**: 0.6 mmol, 3.0 equiv., LiOH: 0.6 mmol, 3.0 equiv.), **3ao** was purified by silica gel chromatography (gradient eluent: PE/DCM/EtOAc = 30/10/1) as yellow solid (21.9 mg, 41%), Mp: 191.1-191.9 °C. ^{1}H NMR (500 MHz, $CDCl_3$) δ 8.08 (t, *J* = 1.1 Hz, 1H), 8.06 (d, *J* = 1.1 Hz, 2H), 6.96 (t, *J* = 53.5 Hz, 1H), 4.01 (s, 3H), 3.79 (s, 3H). ^{13}C NMR (126 MHz, $CDCl_3$) δ 165.6, 153.0, 150.8 (t, *J* = 22.4 Hz), 134.3, 133.9, 133.4, 131.6, 124.9, 115.7, 109.8 (t, *J* = 242.4 Hz), 52.9, 29.3. ^{19}F NMR (471 MHz, $CDCl_3$) δ -124.8 (d, *J* = 53.7 Hz, 2F). MS (ESI): *m/z* 290.91 ($M+Na^+$). HRMS (ESI): *m/z* $[M+Na]^+$ calcd for $C_{12}H_{10}F_2N_2O_3Na^+$ 291.0557, found 291.0561.

7-(tert-butyl)-3-(difluoromethyl)-1-methylquinoxalin-2(1*H*)-one (3ap)

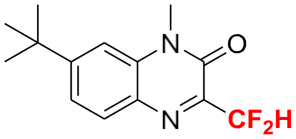

Following the general procedure for the preparation of products **3**, **3ap** was purified by silica gel chromatography (gradient eluent: PE/DCM/EtOAc = from 90/10/1 to 60/10/1) as a yellow solid (26.9 mg, 51%), Mp: 125.6-126.8 °C. ^{1}H NMR (500 MHz, $CDCl_3$) δ 7.92 (d, *J* = 8.5 Hz, 1H), 7.49 (dd, *J* = 8.5, 1.9 Hz, 1H), 7.33 (d, *J* = 1.9 Hz, 1H), 6.96 (t, *J* = 53.8 Hz, 1H), 3.76 (s, 3H), 1.42 (s, 9H). ^{13}C NMR (126 MHz, $CDCl_3$) δ 157.1, 153.5, 147.6 (t, *J* = 22.4 Hz), 133.7, 130.9, 130.1, 122.4, 110.3, 110.2(t, *J* = 241.9 Hz), 35.7, 31.2, 28.8. ^{19}F NMR (471 MHz, $CDCl_3$) δ -124.1 (d, *J* = 54.1 Hz, 2F). MS (ESI): *m/z* 288.96 ($M+Na^+$); HRMS (ESI): *m/z* $[M+Na]^+$ calcd for $C_{14}H_{16}F_2N_2ONa^+$

289.1128, found 289.1130.

3-(difluoromethyl)-1,5-dimethylquinoxalin-2(1*H*)-one (3aq)

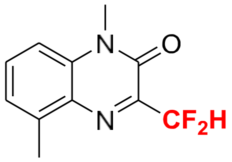

Following the general procedure for the preparation of products **3** (**2**: 0.6 mmol, 3.0 equiv., LiOH: 0.6 mmol, 3.0 equiv., and PC I was used as photocatalyst), **3aq** was purified by silica gel chromatography (gradient eluent: PE/DCM/EtOAc = from 90/10/1 to 60/10/1) as a yellow solid (23.0 mg, 51%). ^{1}H NMR (500 MHz, $CDCl_3$) δ 7.55 (t, *J* = 7.9 Hz, 1H), 7.31 – 7.25 (m, 1H), 7.20 (d, *J* = 8.5 Hz, 1H), 6.92 (t, *J* = 53.8 Hz, 1H), 3.72 (s, 3H), 2.72 (s, 3H). ^{13}C NMR (126 MHz, $CDCl_3$) δ 153.1, 146.7 (t, *J* = 22.7 Hz), 140.7, 134.3, 132.4, 130.6, 125.6, 111.74, 110.75(t, *J* = 241.9 Hz), 29.1, 17.4. ^{19}F NMR (471 MHz, $CDCl_3$) δ -123.71 (d, *J* = 54.2 Hz, 2F). MS (ESI): *m/z* 246.92 (M+Na$^+$).

6,7-difluoro-1-methyl-3-(difluoromethyl)quinoxalin-2(1*H*)-one (3ar)

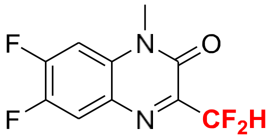

Following the general procedure for the preparation of products **3** , **3ar** was purified by silica gel chromatography (gradient eluent: PE/DCM/EtOAc = from 90/10/1 to 60/10/1) as a white solid (26.5 mg, 54%), Mp: 107.0-107.9 °C. ^{1}H NMR (500 MHz, $CDCl_3$) δ 7.83 (t, *J* = 8.9 Hz, 1H), 7.19 (dd, *J* = 11.0, 6.9 Hz, 1H), 6.92 (t, *J* = 53.5 Hz, 1H), 3.70 (s, 3H). ^{13}C NMR (151 MHz, $CDCl_3$) δ 154.0 (d, *J* = 14.1 Hz), 152.8, 152.3 (d, *J* = 14.4 Hz), 149.1 (td, *J* = 22.8, 3.9 Hz), 148.0 (d, *J* = 14.3 Hz), 146.4 (d, *J* = 14.0 Hz), 131.7 (d, *J* = 9.2 Hz), 128.1 (dd, *J* = 9.3, 2.9 Hz), 119.0 (dd, *J* = 18.2, 2.6 Hz), 109.9 (t, *J* = 242.1 Hz), 102.7 (d, *J* = 23.2 Hz), 29.6. ^{19}F NMR (471 MHz, $CDCl_3$) δ -124.5 (d, *J* = 53.6 Hz, 2F), -125.8 (ddd, *J* = 22.4, 11.1, 8.1 Hz, 1F), -140.0 (ddd, *J* = 22.0, 10.0, 6.9 Hz, 1F). MS (ESI): *m/z* 268.87 (M+Na$^+$); HRMS (ESI): *m/z* [M+H]$^+$ calcd for $C_{10}H_7F_4N_2O^+$ 247.0659, found 247.0656.

6,7-dichloro-3-(difluoromethyl)-1-methylquinoxalin-2(1*H*)-one (3as)

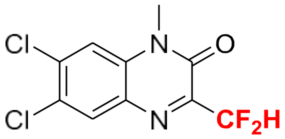

Following the general procedure for the preparation of products **3** (**2**: 0.6 mmol, 3.0 equiv., LiOH: 0.6 mmol, 3.0 equiv.), **3as** was purified by silica gel chromatography (gradient eluent: PE/DCM/EtOAc = from 90/10/1 to 60/10/1) as a white solid (25.7 mg, 46%). ^{1}H NMR (500 MHz, $CDCl_3$) δ 8.09 (s, 1H), 7.49 (s, 1H), 6.91 (t, *J* = 53.5 Hz, 1H), 3.70 (s, 3H). ^{13}C NMR (126 MHz, $CDCl_3$) δ 152.6, 149.9 (t, *J* = 22.7 Hz), 137.2, 133.3, 132.1, 130.8, 128.5, 115.5, 109.8 (t, *J* = 242.5 Hz), 29.3. ^{19}F NMR (471 MHz, $CDCl_3$) δ -124.6 (d, *J* = 53.6 Hz, 2F). MS (ESI): *m/z* 300.88 (M+Na$^+$).

6,7-dibromo-1-methyl-3-(difluoromethyl)quinoxalin-2(1*H*)-one (3at)

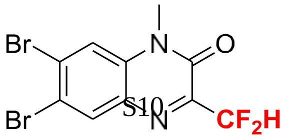

Following the general procedure for the preparation of products **3** (**2**: 0.8 mmol, 4.0 equiv., LiOH: 0.8 mmol, 4.0 equiv.), **3at** was purified by silica gel chromatography (gradient eluent: PE/DCM/EtOAc = from 90/10/1 to 60/10/1) as a yellow solid (25.9 mg, 35%), Mp: 215.8-218.0 °C. ^{1}H NMR (400 MHz, $CDCl_3$) δ 8.24 (s, 1H), 7.66 (s, 1H), 6.90 (t, *J* = 53.5 Hz, 1H), 3.69 (s, 3H). ^{13}C NMR (151 MHz, $CDCl_3$) δ 152.6, 150.1 (t, *J* = 22.9 Hz), 135.2, 133.8, 131.5, 129.7, 119.9, 118.7, 109.8 (t, *J* = 242.5 Hz), 29.3. ^{19}F NMR (471 MHz, $CDCl_3$) δ -124.66 (d, *J* = 53.5 Hz, 2F). MS (ESI): *m/z* 390.61 (M+Na$^+$); HRMS (ESI): *m/z* [M-H]$^+$ calcd for $C_{10}H_5Br_2F_2N_2O^+$ 366.8893, found 366.8890.

1,6,7-trimethyl-3-(difluoromethyl)quinoxalin-2(1*H*)-one (3au)

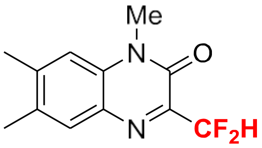

Following the general procedure for the preparation of products **3**, **3au** was purified by silica gel chromatography (gradient eluent: PE/DCM/EtOAc = 100/10/1) as a yellow solid (27.2 mg, 58%). ^{1}H NMR (500 MHz, $CDCl_3$) δ 7.72 (s, 1H), 7.13 (s, 1H), 6.94 (t, *J* = 53.9 Hz, 1H), 3.70 (s, 3H), 2.45 (s, 3H), 2.36 (s, 3H). ^{13}C NMR (126 MHz, $CDCl_3$) δ 153.4, 147.2 (t, *J* = 22.3 Hz), 143.2, 133.6, 132.1, 131.3, 130.4, 114.4, 110.3 (t, *J* = 241.1 Hz), 28.9, 20.8, 19.2. ^{19}F NMR (471 MHz, $CDCl_3$) δ -124.0 (d, *J* = 53.8 Hz, 2F). MS (ESI): *m/z* 260.92 (M+Na$^+$).

3-(difluoromethyl)-1-methylbenzo[g]quinoxalin-2(1*H*)-one (3av)

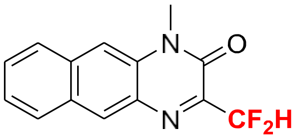

Following the general procedure for the preparation of products **3** （PC I was used as photocatalyst）, **3av** was purified by silica gel chromatography (gradient eluent: PE/DCM/EtOAc = from 90/10/1 to 60/10/1) as a yellow solid (18.1 mg, 35%). ^{1}H NMR (500 MHz, $CDCl_3$) δ 8.53 (s, 1H), 8.01 (d, *J* = 8.3 Hz, 1H), 7.93 (d, *J* = 8.4 Hz, 1H), 7.68 – 7.61 (m, 2H), 7.53 (ddd, *J* = 8.1, 6.8, 1.2 Hz, 1H), 6.99 (t, *J* = 53.7 Hz, 1H), 3.79 (s, 3H). ^{13}C NMR (126 MHz, $CDCl_3$) δ 153.0, 149.2 (t, *J* = 22.5 Hz), 134.8, 131.6, 131.5, 131.0, 129.8, 129.2, 128.9, 127.3, 125.9, 110.5, 110.1(t, *J* = 241.9 Hz), 28.9. ^{19}F NMR (471 MHz, $CDCl_3$) δ -124.31 (d, *J* = 53.7 Hz, 2F). MS (ESI): *m/z* 282.91 (M+Na$^+$).

3-(difluoromethyl)quinoxalin-2(1*H*)-one (3ba)

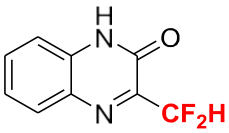

Following the general procedure for the preparation of products **3**(**2**: 0.6 mmol, 3.0 equiv., LiOH: 0.6 mmol, 3.0 equiv., and Perylene was used as photocatalyst), **3ba** was purified by silica gel chromatography (gradient eluent: PE/DCM/EtOAc = from 90/10/1 to 30/10/1) as a white solid (21.6 mg, 55%). ^{1}H NMR (500 MHz, DMSO-*d*$_6$) δ 12.84 (s, 1H), 7.88 (d, *J* = 8.0 Hz, 1H), 7.66 (t, *J* = 7.7 Hz, 1H), 7.38 (t, *J* = 8.1 Hz, 2H), 7.06 (t, *J* = 53.3 Hz, 1H). ^{13}C NMR (126 MHz, DMSO-*d*$_6$) δ 153.7, 150.2 (t, *J* = 21.1 Hz), 133.3, 132.8, 131.2, 130.0, 124.4, 116.2, 110.8 (t, *J* = 239.4 Hz). ^{19}F NMR (471 MHz, DMSO-*d*$_6$) δ -124.29 (d, *J* = 52.8 Hz, 2F). MS (ESI): *m/z* 194.89 (M-H$^+$).

1-benzyl-3-(difluoromethyl)quinoxalin-2(1*H*)-one (3bb)

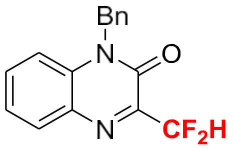

Following the general procedure for the preparation of products **3**, **3bb** was purified by silica gel chromatography (gradient eluent: PE/DCM/EtOAc = from 240/40/1 to 120/40/1) as a yellow solid (28.8 mg, 50%). ^{1}H NMR (500 MHz, $CDCl_3$) δ 7.99 (dd, *J* = 8.1, 1.5 Hz, 1H), 7.55 (ddd, *J* = 8.6, 7.2, 1.6 Hz, 1H), 7.40 – 7.22 (m, 7H), 7.02 (t, *J* = 53.7 Hz, 1H), 5.50 (s, 2H). ^{13}C NMR (126 MHz, $CDCl_3$) δ 153.4, 148.8 (t, *J* = 22.5 Hz), 134.6, 133.4, 132.7, 132.2, 131.6, 129.1, 128.0, 126.9, 124.5, 114.8, 110.1 (t, *J* = 241.8 Hz), 45.9. ^{19}F NMR (471 MHz, $CDCl_3$) δ -124.1 (d, *J* = 53.7 Hz, 2F). MS (ESI): *m/z* 308.89 (M+Na$^+$).

6,7-dimethyl-3-(difluoromethyl)-1-benzylquinoxalin-2(1*H*)-one (3bc)

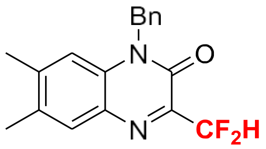

Following the general procedure for the preparation of products **3**, **3bc** was purified by silica gel chromatography (gradient eluent: PE/DCM/EtOAc = from 90/10/1 to 60/10/1) as a yellow solid (31.6 mg, 50%), Mp: 132.0-133.2 °C. ^{1}H NMR (400 MHz, $CDCl_3$) δ 7.74 (s, 1H), 7.39 – 7.20 (m, 6H), 7.22 – 6.80 (m, 2H), 5.48 (s, 2H), 2.33 (d, *J* = 5.3 Hz, 6H). ^{13}C NMR (101 MHz, $CDCl_3$) δ 153.5, 147.4 (t, *J* = 22.3 Hz), 143.2, 134.8, 133.7, 131.6, 131.4, 130.7, 129.0, 127.9, 127.0, 115.1, 110.2 (t, *J* = 241.3 Hz), 45.7, 20.9, 19.2. ^{19}F NMR (377 MHz, $CDCl_3$) δ -123.8 (d, *J* = 53.9 Hz, 2F). MS (ESI): *m/z* 336.91 (M+Na$^+$); HRMS (ESI): *m/z* [M+Na]$^+$ calcd for $C_{18}H_{16}F_2N_2ONa^+$ 337.1128, found 337.1130.

6,7-difluoro-3-(difluoromethyl)-1-benzylquinoxalin-2(1*H*)-one (3bd)

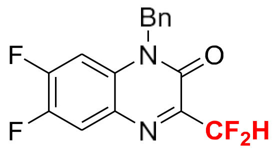

Following the general procedure for the preparation of products **3** (**2**: 0.6 mmol, 3.0 equiv., LiOH: 0.6 mmol, 3.0 equiv.), **3bd** was purified by silica gel chromatography (gradient eluent: PE/DCM/EtOAc = from 90/10/1 to 60/10/1) as a yellow solid (32.8 mg, 51%), Mp: 84.7-86.3 °C. ^{1}H NMR (500 MHz, $CDCl_3$) δ 7.81 (t, *J* = 8.9 Hz, 1H), 7.43 – 7.18 (m, 5H), 7.12 (dd, *J* = 11.2, 6.9 Hz, 1H), 6.98 (t, *J* = 53.5 Hz, 1H), 5.45 (s, 2H). ^{13}C NMR (151 MHz, $CDCl_3$) δ 153.9 (d, *J* = 14.6 Hz), 153.0, 152.2 (d, *J* = 14.5 Hz), 149.2 (td, *J* = 22.9, 4.0 Hz), 148.0 (d, *J* = 14.0 Hz), 146.4 (d, *J* = 14.1 Hz), 133.8, 131.0 (d, *J* = 9.4 Hz), 129.4, 128.7 – 128.3 (m), 126.90, 119.1 (d, *J* = 2.6 Hz), 119.0 (d, *J* = 2.6 Hz), 109.8 (t, *J* = 242.2 Hz), 103.5 (d, *J* = 23.7 Hz), 46.5. ^{19}F NMR (377 MHz, $CDCl_3$) δ -124.3 (d, *J* = 53.6 Hz, 2F), -125.7 (ddd, *J* = 22.3, 11.2, 8.2 Hz, 1F), -139.7 (ddd, *J* = 22.4, 10.0, 7.0 Hz, 1F). MS (ESI): *m/z* 336.91 (M+Na$^+$); HRMS (ESI): *m/z* [M+Na]$^+$ calcd for $C_{16}H_{10}F_4N_2ONa^+$ 345.0627, found 345.0621.

3-(difluoromethyl)-1-ethylquinoxalin-2(1*H*)-one (3be)

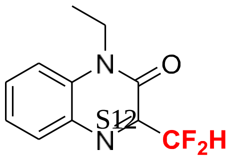

Following the general procedure for the preparation of products **3**, **3be** was purified by silica gel chromatography (gradient eluent: PE/DCM/EtOAc = from 90/10/1 to 60/10/1) as a white solid (27 mg, 60%). ^{1}H NMR (500 MHz, $CDCl_3$) δ 8.02 (dd, *J* = 8.1, 1.5 Hz, 1H), 7.69 (ddd, *J* = 8.7, 7.3, 1.6 Hz, 1H), 7.48 – 7.37 (m, 2H), 6.98 (t, *J* = 53.7 Hz, 1H), 4.36 (q, *J* = 7.2 Hz, 2H), 1.41 (t, *J* = 7.2 Hz, 3H). ^{13}C NMR (151 MHz, $CDCl_3$) δ 152.8, 148.7 (t, *J* = 22.4 Hz), 133.1, 132.6, 132.3, 131.8, 124.2, 113.8, 110.1 (t, *J* = 241.6 Hz), 37.4, 12.4. ^{19}F NMR (471 MHz, $CDCl_3$) δ -124.31 (d, *J* = 53.6 Hz, 2F). MS (ESI): *m/z* 246.95 (M+Na$^+$).

1-butyl-3-(difluoromethyl)quinoxalin-2(1*H*)-one (3bf)

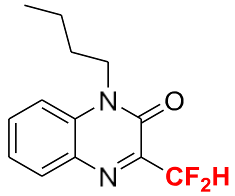

Following the general procedure for the preparation of products **3**, **3bf** was purified by silica gel chromatography (gradient eluent: PE/DCM/EtOAc = from 90/10/1 to 60/10/1) as a white solid (25.7 mg, 51%). ^{1}H NMR (500 MHz, $CDCl_3$) δ 8.01 (dd, *J* = 8.0, 1.5 Hz, 1H), 7.68 (ddd, *J* = 8.7, 7.3, 1.6 Hz, 1H), 7.48 – 7.34 (m, 2H), 6.97 (t, *J* = 53.8 Hz, 1H), 4.31 – 4.23 (m, 2H), 1.83 – 1.72 (m, 2H), 1.62 – 1.42 (m, 2H), 1.01 (t, *J* = 7.4 Hz, 3H). ^{13}C NMR (126 MHz, $CDCl_3$) δ 153.0, 148.6 (t, *J* = 22.3 Hz), 133.3, 132.5, 132.2, 131.7, 124.2, 114.0, 110.0 (t, *J* = 241.6 Hz), 42.2, 29.3, 20.2, 13.7. ^{19}F NMR (471 MHz, $CDCl_3$) δ -124.33 (d, *J* = 53.9 Hz, 2F). MS (ESI): *m/z* 274.93 (M+Na$^+$).

3-(difluoromethyl)-1-(2-oxo-2-phenylethyl)quinoxaline-2(1*H*)-one (3bg)

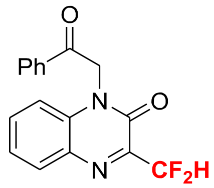

Following the general procedure for the preparation of products **3**, **3bg** was purified by silica gel chromatography (gradient eluent: PE/DCM/EtOAc = from 100/1/10 to 80/1/10) as a yellow solid (35.9 mg, 57%). ^{1}H NMR (500 MHz, DMSO-d_6) δ 8.21 – 8.13 (m, 2H), 8.01 (dd, *J* = 8.1, 1.5 Hz, 1H), 7.86 – 7.68 (m, 2H), 7.67 – 7.57 (m, 3H), 7.53 – 7.46 (m, 1H), 7.13 (t, *J* = 53.1 Hz, 1H), 5.99 (s, 2H). ^{13}C NMR (126 MHz, DMSO-d_6) δ 192.4, 152.9, 148.5 (t, *J* = 22.1 Hz), 134.9, 134.7, 134.2, 133.3, 131.6, 131.0, 129.5, 128.8, 124.9, 115.8, 110.9 (t, *J* = 239.7 Hz), 49.4. ^{19}F NMR (471 MHz, DMSO-d_6) δ -124.1 (d, *J* = 53.2 Hz, 2F). MS (ESI): *m/z* 336.88 (M+Na$^+$).

Ethyl 2-(3-(difluoromethyl)-2-oxoquinoxalin-1(2*H*)-yl)acetate (3bh)

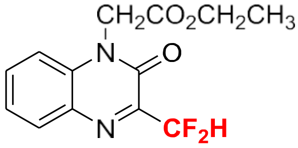

Following the general procedure for the preparation of products **3**, **3bh** was purified by silica gel chromatography (gradient eluent: PE/DCM/EtOAc = from 200/1/10 to 100/1/10) as a yellow solid (28.1 mg, 50%). ^{1}H NMR (500 MHz, $CDCl_3$) δ 8.02 (dd, *J* = 8.1, 1.5 Hz, 1H), 7.66 (ddd, *J* = 8.7, 7.3, 1.5 Hz, 1H), 7.44 (ddd, *J* = 8.2, 7.3, 1.2 Hz, 1H), 7.15 (dd, *J* = 8.4, 1.2 Hz, 1H), 6.95 (t, *J* = 53.6 Hz, 1H), 5.05 (s, 2H), 4.26 (q, *J* = 7.1 Hz, 2H), 1.29 (t, *J* = 7.1 Hz, 3H). ^{13}C NMR (126 MHz,

$CDCl_3$) δ 166.5, 152.8, 148.5 (t, *J* = 22.8 Hz), 133.2, 132.8, 132.0, 131.8, 124.7, 113.5, 110.0 (t, *J* = 241.9 Hz), 62.4, 43.3, 14.1. ^{19}F NMR (471 MHz, $CDCl_3$) δ -124.3 (d, *J* = 53.7 Hz, 2F). MS (ESI): *m/z* 304.91 ($M+Na^+$).

Isopropyl 2-(3-(difluoromethyl)-2-oxoquinoxalin-1(2*H*)-yl)acetate (3bi)

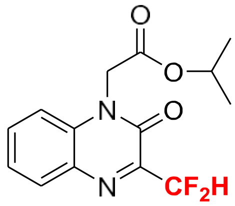

Following the general procedure for the preparation of products **3**, **3bi** was purified by silica gel chromatography (gradient eluent: PE/DCM/EtOAc = from 90/10/1 to 60/10/1) as a light yellow solid (29.8 mg, 50%), Mp: 115.0-117.4 °C. ^1H NMR (500 MHz, $CDCl_3$) δ 8.03 (dd, *J* = 8.1, 1.5 Hz, 1H), 7.65 (ddd, *J* = 8.6, 7.3, 1.5 Hz, 1H), 7.44 (td, *J* = 7.7, 7.3, 1.1 Hz, 1H), 7.13 (dd, *J* = 8.5, 1.1 Hz, 1H), 6.95 (t, *J* = 53.6 Hz, 1H), 5.11 (p, *J* = 6.3 Hz, 1H), 5.02 (s, 2H), 1.27 (d, *J* = 6.3 Hz, 6H). ^{13}C NMR (126 MHz, $CDCl_3$) δ 166.0, 152.8, 148.6 (t, *J* = 22.8 Hz), 133.3, 132.8, 132.0, 131.8, 124.6, 113.5, 110.0 (t, *J* = 242.0 Hz), 70.4, 43.5, 21.7. ^{19}F NMR (471 MHz, $CDCl_3$) δ -124.3 (d, *J* = 53.5 Hz, 2F). MS (ESI): *m/z* 318.87 ($M+Na^+$); HRMS (ESI): *m/z* $[M+ Na]^+$ calcd for $C_{14}H_{14}F_2N_2O_3Na^+$ 319.0870, found 319.0871.

Tert-butyl 2-(3-(difluoromethyl)-2-oxoquinoxalin-1(2*H*)-yl) acetate (3bj)

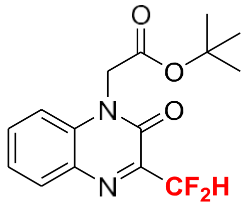

Following the general procedure for the preparation of products **3**, **3bj** was purified by silica gel chromatography (gradient eluent: PE/DCM/EtOAc = from 90/10/1 to 60/10/1) as a yellow solid (31.4 mg, 51%). ^1H NMR (500 MHz, $CDCl_3$) δ 8.03 (dd, *J* = 8.1, 1.5 Hz, 1H), 7.67 (ddd, *J* = 8.7, 7.3, 1.5 Hz, 1H), 7.45 (ddd, *J* = 8.3, 7.3, 1.2 Hz, 1H), 7.16 (dd, *J* = 8.4, 1.1 Hz, 1H), 6.97 (t, *J* = 53.6 Hz, 1H), 4.98 (s, 2H), 1.48 (s, 9H). ^{13}C NMR (126 MHz, $CDCl_3$) δ 165.5, 152.8, 148.6 (t, *J* = 22.8 Hz), 133.3, 132.7, 132.0, 131.7, 124.6, 113.5, 110.0 (t, *J* = 241.9 Hz), 83.7, 44.0, 28.0. ^{19}F NMR (471 MHz, $CDCl_3$) δ -124.3 (d, *J* = 53.7 Hz, 2F). MS (ESI): *m/z* 322.90 ($M+Na^+$).

1-allyl-3-(difluoromethyl)quinoxalin-2(1*H*)-one (3bk)

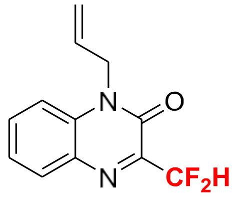

Following the general procedure for the preparation of products **3**, **3bk** was purified by silica gel chromatography (gradient eluent: PE/DCM/EtOAc = from 90/10/1 to 60/10/1) as a yellow solid (17.2 mg, 36%). ^1H NMR (500 MHz, $CDCl_3$) δ 8.03 (dd, *J* = 8.1, 1.5 Hz, 1H), 7.67 (ddd, *J* = 8.6,

7.2, 1.5 Hz, 1H), 7.44 (ddd, *J* = 8.3, 7.3, 1.2 Hz, 1H), 7.38 (dd, *J* = 8.5, 1.2 Hz, 1H), 6.99 (t, *J* = 53.7 Hz, 1H), 5.33 (d, *J* = 10.4 Hz, 1H), 5.23 (d, *J* = 17.2 Hz, 1H), 4.95 (dt, *J* = 5.2, 1.8 Hz, 2H). ^{13}C NMR (126 MHz, $CDCl_3$) δ 152.9, 148.7 (t, *J* = 22.3 Hz), 133.3, 132.6, 132.1, 131.6, 130.0, 124.4, 118.8, 114.5, 110.0 (t, *J* = 241.8 Hz), 44.5. ^{19}F NMR (471 MHz, $CDCl_3$) δ -124.3 (d, *J* = 53.6 Hz, 2F). MS (ESI): *m/z* 258.95 ($M+Na^+$).

3-(difluoromethyl)-1-(prop-2-yn-1-yl)quinoxalin-2(1*H*)-one (3bl)

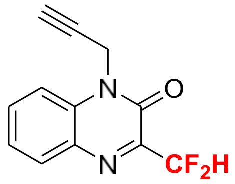

Following the general procedure for the preparation of products **3**, **3bl** was purified by silica gel chromatography (gradient eluent: PE/DCM/EtOAc = from 150/10/1 to 100/10/1) as a yellow solid (19.6 mg, 42%). ^{1}H NMR (500 MHz, $CDCl_3$) δ 8.02 (dd, *J* = 8.1, 1.5 Hz, 1H), 7.73 (ddd, *J* = 8.6, 7.2, 1.5 Hz, 1H), 7.55 (dd, *J* = 8.5, 1.2 Hz, 1H), 7.46 (td, *J* = 7.7, 7.2, 1.2 Hz, 1H), 6.95 (t, *J* = 53.6 Hz, 1H), 5.08 (d, *J* = 2.6 Hz, 2H), 2.33 (t, *J* = 2.6 Hz, 1H). ^{13}C NMR (126 MHz, $CDCl_3$) δ 152.2, 148.6 (t, *J* = 22.7 Hz), 132.8, 132.5, 132.1, 131.6, 124.8, 114.5, 110.0 (t, *J* = 242.0 Hz), 76.1, 73.8, 31.4. ^{19}F NMR (471 MHz, $CDCl_3$) δ -124.20 (d, *J* = 53.7 Hz, 2F). MS (ESI): *m/z* 256.90 ($M+Na^+$).

9. References.

1. Lu, S. L.; Li, X.; Qin, W. B.; Liu, J. J.; Huang, Y. Y.; Wong, H. N. C.; Liu, G. K., Air- and Light-Stable S-(Difluoromethyl)sulfonium Salts: C-Selective Electrophilic Difluoromethylation of beta-Ketoesters and Malonates. *Org. Lett.* **2018,** *20* , 6925-6929.
2. Chen, D.; Wang, Z. J.; Bao, W., Copper-catalyzed cascade syntheses of 2*H*-benzo[b][1,4]thiazin-3(4*H*)-ones and quinoxalin-2(1*H*)-ones through capturing S and N atom respectively from AcSH and TsNH(2). *J Org Chem.* **2010,** *75* , 5768-5771.
3. K. Aoki, T. Obata, Y. Yamazaki, Y. Mori, H. Hirokawa, J.-I. Koseki, T. Hattori, K. Niitsu, S. Takeda, M. Aburada, K. I. Miyamoto., Potent Platelet-Derived Growth Factor-b Receptor (PDGF-bR) Inhibitors: Synthesis and Structure–Activity Relationships of 7-[3-(Cyclohexylmethyl)ureido]-3-{1-methyl-1*H*-pyrrolo[2,3-b]pyridin-3-yl}quinoxalin-2(1*H*)-one Derivatives. *Chem. Pharm. Bull.* **2007**, 55, 255-267.
4. E. J. Jacobsen, R. E. TenBrink, L. S. Stelzer, K. L. Belonga, D. B. Carter, H. K. Im, W. B. Im, V. H. Sethy, A. H. Tang, P. F. VonVoigtlander, J. D. Petke., High-Affinity Partial Agonist Imidazo[1,5-a]quinoxaline Amides, Carbamates, and Ureas at the γ-Aminobutyric Acid A/Benzodiazepine Receptor Complex. *J. Med. Chem.* **1996**, 39, 158-175.
5. Loughran, H. M.; Han, Z.; Wrobel, J. E.; Decker, S. E.; Ruthel, G.; Freedman, B. D.; Harty, R. N.; Reitz, A. B., Quinoxaline-based inhibitors of Ebola and Marburg VP40 egress. *Bioorg Med Chem Lett.* **2016,** *26* , 3429-3435.

10. NMR Spectra for New Compound

3-(difluoromethyl)-1-methylquinoxalin-2(1*H*)-one (3aa)

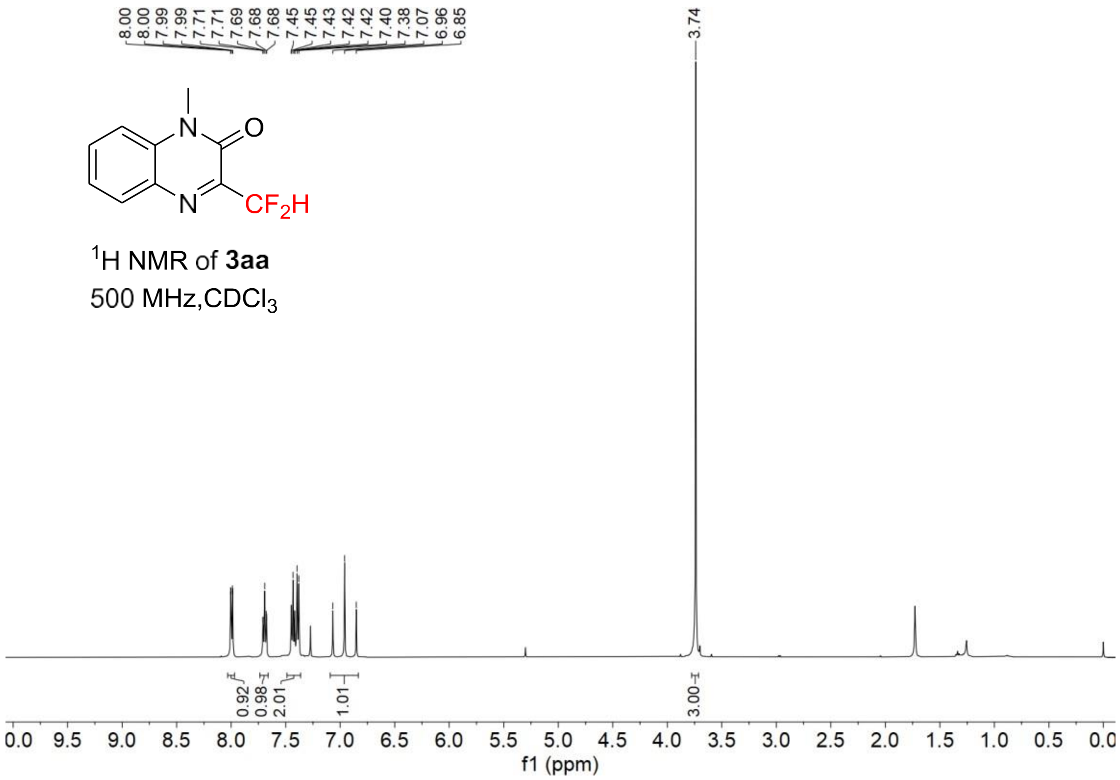
8.00
8.00
7.99
7.99
7.71
7.71
7.69
7.68
7.68
7.45
7.45
7.43
7.42
7.42
7.40
7.38
7.07
6.96
6.85
3.74
N
O
N
CF2H
1H NMR of 3aa
500 MHz,CDCl3
0.92
0.98
2.01
1.01
3.00
0.0 9.5 9.0 8.5 8.0 7.5 7.0 6.5 6.0 5.5 5.0 4.5 4.0 3.5 3.0 2.5 2.0 1.5 1.0 0.5 0.0
f1 (ppm)

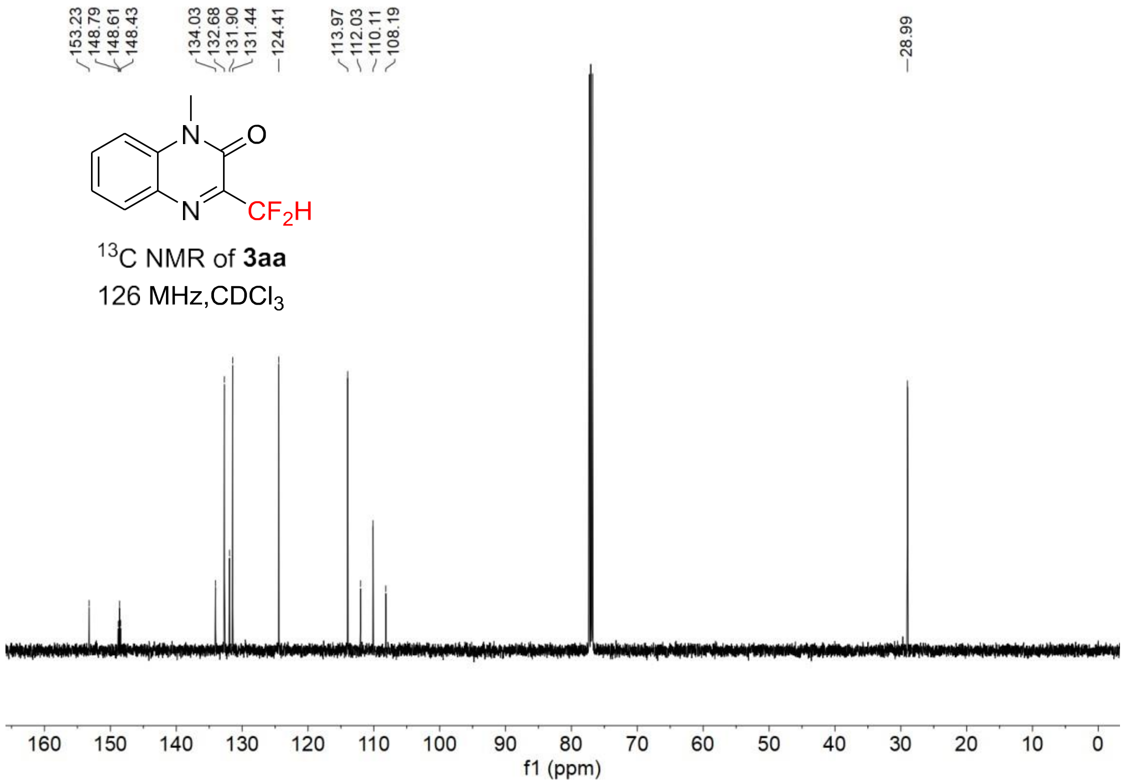
153.23
148.79
148.61
148.43
134.03
132.68
131.90
131.44
124.41
113.97
112.03
110.11
108.19
28.99
N
O
N
CF2H
13C NMR of 3aa
126 MHz,CDCl3
160 150 140 130 120 110 100 90 80 70 60 50 40 30 20 10 0
f1 (ppm)

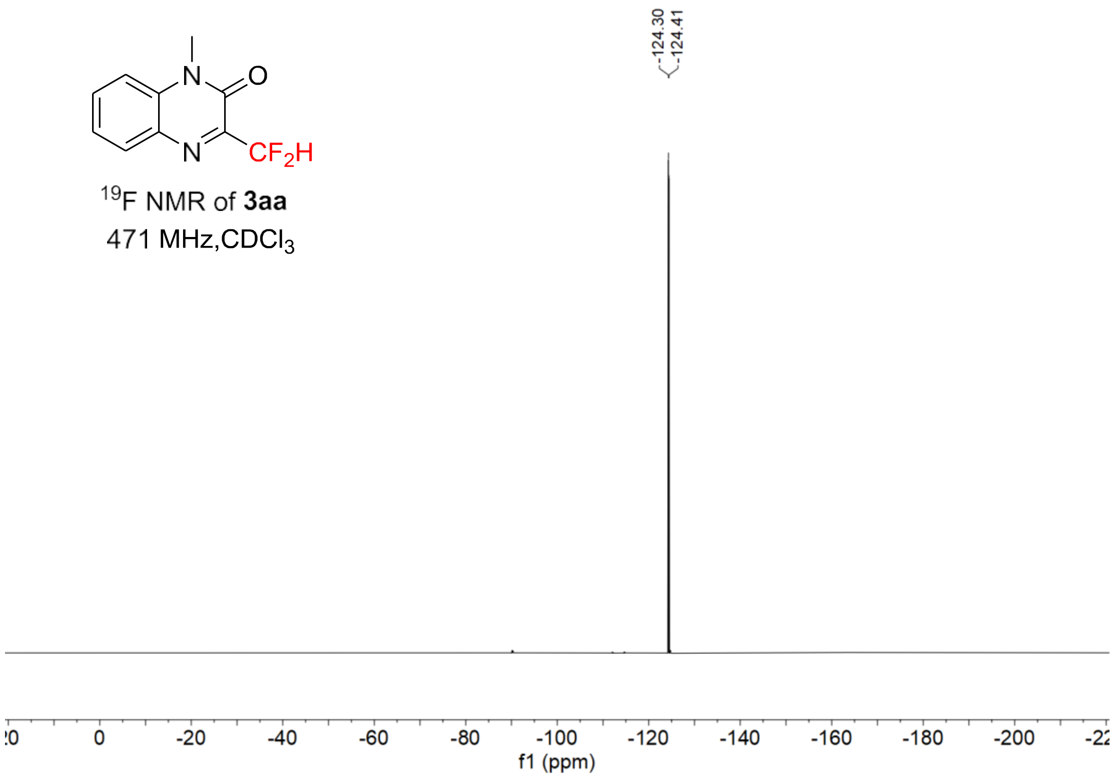

6-fluoro-3-(difluoromethyl)-1-methylquinoxalin-2(1*H*)-one (3ab)

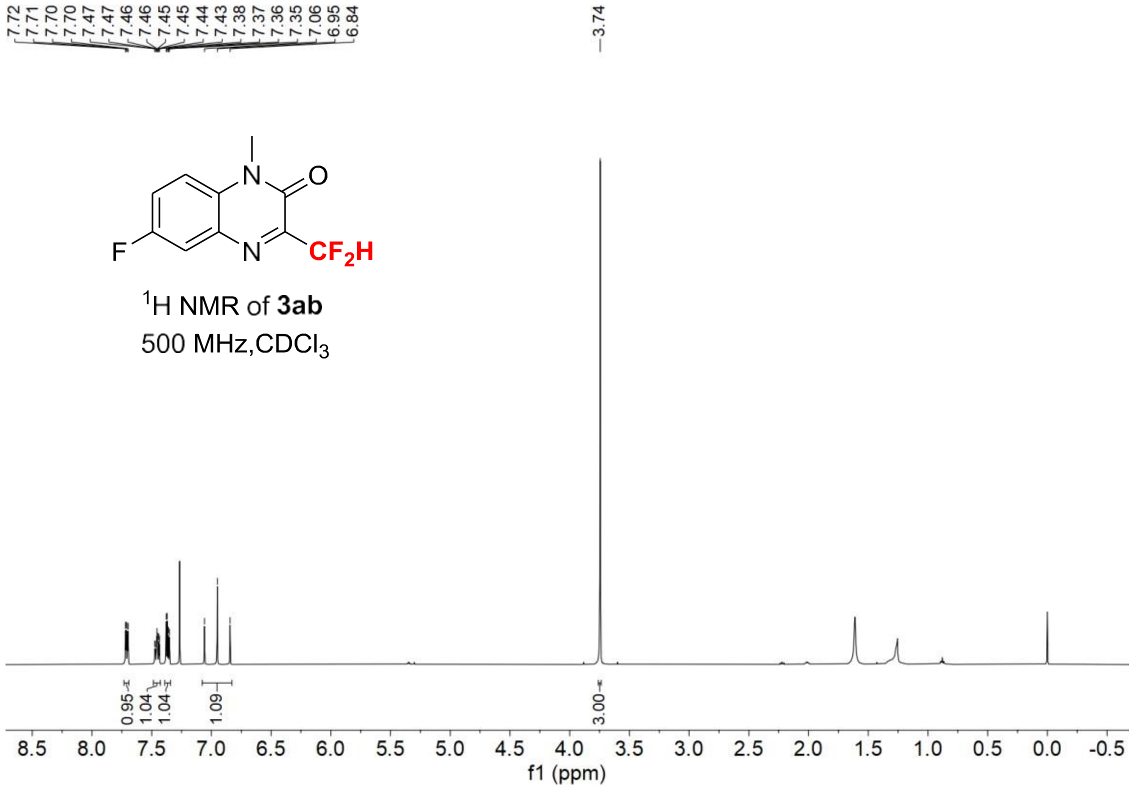

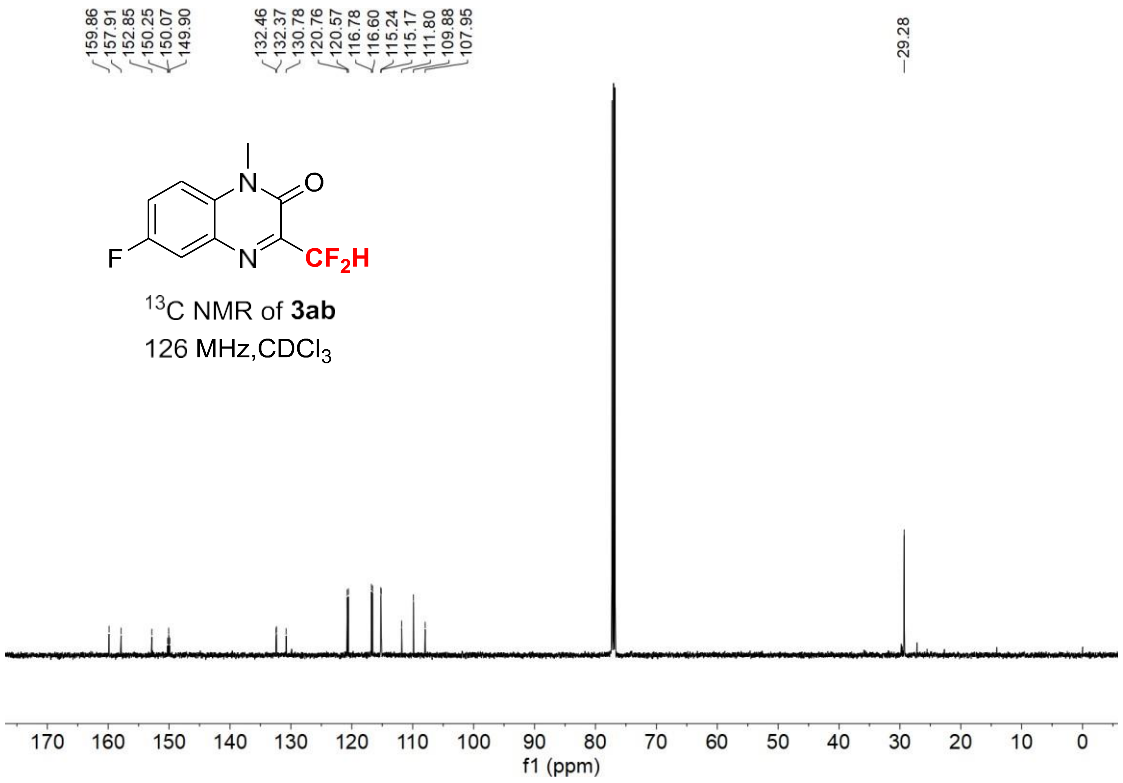

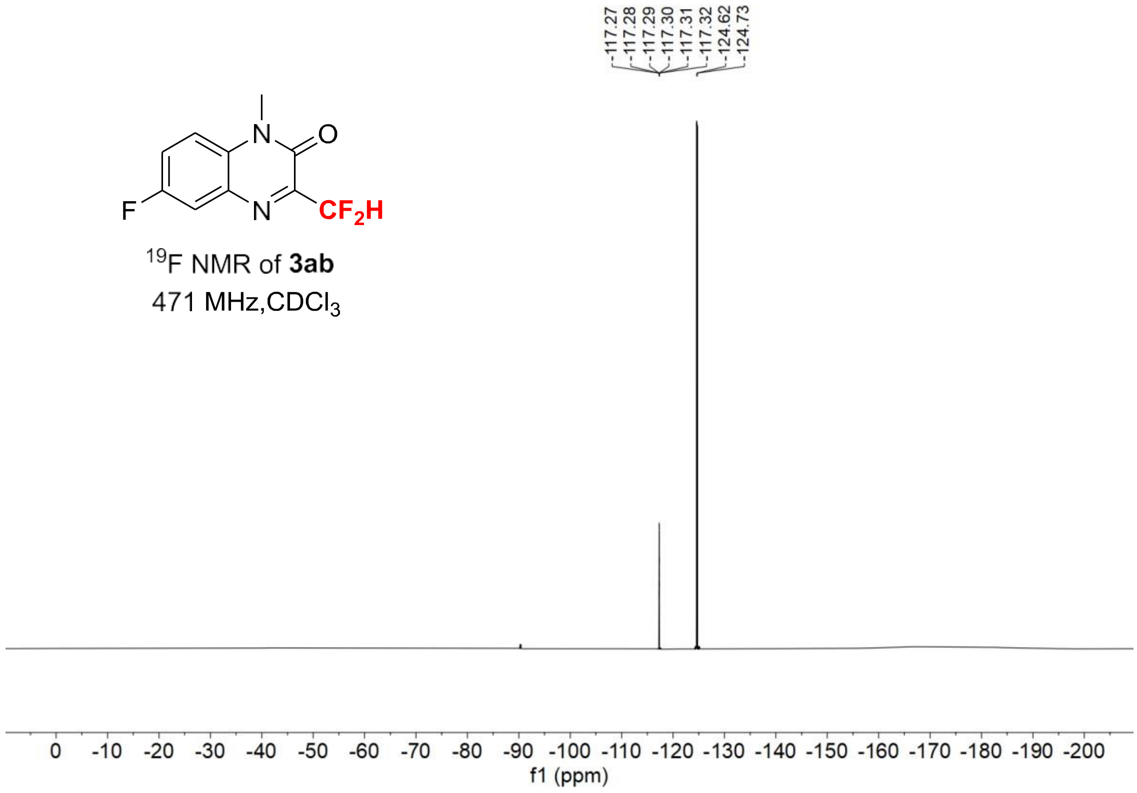

6-chloro-3-(difluoromethyl)-1-methylquinoxalin-2(1*H*)-one (3ac)

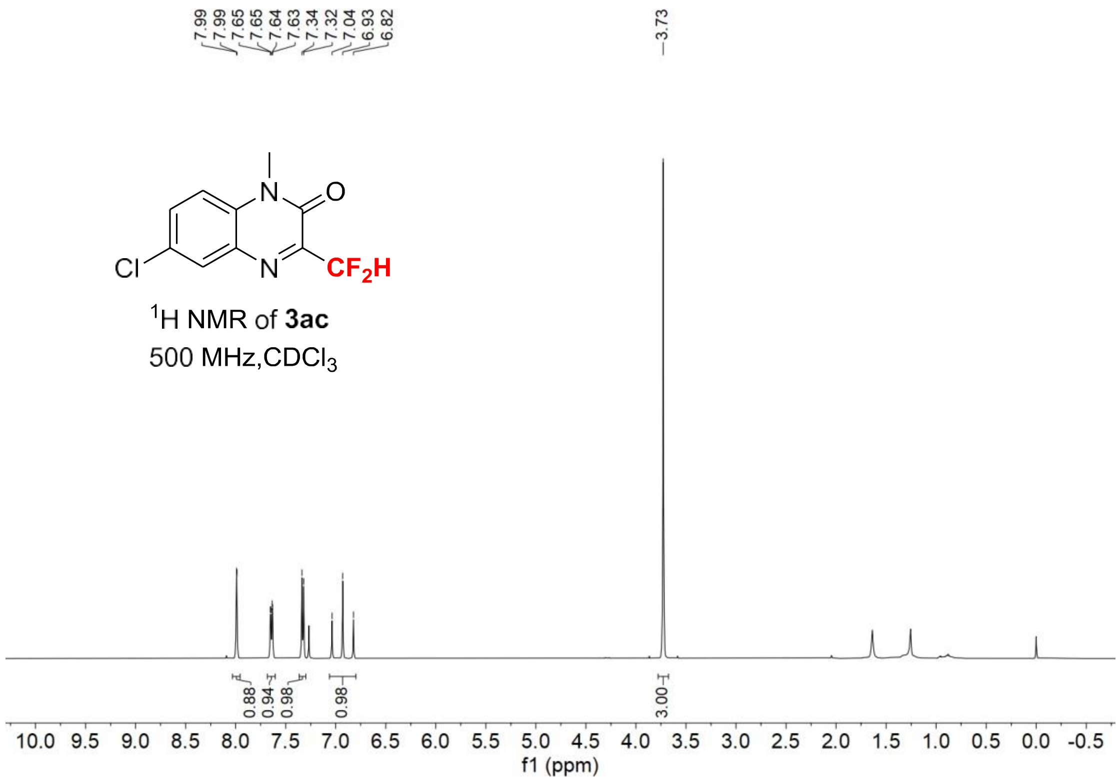
7.99
7.99
7.65
7.65
7.64
7.63
7.34
7.32
7.04
6.93
6.82
3.73
Cl
N
N
O
CF2H
1H NMR of 3ac
500 MHz,CDCl3
0.88
0.94
0.98
0.98
3.00
10.0 9.5 9.0 8.5 8.0 7.5 7.0 6.5 6.0 5.5 5.0 4.5 4.0 3.5 3.0 2.5 2.0 1.5 1.0 0.5 0.0 -0.5
f1 (ppm)

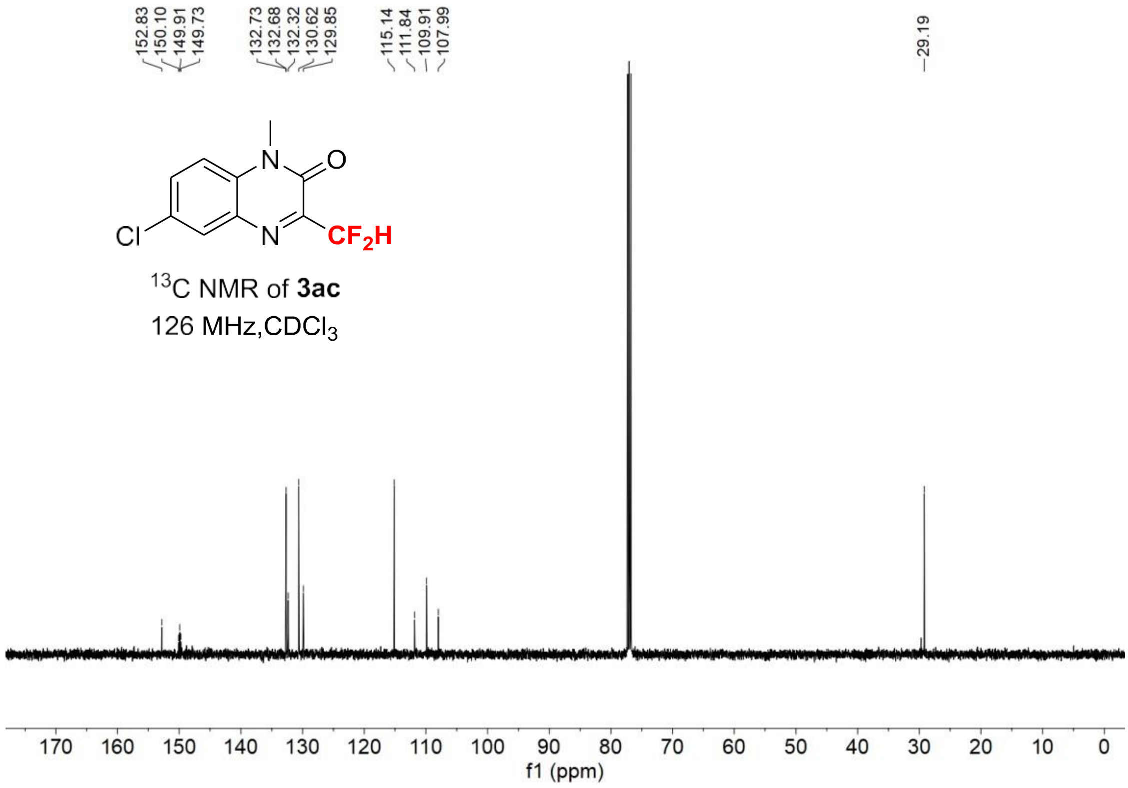
152.83
150.10
149.91
149.73
132.73
132.68
132.32
130.62
129.85
115.14
111.84
109.91
107.99
29.19
Cl
N
N
O
CF2H
13C NMR of 3ac
126 MHz,CDCl3
170 160 150 140 130 120 110 100 90 80 70 60 50 40 30 20 10 0
f1 (ppm)

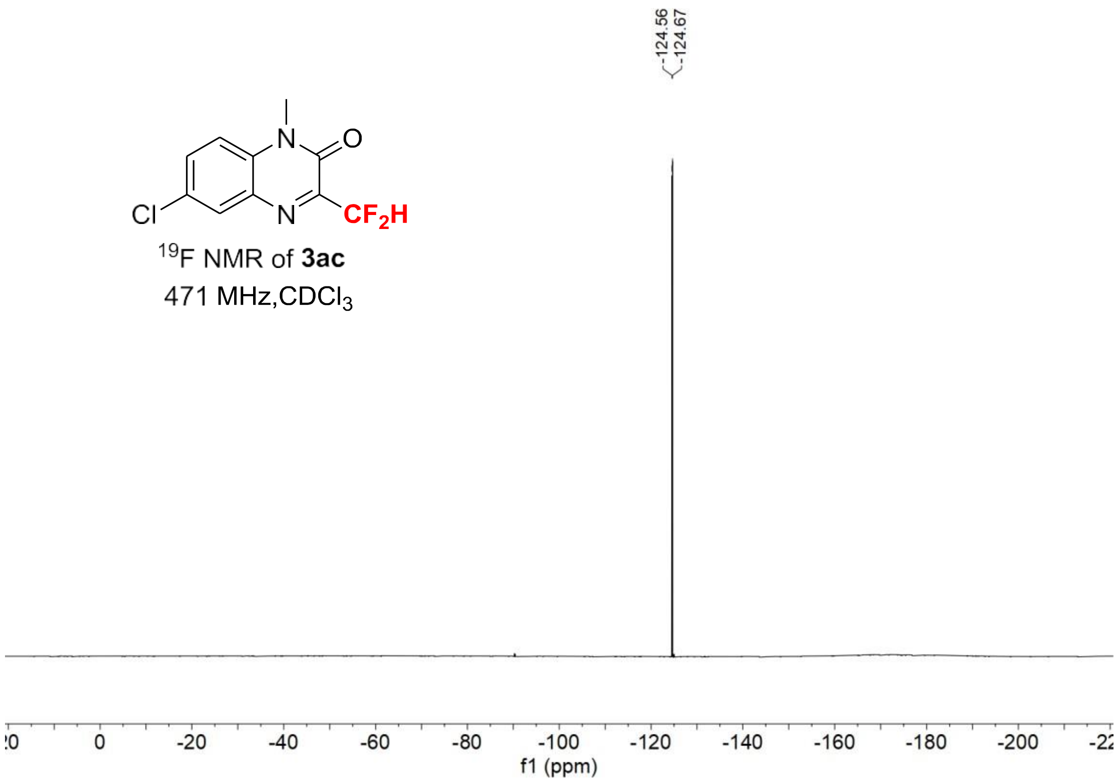

6-bromo-3-(difluoromethyl)-1-methylquinoxalin-2(1*H*)-one (3ad)

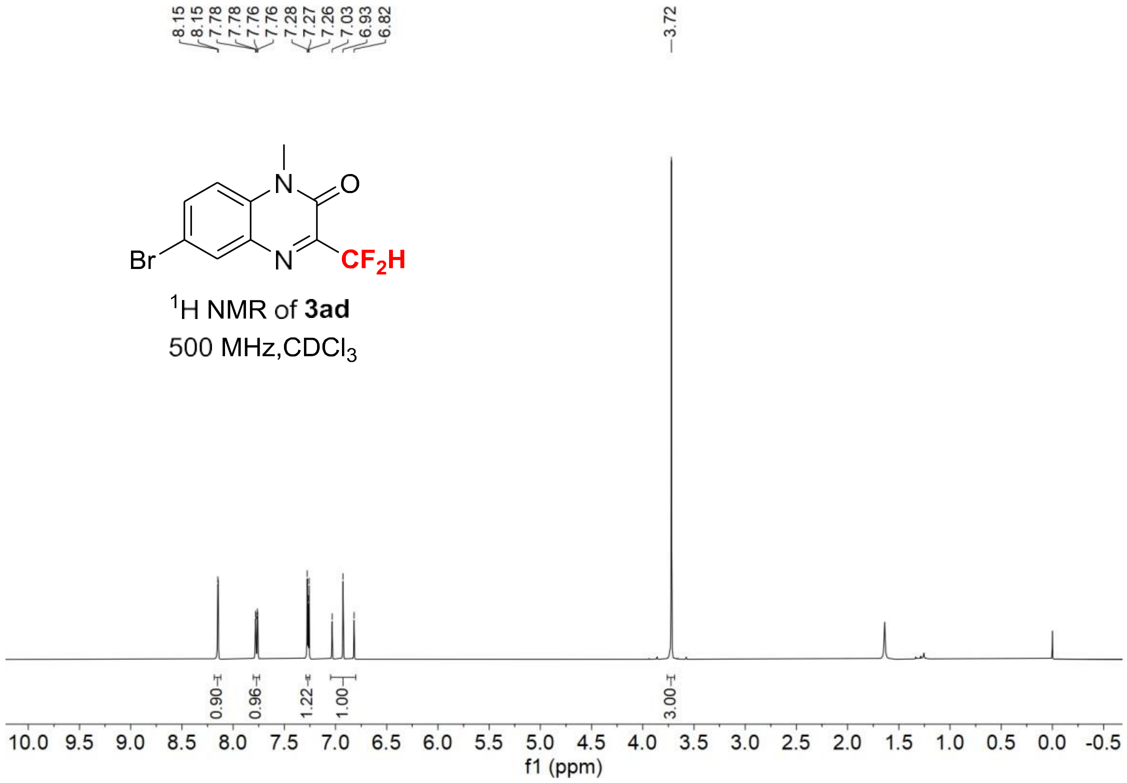

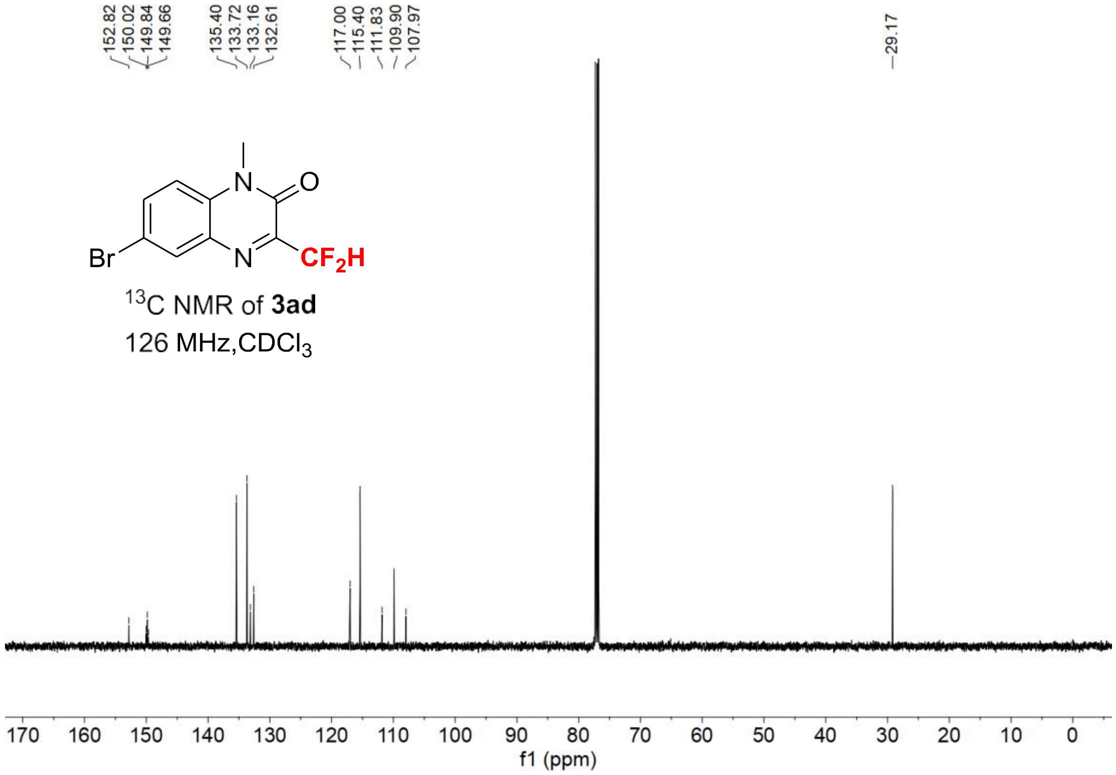

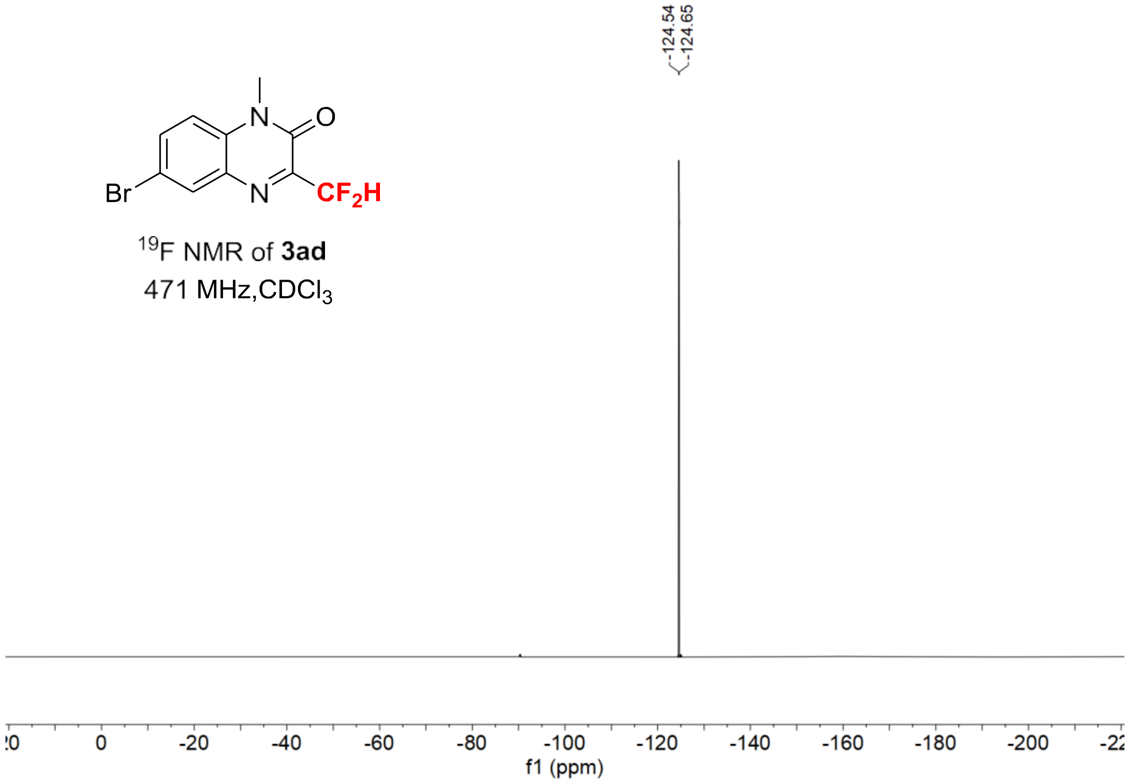

6-nitro-3-(difluoromethyl)-1-methylquinoxalin-2(1*H*)-one (3ae)

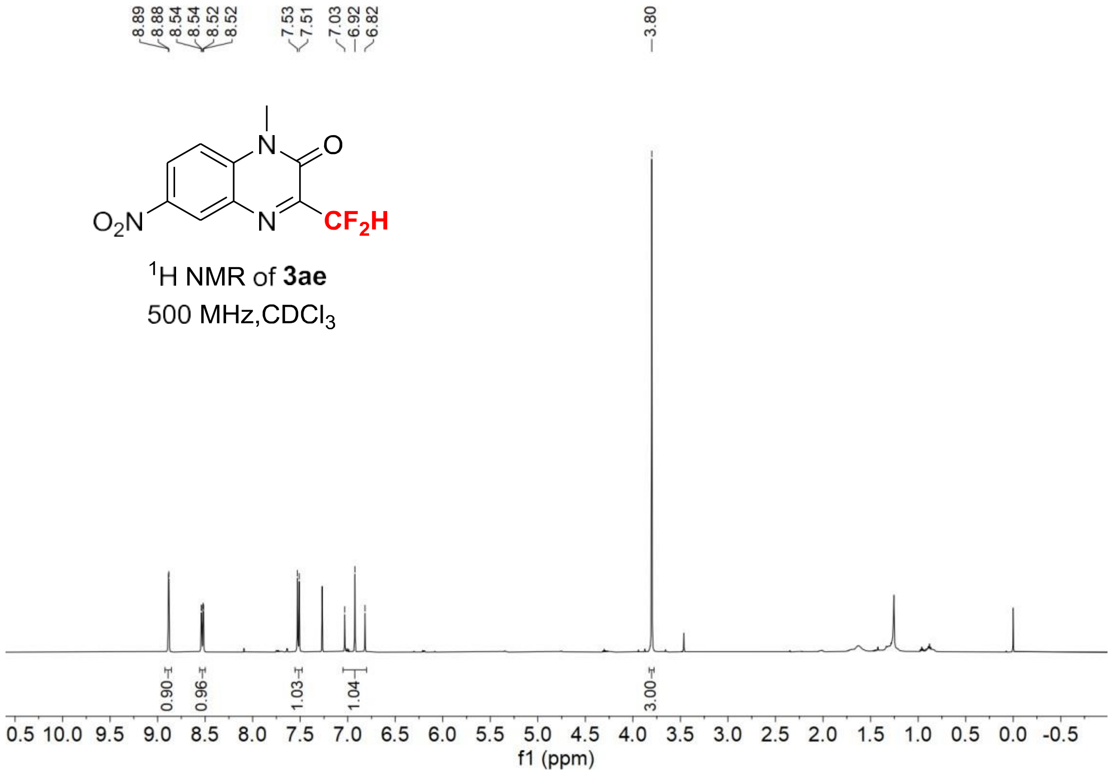
8.89
8.88
8.54
8.54
8.52
8.52
7.53
7.51
7.03
6.92
6.82
3.80
O2N
N
O
N
CF2H
1H NMR of 3ae
500 MHz,CDCl3
0.90
0.96
1.03
1.04
3.00
f1 (ppm)

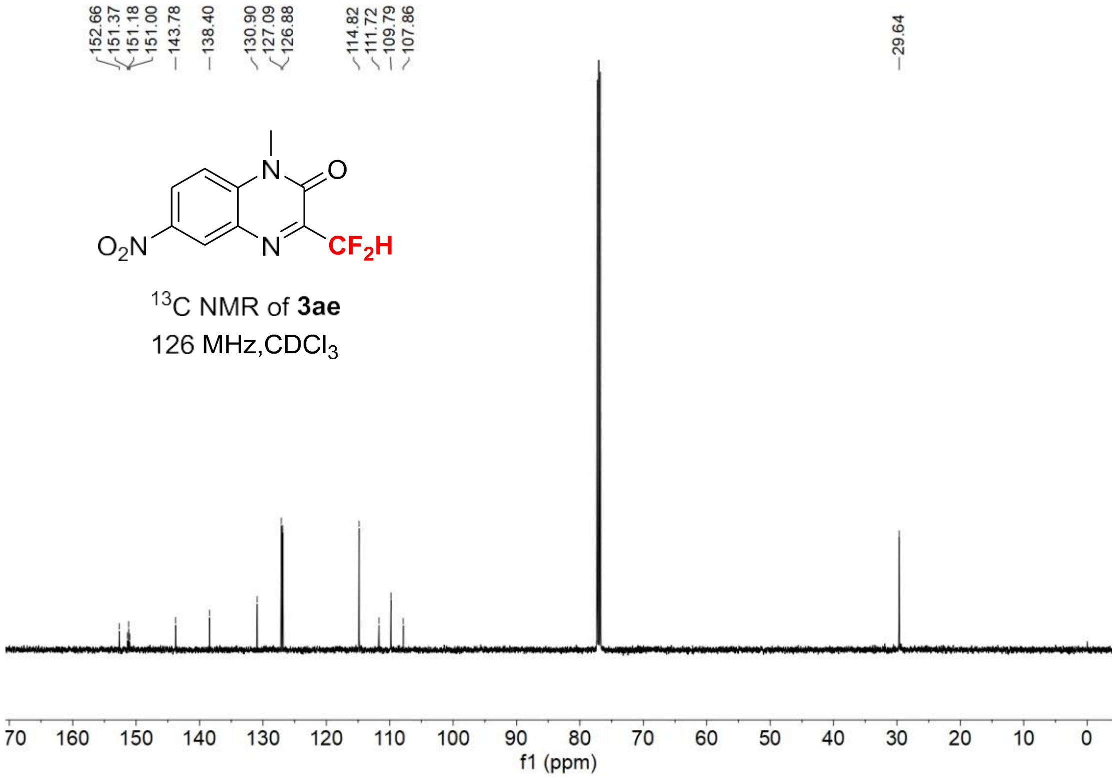
152.66
151.37
151.18
151.00
143.78
138.40
130.90
127.09
126.88
114.82
111.72
109.79
107.86
29.64
O2N
N
O
N
CF2H
13C NMR of 3ae
126 MHz,CDCl3
f1 (ppm)

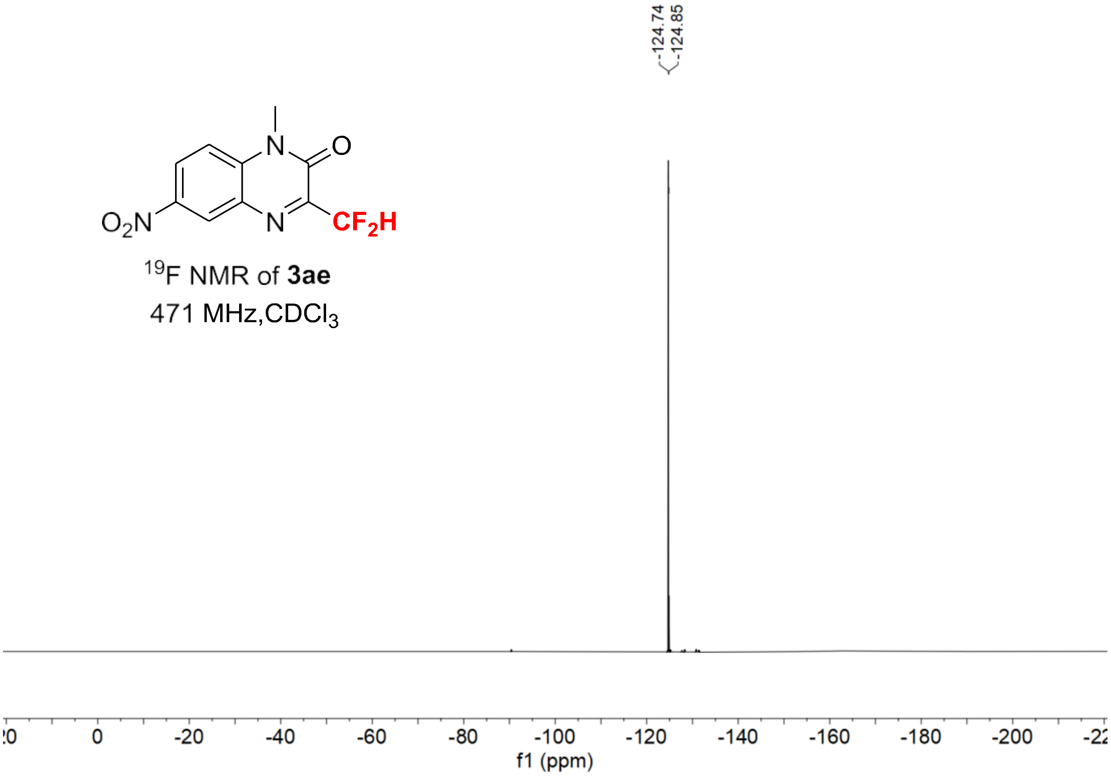

6-trifluoromethyl-3-(difluoromethyl)-1-methylquinoxalin-2(1*H*)-one (3af)

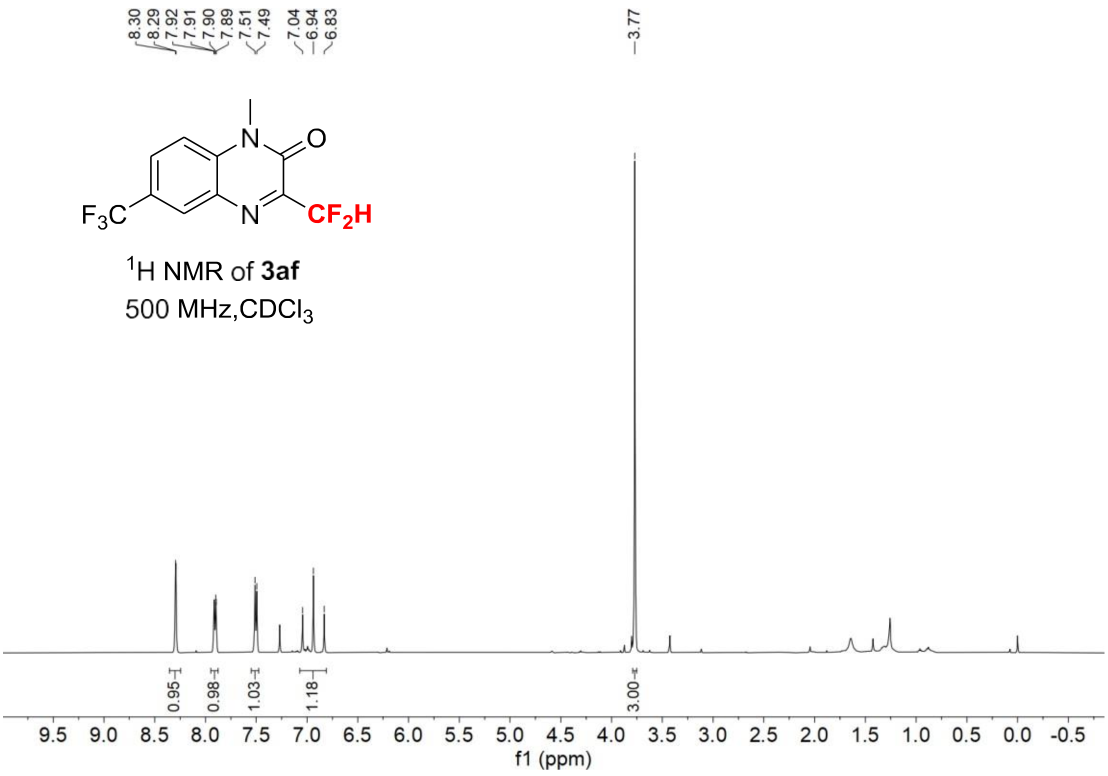

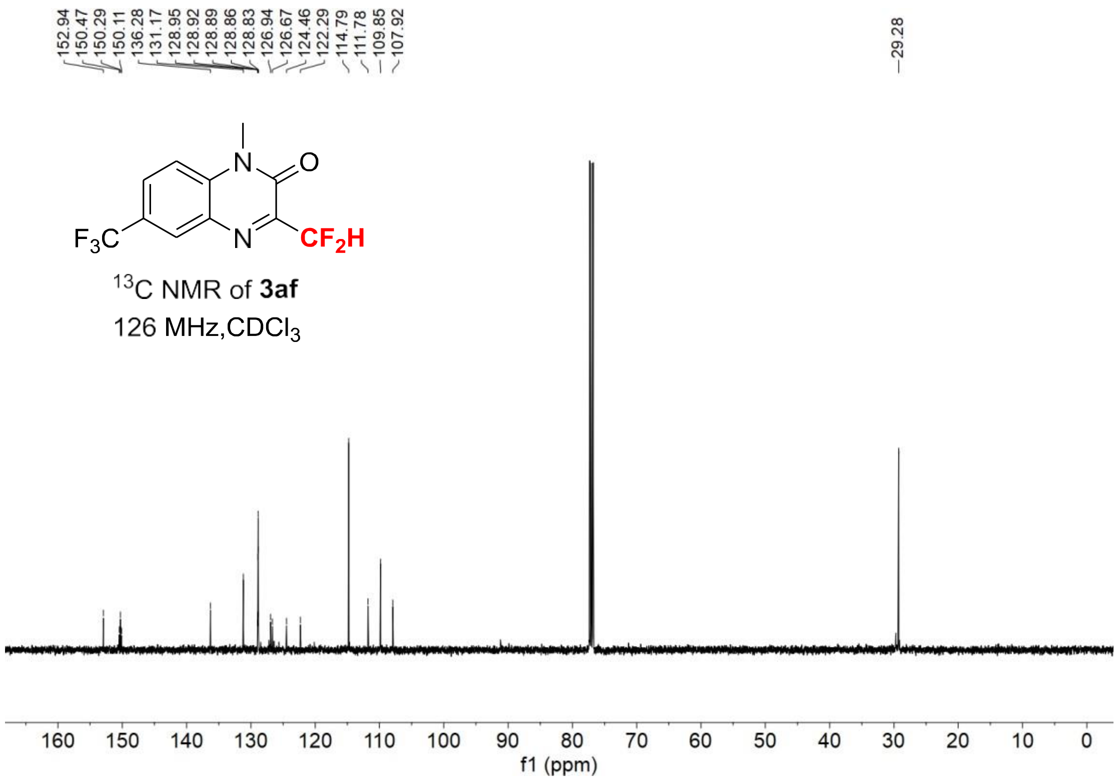

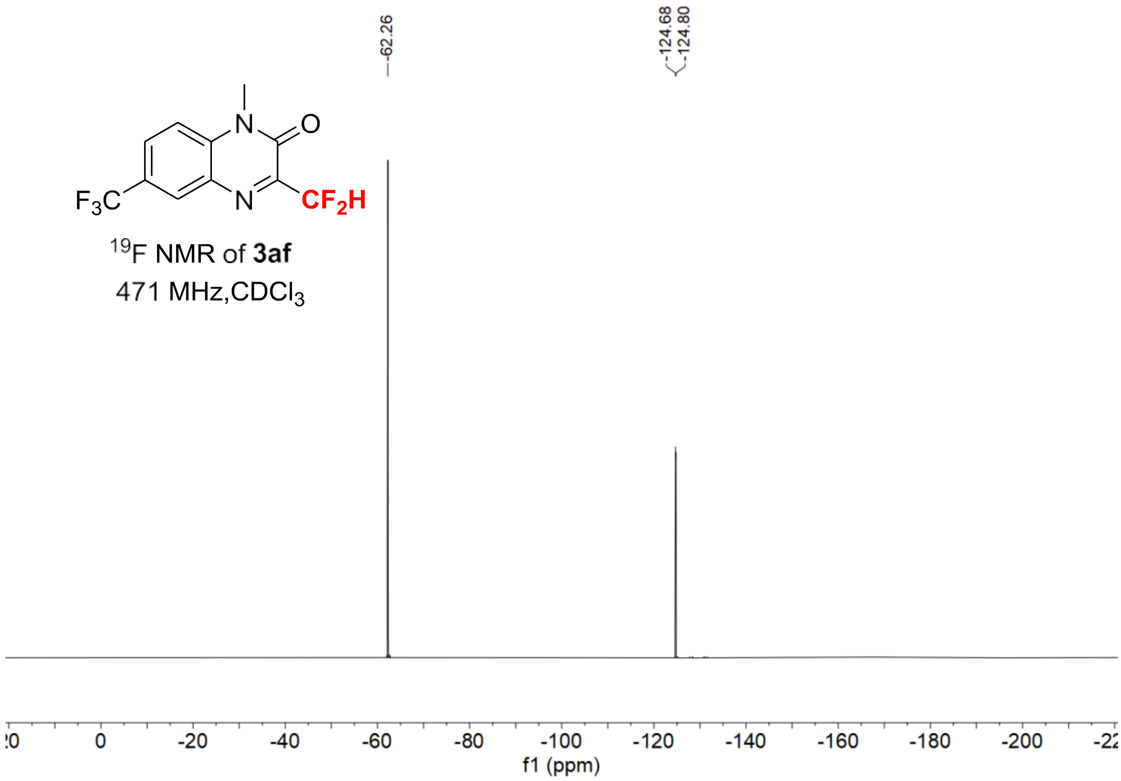

1-methyl-2-oxo-3-(difluoromethyl)-1,2-dihydroquinoxaline-6-carbonitrile (3ag)

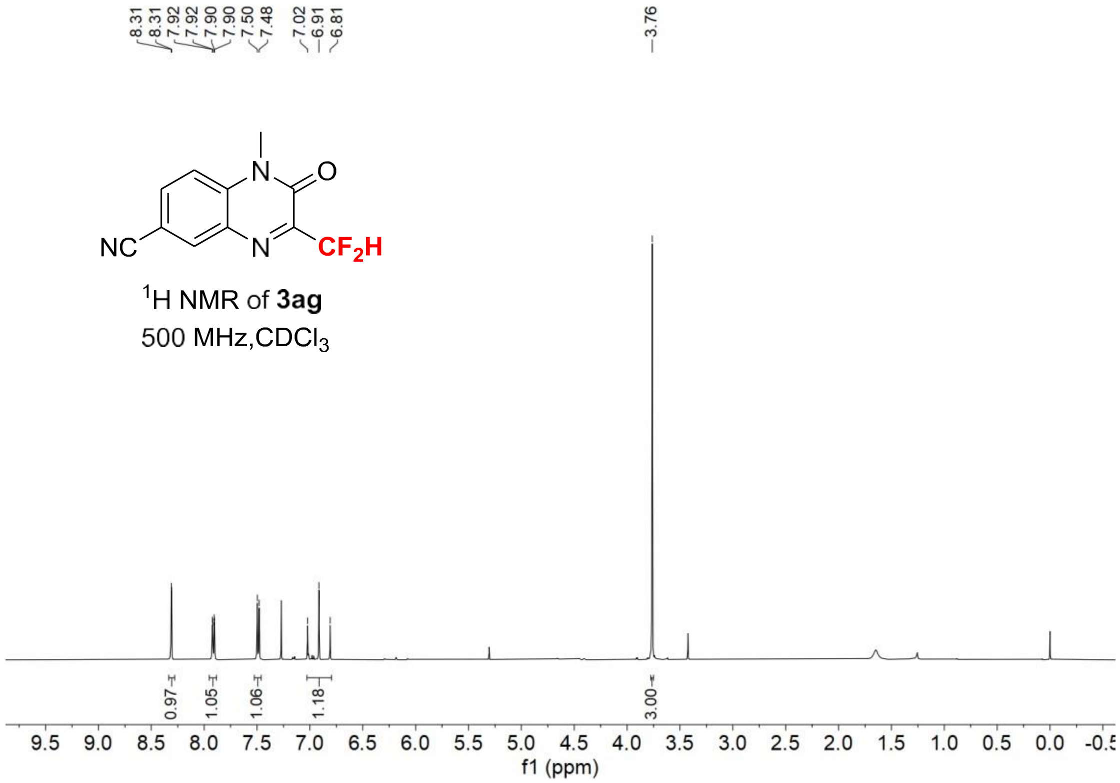
8.31
8.31
7.92
7.92
7.90
7.90
7.50
7.48
7.02
6.91
6.81
3.76
N
O
NC
N
CF2H
1H NMR of 3ag
500 MHz,CDCl3
0.97
1.05
1.06
1.18
3.00
f1 (ppm)

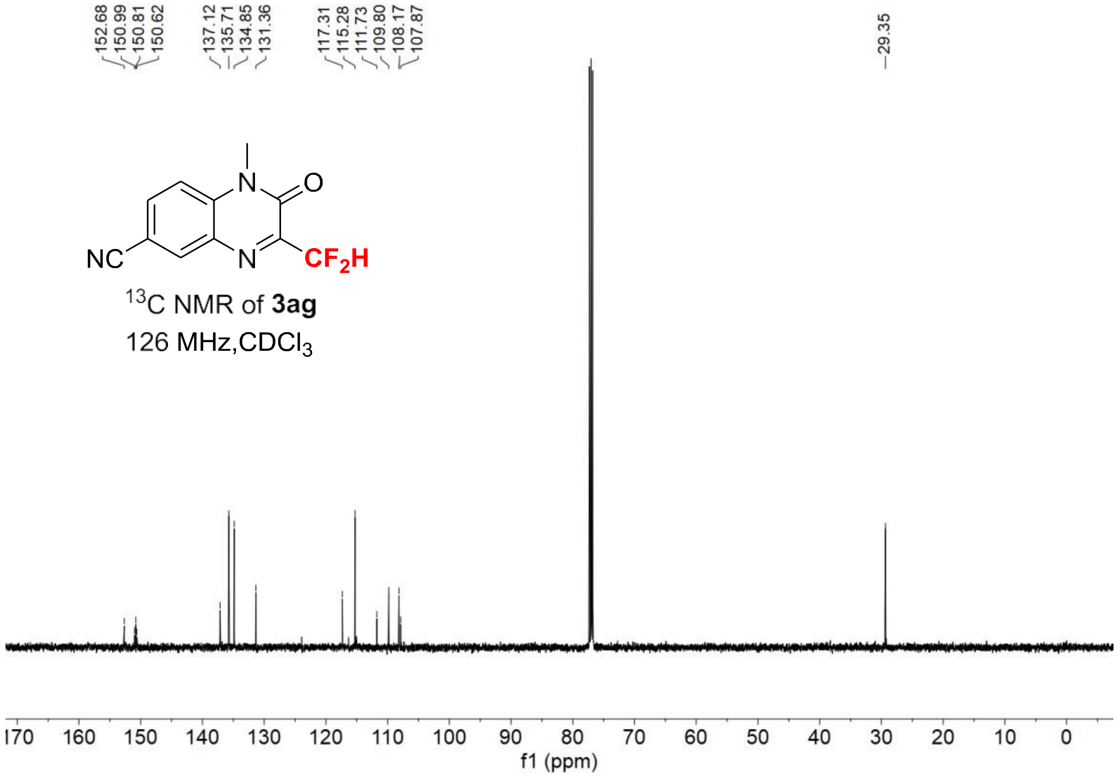
152.68
150.99
150.81
150.62
137.12
135.71
134.85
131.36
117.31
115.28
111.73
109.80
108.17
107.87
29.35
N
O
NC
N
CF2H
13C NMR of 3ag
126 MHz,CDCl3
f1 (ppm)

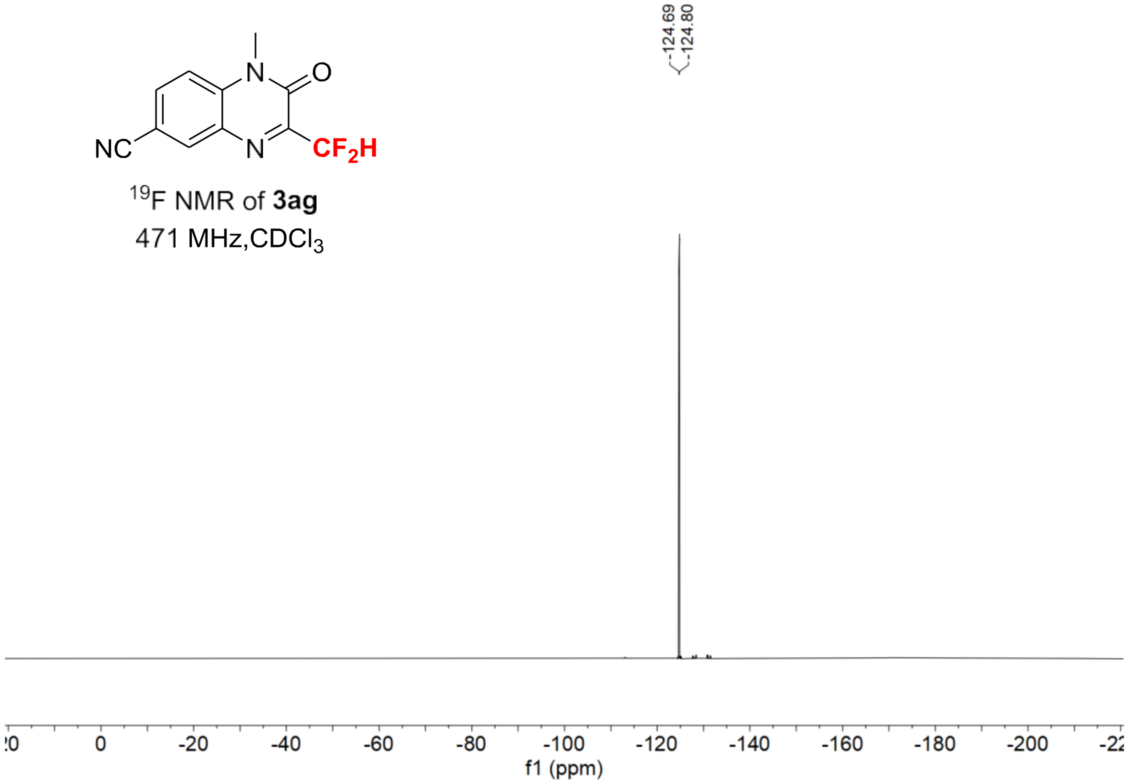

Methyl-3-(difluoromethyl)-1-methyl-2-oxo-1,2- dihydroquinoxaline-6-carboxylate (3ah)

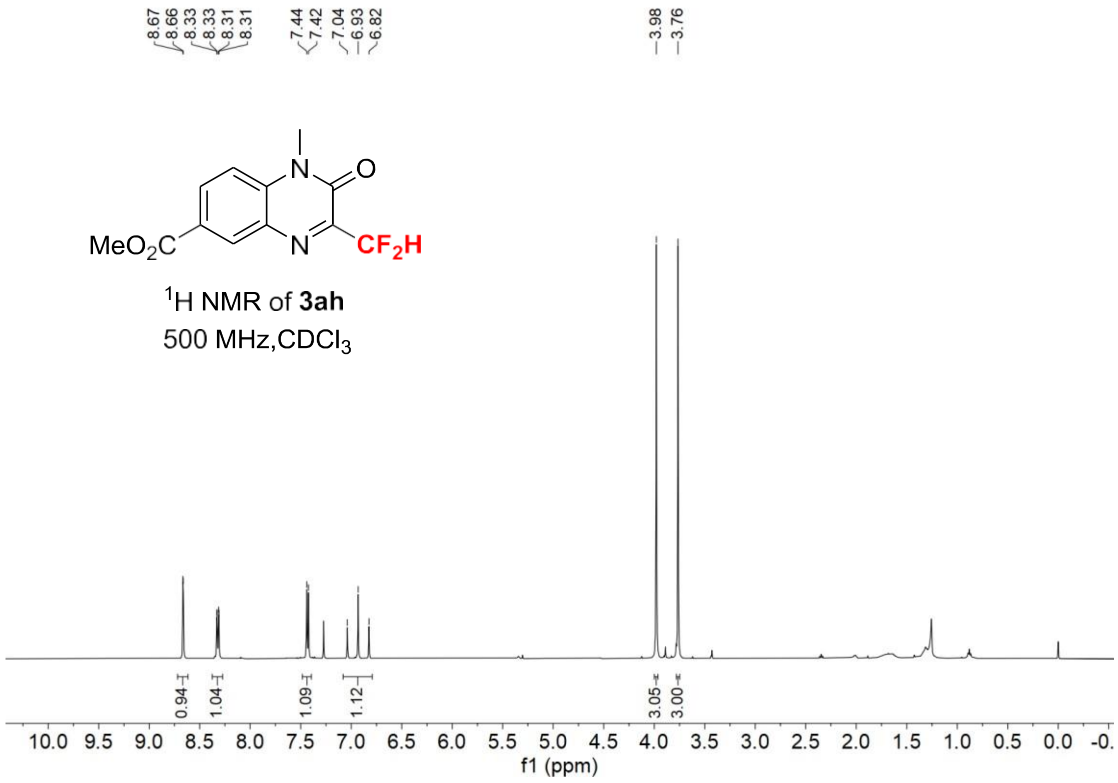

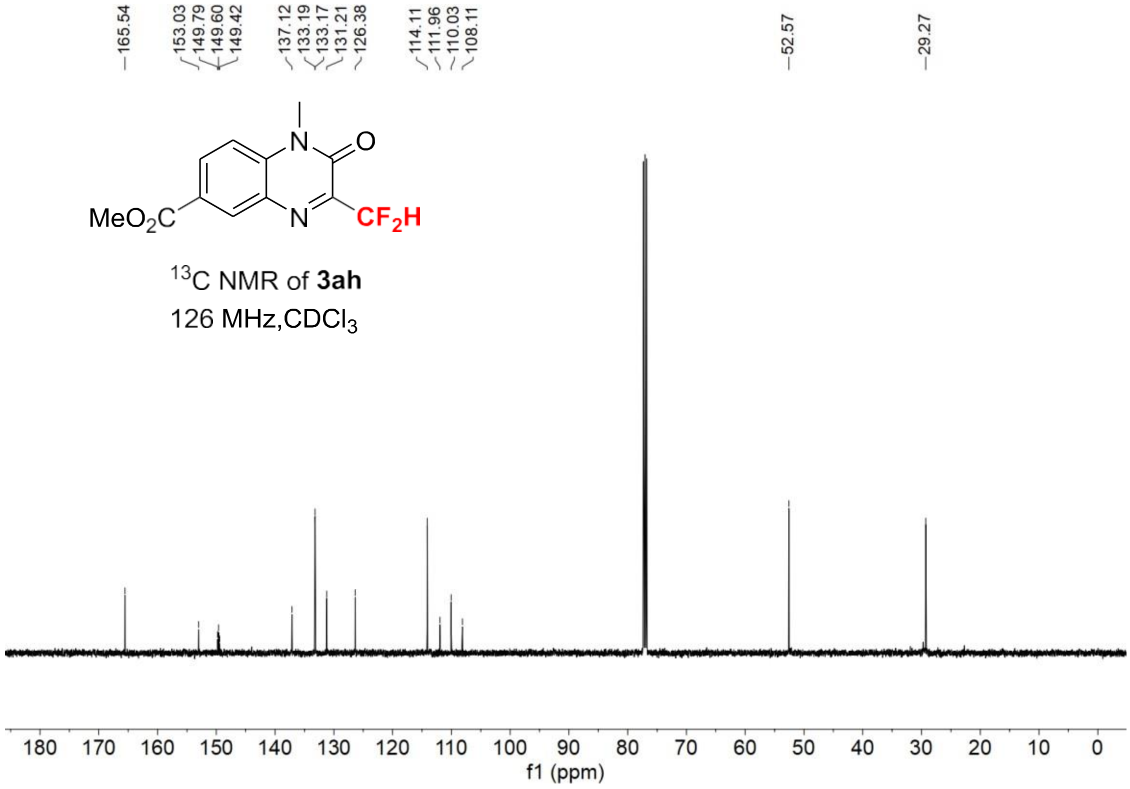

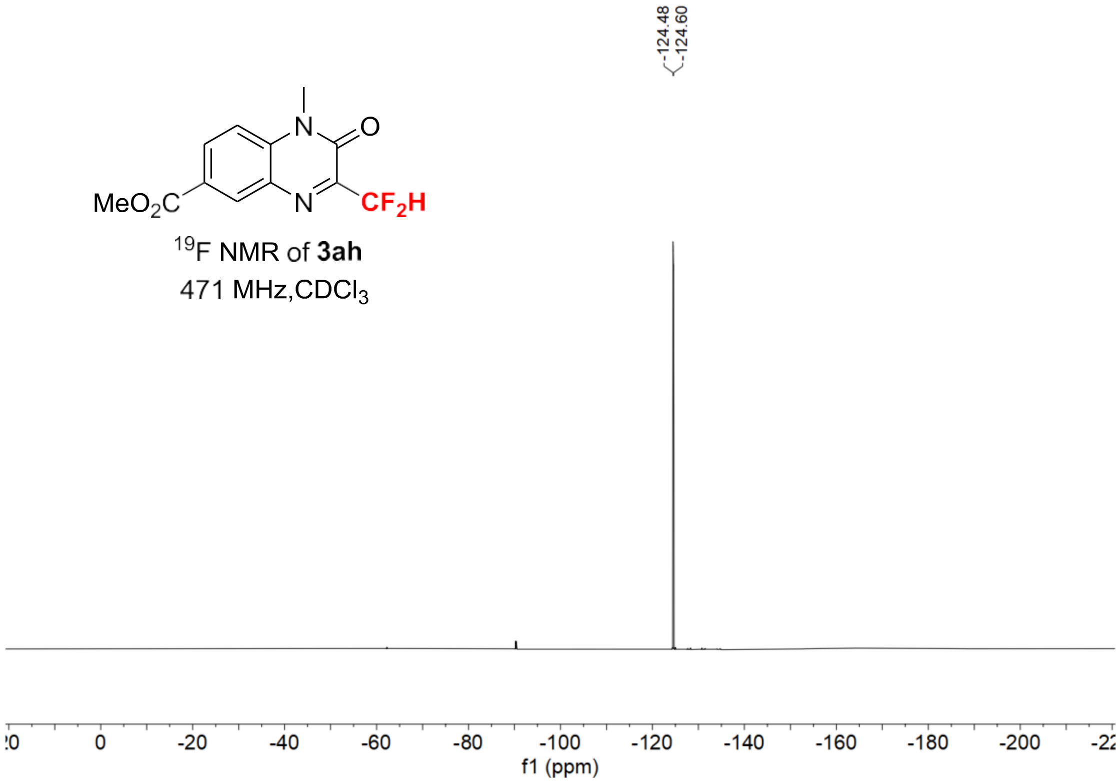

6-(tert-butyl)-3-(difluoromethyl)-1-methylquinoxalin-2(1*H*)-one (3ai)

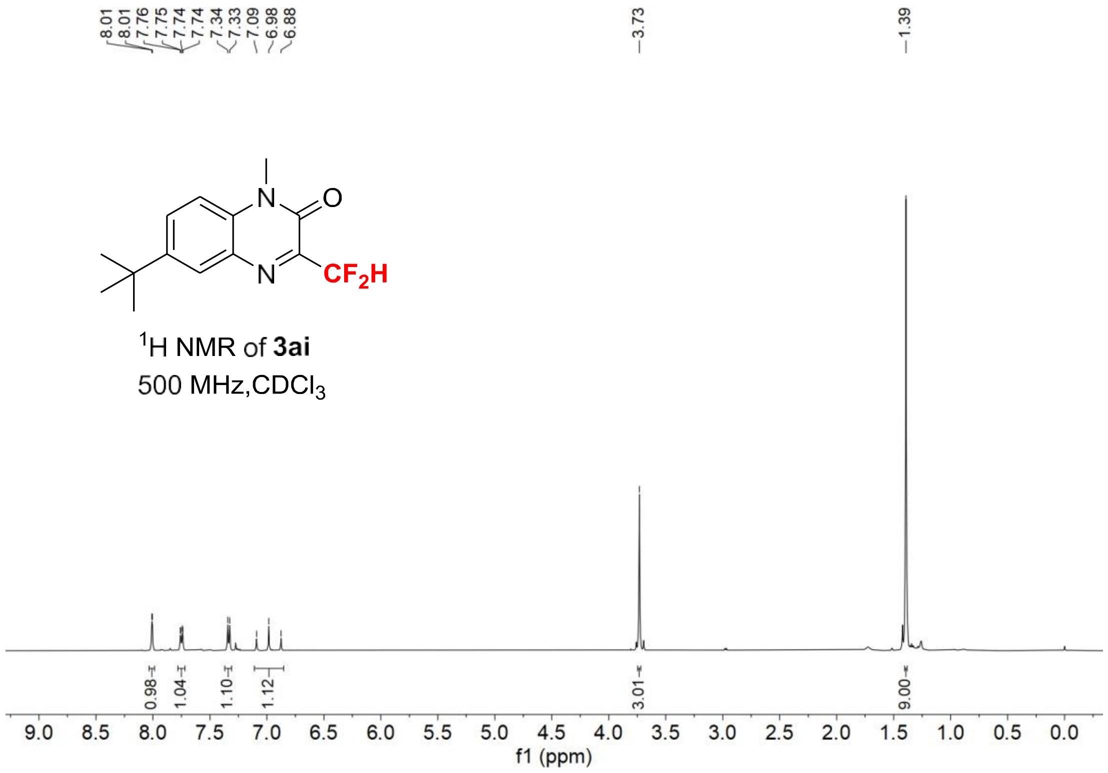
8.01
8.01
7.76
7.75
7.74
7.74
7.34
7.33
7.09
6.98
6.88
3.73
1.39
CF2H
1H NMR of 3ai
500 MHz,CDCl3
0.98
1.04
1.10
1.12
3.01
9.00
9.0 8.5 8.0 7.5 7.0 6.5 6.0 5.5 5.0 4.5 4.0 3.5 3.0 2.5 2.0 1.5 1.0 0.5 0.0
f1 (ppm)

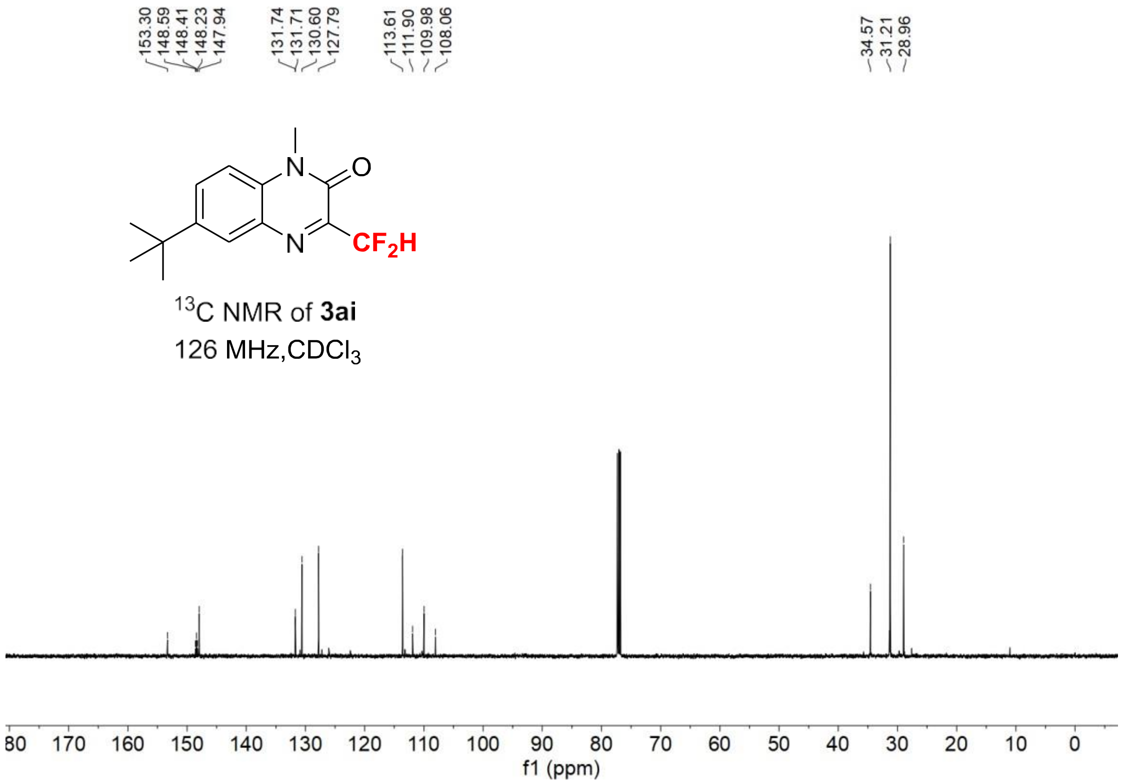
153.30
148.59
148.41
148.23
147.94
131.74
131.71
130.60
127.79
113.61
111.90
109.98
108.06
34.57
31.21
28.96
CF2H
13C NMR of 3ai
126 MHz,CDCl3
80 170 160 150 140 130 120 110 100 90 80 70 60 50 40 30 20 10 0
f1 (ppm)

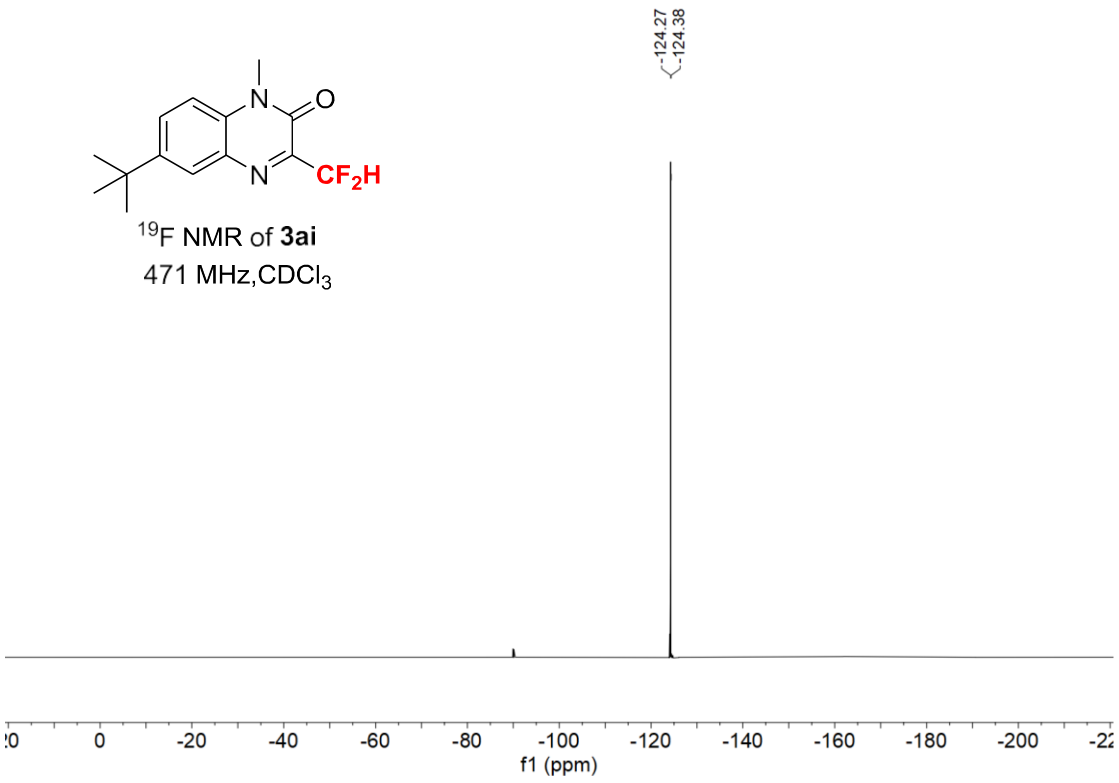

7-fluoro-3-(difluoromethyl)-1-methylquinoxalin-2(1*H*)-one (3aj)

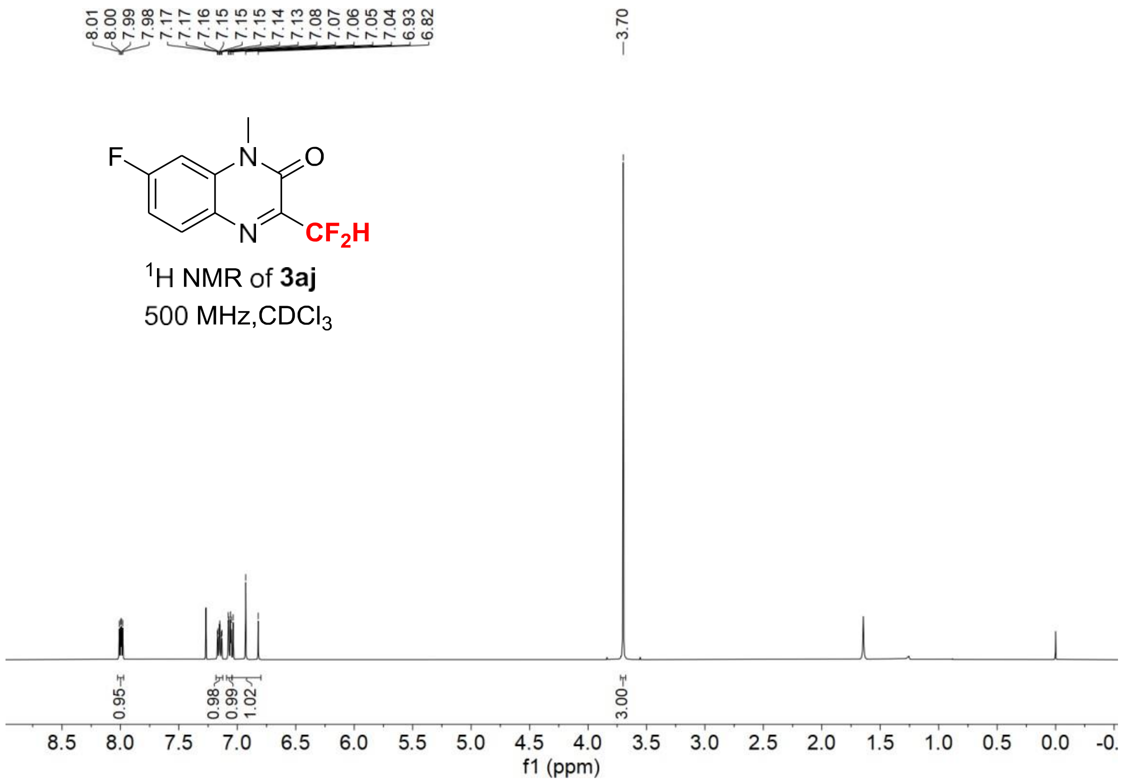

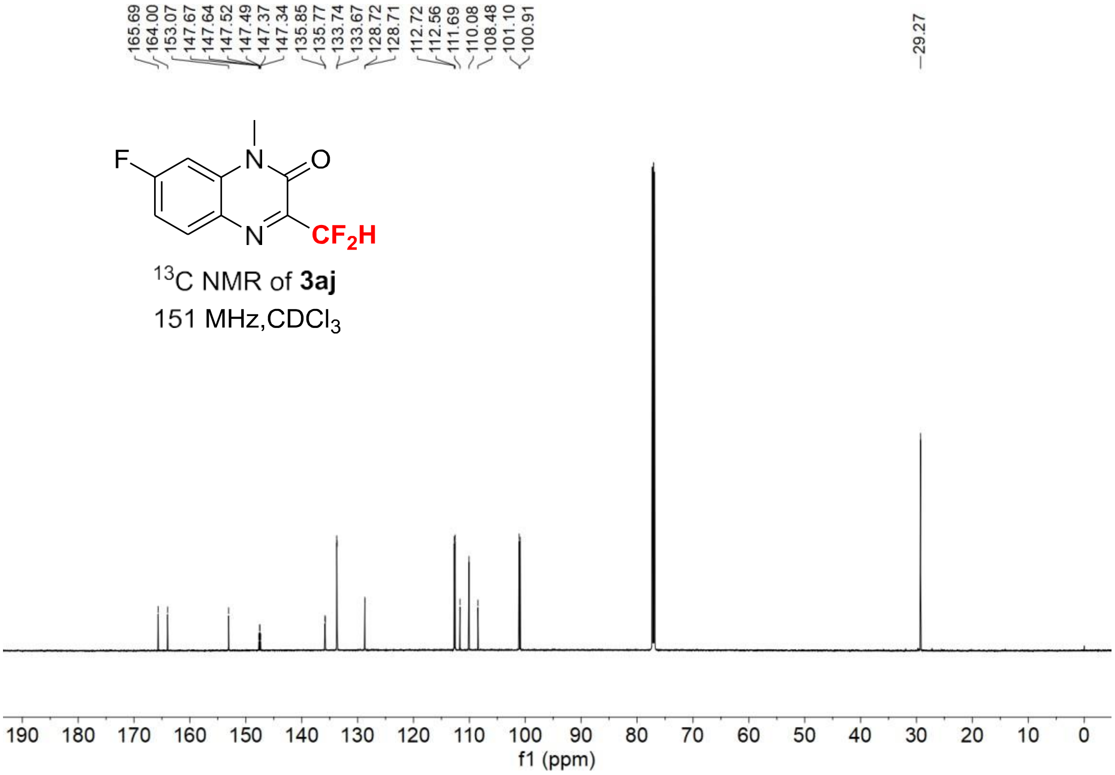

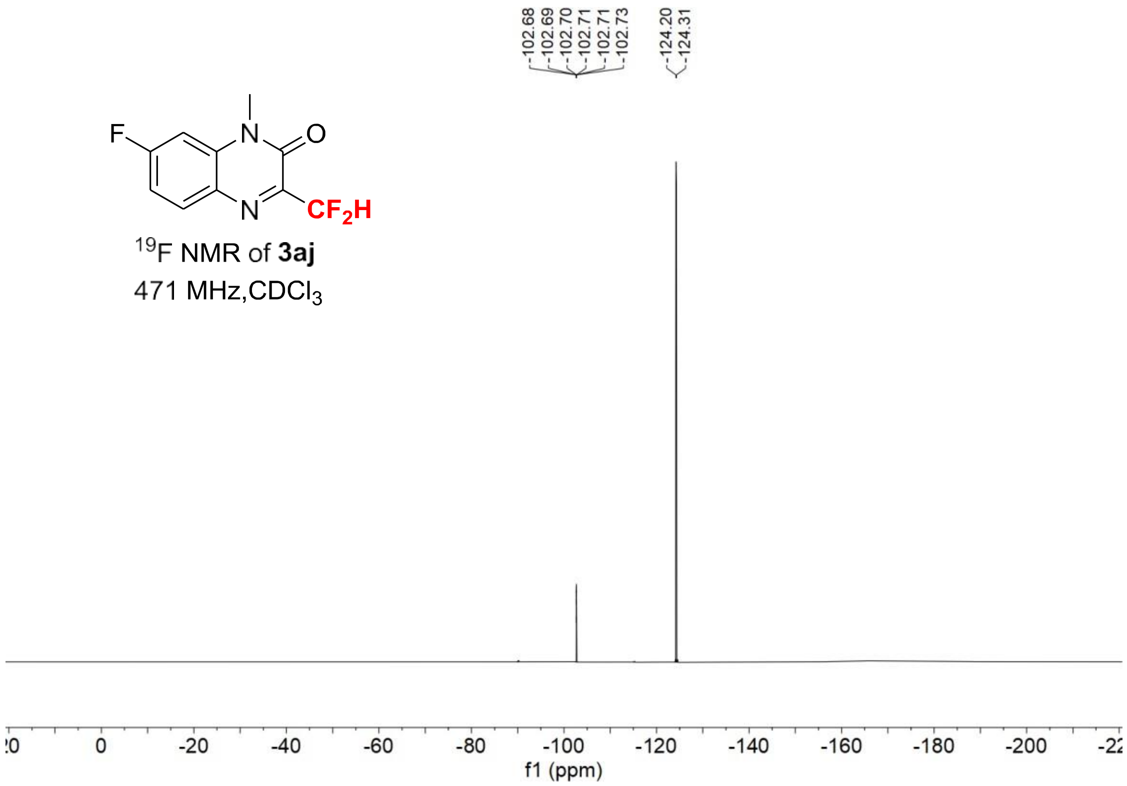

7-chloro-3-(difluoromethyl)-1-methylquinoxalin-2(1*H*)-one (3ak)

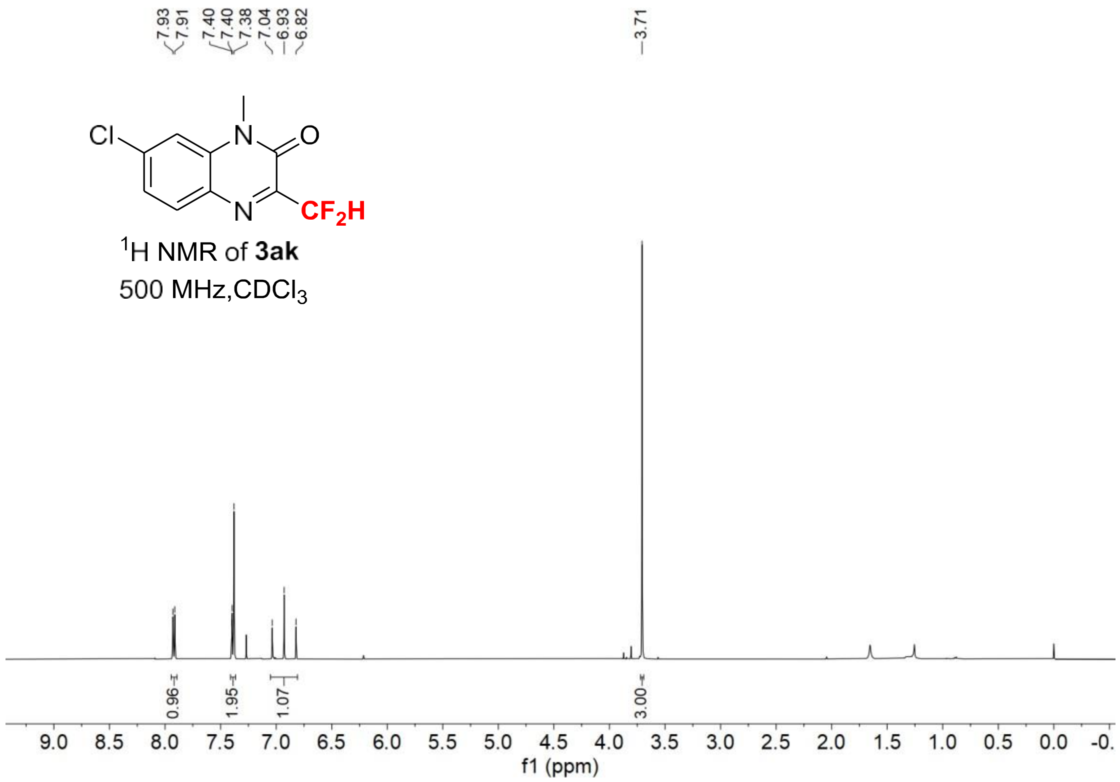
7.93
7.91
7.40
7.40
7.38
7.04
6.93
6.82
3.71
Cl
N
O
N
CF2H
1H NMR of 3ak
500 MHz,CDCl3
0.96
1.95
1.07
3.00
f1 (ppm)

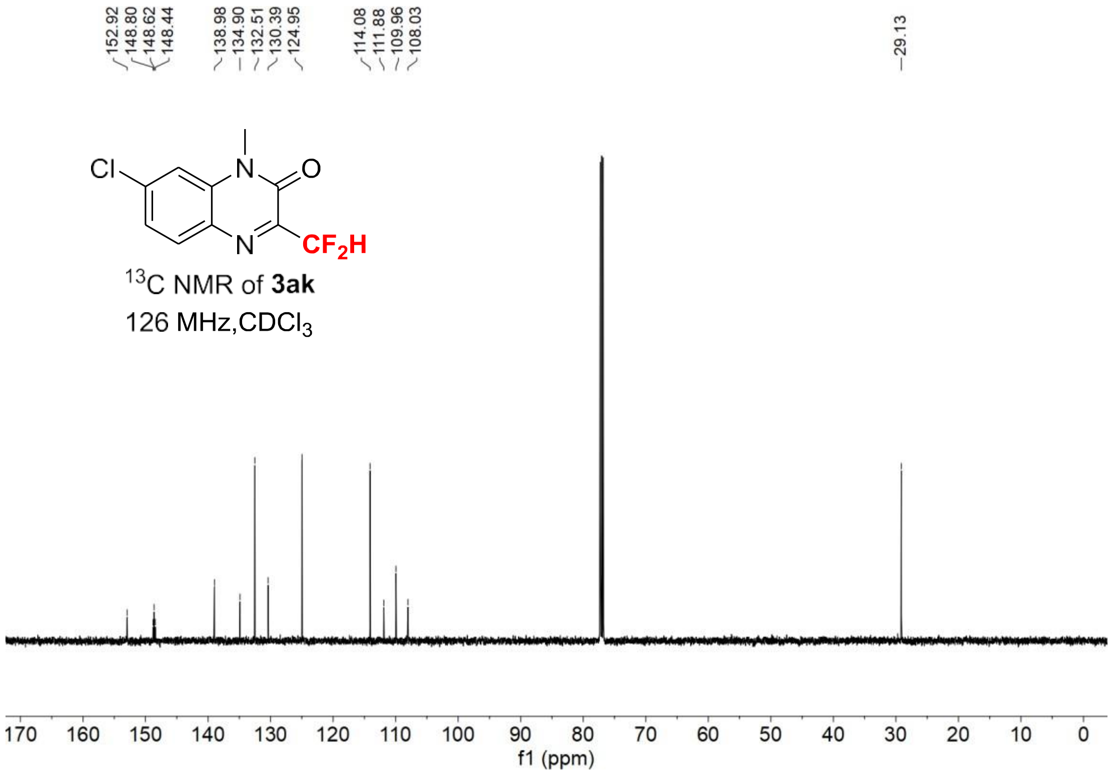
152.92
148.80
148.62
148.44
138.98
134.90
132.51
130.39
124.95
114.08
111.88
109.96
108.03
29.13
Cl
N
O
N
CF2H
13C NMR of 3ak
126 MHz,CDCl3
f1 (ppm)

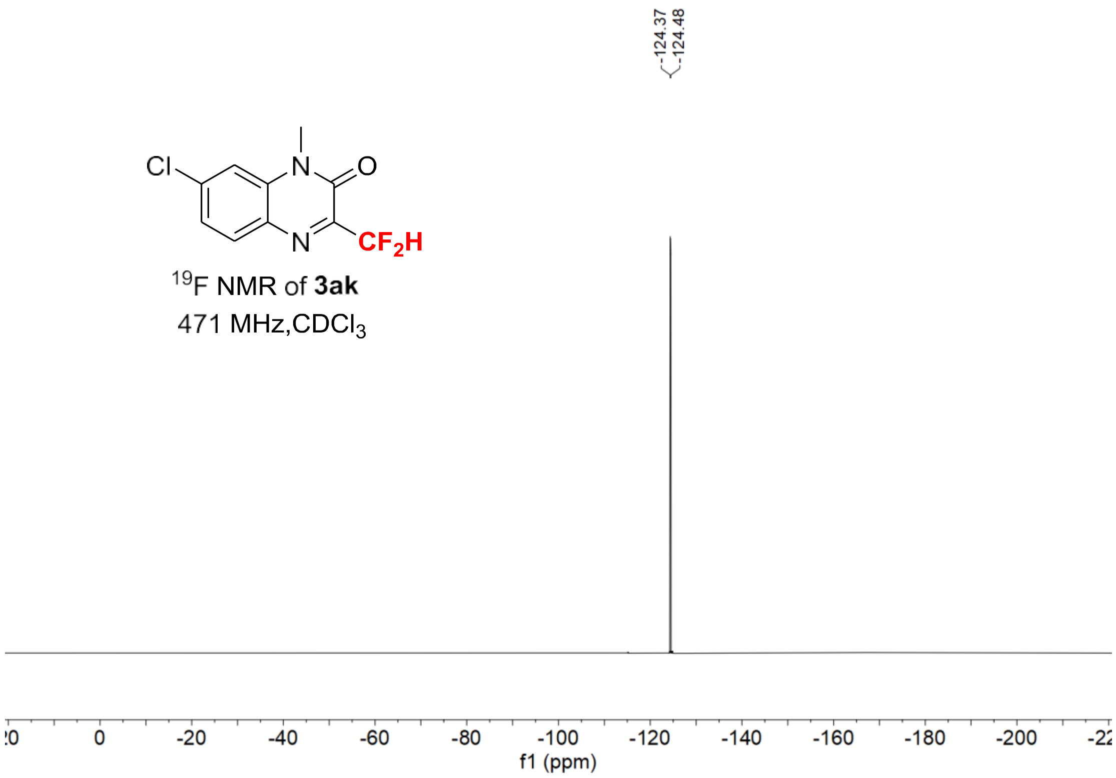

7-bromo-3-(difluoromethyl)-1-methylquinoxalin-2(1*H*)-one (3al)

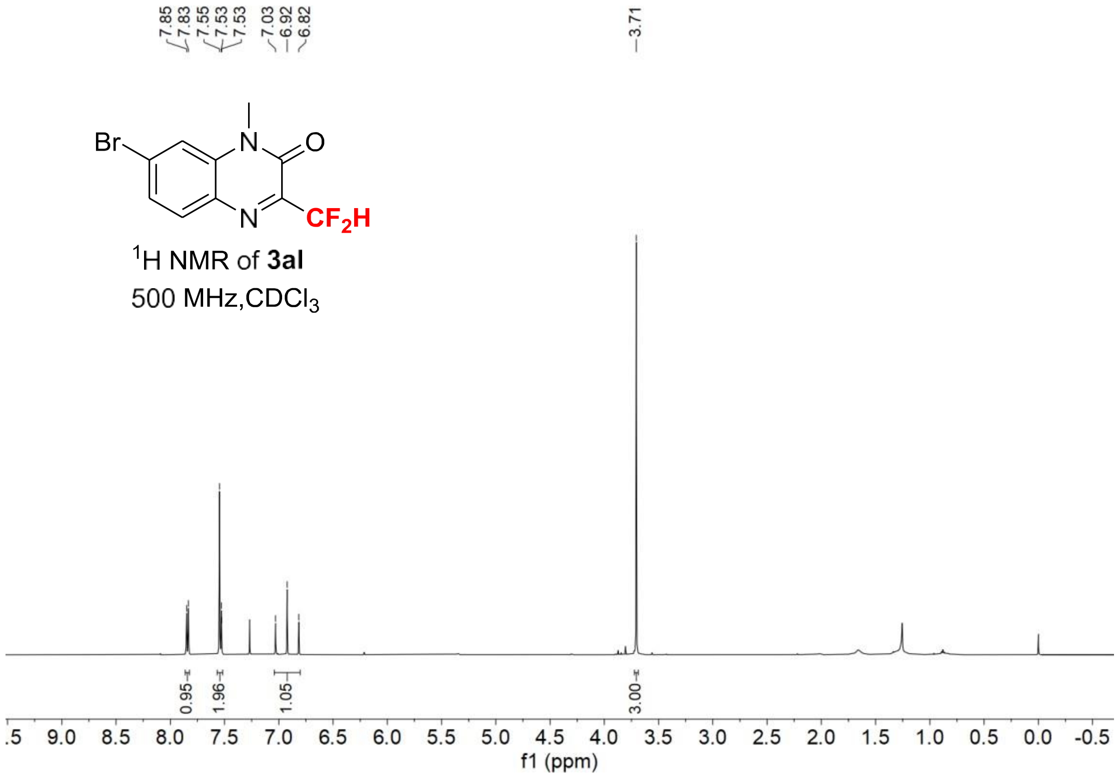

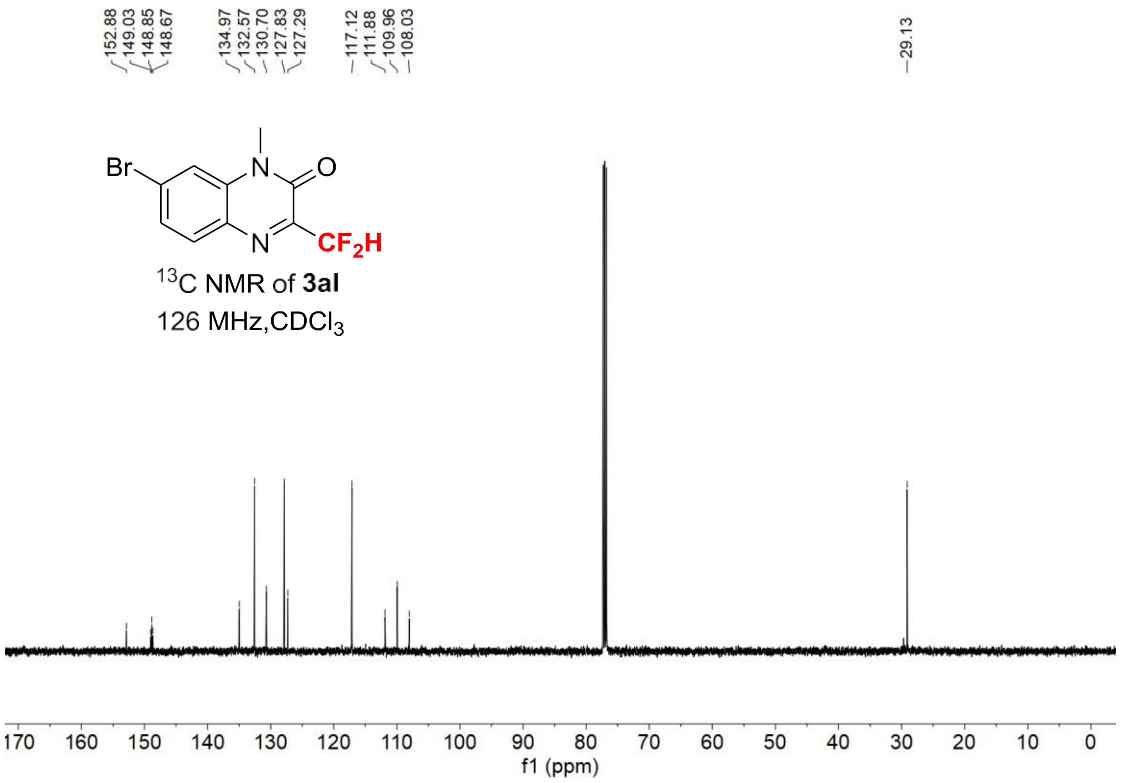

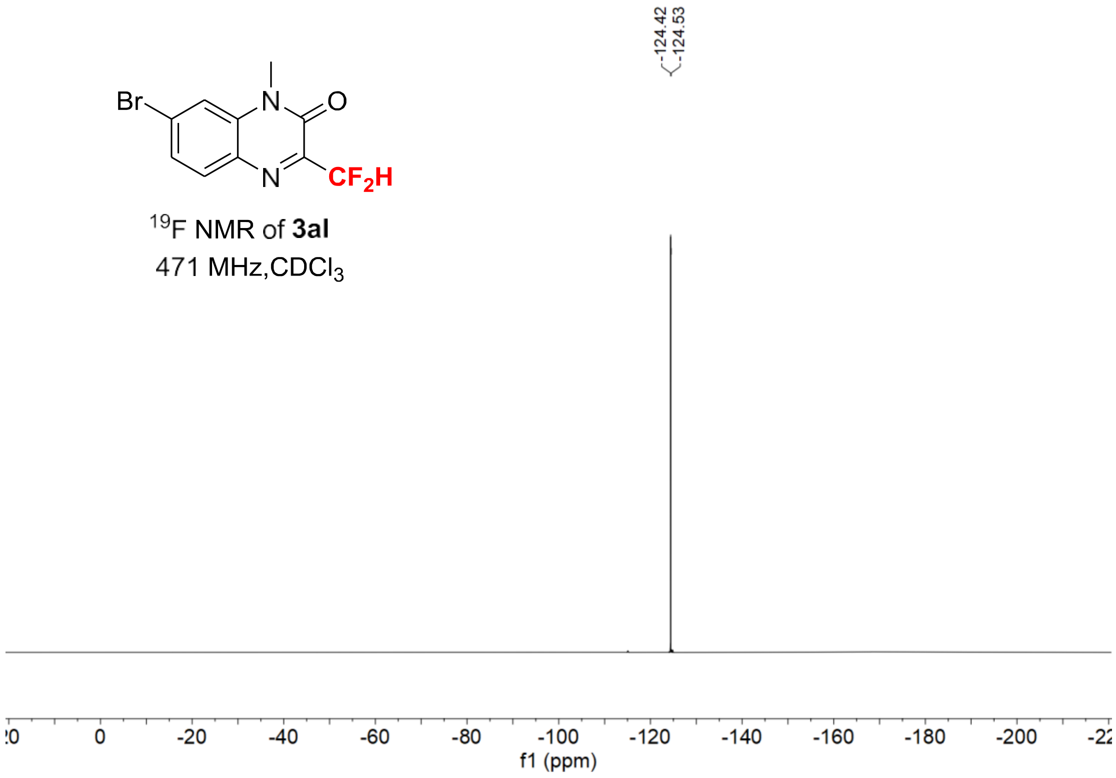

1-methyl-2-oxo-3-(difluoromethyl)-1,2-dihydroquinoxaline-7-carbonitrile (3am)

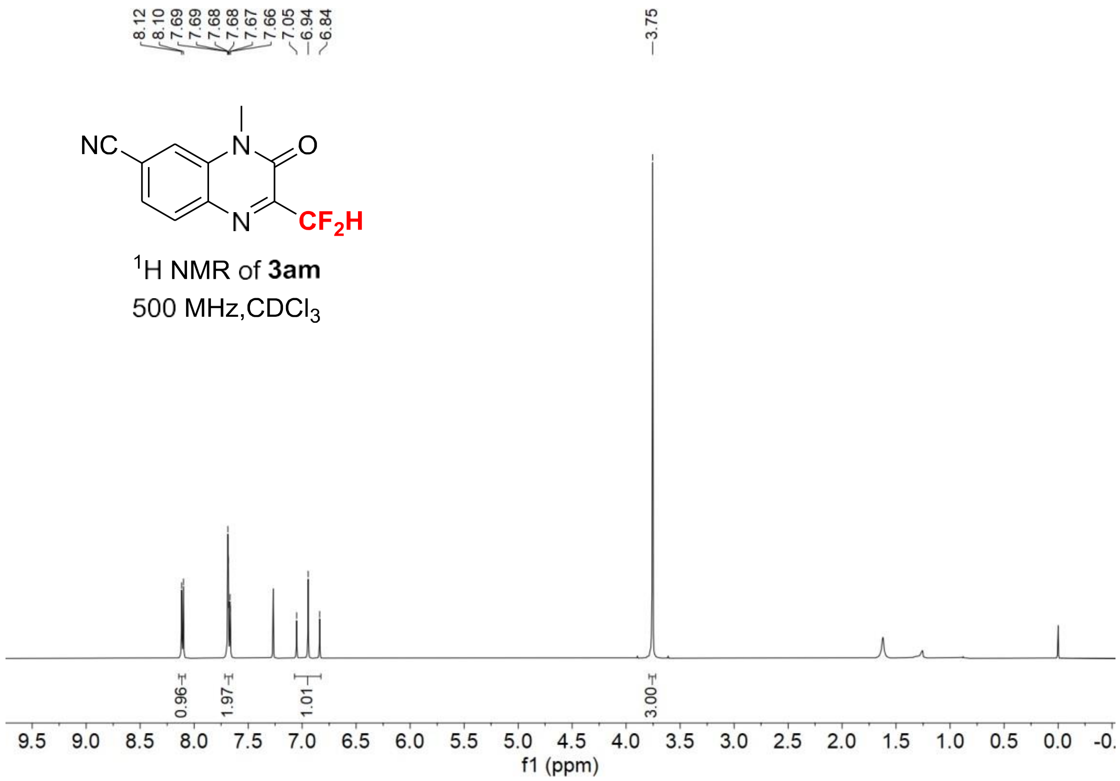
8.12
8.10
7.69
7.69
7.68
7.68
7.67
7.66
7.05
6.94
6.84
3.75
NC
N
O
N
CF2H
1H NMR of 3am
500 MHz,CDCl3
0.96
1.97
1.01
3.00
f1 (ppm)

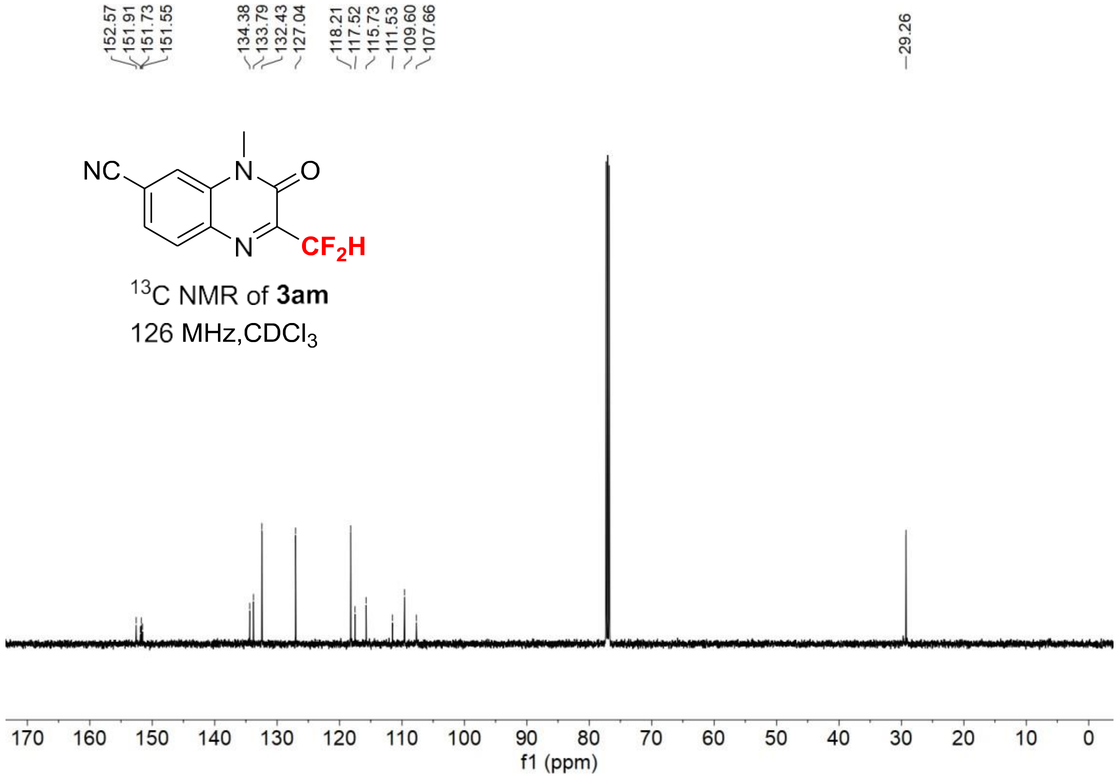
152.57
151.91
151.73
151.55
134.38
133.79
132.43
127.04
118.21
117.52
115.73
111.53
109.60
107.66
29.26
NC
N
O
N
CF2H
13C NMR of 3am
126 MHz,CDCl3
f1 (ppm)

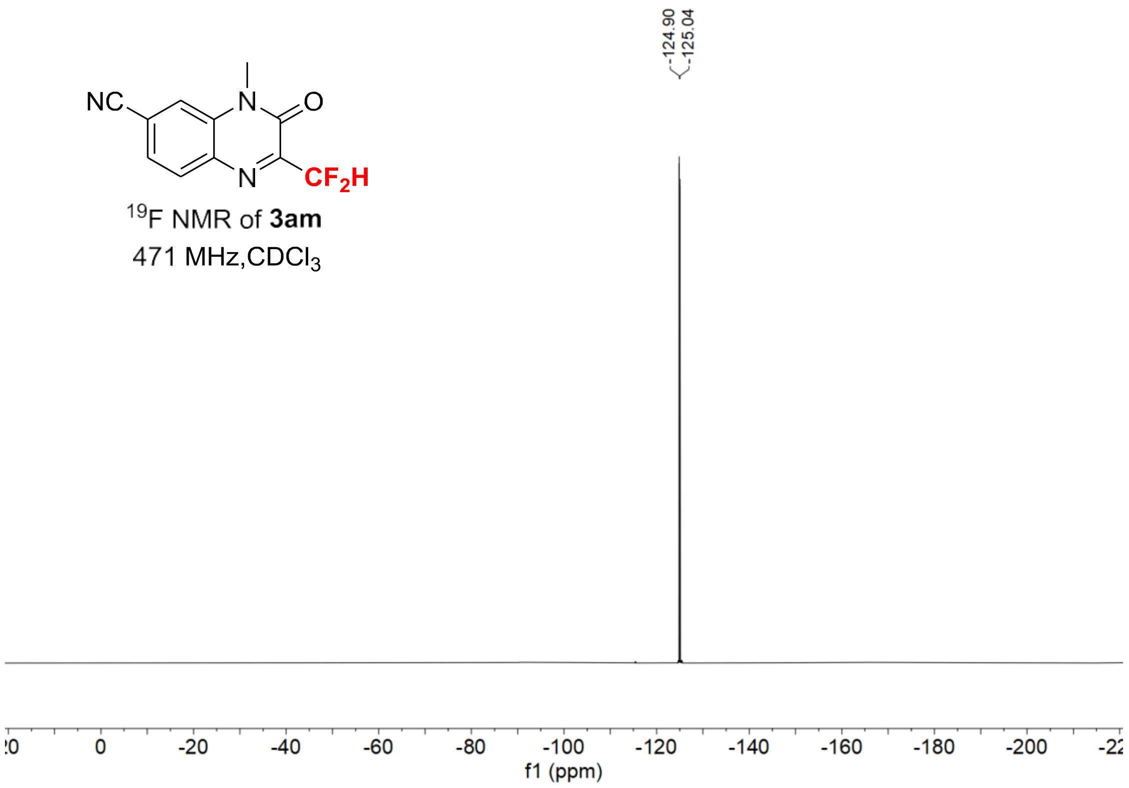

7-trifluoromethyl-3-(difluoromethyl)-1-methylquinoxalin-2(1*H*)-one (3an)

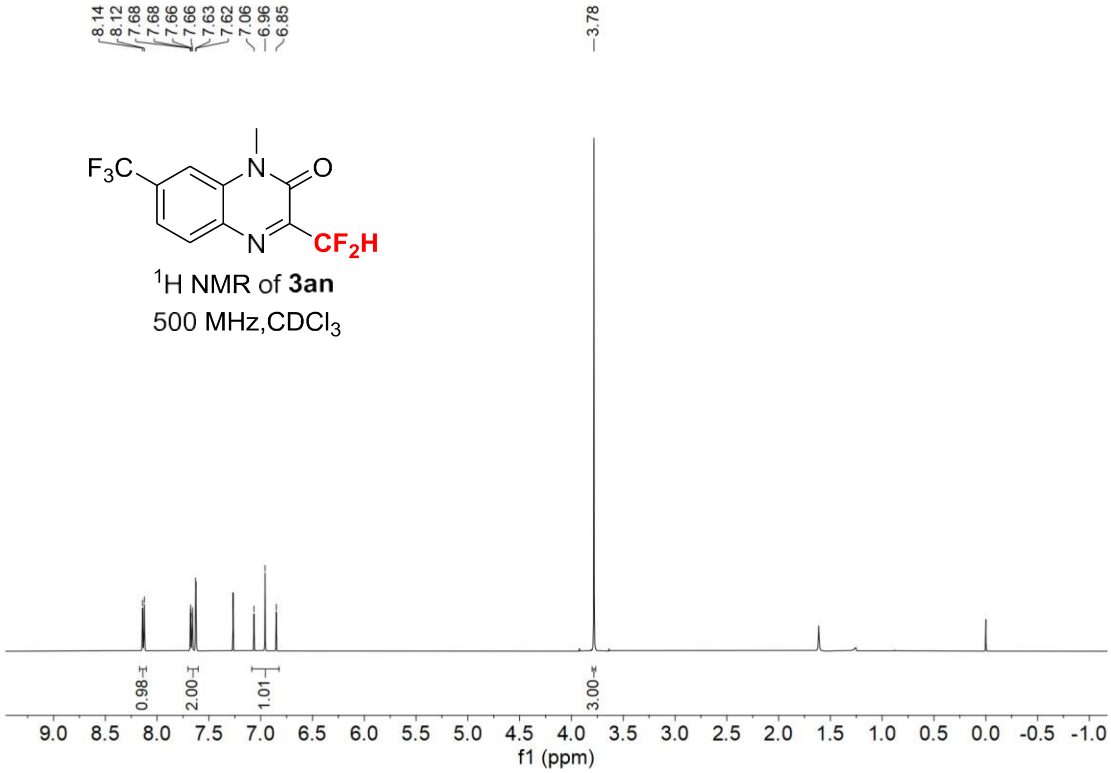

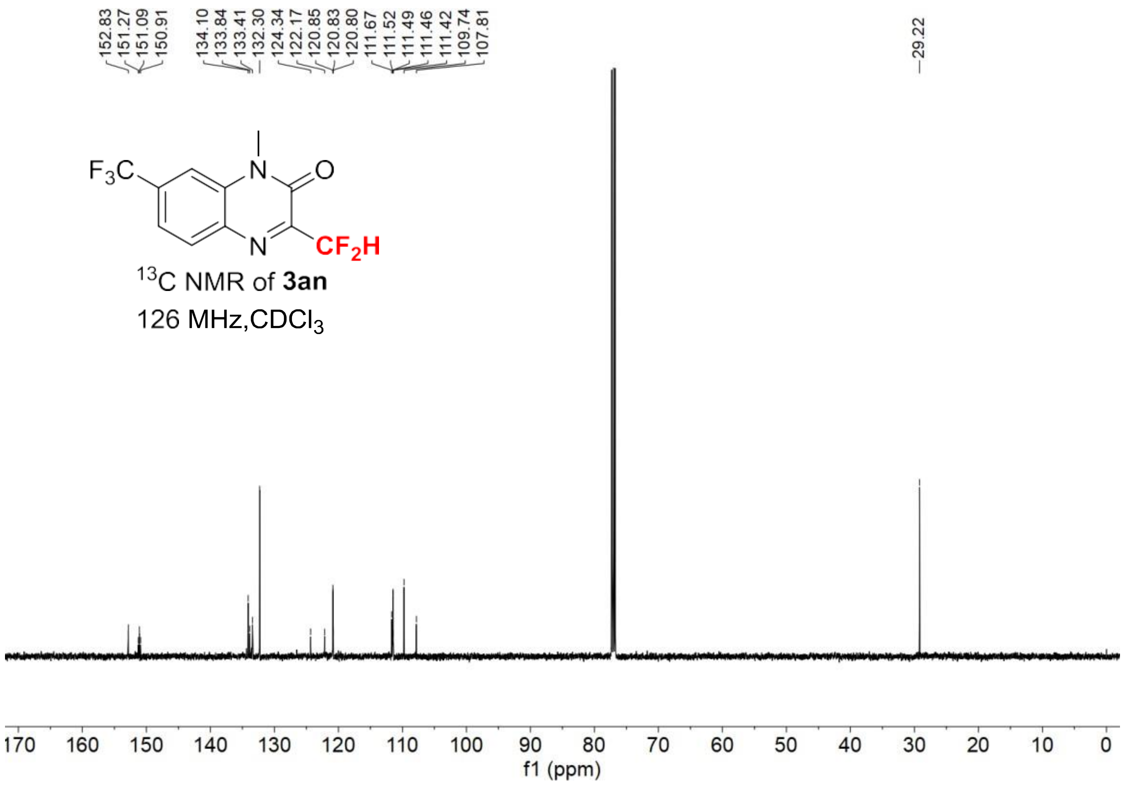

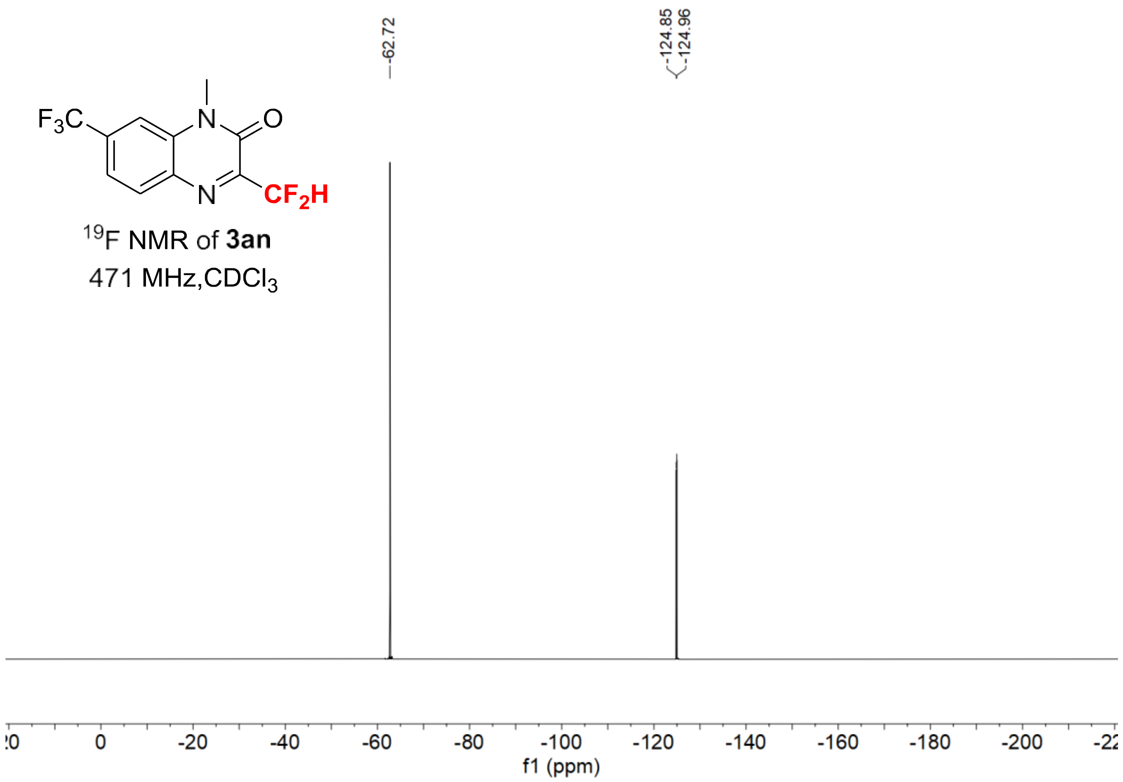

Methyl-3-(difluoromethyl)-1-methyl-2-oxo-1,2- dihydroquinoxaline-7-carboxylate (3ao)

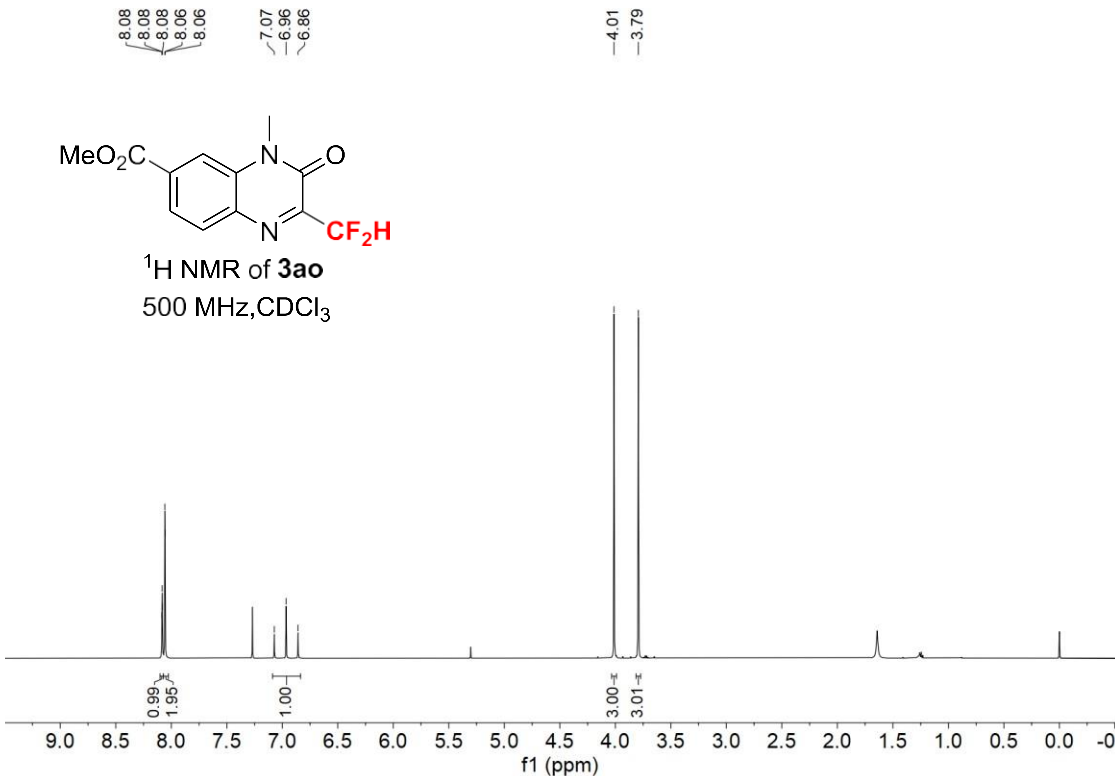
8.08
8.08
8.08
8.06
8.06
7.07
6.96
6.86
4.01
3.79
MeO2C
N
O
N
CF2H
1H NMR of 3ao
500 MHz,CDCl3
0.99
1.95
1.00
3.00
3.01
f1 (ppm)

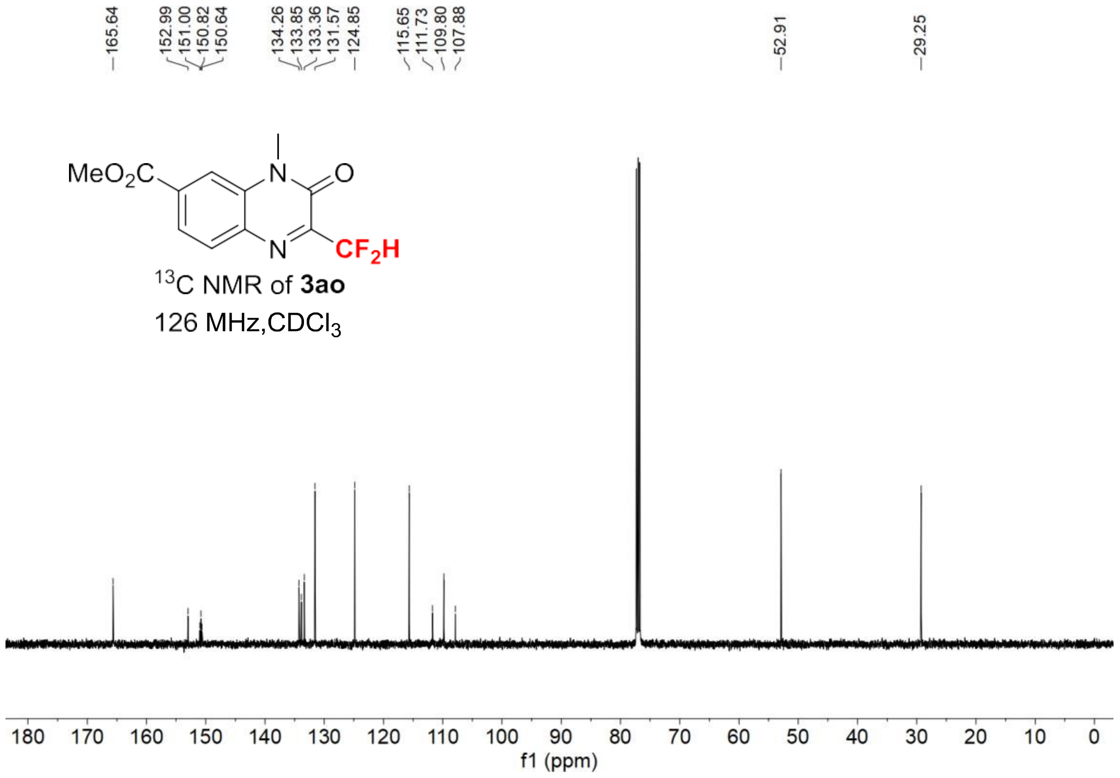
165.64
152.99
151.00
150.82
150.64
134.26
133.85
133.36
131.57
124.85
115.65
111.73
109.80
107.88
52.91
29.25
MeO2C
N
O
N
CF2H
13C NMR of 3ao
126 MHz,CDCl3
f1 (ppm)

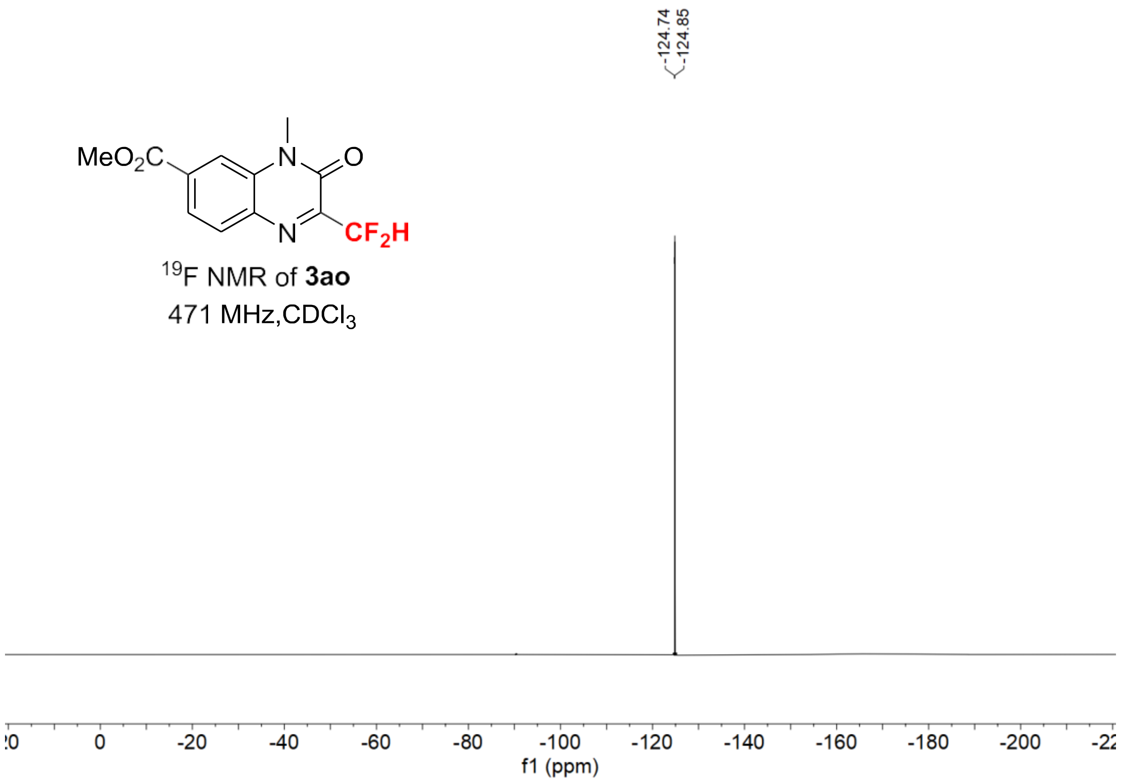

7-(tert-butyl)-3-(difluoromethyl)-1-methylquinoxalin-2(1*H*)-one (3ap)

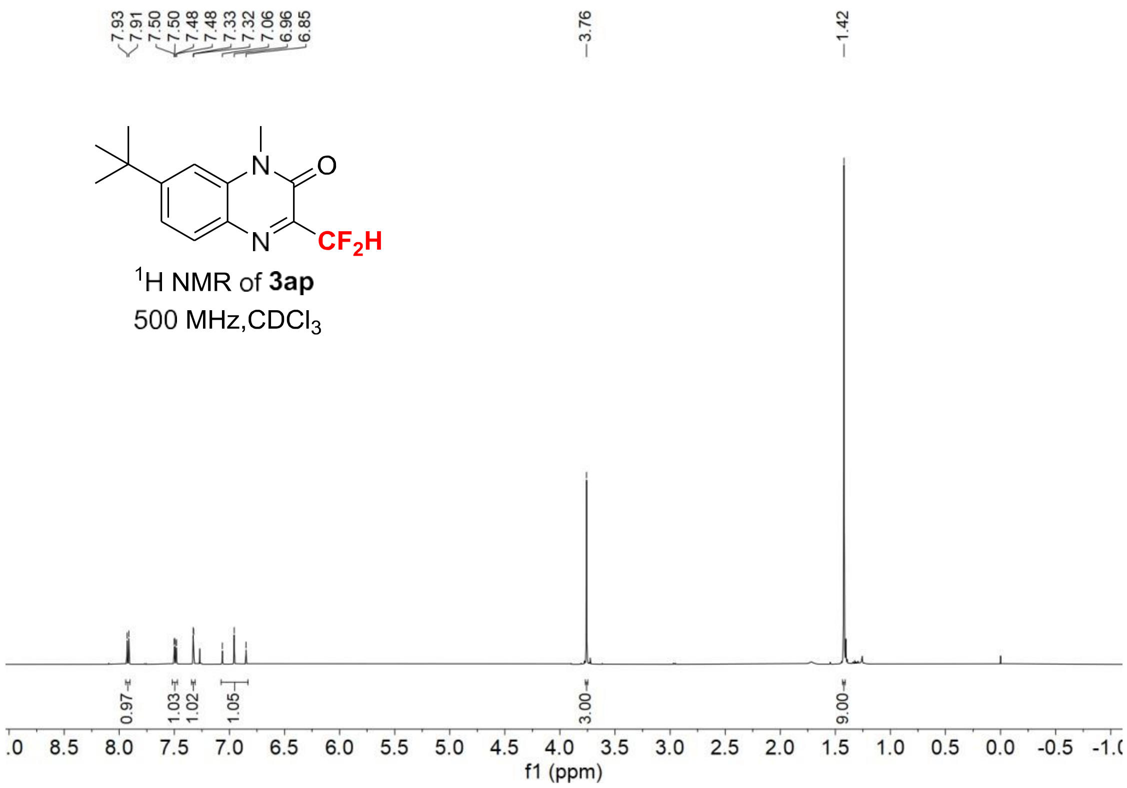

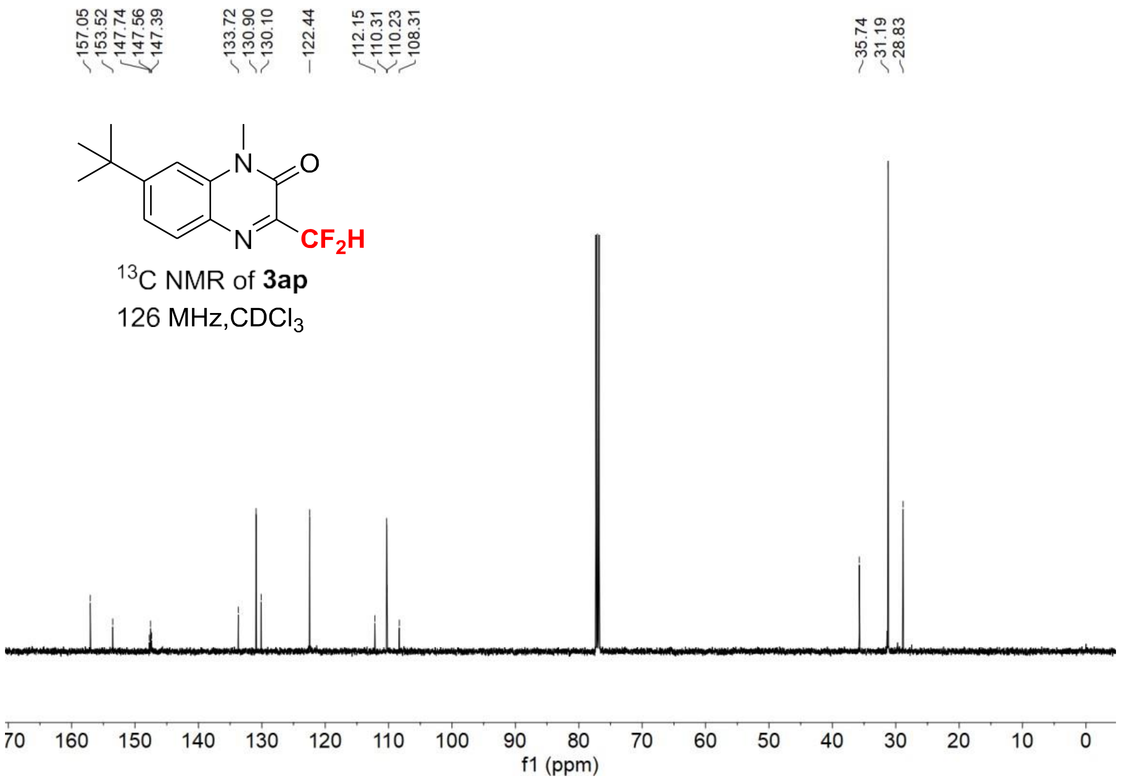

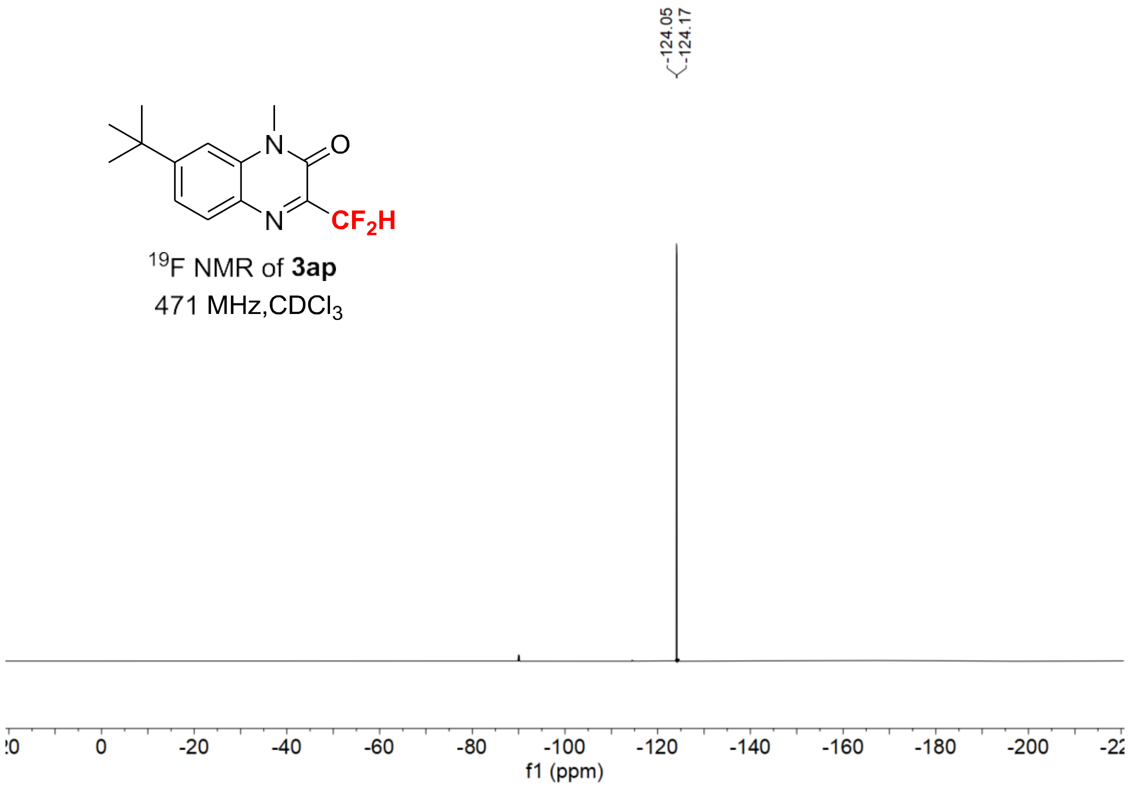

3-(difluoromethyl)-1,5-dimethylquinoxalin-2(1*H*)-one (3aq)

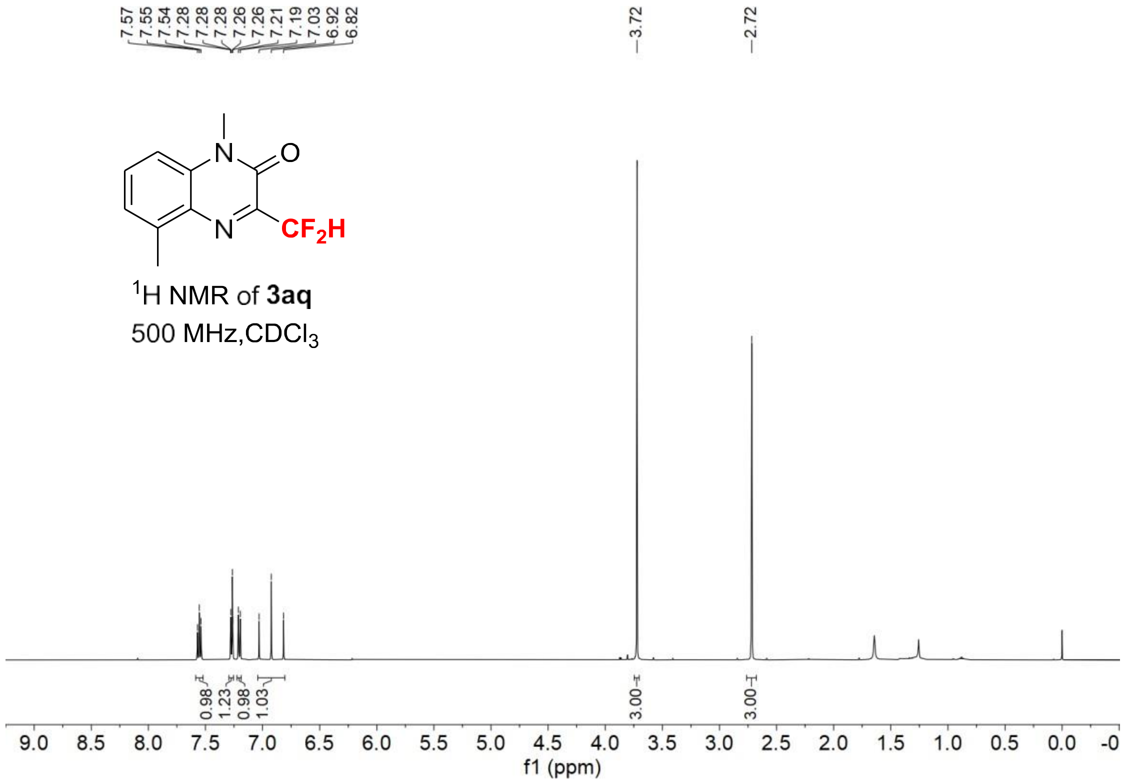
7.57
7.55
7.54
7.28
7.28
7.28
7.26
7.26
7.21
7.19
7.03
6.92
6.82
3.72
2.72
N
O
N
CF2H
1H NMR of 3aq
500 MHz,CDCl3
0.98
1.23
0.98
1.03
3.00
3.00
9.0 8.5 8.0 7.5 7.0 6.5 6.0 5.5 5.0 4.5 4.0 3.5 3.0 2.5 2.0 1.5 1.0 0.5 0.0 -0
f1 (ppm)

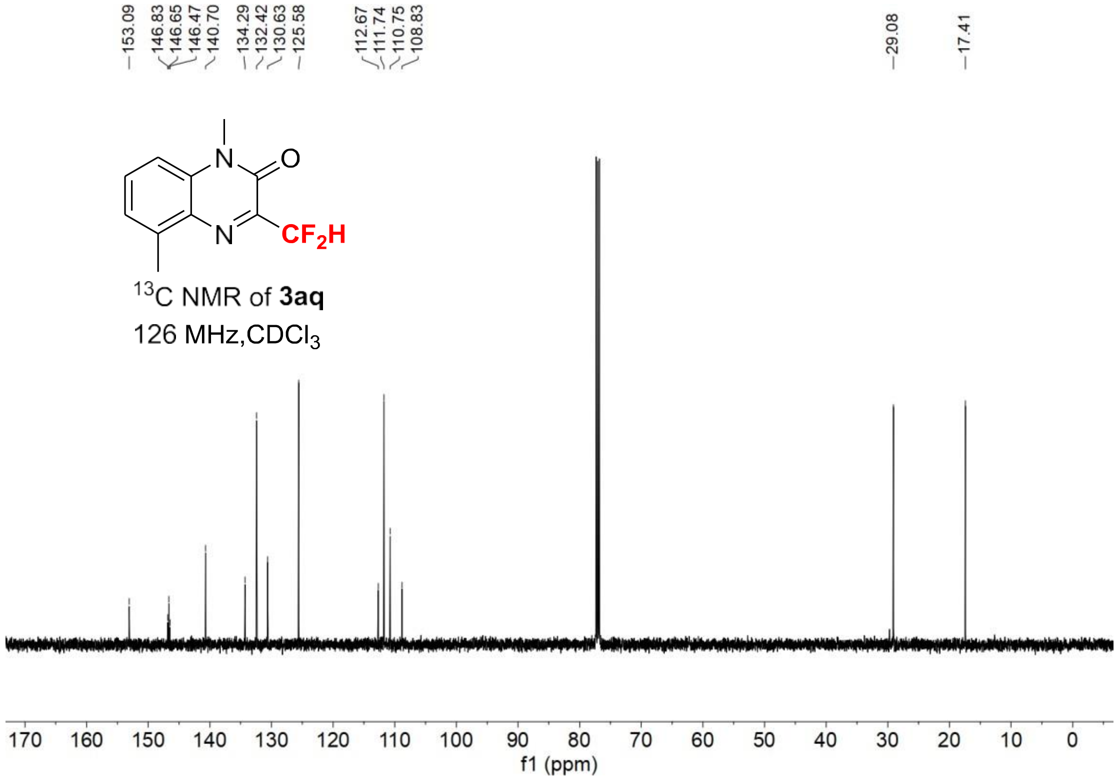
153.09
146.83
146.65
146.47
140.70
134.29
132.42
130.63
125.58
112.67
111.74
110.75
108.83
29.08
17.41
N
O
N
CF2H
13C NMR of 3aq
126 MHz,CDCl3
170 160 150 140 130 120 110 100 90 80 70 60 50 40 30 20 10 0
f1 (ppm)

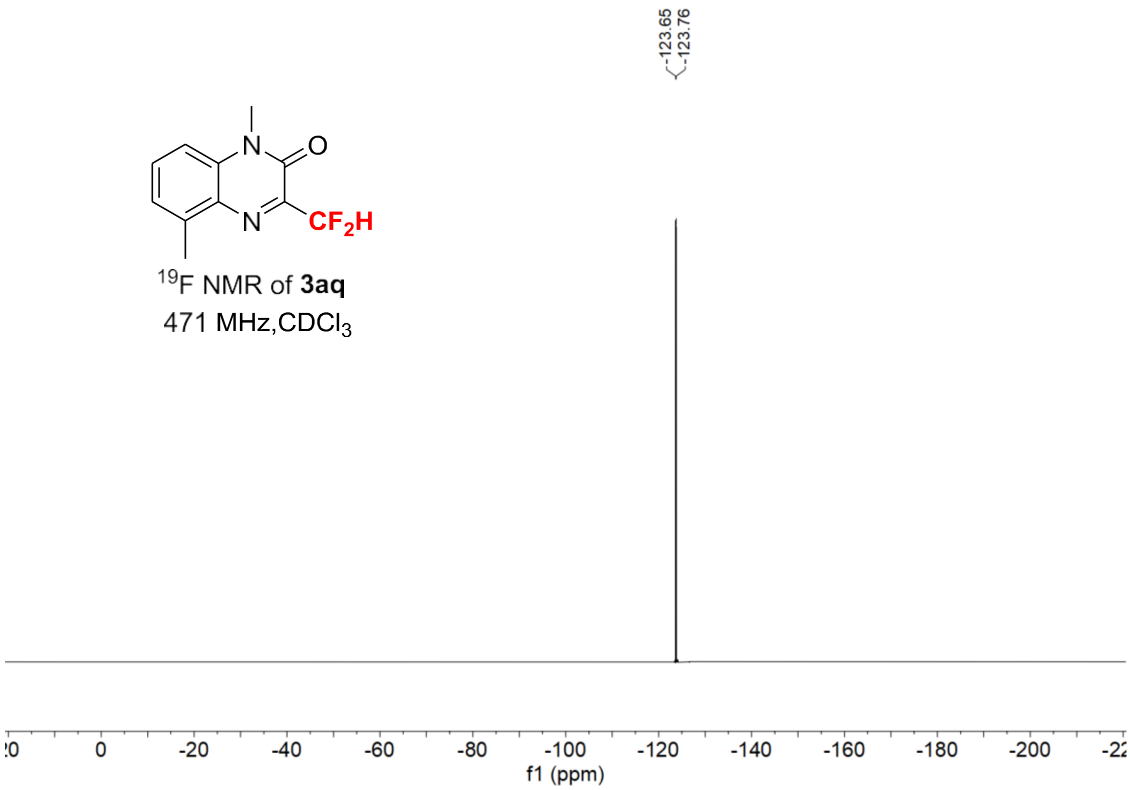

6,7-difluoro-1-methyl-3-(difluoromethyl)quinoxalin-2(1*H*)-one (3ar)

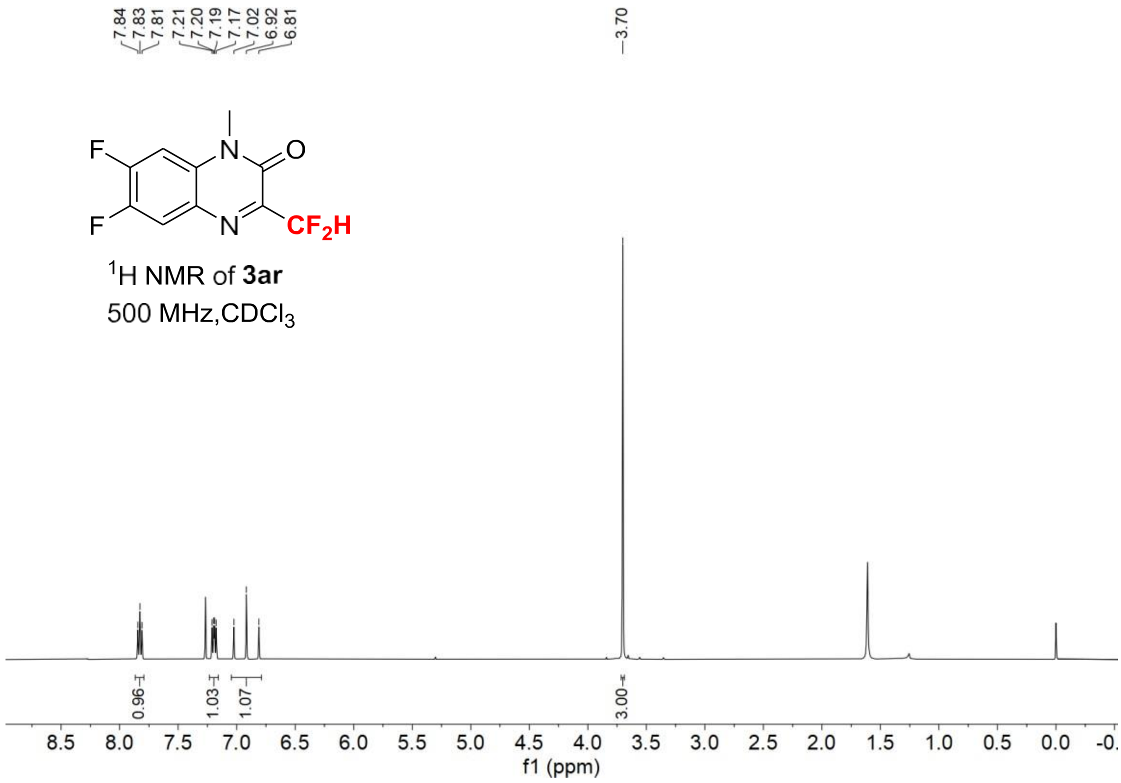

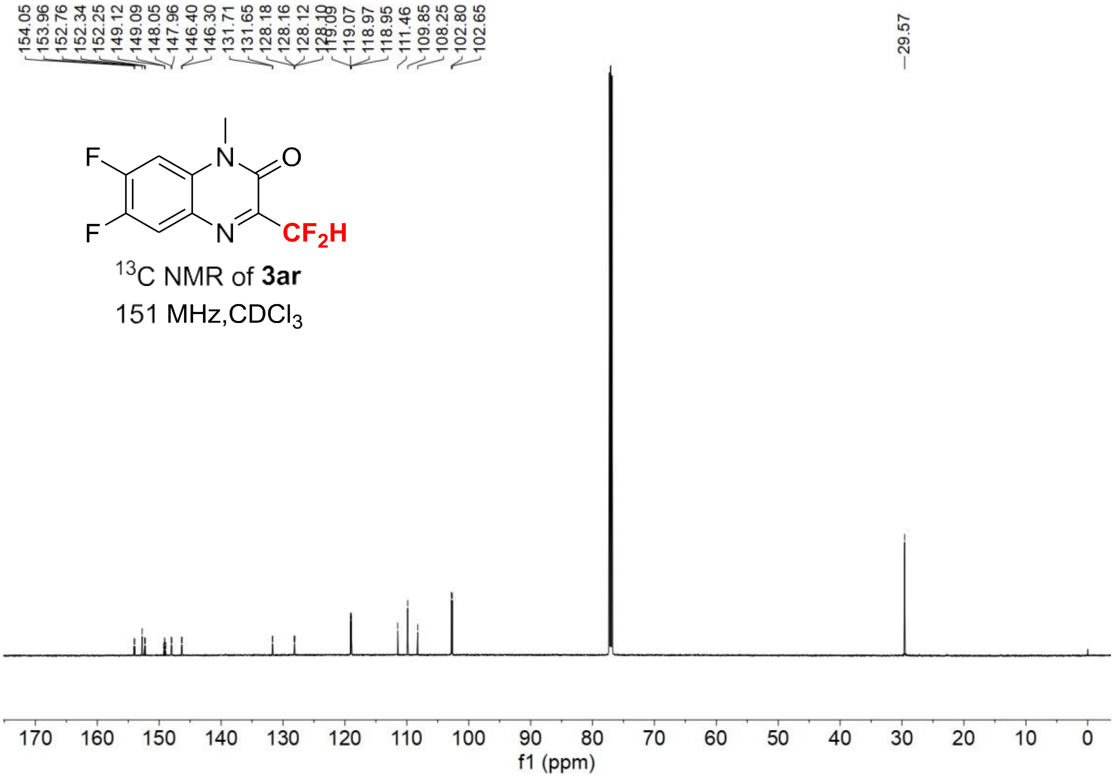

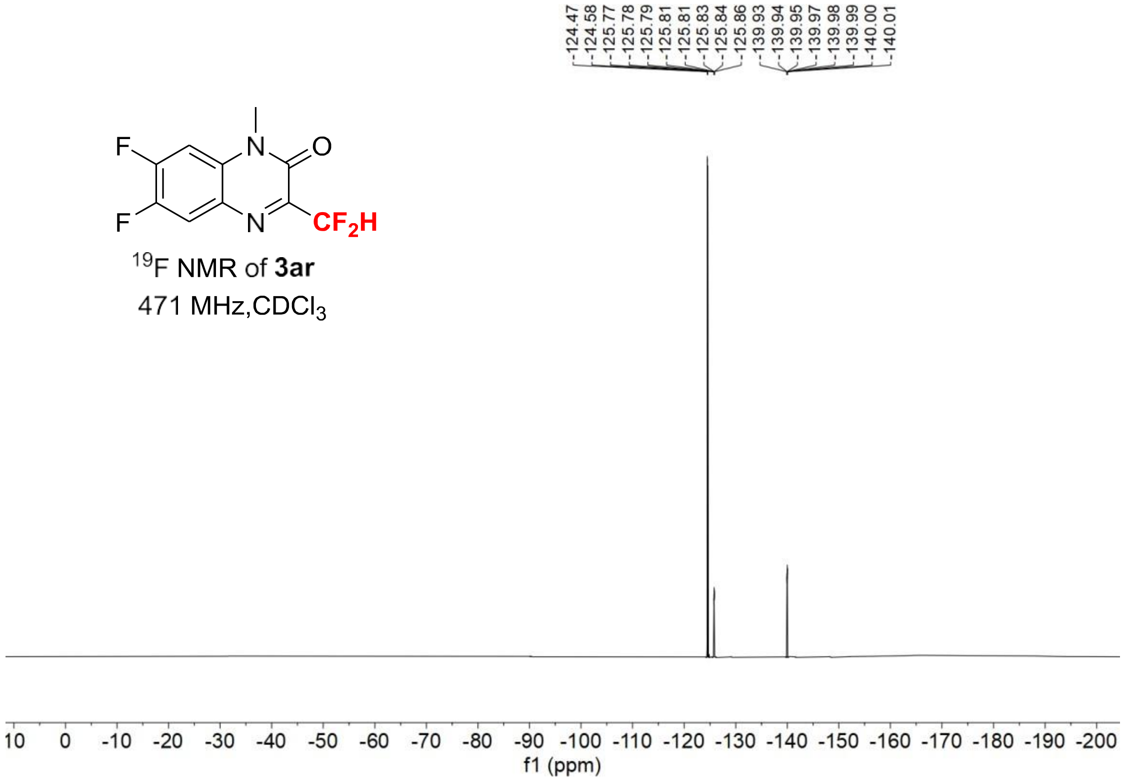

6,7-dichloro-3-(difluoromethyl)-1-methylquinoxalin-2(1*H*)-one (3as)

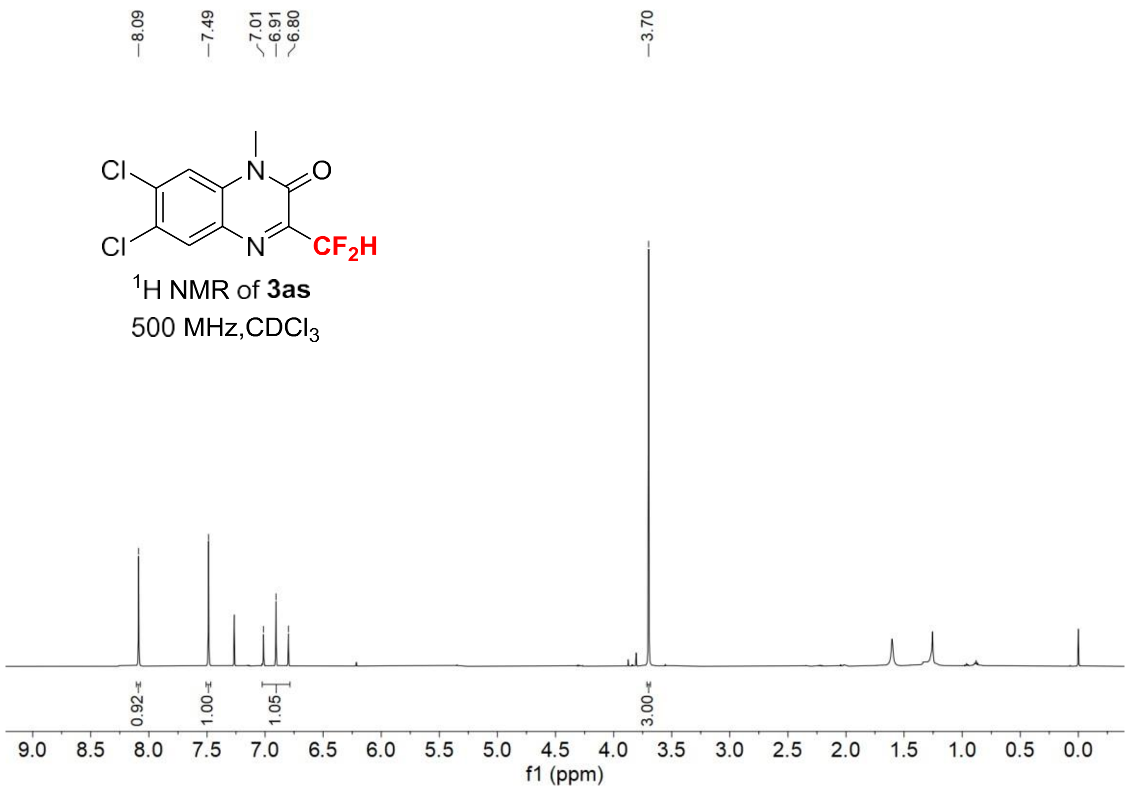
8.09
7.49
7.01
6.91
6.80
3.70
Cl
Cl
N
O
N
CF2H
1H NMR of 3as
500 MHz,CDCl3
0.92
1.00
1.05
3.00
9.0 8.5 8.0 7.5 7.0 6.5 6.0 5.5 5.0 4.5 4.0 3.5 3.0 2.5 2.0 1.5 1.0 0.5 0.0
f1 (ppm)

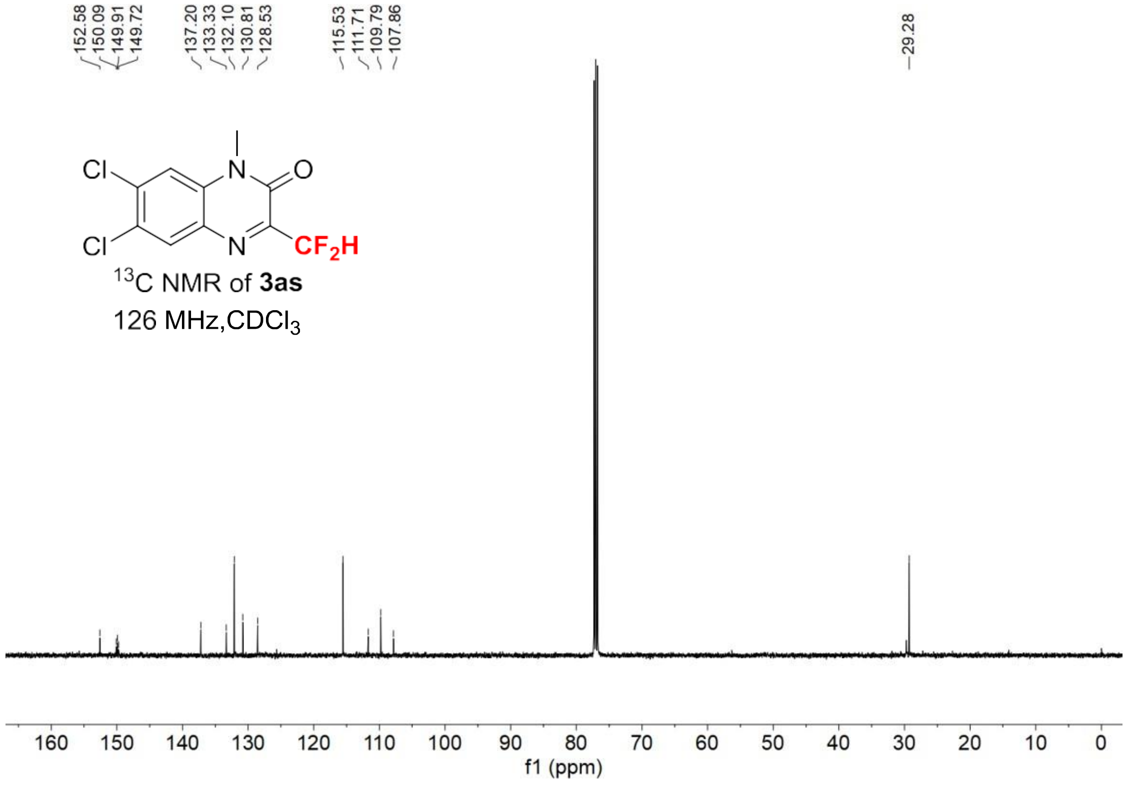
152.58
150.09
149.91
149.72
137.20
133.33
132.10
130.81
128.53
115.53
111.71
109.79
107.86
29.28
Cl
Cl
N
O
N
CF2H
13C NMR of 3as
126 MHz,CDCl3
160 150 140 130 120 110 100 90 80 70 60 50 40 30 20 10 0
f1 (ppm)

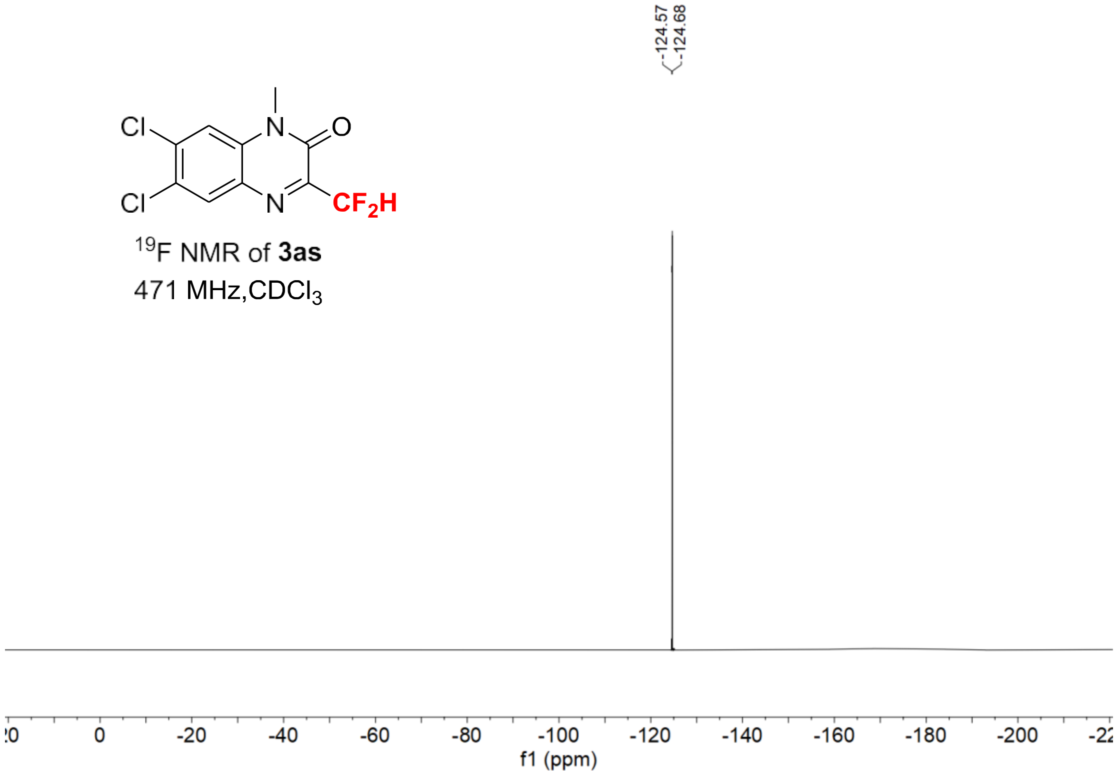

6,7-bromo-1-methyl-3-(difluoromethyl)quinoxalin-2(1*H*)-one (3at)

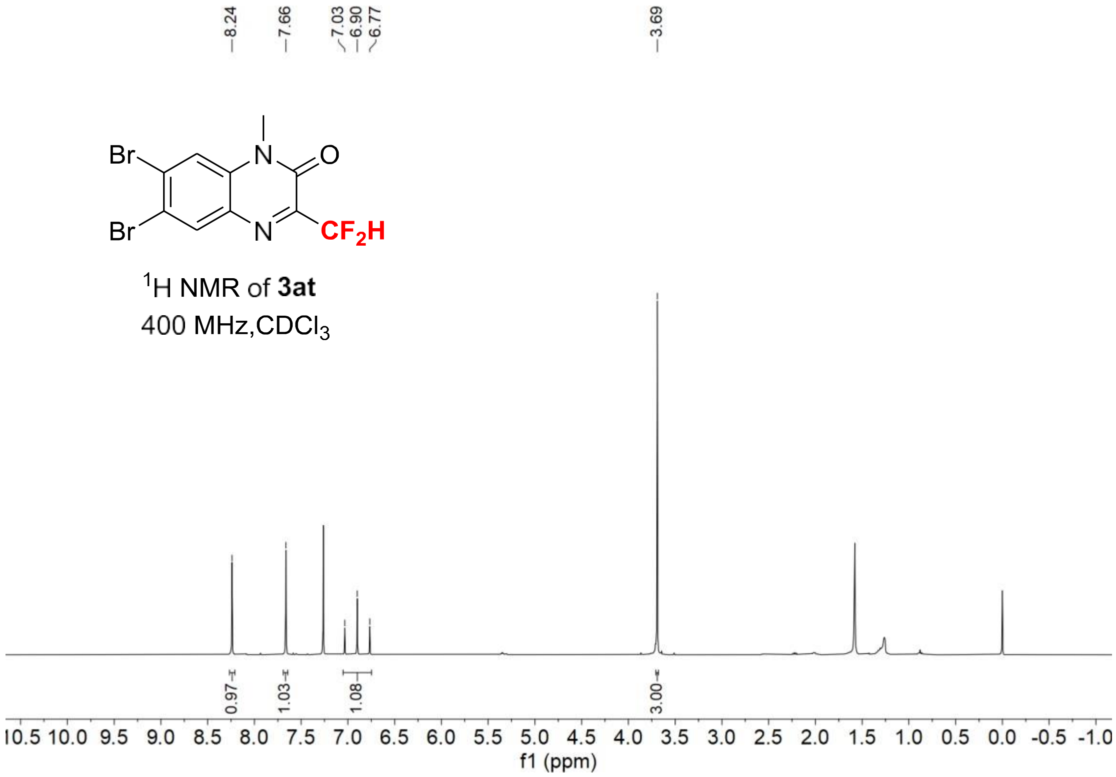

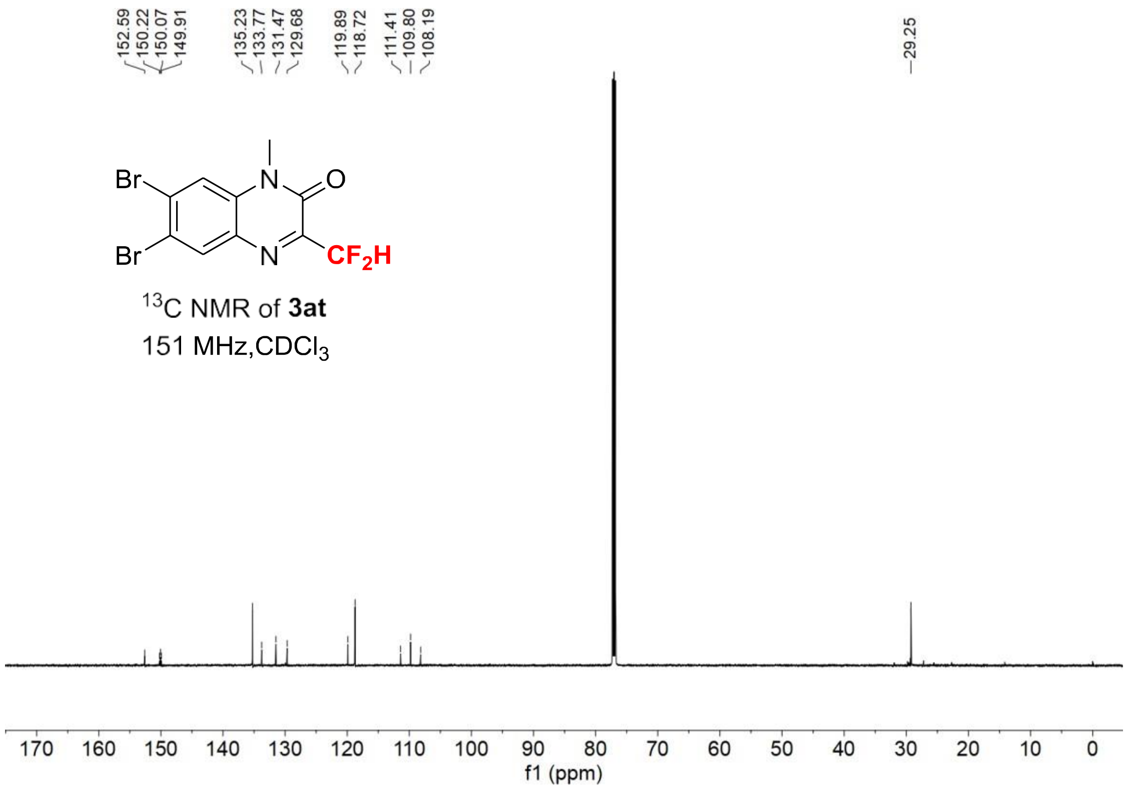

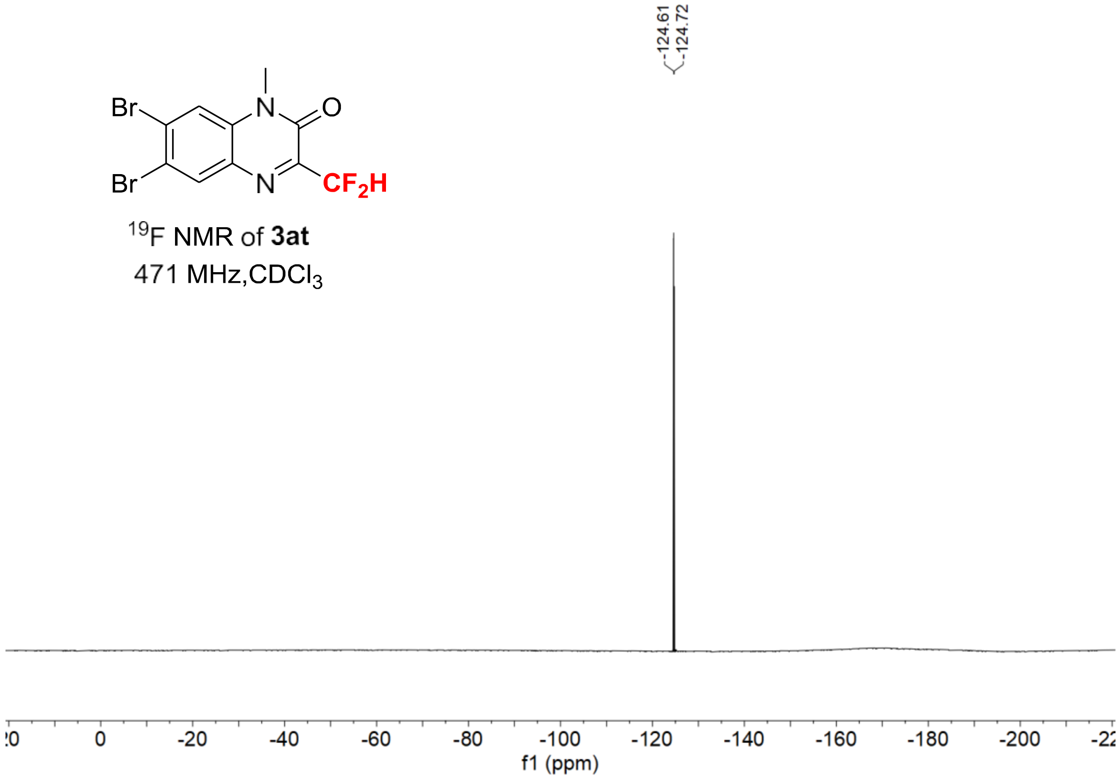

1,6,7-trimethyl-3-(difluoromethyl)quinoxalin-2(1*H*)-one (3au)

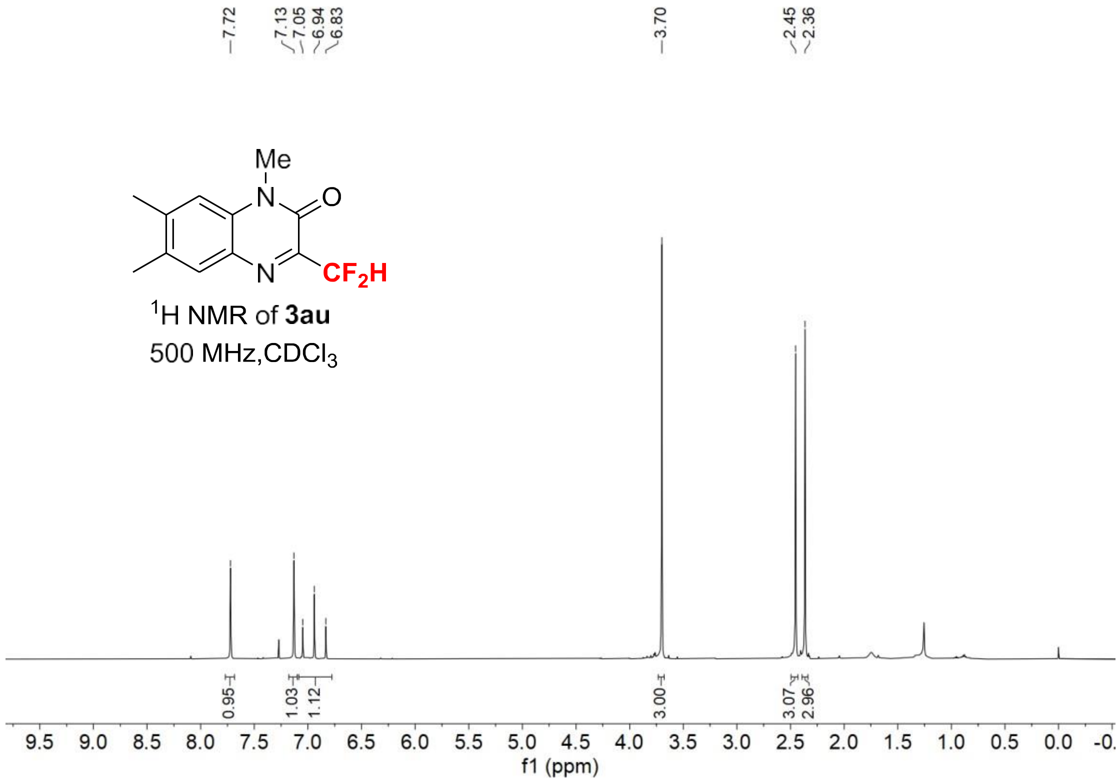

7.72
7.13
7.05
6.94
6.83
3.70
2.45
2.36
Me
N
O
N
CF2H
1H NMR of 3au
500 MHz,CDCl3
0.95
1.03
1.12
3.00
3.07
2.96
9.5 9.0 8.5 8.0 7.5 7.0 6.5 6.0 5.5 5.0 4.5 4.0 3.5 3.0 2.5 2.0 1.5 1.0 0.5 0.0
f1 (ppm)

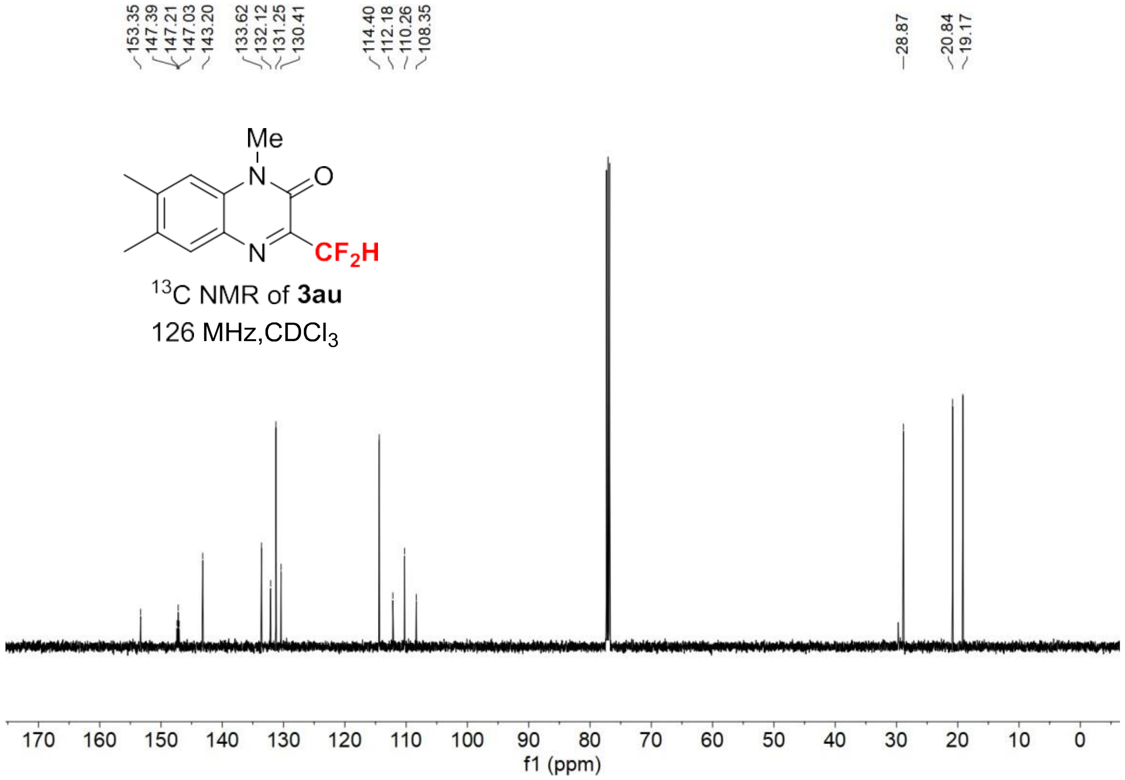

153.35
147.39
147.21
147.03
143.20
133.62
132.12
131.25
130.41
114.40
112.18
110.26
108.35
28.87
20.84
19.17
Me
N
O
N
CF2H
13C NMR of 3au
126 MHz,CDCl3
170 160 150 140 130 120 110 100 90 80 70 60 50 40 30 20 10 0
f1 (ppm)

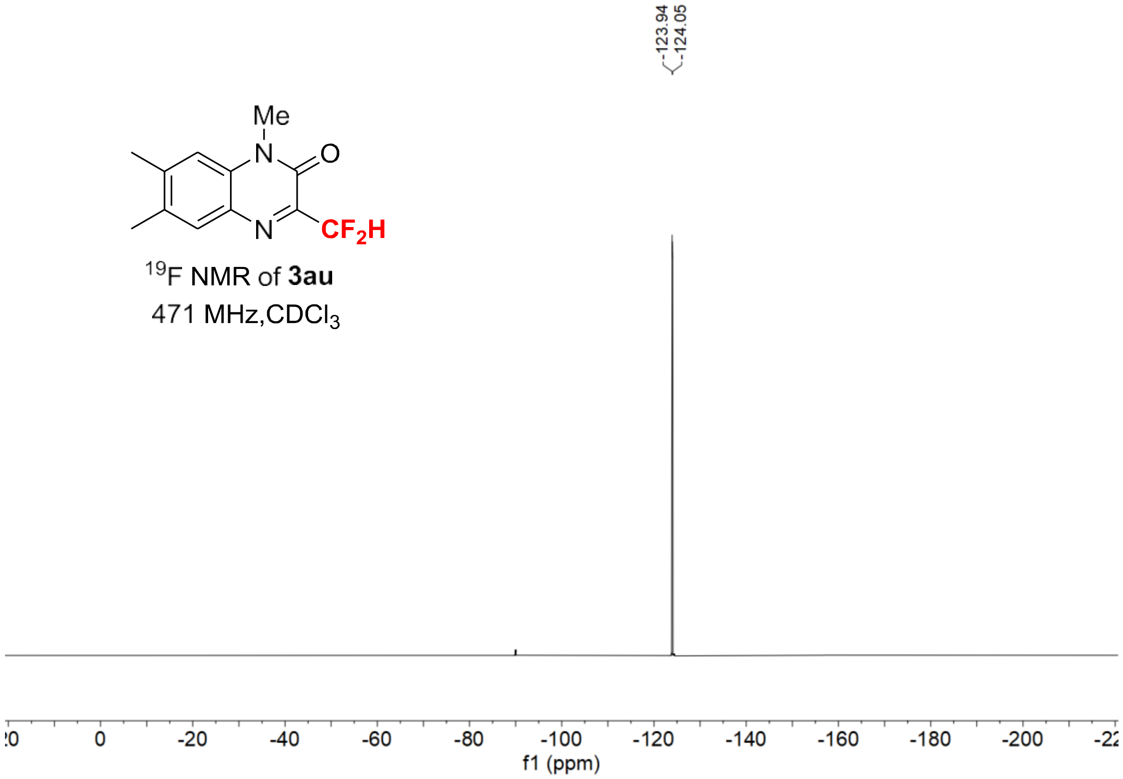

3-(difluoromethyl)-1-methylbenzo[g]quinoxalin-2(1*H*)-one (3av)

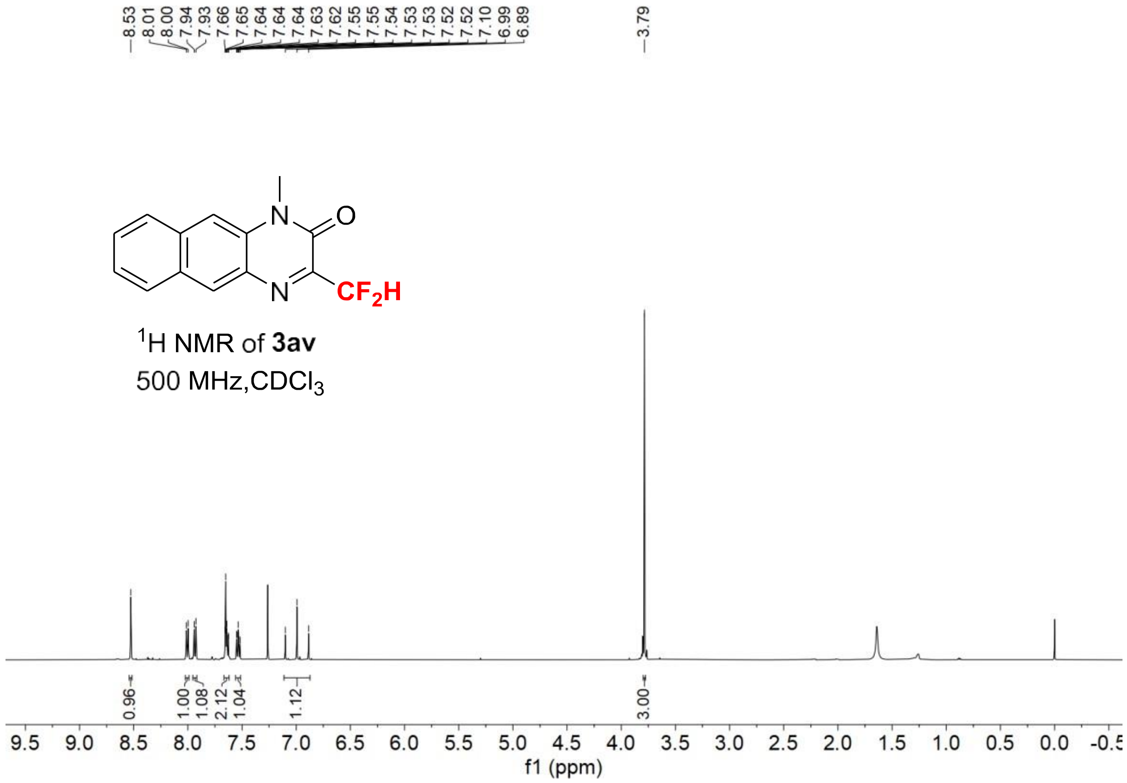

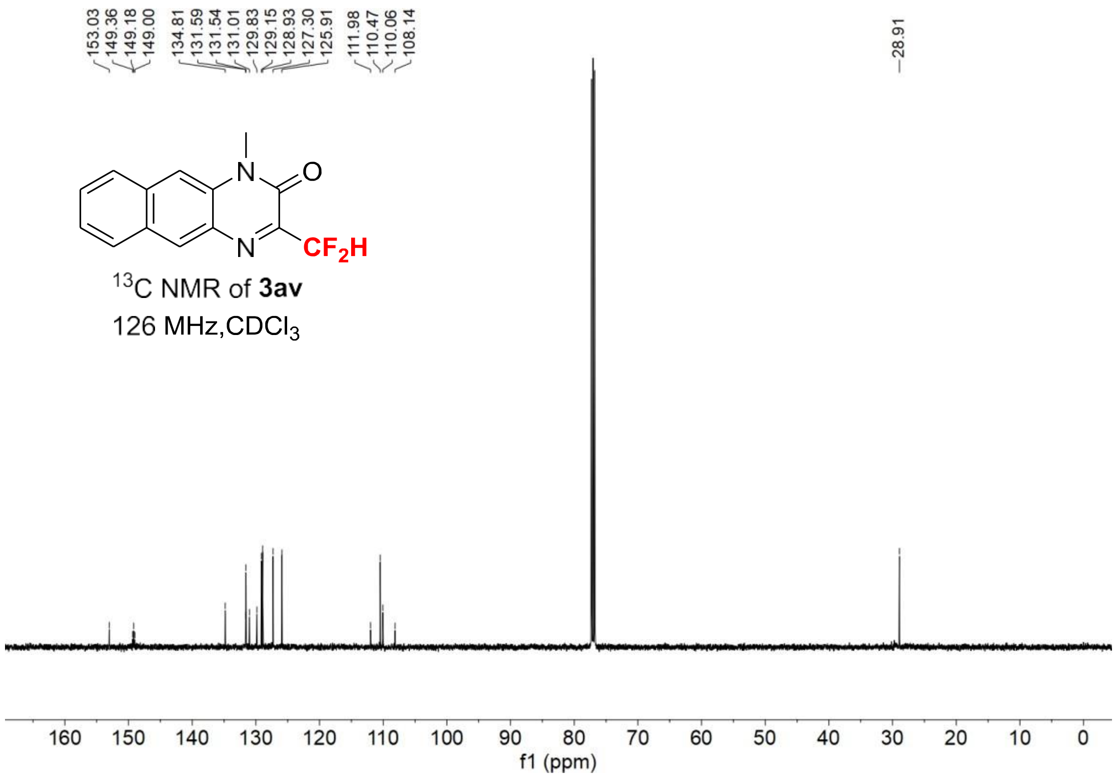

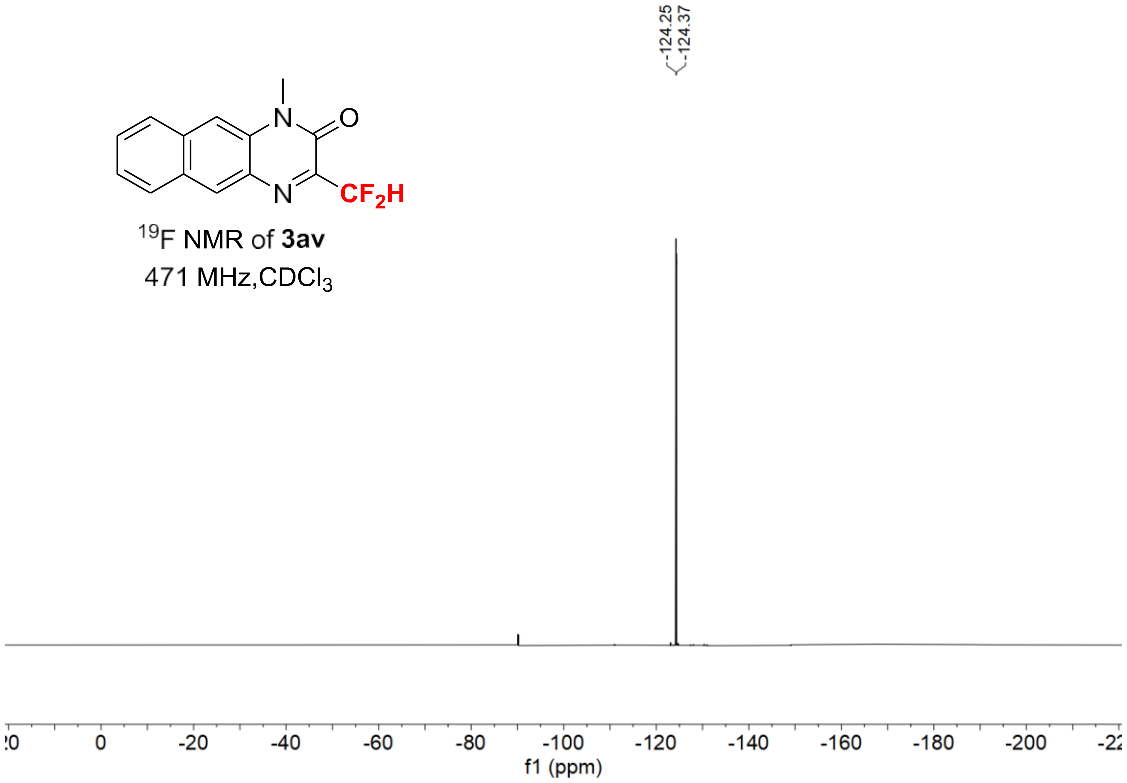

3-(difluoromethyl)quinoxalin-2(1*H*)-one (3ba)

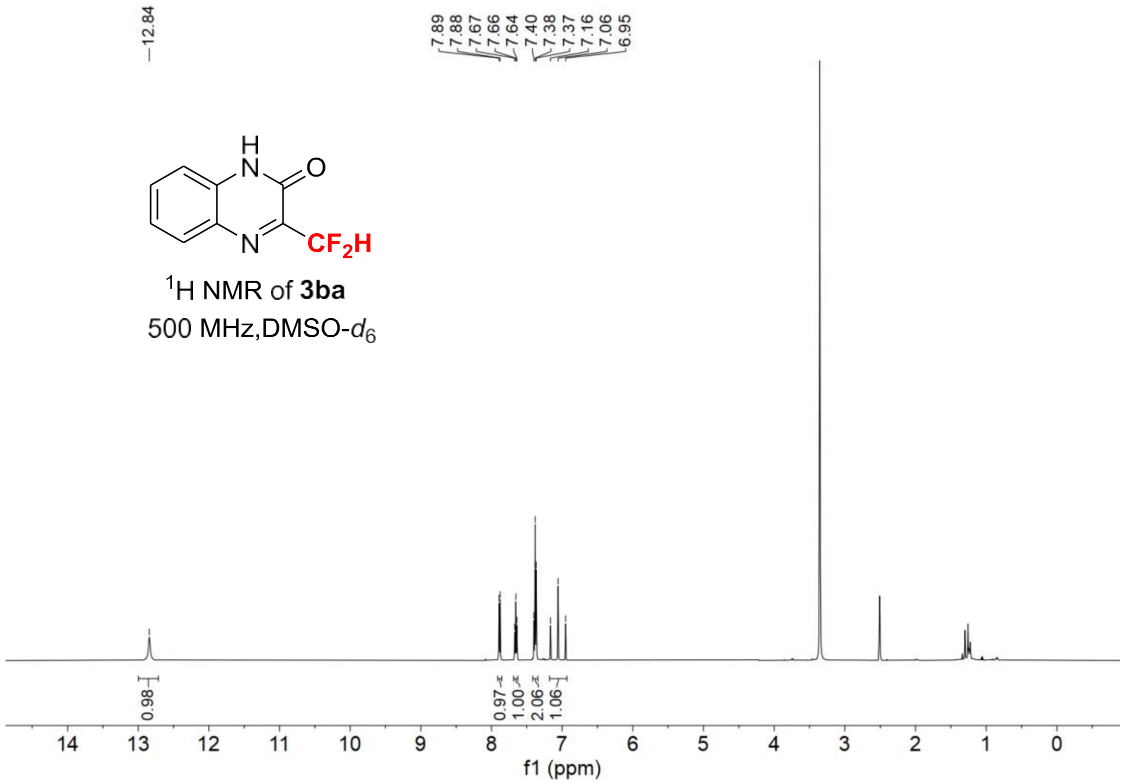

12.84
7.89
7.88
7.67
7.66
7.64
7.40
7.38
7.37
7.16
7.06
6.95
CF2H
1H NMR of 3ba
500 MHz,DMSO-d6
0.98
0.97
1.00
2.06
1.06
f1 (ppm)

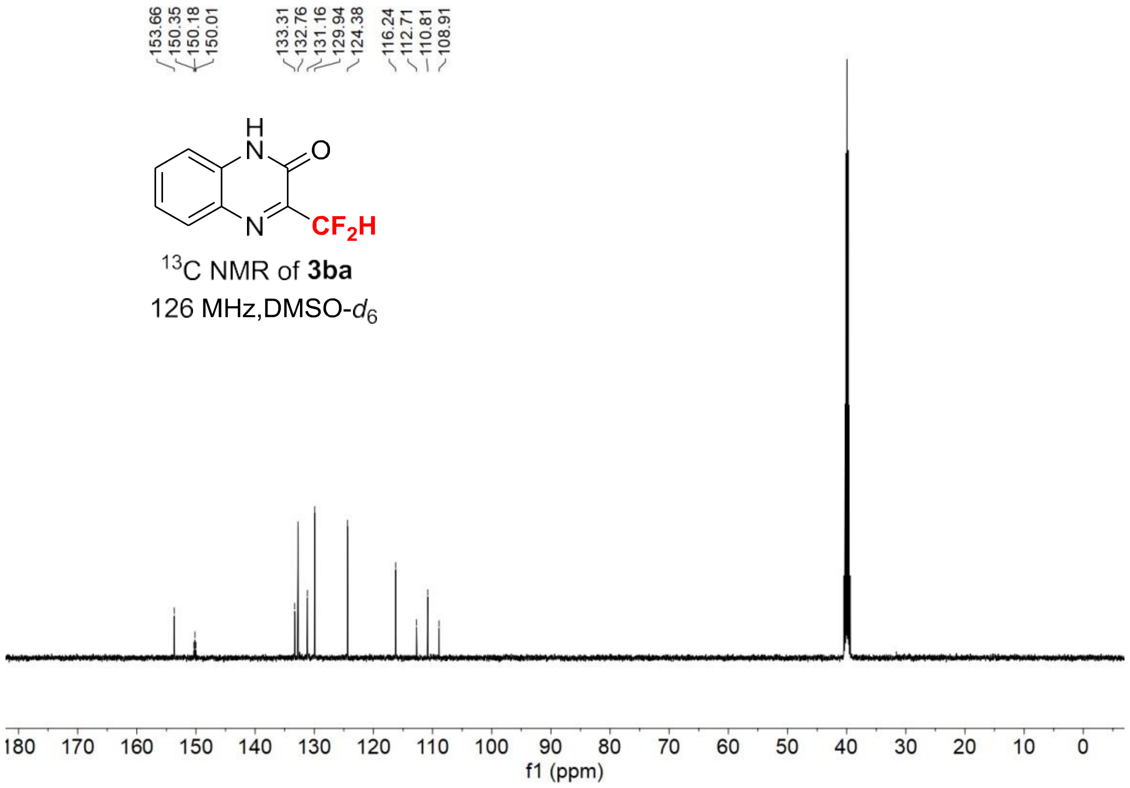

153.66
150.35
150.18
150.01
133.31
132.76
131.16
129.94
124.38
116.24
112.71
110.81
108.91
CF2H
13C NMR of 3ba
126 MHz,DMSO-d6
f1 (ppm)

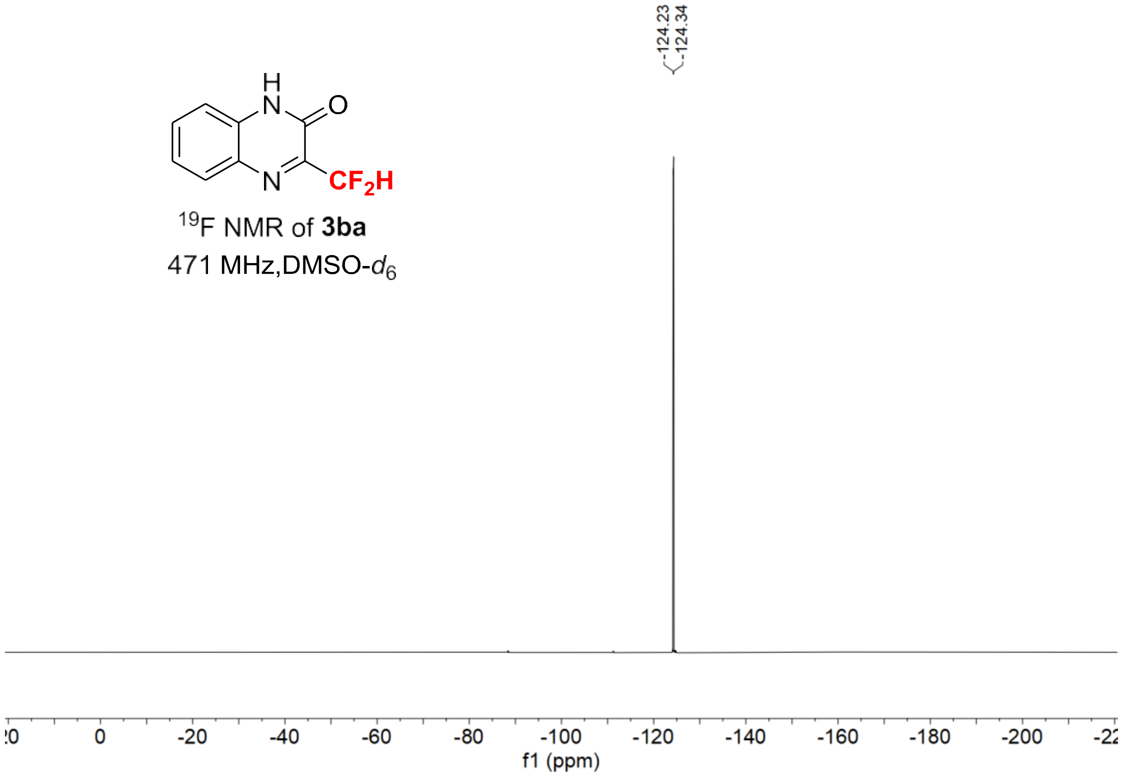

1-benzyl-3-(difluoromethyl)quinoxalin-2(1*H*)-one (3bb)

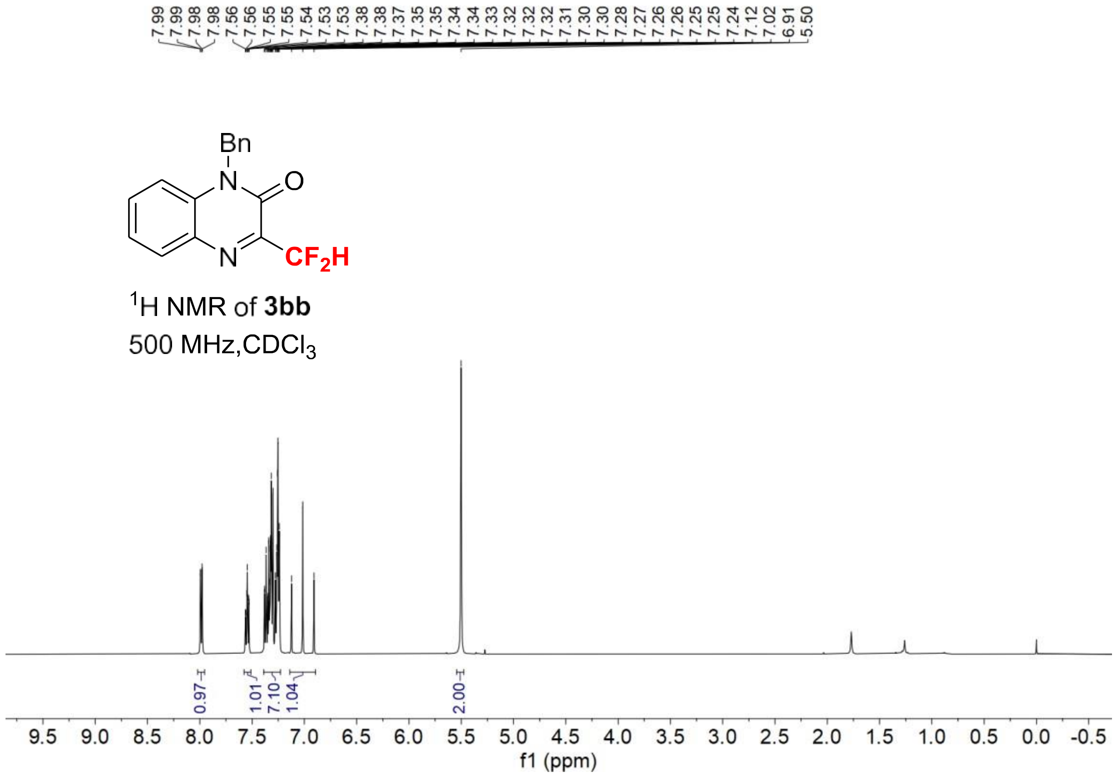

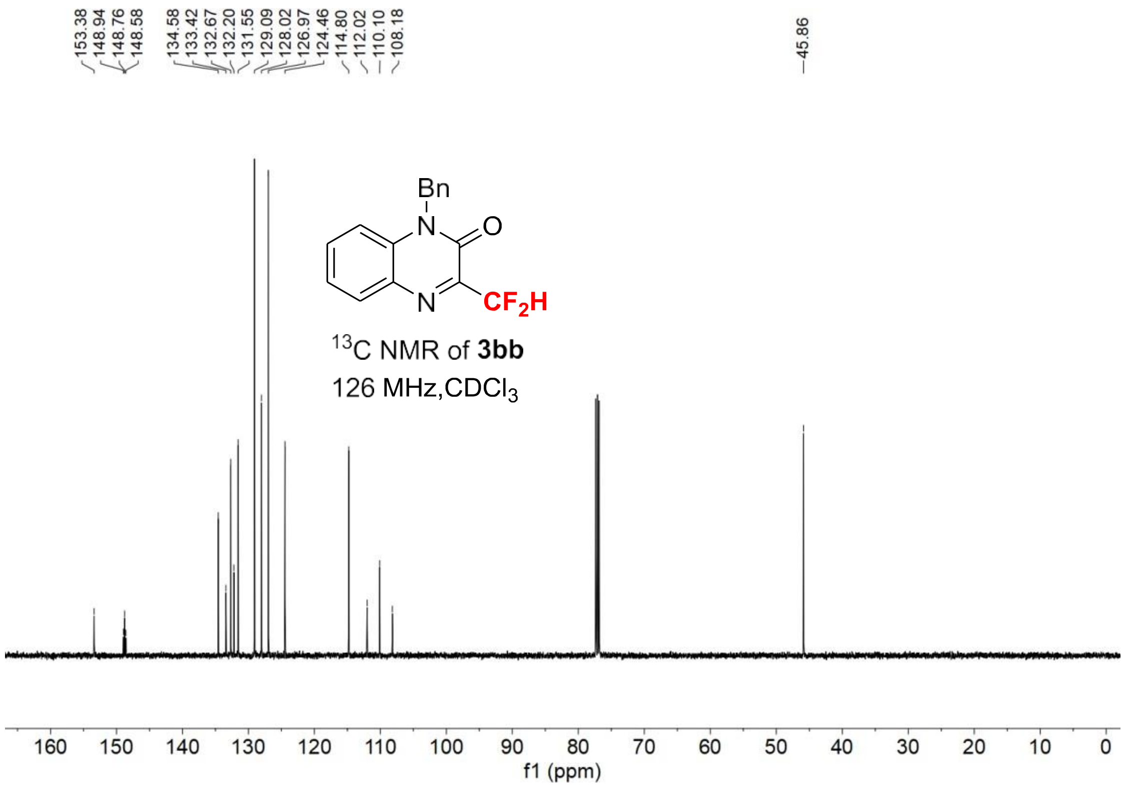

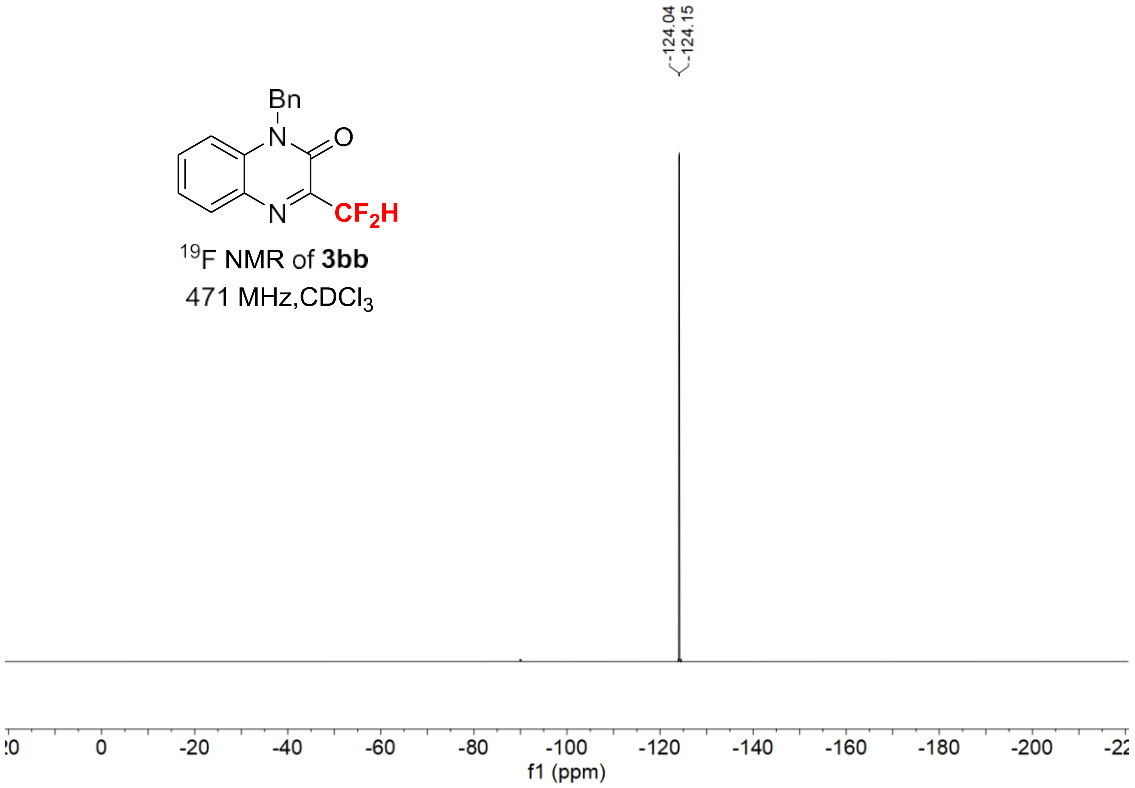

6,7-dimethyl-3-(difluoromethyl)-1-benzylquinoxalin-2(1*H*)-one (3bc)

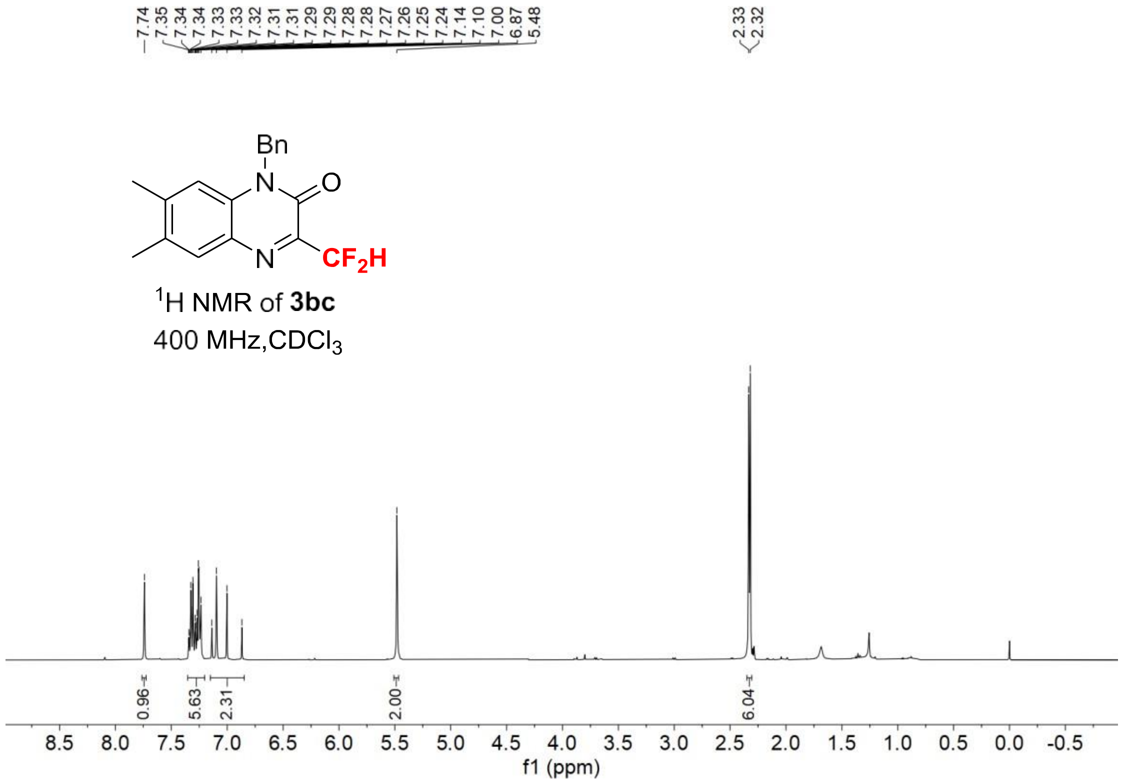

^{1}H NMR of **3bc**

400 MHz,$CDCl_3$

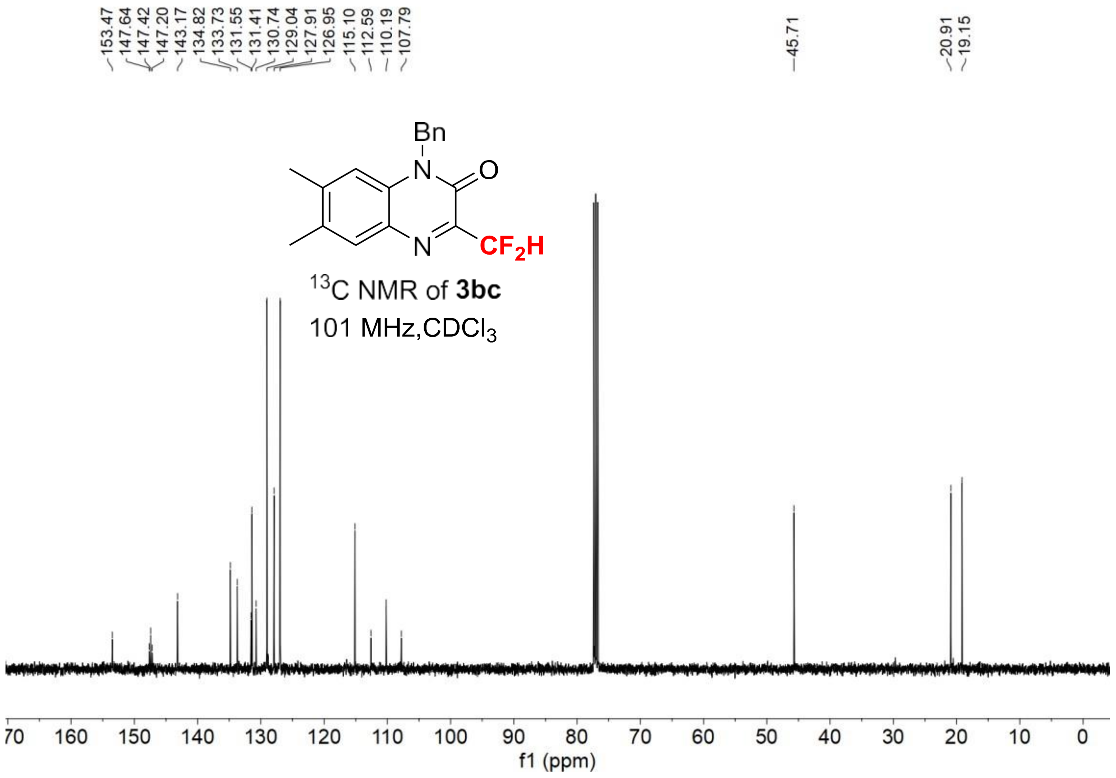

^{13}C NMR of **3bc**

101 MHz,$CDCl_3$

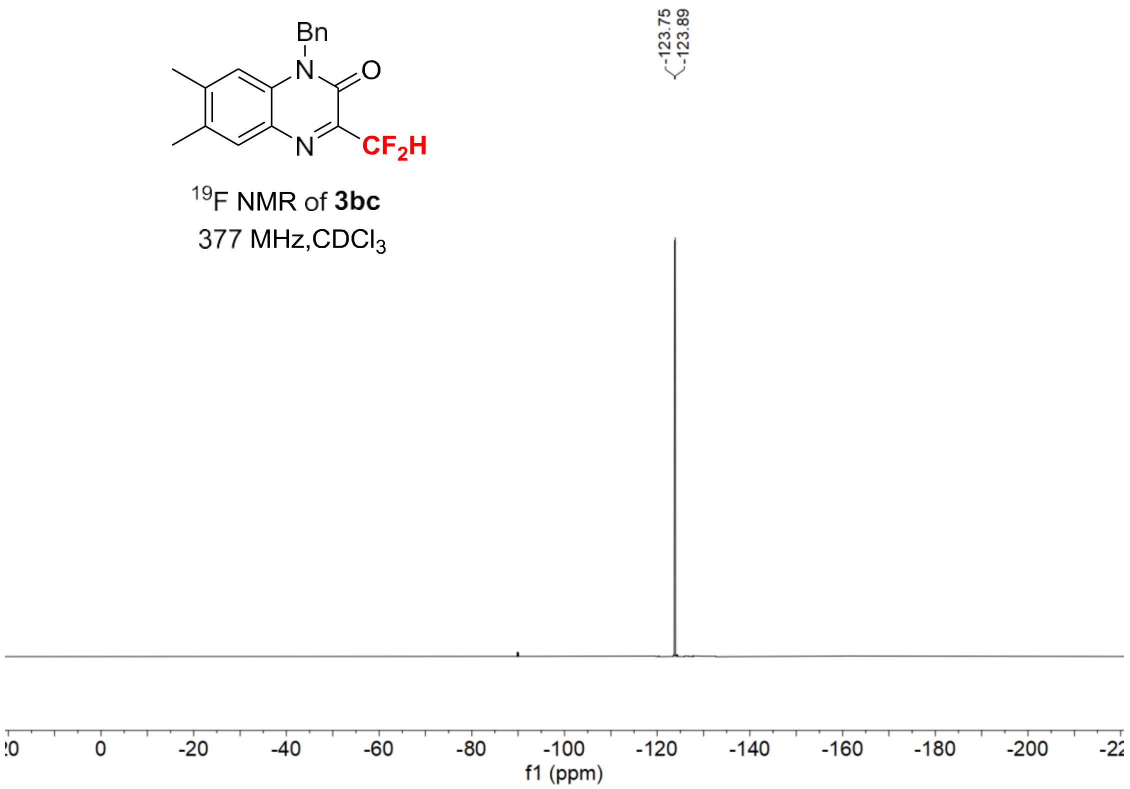

6,7-difluoro-3-(difluoromethyl)-1-benzylquinoxalin-2(1*H*)-one (3bd)

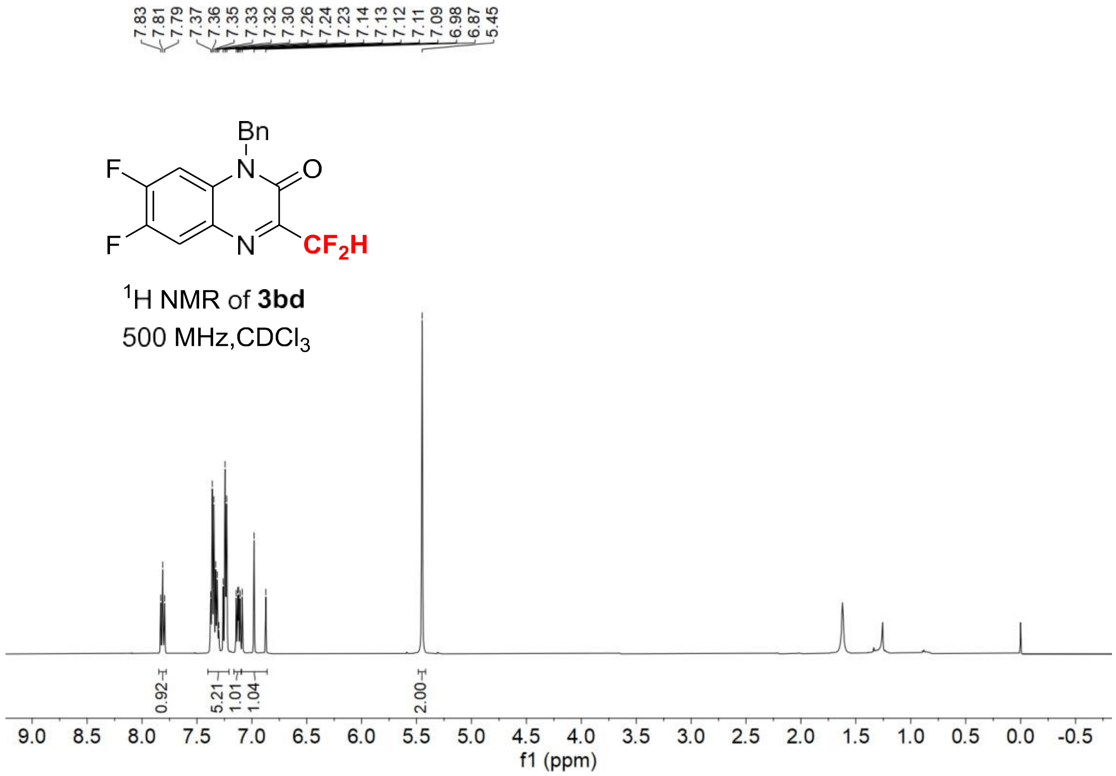

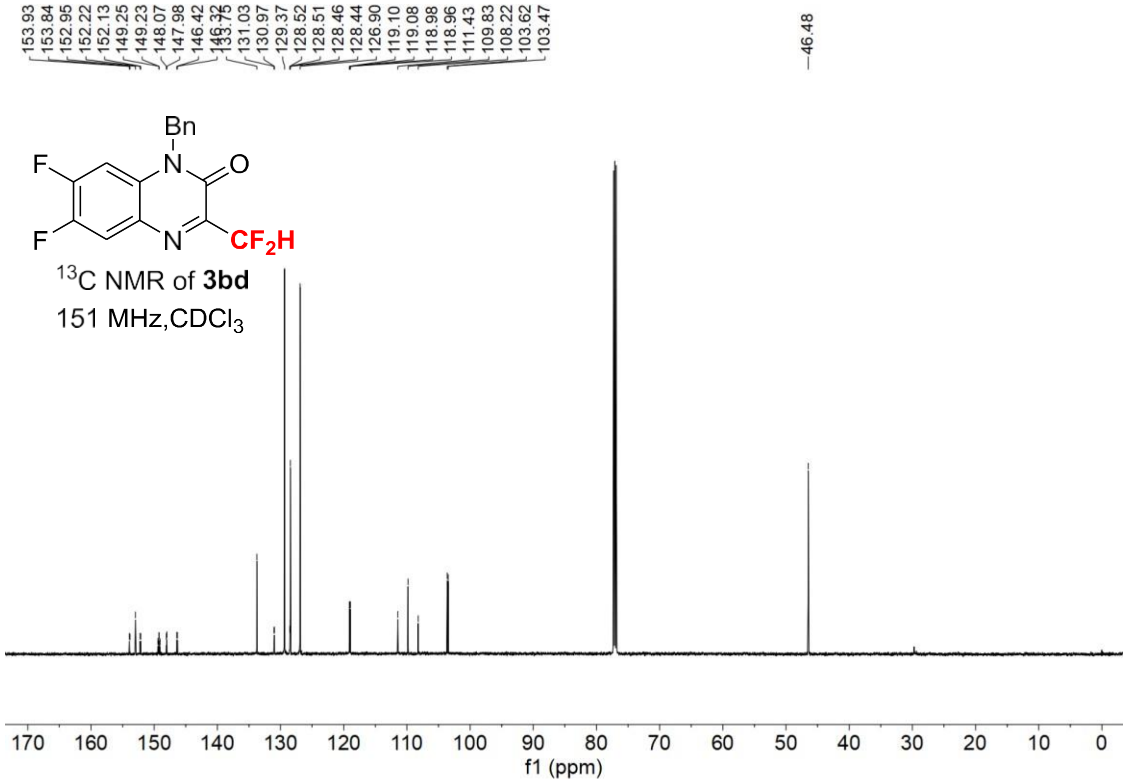

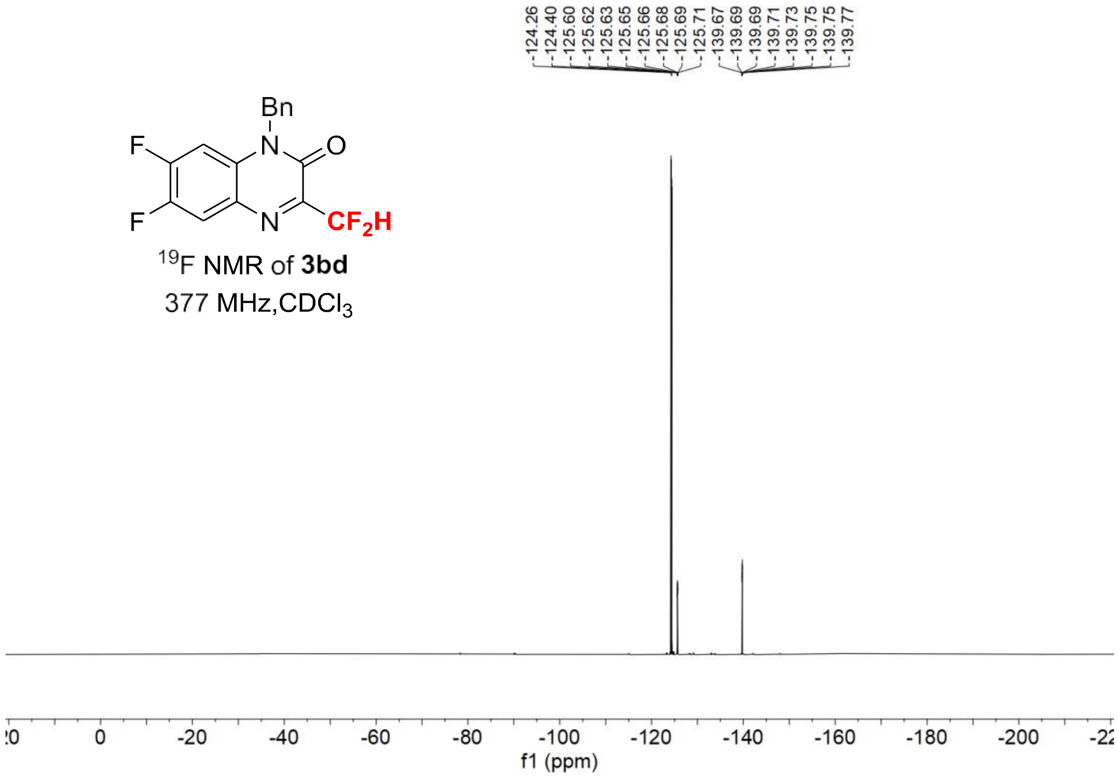

3-(difluoromethyl)-1-ethylquinoxalin-2(1*H*)-one (3be)

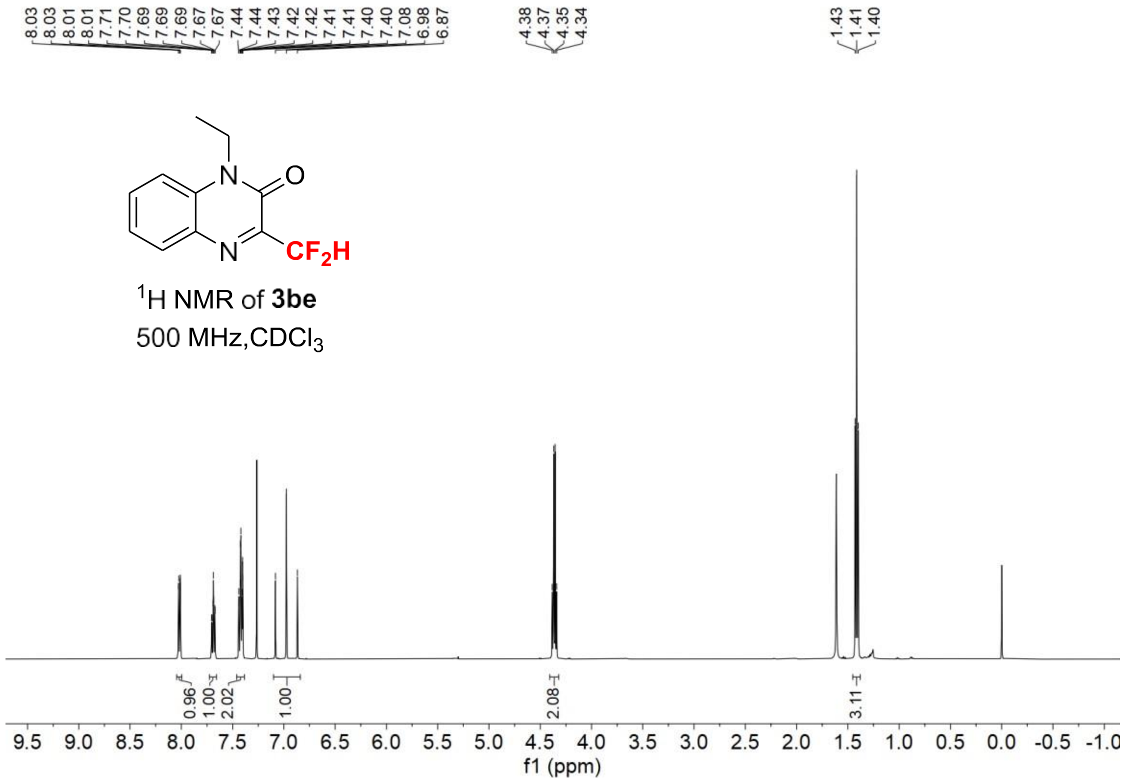
8.03
8.03
8.01
8.01
7.71
7.70
7.69
7.69
7.69
7.67
7.67
7.44
7.44
7.43
7.42
7.42
7.41
7.41
7.40
7.40
7.08
6.98
6.87
4.38
4.37
4.35
4.34
1.43
1.41
1.40
N
O
N
CF2H
1H NMR of 3be
500 MHz,CDCl3
0.96
1.00
2.02
1.00
2.08
3.11
9.5 9.0 8.5 8.0 7.5 7.0 6.5 6.0 5.5 5.0 4.5 4.0 3.5 3.0 2.5 2.0 1.5 1.0 0.5 0.0 -0.5 -1.0
f1 (ppm)

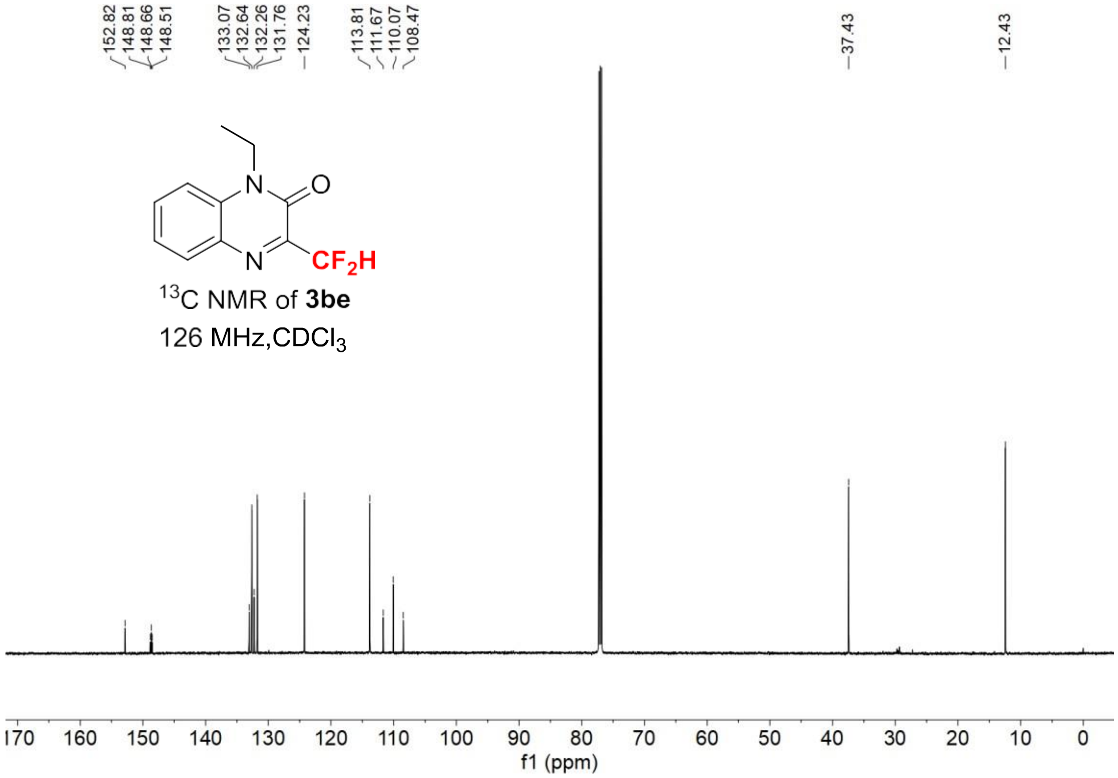
152.82
148.81
148.66
148.51
133.07
132.64
132.26
131.76
124.23
113.81
111.67
110.07
108.47
37.43
12.43
N
O
N
CF2H
13C NMR of 3be
126 MHz,CDCl3
170 160 150 140 130 120 110 100 90 80 70 60 50 40 30 20 10 0
f1 (ppm)

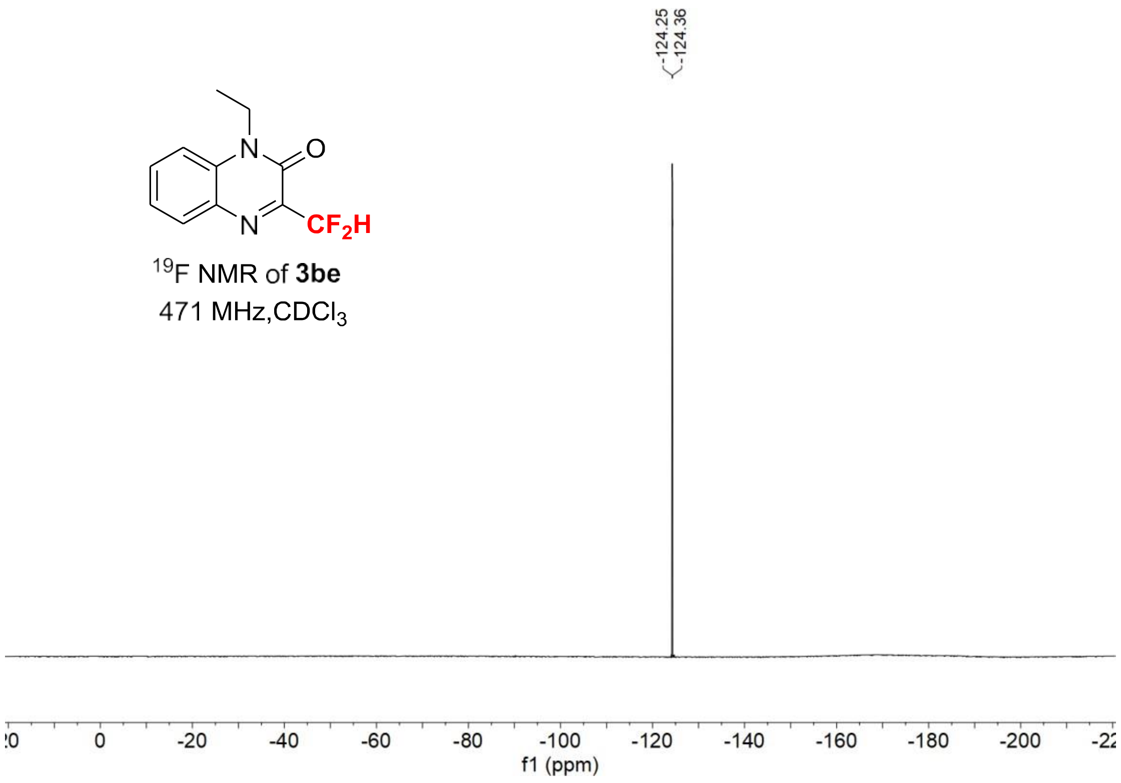

1-butyl-3-(difluoromethyl)quinoxalin-2(1*H*)-one (3bf)

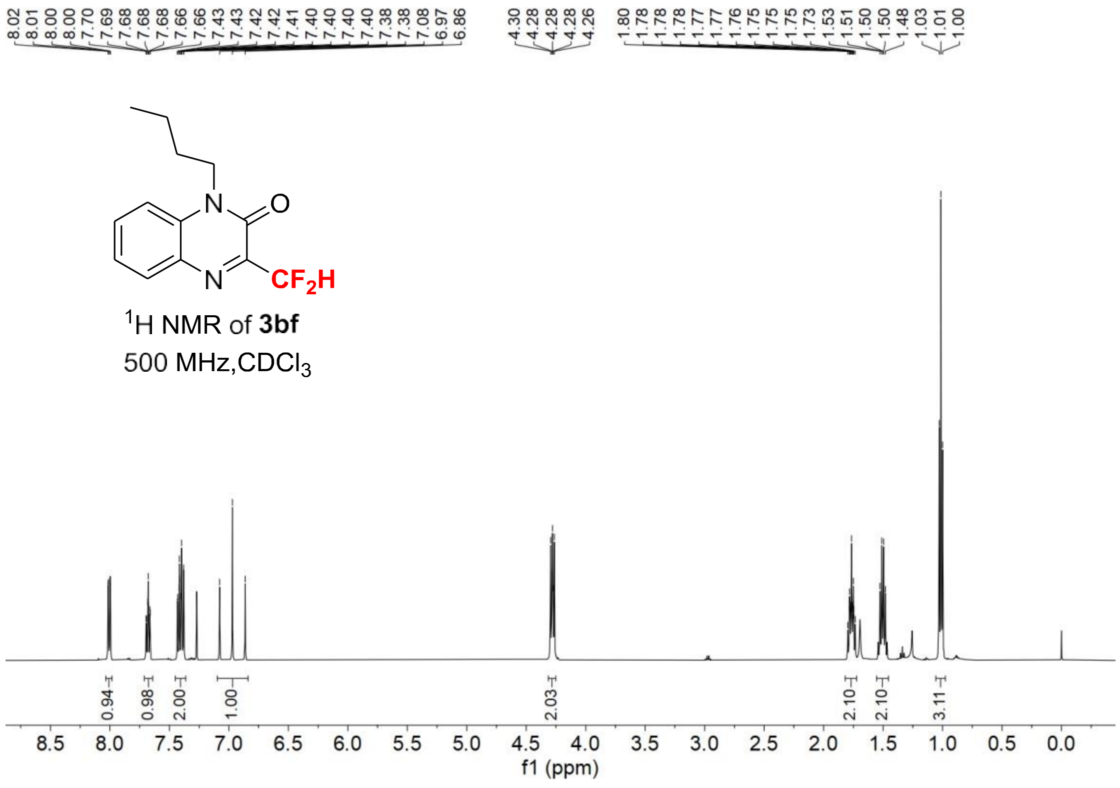

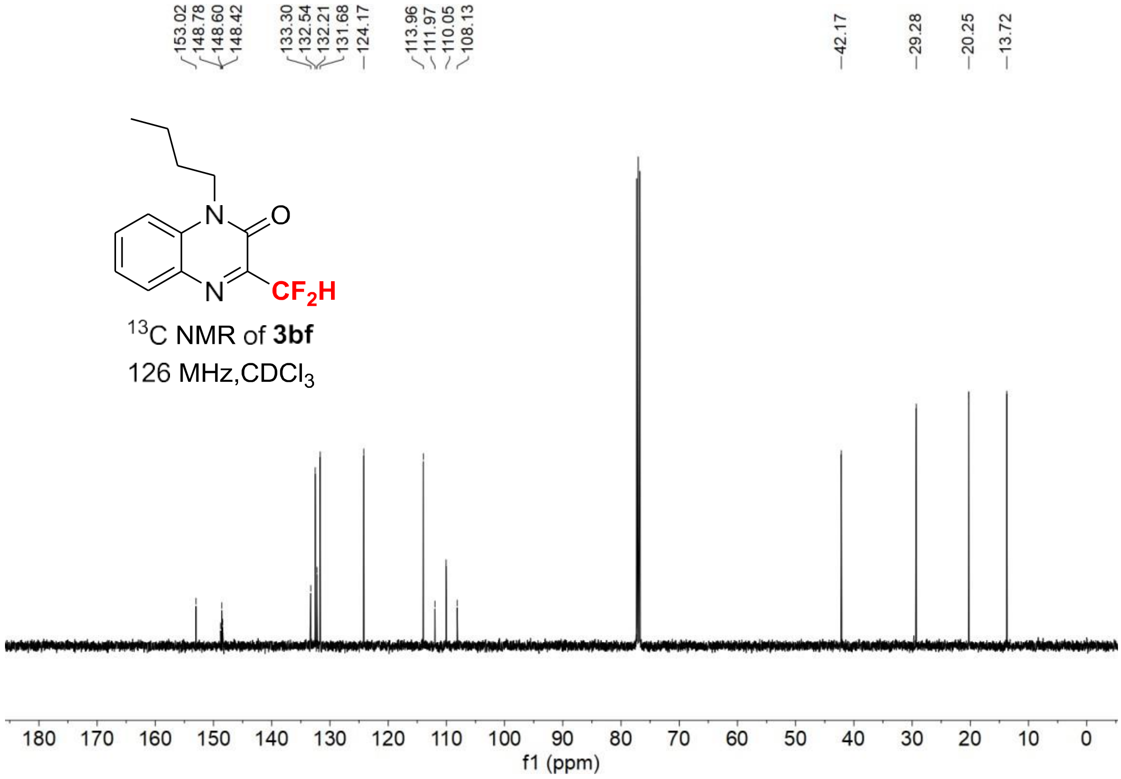

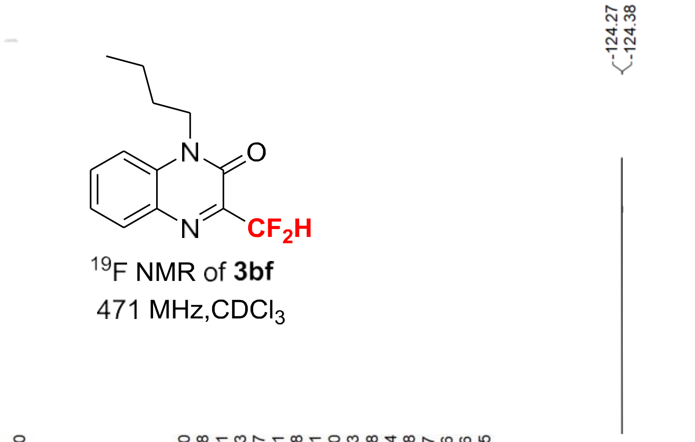

3-(difluoromethyl)-1-(2-oxo-2-phenylethyl)quinoxaline-2(1*H*)-one (3bg)

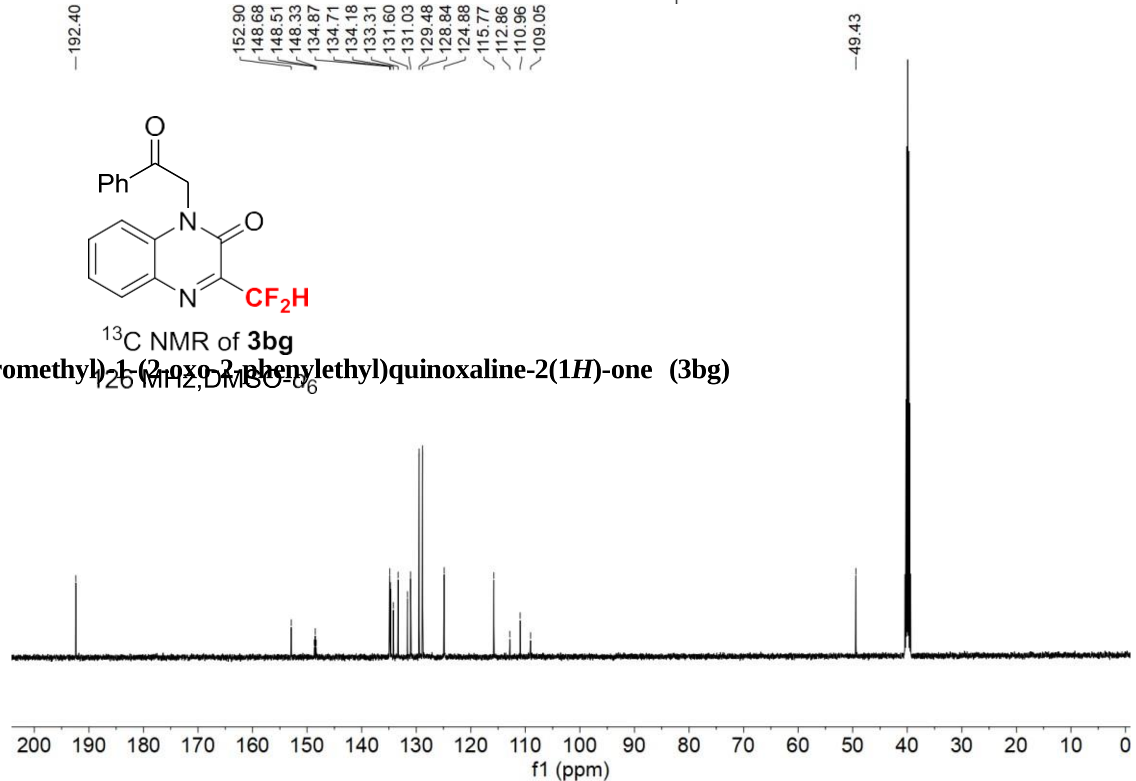

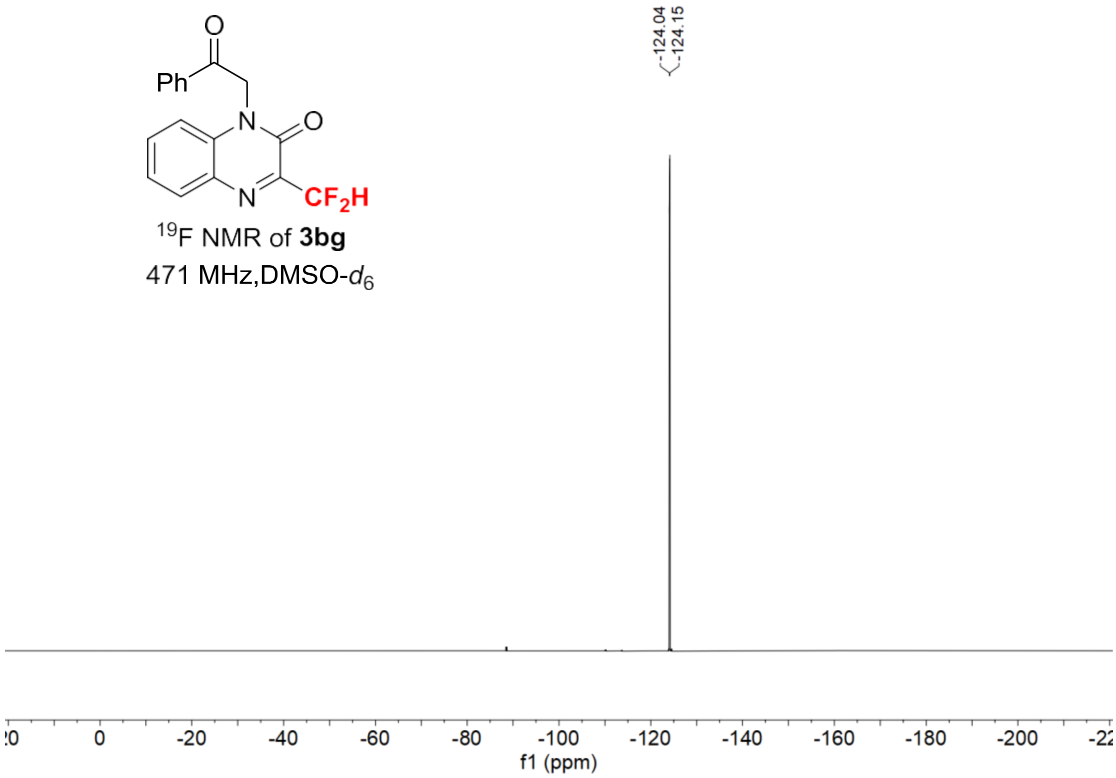

Ethyl 2-(3-(difluoromethyl)-2-oxoquinoxalin-1(2*H*)-yl)acetate (3bh)

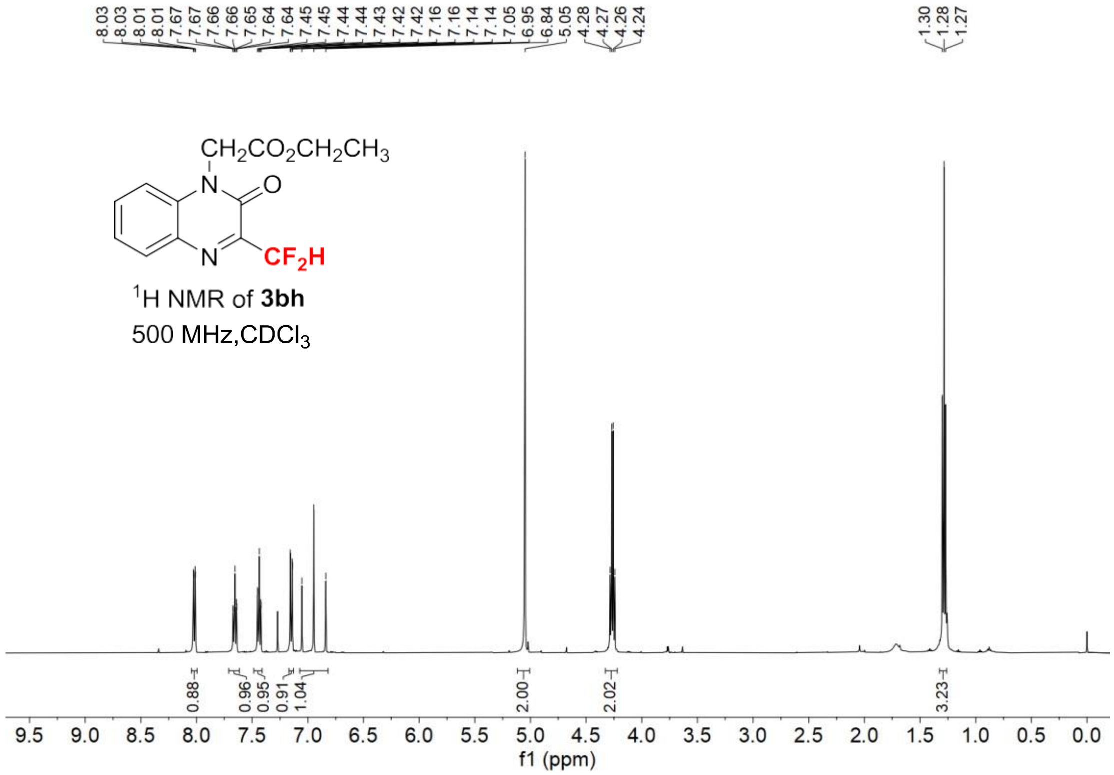

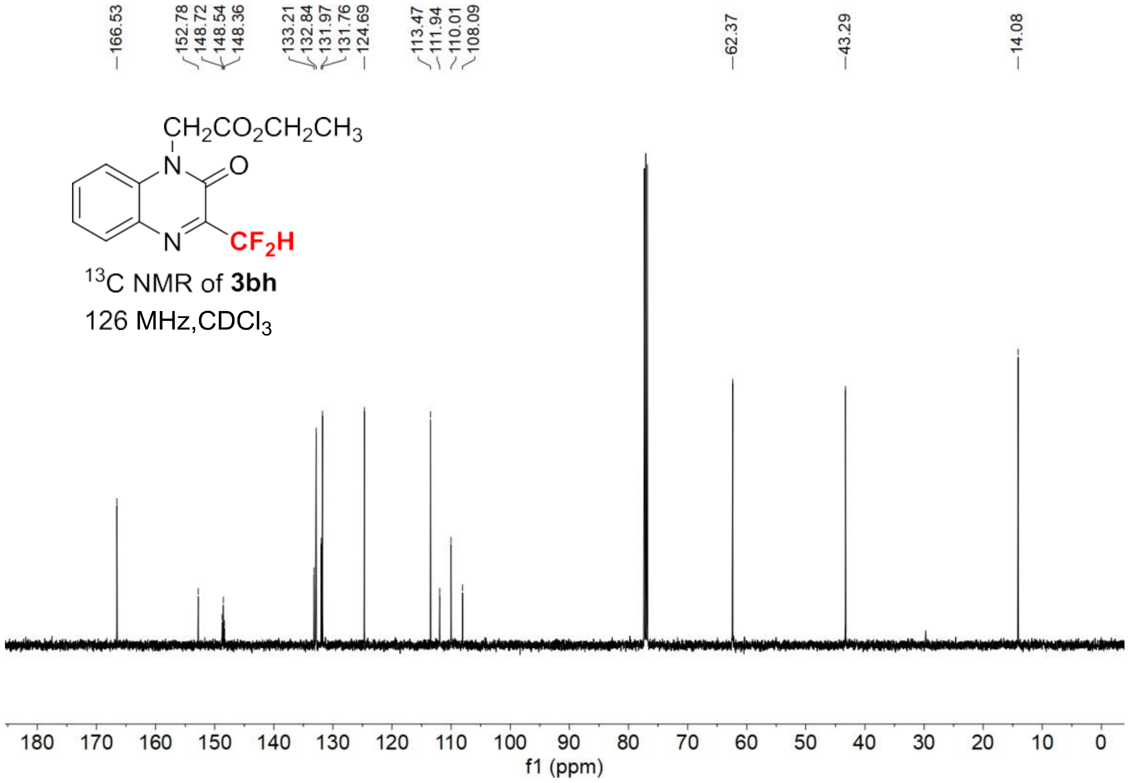

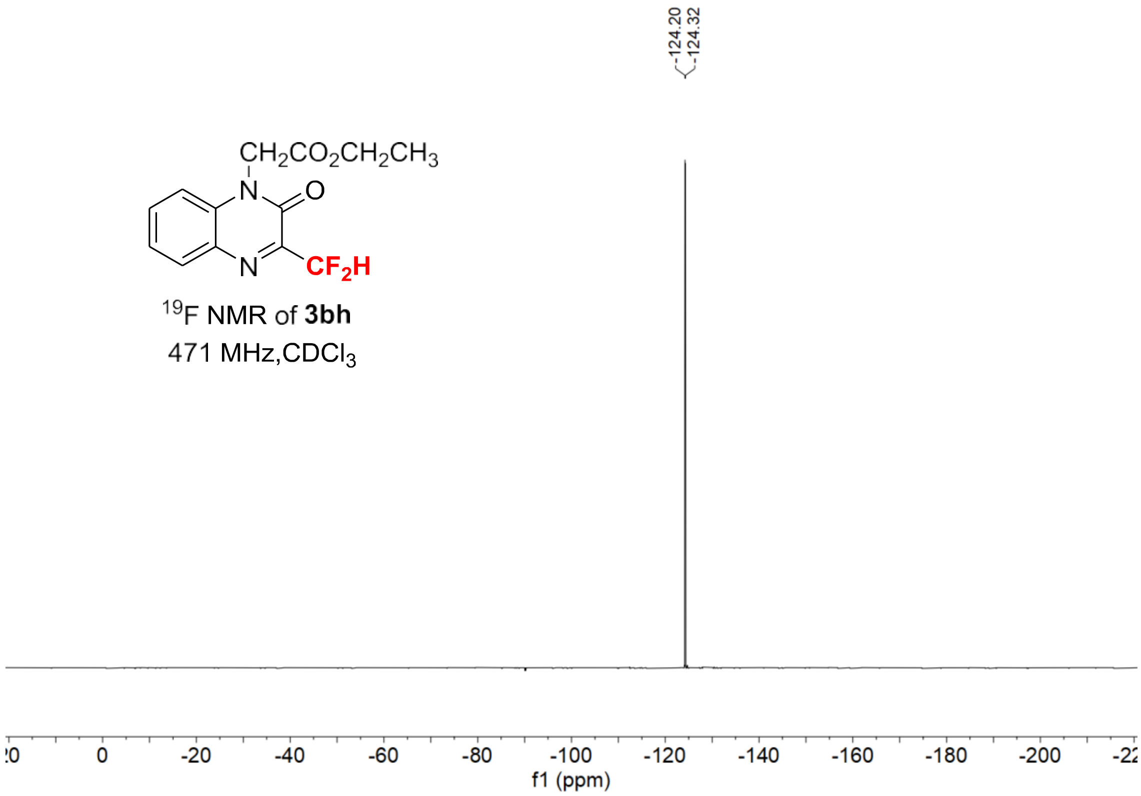

Isopropyl 2-(3-(difluoromethyl)-2-oxoquinoxalin-1(2*H*)-yl)acetate (3bi)

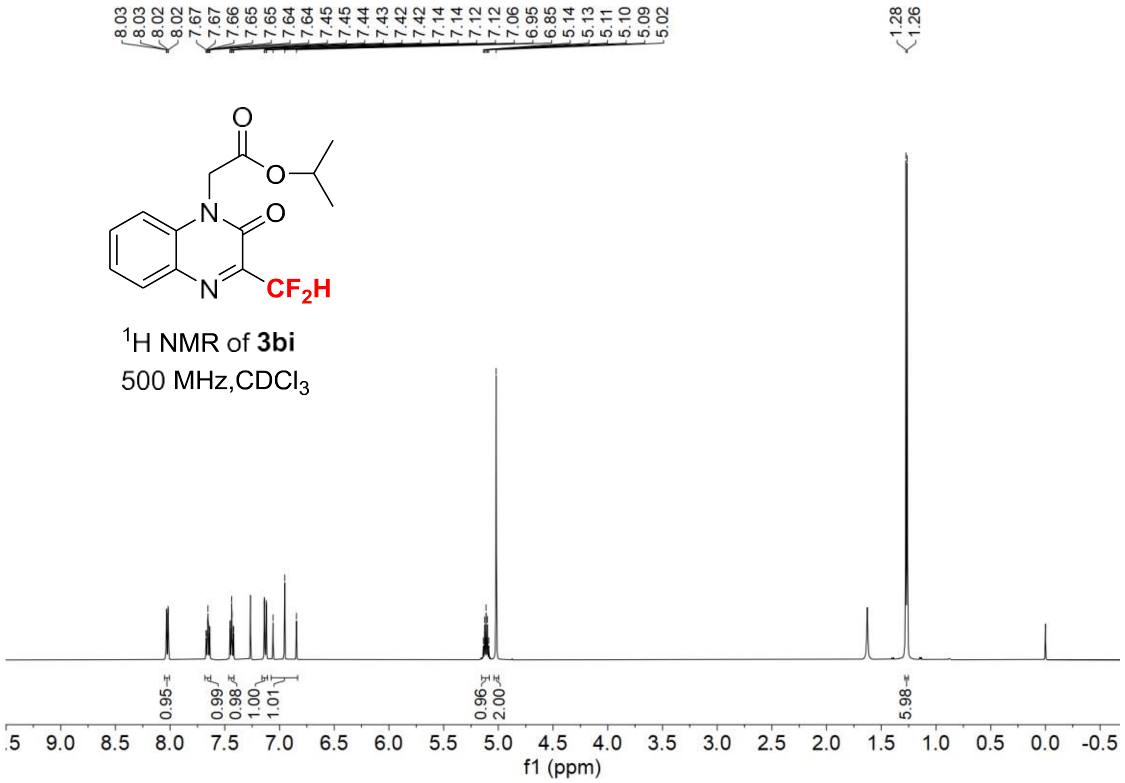
8.03
8.03
8.02
8.02
7.67
7.67
7.66
7.65
7.65
7.64
7.64
7.45
7.45
7.44
7.43
7.42
7.42
7.14
7.14
7.12
7.12
7.06
6.95
6.85
5.14
5.13
5.11
5.10
5.09
5.02
1.28
1.26
O
O
N
O
N
CF2H
1H NMR of 3bi
500 MHz,CDCl3
0.95
0.99
0.98
1.00
1.01
0.96
2.00
5.98
f1 (ppm)

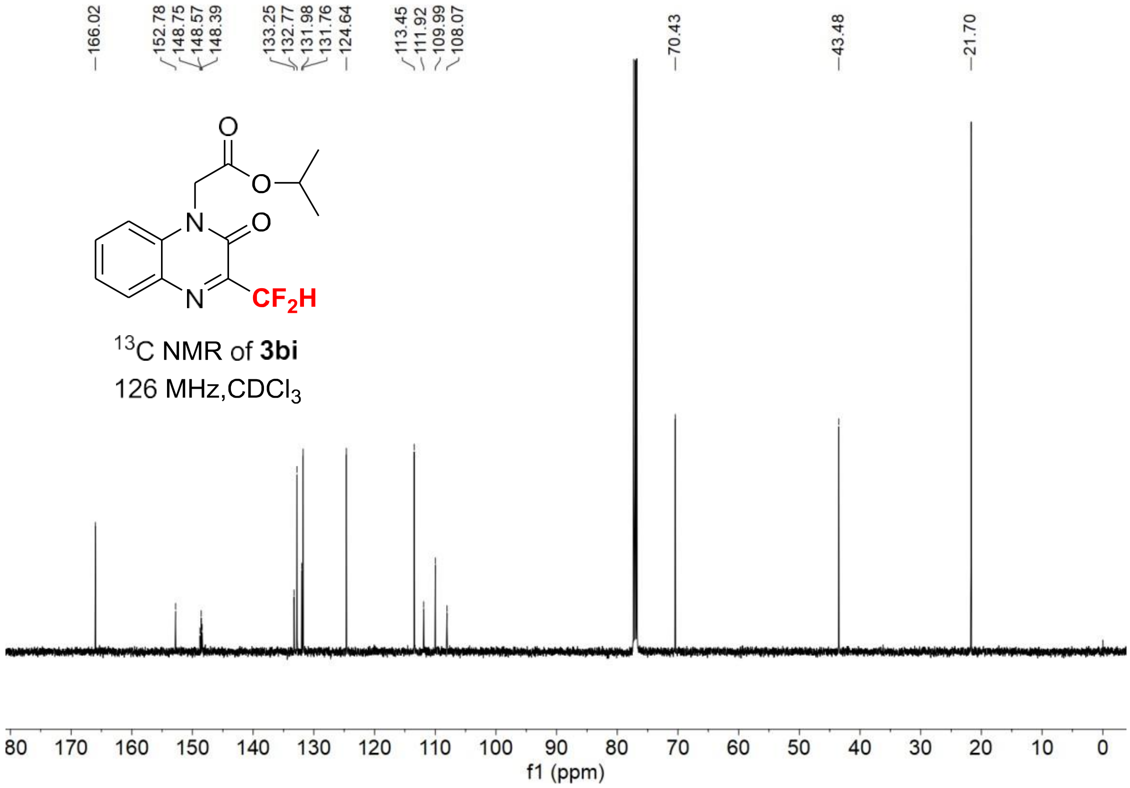
166.02
152.78
148.75
148.57
148.39
133.25
132.77
131.98
131.76
124.64
113.45
111.92
109.99
108.07
70.43
43.48
21.70
O
O
N
O
N
CF2H
13C NMR of 3bi
126 MHz,CDCl3
f1 (ppm)

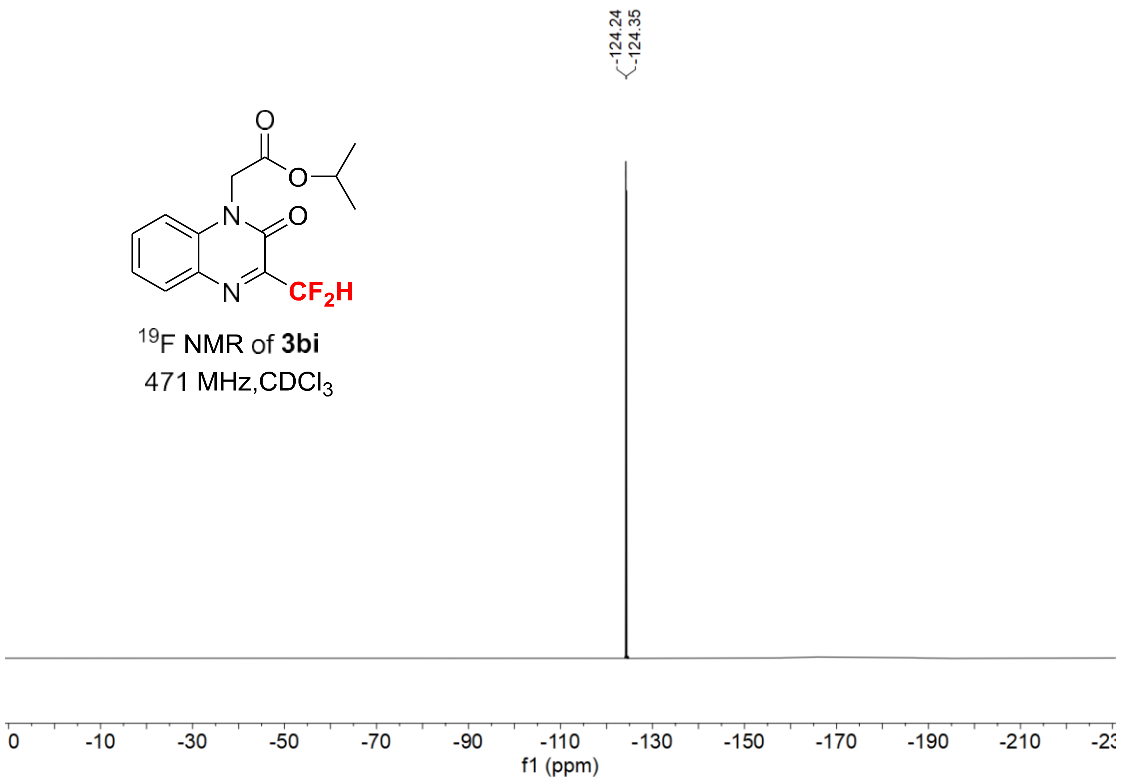

Tert-butyl 2-(3-(difluoromethyl)-2-oxoquinoxalin-1(2*H*)-yl) acetate (3bj)

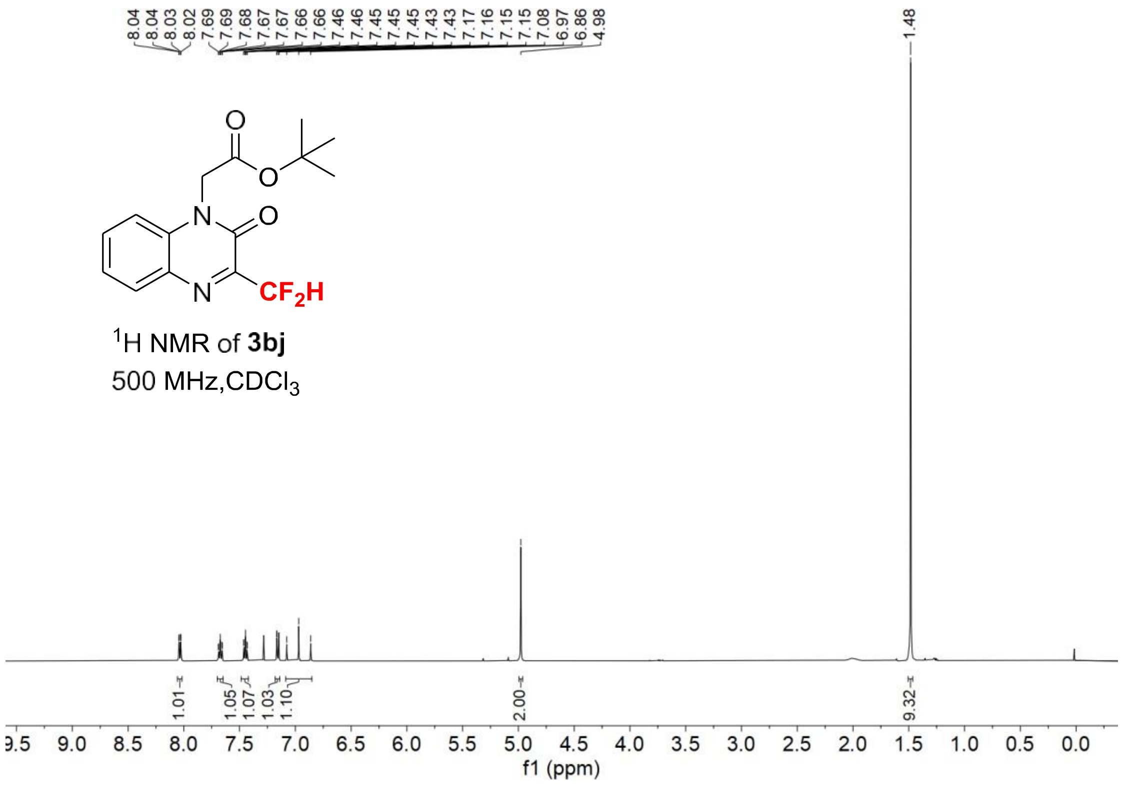

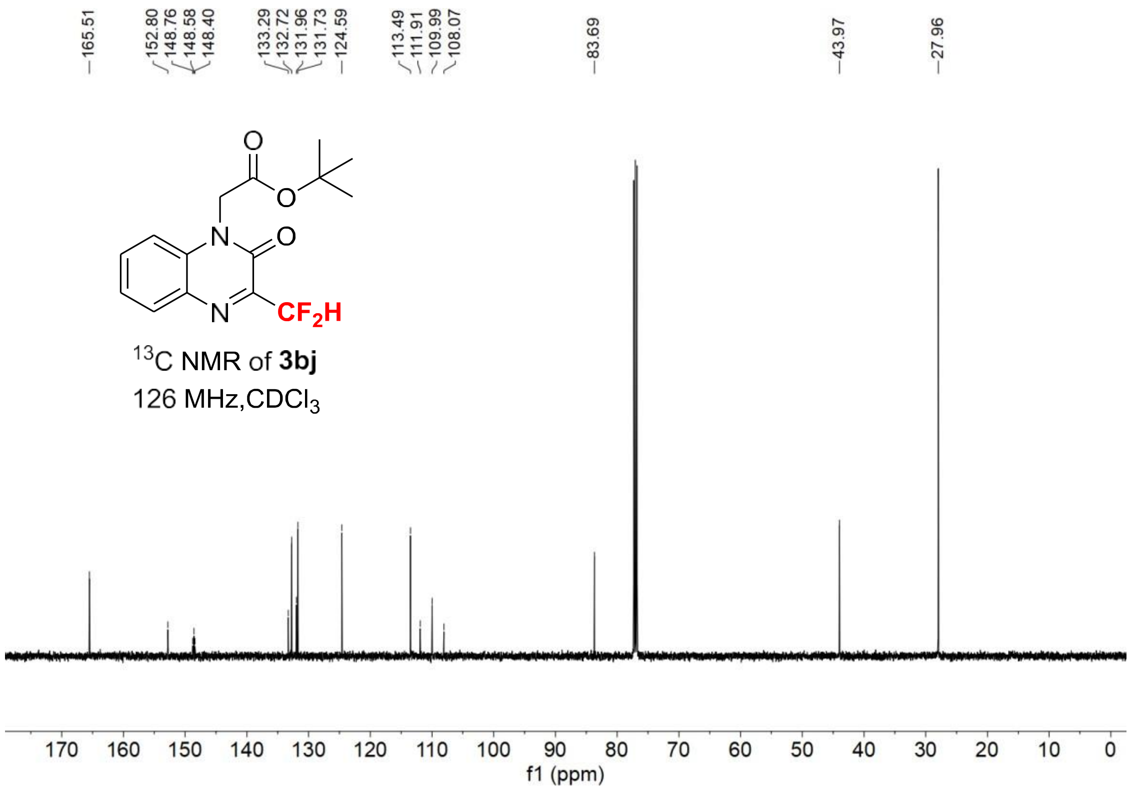

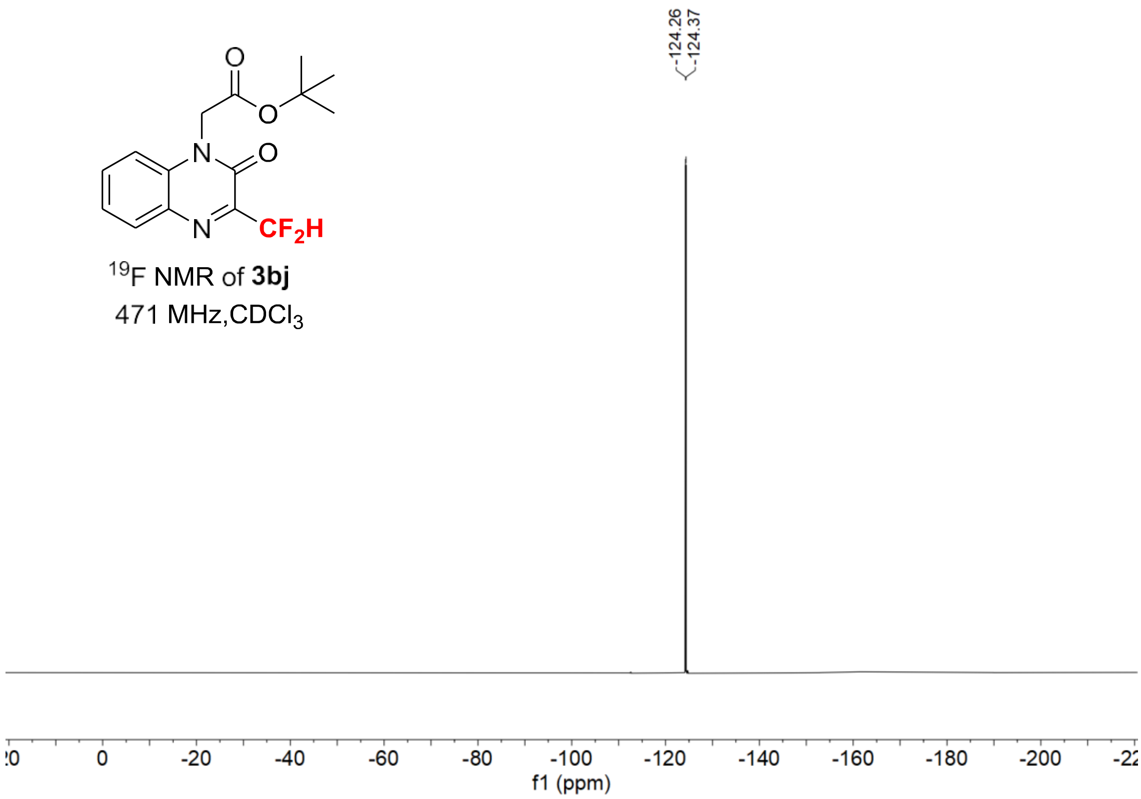

1-allyl-3-(difluoromethyl)quinoxalin-2(1*H*)-one (3bk)

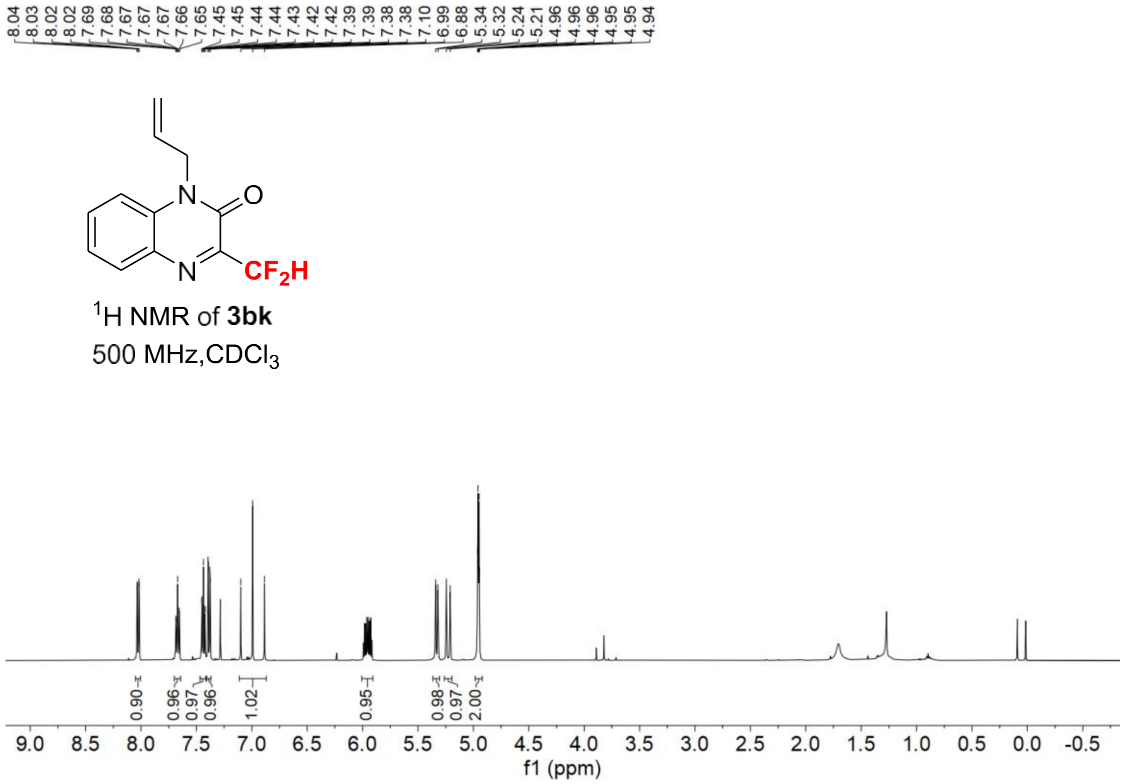
8.04
8.03
8.02
8.02
7.69
7.68
7.67
7.67
7.67
7.66
7.65
7.45
7.45
7.44
7.44
7.43
7.42
7.42
7.39
7.39
7.38
7.38
7.10
6.99
6.88
5.34
5.32
5.24
5.21
4.96
4.96
4.96
4.95
4.94
N
O
N
CF2H
1H NMR of 3bk
500 MHz,CDCl3
0.90
0.96
0.97
0.96
1.02
0.95
0.98
0.97
2.00
9.0 8.5 8.0 7.5 7.0 6.5 6.0 5.5 5.0 4.5 4.0 3.5 3.0 2.5 2.0 1.5 1.0 0.5 0.0 -0.5
f1 (ppm)

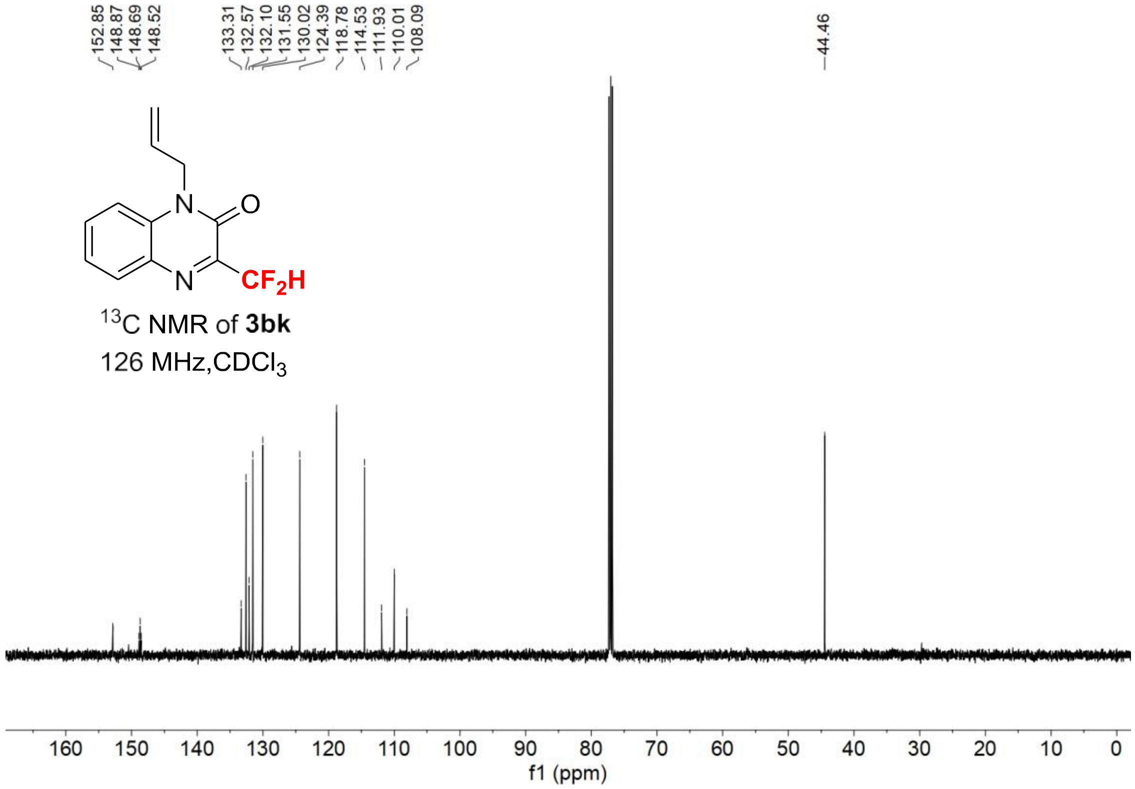
152.85
148.87
148.69
148.52
133.31
132.57
132.10
131.55
130.02
124.39
118.78
114.53
111.93
110.01
108.09
44.46
N
O
N
CF2H
13C NMR of 3bk
126 MHz,CDCl3
160 150 140 130 120 110 100 90 80 70 60 50 40 30 20 10 0
f1 (ppm)

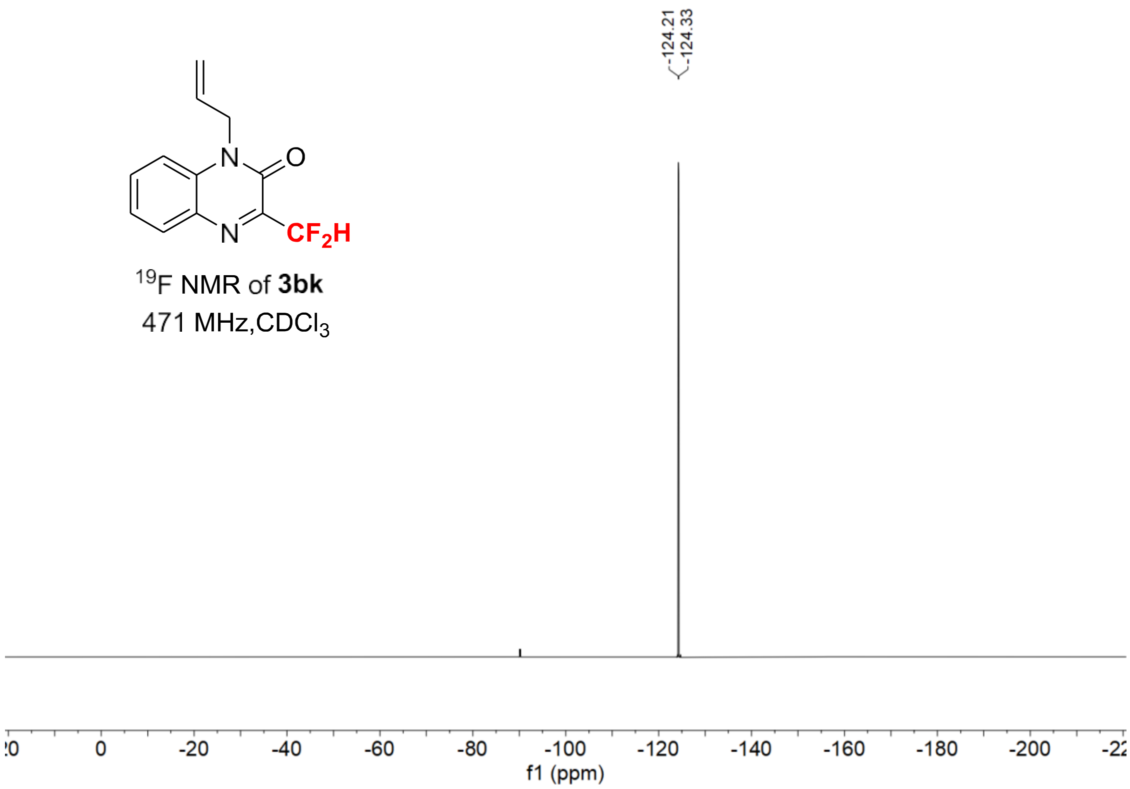

3-(difluoromethyl)-1-(prop-2-yn-1-yl)quinoxalin-2(1*H*)-one (3bl)

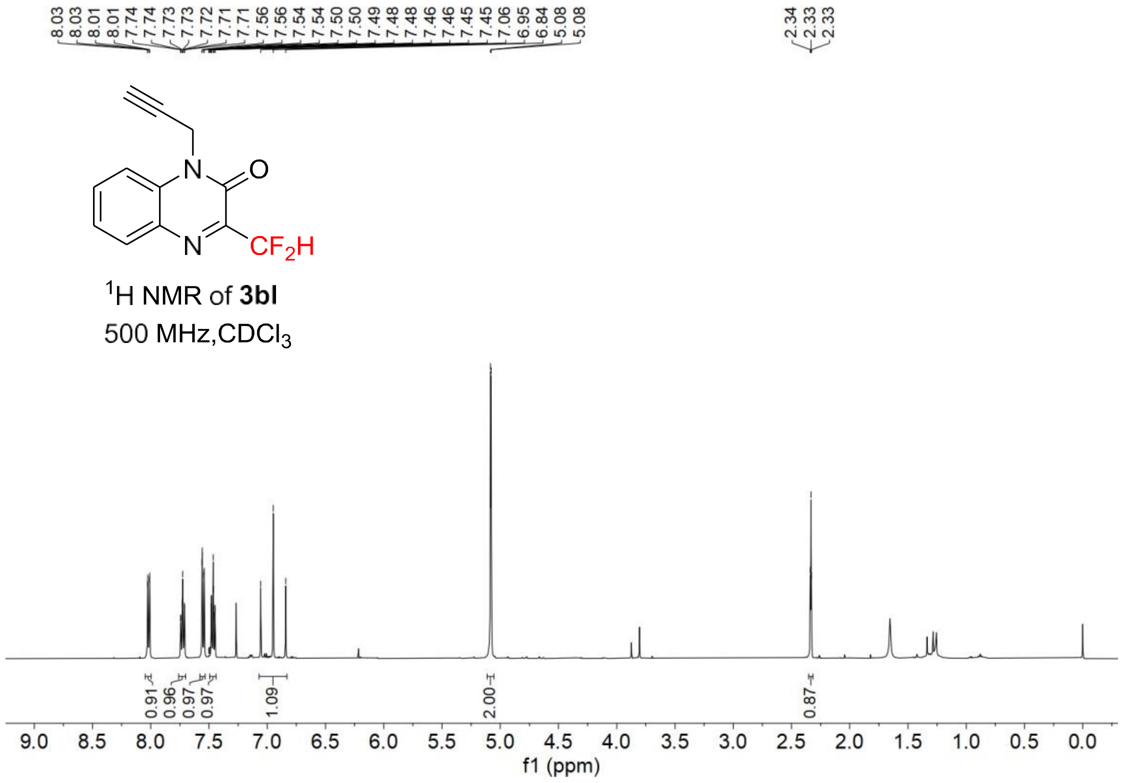

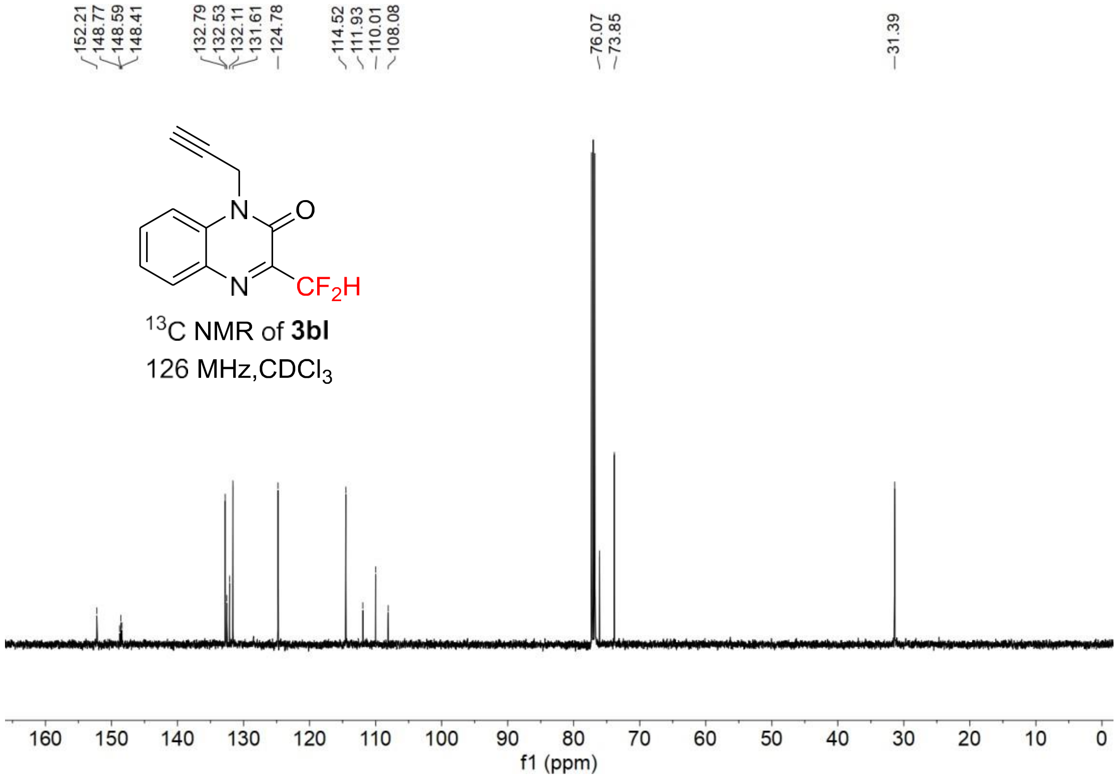

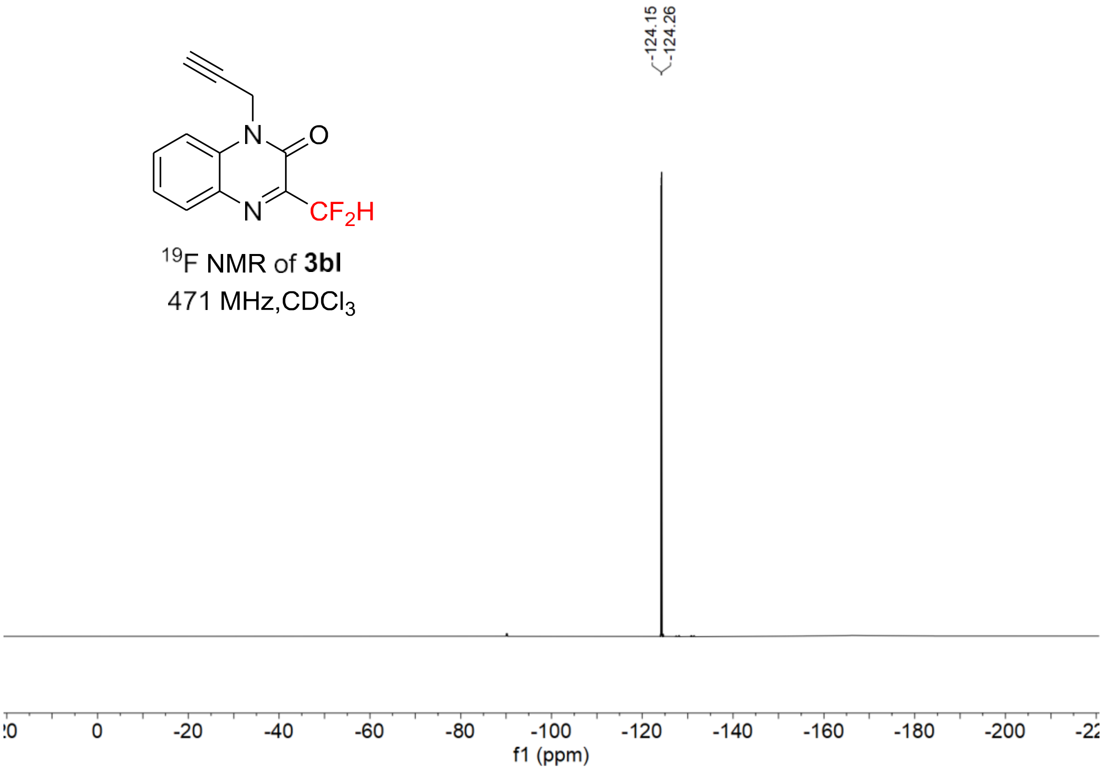

3-difluoromethyl-quinoxaline-2-thiol（5）

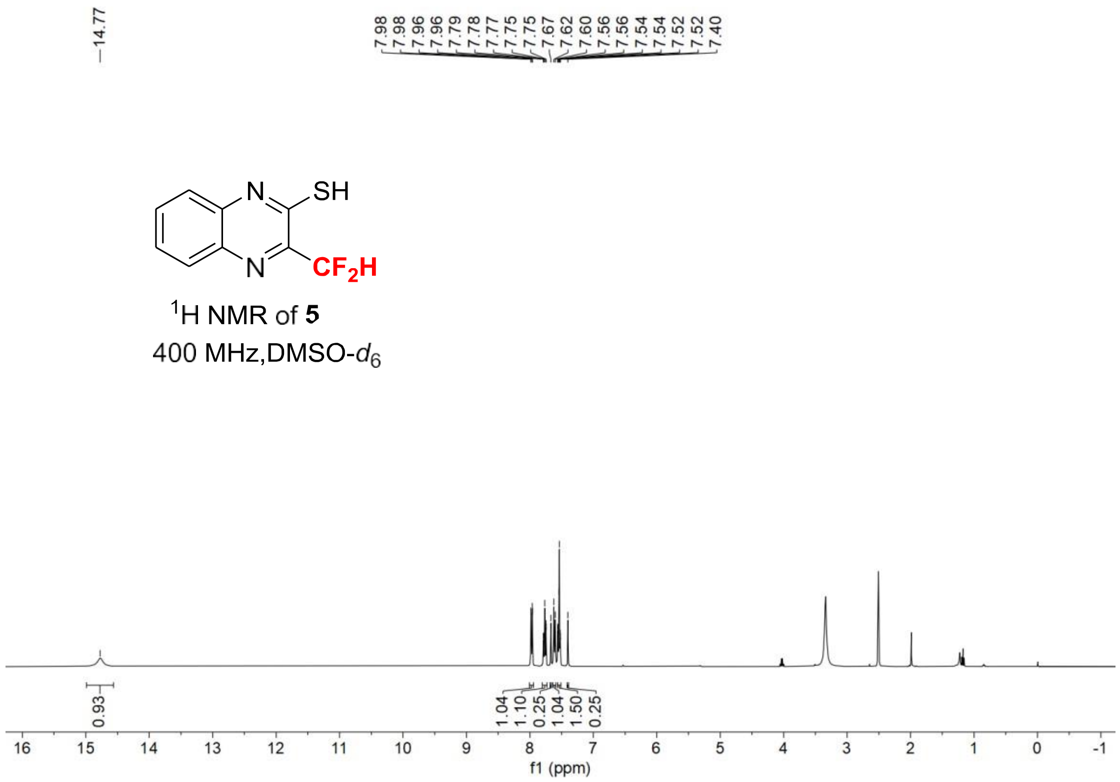
14.77
7.98
7.98
7.96
7.96
7.79
7.78
7.77
7.75
7.75
7.67
7.62
7.60
7.56
7.56
7.54
7.52
7.52
7.40
N
SH
N
CF2H
1H NMR of 5
400 MHz,DMSO-d6
0.93
1.04
1.10
0.25
1.04
1.50
0.25
f1 (ppm)

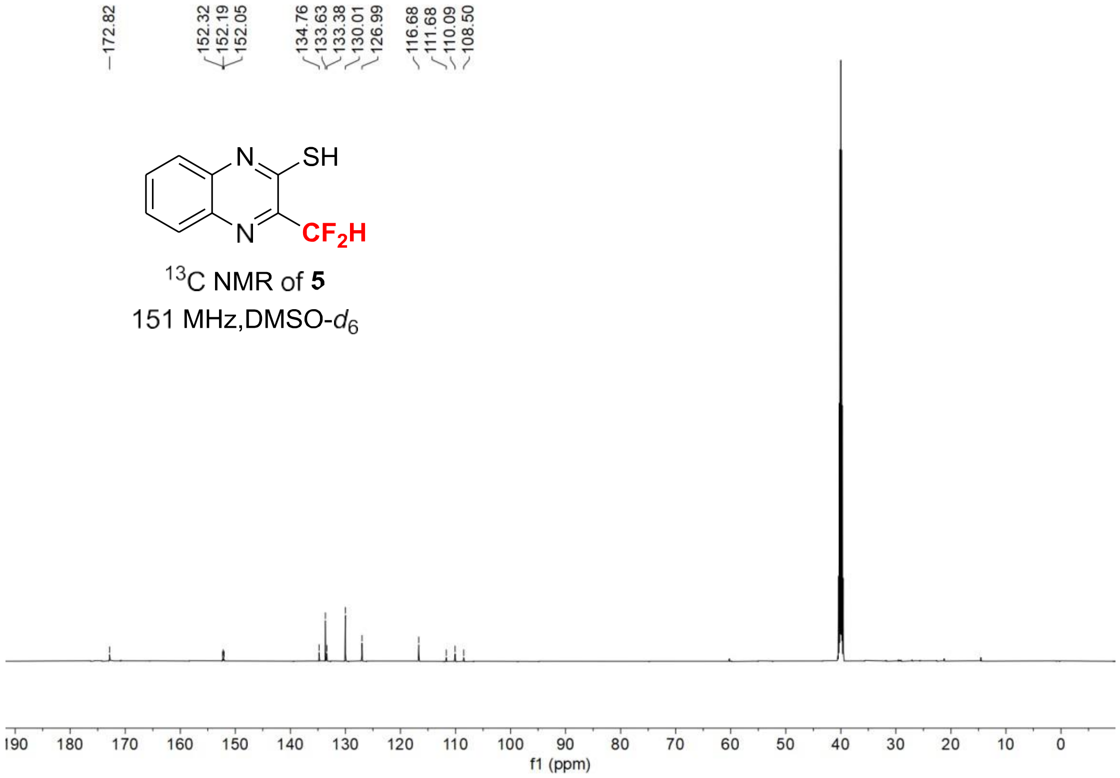
172.82
152.32
152.19
152.05
134.76
133.63
133.38
130.01
126.99
116.68
111.68
110.09
108.50
N
SH
N
CF2H
13C NMR of 5
151 MHz,DMSO-d6
f1 (ppm)

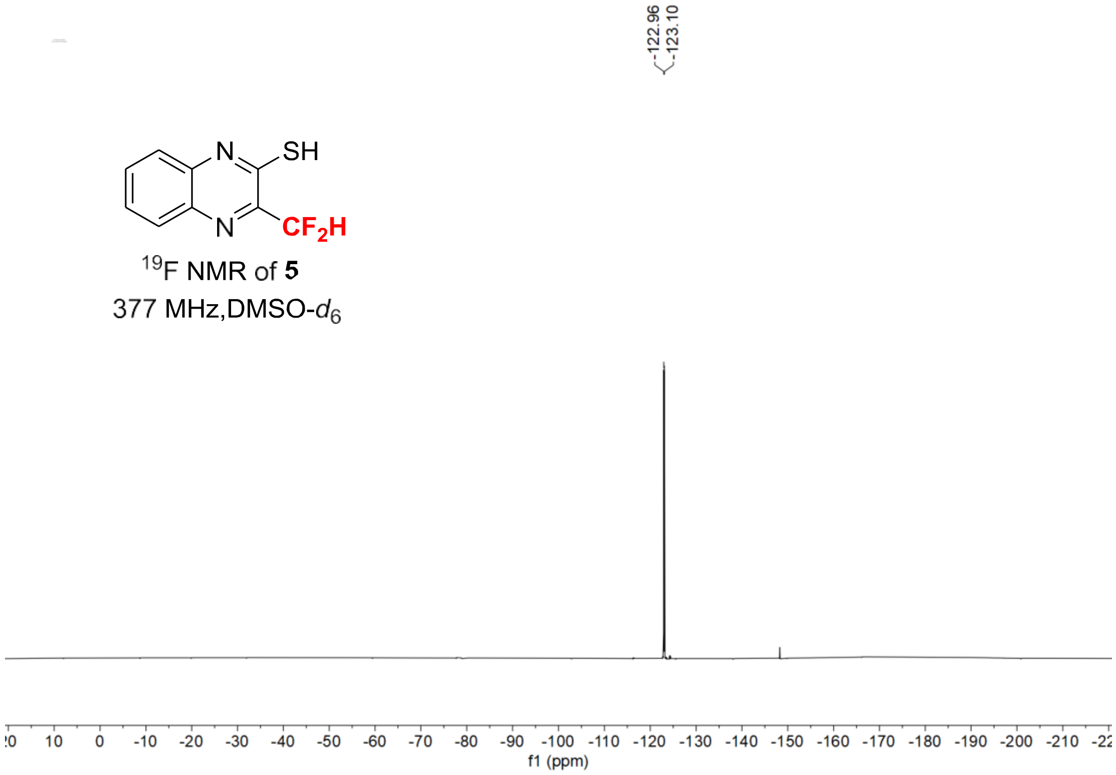

1-(2-methyl-phenyl)-3-[2-(3-Difluoromethyl-quinoxalin-2-ylsulfanyl)-acetyl]-urea (7)

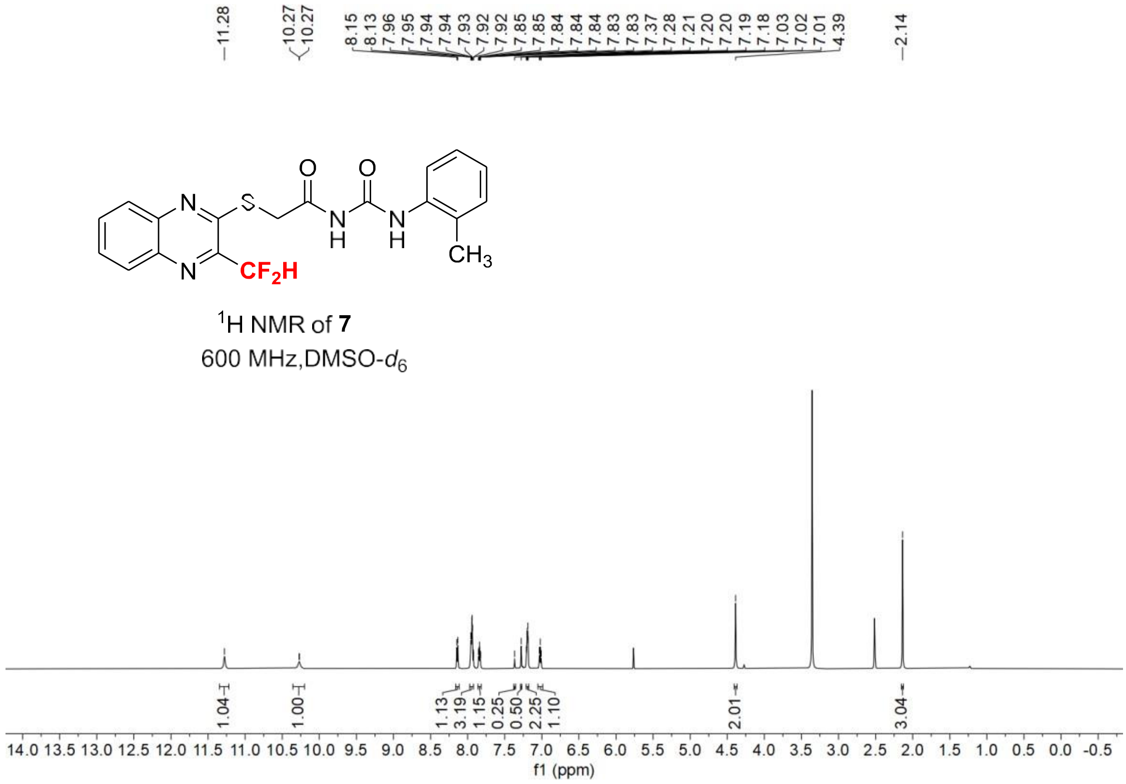

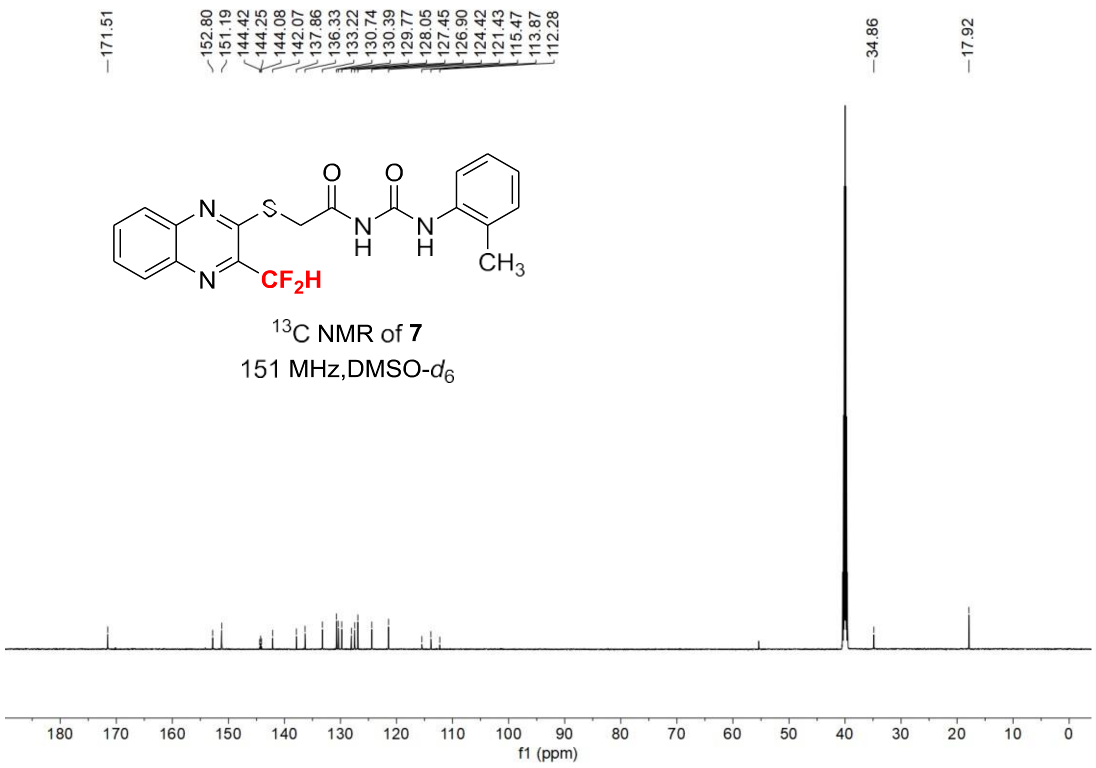

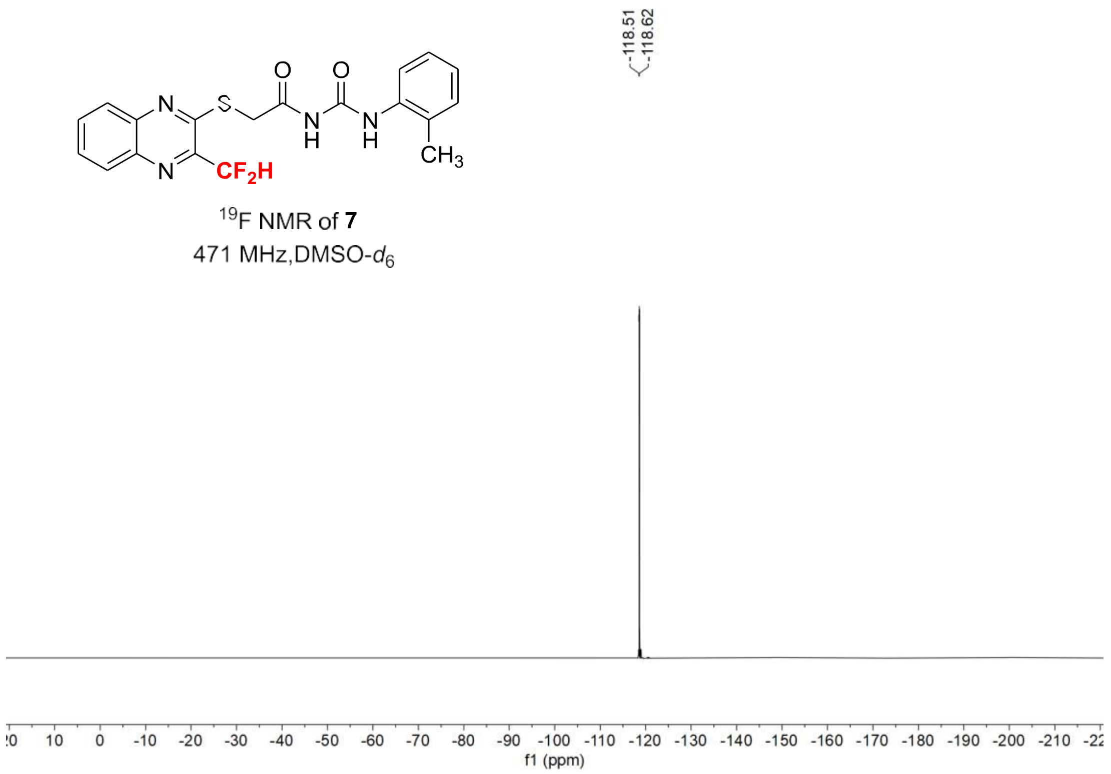

7-chloro-4-(2,2-difluoroethyl)-1,2-dihydronaphthalene (9).

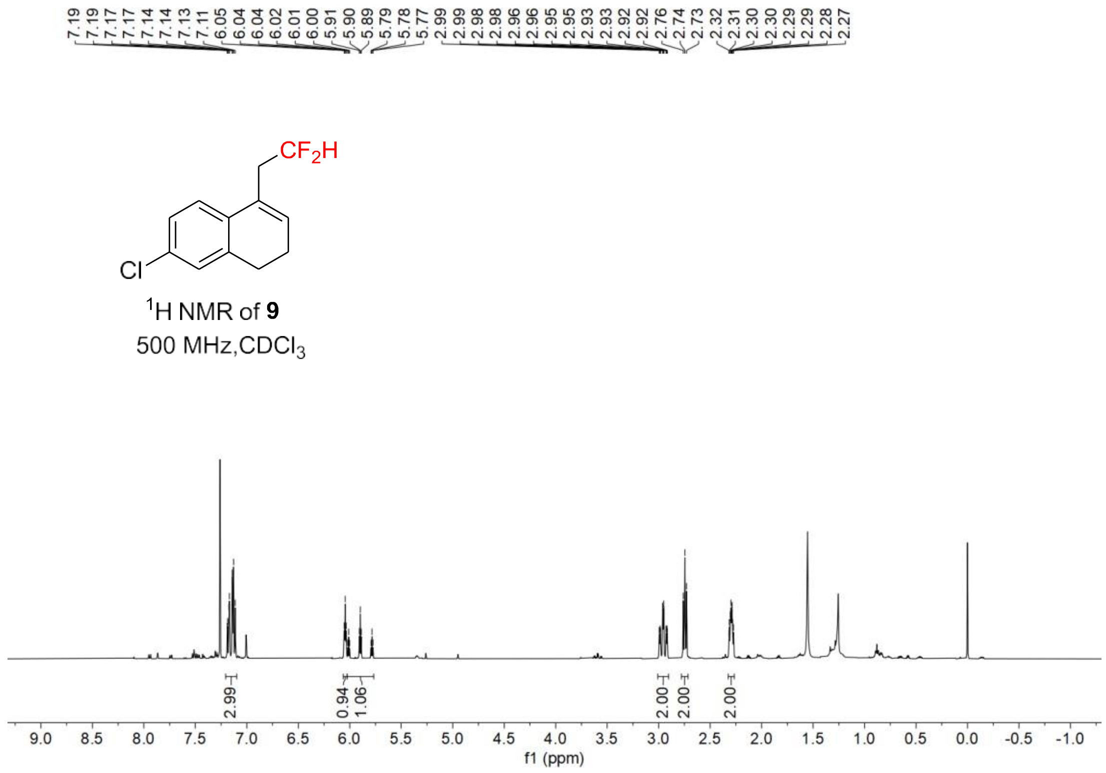

^{1}H NMR of **9**

500 MHz, $CDCl_3$

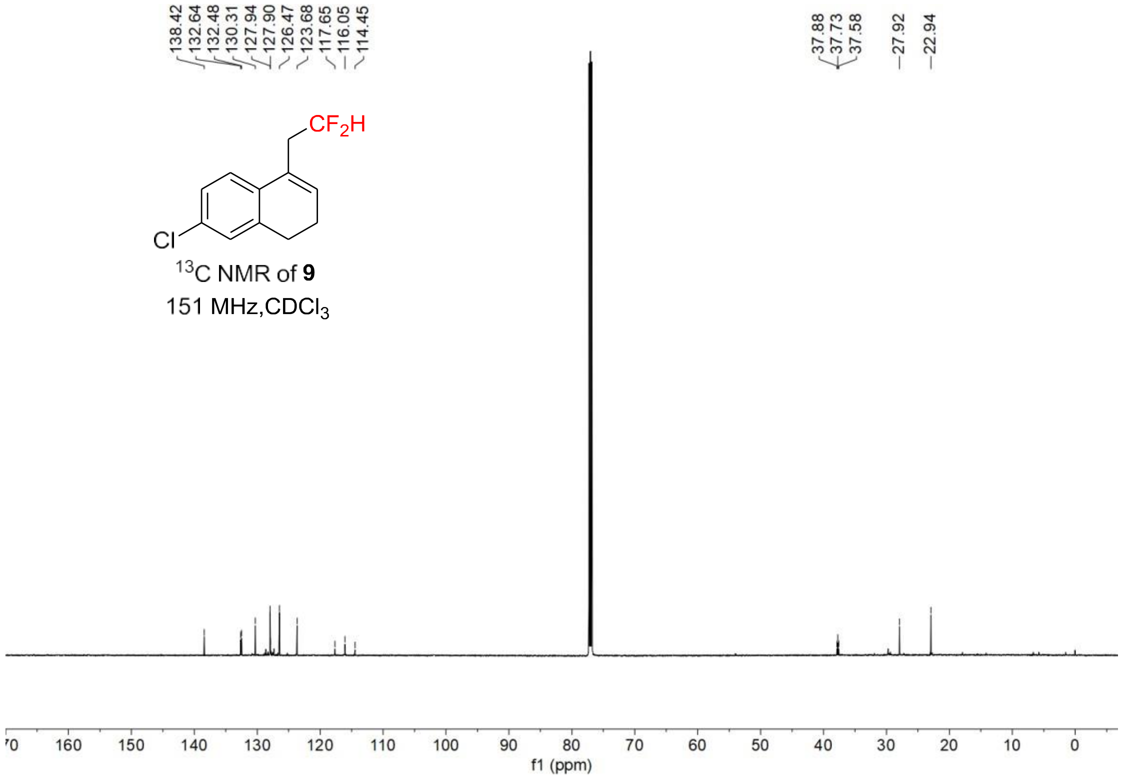

^{13}C NMR of **9**

151 MHz, $CDCl_3$

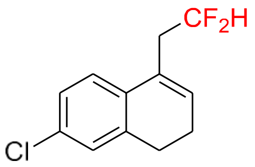

^{19}F NMR of **9**

471 MHz, $CDCl_3$

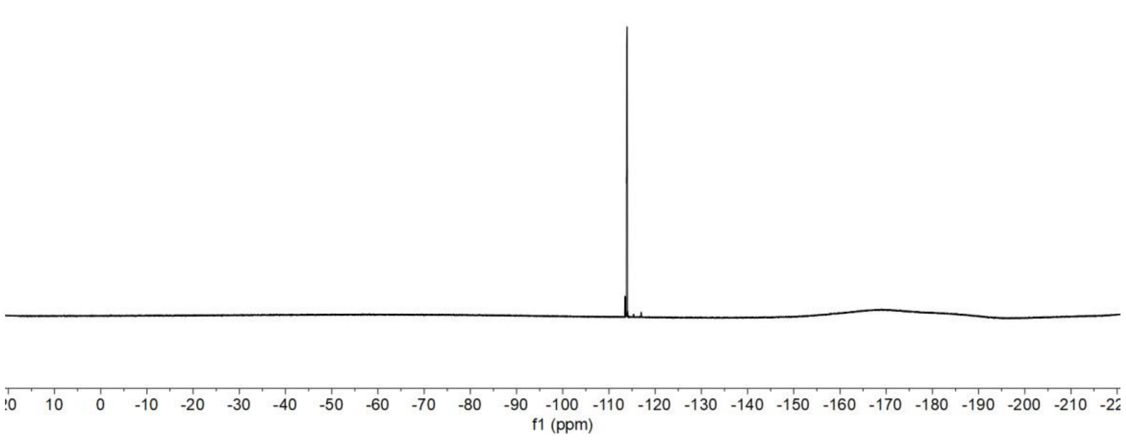

Supplement: Supplementary file 1 [file pharmaceuticals-15-01552-s001.zip › pharmaceuticals-2053659-supplementary.pdf]
